# Comprehensive Evaluation of Oxidation Resistance for Tertiary Phosphines Using Universal Machine Learning Interatomic Potential-Aided Molecular Dynamics Simulation

**DOI:** 10.1021/jacsau.6c00783

**Published:** 2026-08-23

**Authors:** Toshiya Sato, Yuri Asai, Uika Koshimizu, Yuji Hakozaki, Kenshin Takei, Kazuki Mori, Shunsaku Yasumura, Tatsushi Ikeda, Yoshihiro Yayama

**Affiliations:** † Central Technical Research Laboratory, 358032 ENEOS Corporation, 8 Chidoricho, Naka-ku, Yokohama, Kanagawa 231-0815, Japan; ‡ AI Innovation Department, ENEOS Holdings, Inc., 8 Chidoricho, Naka-ku, Yokohama, Kanagawa 231-0815, Japan; § Department of Chemical System Engineering, 13143 The University of Tokyo, Tokyo 113-8656, Japan; ∥ Institute of Industrial Science, 13143 The University of Tokyo, Komaba 4-6-1, Meguro, Tokyo 153-8505, Japan

**Keywords:** phosphine, oxidation resistance, universal machine learning interatomic potential (uMLIP), Matlantis PFP, molecular dynamics (MD), survival analysis

## Abstract

Tertiary phosphines play important roles in a wide range of chemical reactions, but they suffer from oxidation in air. Here, we propose a computational workflow for the comprehensive prediction of oxidation resistance in tertiary phosphines based on molecular dynamics (MD) simulations using Matlantis PFP, a universal machine learning interatomic potential (uMLIP). MD simulations were conducted for 51 different tertiary phosphines and analyzed to estimate mean survival time by survival analysis (τ). The resulting τ reproduced experimentally and theoretically recognized trends in oxidation resistance across chemically diverse phosphines. In particular, it captured electronic, steric, and aromatic effects on oxidation resistance, even in systems where these factors are intertwined. Comparisons with reported oxidation behaviors further showed that τ correctly identifies both highly air-sensitive and highly air-stable phosphines. These results demonstrate that the present workflow provides a practical, generalizable tool for pre-experimental assessment of phosphine oxidation resistance and for more rational ligand design.

## Graphical abstract

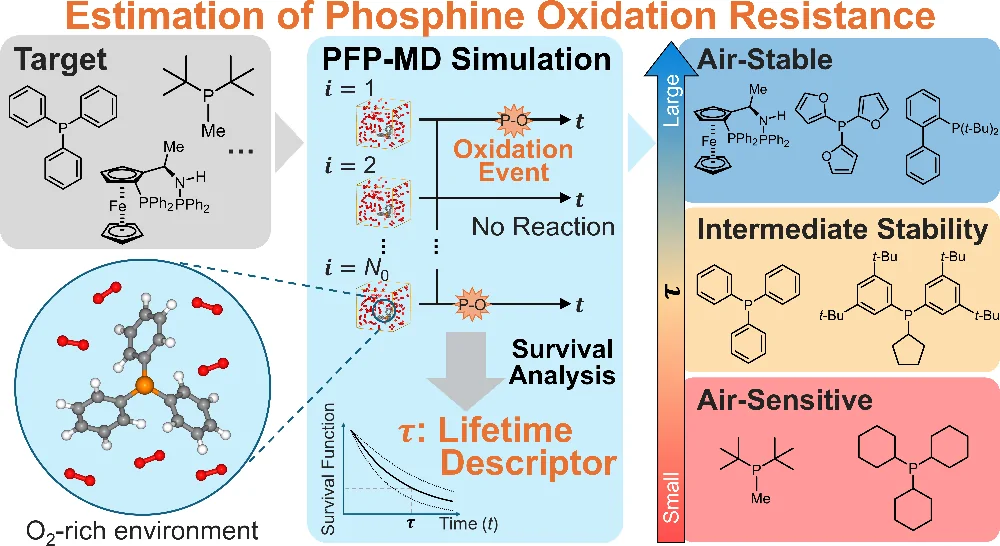

## 1 Introduction

Tertiary phosphines are widely used in homogeneous catalysis because of their high reactivity and strong ability to promote a broad range of reactions. A notable example is cross-coupling chemistry, including Suzuki–Miyaura,^1^ Stille,^2^ Negishi,^3,4^ and Buchwald–Hartwig reactions,^5,6^ which enable the formation of diverse carbon–carbon and carbon–heteroatom bonds. These transformations allow for the synthesis of complex molecules in fewer steps, thus making them essential to produce pharmaceuticals, liquid-crystal materials, organic light-emitting diode (OLED) materials, and photovoltaic materials, conferring substantial industrial value.^7^

On the other hand, numerous tertiary phosphines exhibit limited resistance to oxidation and are readily oxidized when exposed to air, complicating their isolation and handling.^8^ This instability raises a major practical challenge in ligand development: even when a newly designed phosphine affords excellent catalytic performance, poor air stability can severely restrict its applicability and necessitate additional effort for safe synthesis, purification, storage, and operation under inert conditions. Consequently, significant research cost and development time may be spent on ligands that prove unsuitable for practical use because of insufficient oxidation resistance.

Several empirical trends regarding the oxidation resistance of tertiary phosphines have been reported. For example, it has been reported that tertiary phosphines with biaryl frameworks exhibit improved oxidation resistance because they are less likely to adopt A-away conformations that readily react with molecular oxygen.^9^ Exceptionally air-stable tertiary phosphines have also been reported, such as BoPhoz ligands, which do not decompose even after being stored in air for three years,^10^ and triisobutylene (TIB)-derived ligands, which remain unoxidized even after being left in toluene solution for 1 week.^11^ Although knowledge regarding the oxidation resistance of tertiary phosphines with specific structural features is steadily accumulating, comprehensive and systematic trends across a broad range of tertiary phosphines remain unclear. If oxidation resistance could be predicted prior to synthesis, it would enable the evaluation of stability at the ligand-design stage, representing a valuable and impactful advance in catalyst development.

Computational chemistry based on quantum-chemical calculations has been widely used to predict catalytic activity and selectivity prior to synthesis. Transition-state (TS) calculations have enabled mechanistic understanding and quantitative prediction of reaction barriers, allowing rational screening of catalyst candidates.^12^ In contrast, only a limited number of studies aimed at predicting oxidation resistance have been conducted. One reason is that oxidation resistance is governed not only by the intrinsic reactivity toward molecular oxygen but also by a complex interplay of steric, electronic, and conformational factors within the ligand framework.

Molecular dynamics (MD) simulation is one of the most effective approaches for predicting oxidation resistance time prior to synthesis. Reactive MD simulations have been conducted to elucidate dynamic properties associated with chemical reactions; for example, ReaxFF-based simulations have been applied to model combustion reactions in systems containing large numbers of oxygen and water molecules surrounding lignite or ethyl acetate.^13,14^ However, conventional classical MD and reactive force fields such as ReaxFF face inherent limitations in accurately describing chemical reactions due to their coarse approximations and the need for extensive parameter tuning for each reaction system.^15–17^ Although ab initio MD enables highly accurate simulations of chemical reactions, the associated computational cost remains prohibitively high for large-scale sampling and statistical analysis.

As a potential solution to the trade-off between accuracy and computational cost, universal machine learning interatomic potentials (uMLIPs) have attracted increasing attention.^18–22^ They predict atomic interactions using machine learning models, thus bypassing explicit electronic structure calculations while retaining near first-principles accuracy and substantially reducing computational cost. In particular, the Matlantis PFP removes the necessity for reaction-specific parameter tuning required in empirical MD.^23–25^ PFP-based MD (PFP-MD) can achieve a dramatic acceleration in computational time while maintaining an accuracy comparable to that of ab initio MD. PFP has also been trained on a large-scale DFT data set, enabling robust and transferable simulations across a wide range of systems.

Herein, we propose a computational workflow that enables comprehensive and quantitative evaluation of the oxidation resistance of structurally diverse tertiary phosphines. By using the high accuracy and computational efficiency of PFP-MD, several MD simulations were conducted for each of 51 phosphines. The resulting trajectory was analyzed within a survival-analysis framework to estimate the mean survival time (τ), defined as the reaction-onset lifetime against molecular oxygen.

## 2 Methods

### 2.1 Evaluation of Oxidation Resistance

In this study, the oxidation resistance of phosphines was evaluated by estimating their mean survival time until the occurrence of an oxidation event using survival analysis. This statistical approach enables the appropriate treatment of simulation data even when no oxidation events are observed within the finite duration of MD simulations by handling them as right-censored data. The proposed workflow is shown in Figure 1.

**Figure 1:**
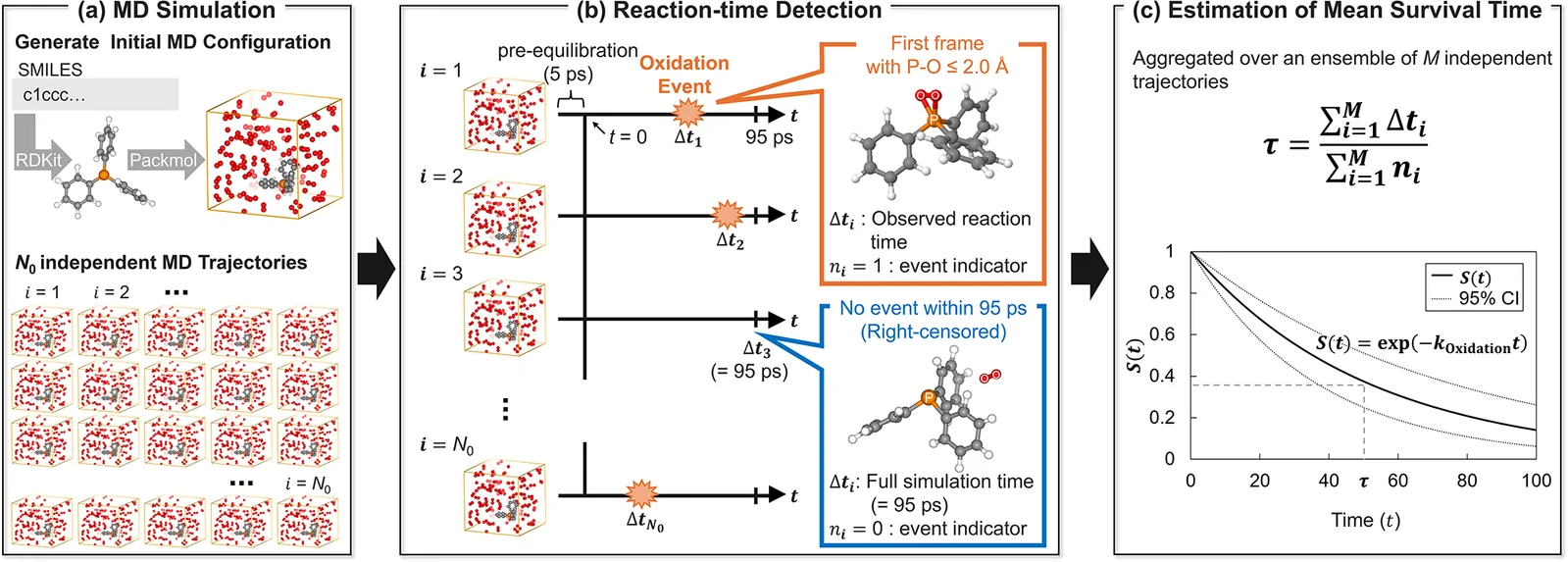
Proposed workflow for τ of phosphines using PFP-MD combined with survival analysis. (a) MD simulation: for each phosphine, *N*_0_ independent trajectories were conducted (RDKit-generated structures packed using Packmol). (b) Reaction-time detection: oxidation events were defined as the first MD frame with P–O ≤ 2.0 Å. After an initial 5 ps pre-equilibration, reaction times were evaluated over a 95 ps analysis window; trajectories without oxidation events were treated as right-censored. (c) Aggregation over an ensemble of all trajectories to evaluate the mean survival time τ.

The MD simulations were first conducted (Figure 1a). The initial molecular structures of all phosphines were generated from SMILES using RDKit.^26^ The initial MD configurations were constructed using the molecular packing program Packmol.^27^ For each phosphine, an ensemble of *N*_0_ independent MD trajectories was conducted by preparing four distinct molecular packings and assigning ten randomized initial velocity sets drawn from a Maxwell–Boltzmann distribution. In all cases, there were sufficient number of oxygen molecules to maintain an effectively constant oxygen concentration regardless of the oxidation reaction. Details are provided in Section 2.2. Each independent MD trajectory was conducted for a total simulation time of 100 ps.

The reaction time between phosphine and molecular oxygen was next detected from the MD trajectories (Figure 1b). An oxidation event was defined as the first formation of a phosphorus–oxygen (P–O) bond with an interatomic distance shorter than 2.0 Å. The validity of this threshold is discussed in detail in Section 3.1.2. To avoid artificial early reactions originating from highly reactive initial configurations and initial velocity assignments, the first 5 ps of each trajectory was regarded as pre-equilibration and was excluded from analysis. For each independent MD trajectory *i*, in which oxidation events did not occur in the pre-equilibration, the survival time Δ*t*_*i*_ and event indicator *n*_*i*_ were recorded as follows, and the total number of such trajectories is denoted as *M*. When an oxidation event was observed within a trajectory, the observation time Δ*t*_*i*_ was defined as the elapsed simulation time from the end of the pre-equilibration to the first occurrence of the oxidation event, and the event indicator was set to *n*_*i*_ = 1. When no oxidation event was detected within the simulation time, the trajectory was treated as a nonreactive (right-censored) case, for which Δ*t*_*i*_ was set to the full simulation time after the pre-equilibration (95 ps) and the event indicator was set to *n*_*i*_ = 0.

Finally, to estimate the mean survival time, the event indicators *n*_*i*_ and observation times Δ*t*_*i*_ obtained were aggregated over an ensemble of *M* independent trajectories (Figure 1c). The mean survival time was estimated using the probabilistic framework proposed by Kröger et al.,^28^ which enables the extraction of reaction rate constants from reactive MD simulations. In the framework, each trajectory is regarded as a realization of a continuous-time stochastic process of the chemical composition characterized by reaction rate constants. The reaction rate constants are then statistically estimated using the fact that the trajectory can be decomposed at reaction events into intervals, each of which is described as a Poisson process with a constant rate, so that the waiting time in each interval follows an exponential distribution. In the present study, the oxidation of phosphine was investigated under conditions where each independent trajectory contains a single phosphine molecule, and the oxygen concentration is sufficiently high and stationary. The oxidation reaction can therefore be approximated as pseudo-first-order with respect to phosphine. In this case, the reaction rate constant estimation in ref 28 reduces to

1 $$k_{\mathrm{Oxidation}}=\frac{\overset{M}{\underset{i=1}{\sum }}n_{i}}{\overset{M}{\underset{i=1}{\sum }}\Delta t_{i}}$$

For an irreversible pseudo-first-order reaction with rate constant *k*_Oxidation_, the waiting time *t* until reaction follows an exponential probability density function

2 $$P\left(t\right)=k_{\mathrm{Oxidation}}\exp\left(-k_{\mathrm{Oxidation}}t\right)$$

The corresponding survival function, defined as the probability that a phosphine molecule remains unreacted up to time *t*, is given by

3 $$S\left(t\right)=\int_{t}^{\infty }P\left(u\right)du=\exp\left(-k_{\mathrm{Oxidation}}t\right)$$

The mean survival time τ of a phosphine molecule is obtained as the expectation value of this distribution,

4 $$\tau =\int_{0}^{\infty }tP\left(t\right)dt=\frac{1}{k_{\mathrm{Oxidation}}}=\frac{\overset{M}{\underset{i=1}{\sum }}\Delta t_{i}}{\overset{M}{\underset{i=1}{\sum }}n_{i}}$$

Thus, τ directly reflects the expected molecular lifetime and therefore serves as a lifetime descriptor for comparing the oxidation resistance of different phosphine molecules. To quantify the associated statistical uncertainty in τ, a confidence interval (CI) was estimated for each compound following Kröger et al.^28^ The expected number of reaction events λ is given by

5 $$\lambda =k_{\mathrm{Oxidation}}\cdot \overset{M}{\underset{i=1}{\sum }}\Delta t_{i}$$

The 95% CI for λ, [λ_low_, λ_up_], was obtained by numerically solving the following system of equations:

6 $$\chi =\Gamma_{\mathrm{inc}}\left(N+1,\lambda_{\mathrm{up}}\right)-\Gamma_{\mathrm{inc}}\left(N+1,\lambda_{\mathrm{low}}\right)$$

7 $$\lambda_{\mathrm{low}}=-N\cdot W_{0}\left(-\frac{\lambda_{\mathrm{up}}}{N}\cdot \exp\left[-\frac{\lambda_{\mathrm{up}}}{N}\right]\right)$$

where *N* is the number of trajectories among the *M* independent trajectories in which an oxidation event was observed, Γ_inc_(*a*, *x*) is the regularized lower incomplete gamma function, *W*_0_ is the principal branch of the Lambert-W function, and χ = 0.95 is the confidence level. Since τ and λ are inversely proportional, the confidence bounds on τ are obtained as

8 $$\tau_{\mathrm{low}}=\frac{\overset{M}{\underset{i=1}{\sum }}\Delta t_{i}}{\lambda_{\mathrm{up}}},,\tau_{\mathrm{up}}=\frac{\overset{M}{\underset{i=1}{\sum }}\Delta t_{i}}{\lambda_{\mathrm{low}}}$$

When no oxidation event was observed in any trajectory, eq 4 yields τ = ∞, and eq 7 is not applicable. In this case, the CI reduces to a one-sided lower bound on τ.^28^ The corresponding upper bound on λ is obtained analytically from eq 6 with λ_low_ = 0 as

9 $$\lambda_{\mathrm{up}}=-\ln\left(1-\chi \right)$$

For χ = 0.95, λ_up_ ≈ 2.996, and the lower bound on τ follows directly from eq 8. The upper bound on τ remains infinity, reflecting the inherently larger statistical uncertainty for highly air-stable phosphines. These statistical uncertainties in τ are reported alongside τ throughout this study.

### 2.2 Computational Details

All MD simulations and geometry optimizations were conducted using PFP on Matlantis.^24^ Calculations were carried out with PFP version 5.0.0, which supports 72 elements across the periodic table. The PBE_PLUS_D3 mode was used, which employs a model trained on data generated using the Perdew–Burke–Ernzerhof (PBE) exchange–correlation functional,^29^ with Grimme’s D3 dispersion correction^30^ applied to the model’s predictions. All MD simulations were conducted in the NVT ensemble, with temperature control implemented using a Nosé–Hoover thermostat with a time constant of 20.0 fs.^31,32^

Before the survival time analysis, MD simulations were performed to analyze the oxidation mechanism of phosphines using triphenylphosphine (PPh_3_) as a typical example, as discussed later in Section 3.1.1. Each MD simulation was conducted at high temperature and high density, which is a common strategy to accelerate reactions within accessible MD time scales.^33^ Each initial MD configuration consisted of five PPh_3_ molecules and 100 oxygen molecules randomly packed with a minimum intermolecular distance of 2.0 Å in a cubic periodic cell at a density of 1.0 g cm^–3^. To examine temperature-dependent behavior, simulations were conducted at 300 and 900 K. The time step was set to 0.1 fs to accurately track bond-formation and bond-breaking processes before and after the reaction events. In this section, a MD trajectory was run to 20 ps. Accordingly, to further elucidate the oxidation mechanism, transition state calculations were conducted to construct the reaction potential energy diagram. Initial and final state geometries were optimized using the limited-memory Broyden–Fletcher–Goldfarb–Shanno (L-BFGS) algorithm with a force convergence criterion of fmax = 0.001 eV Å^–1^.^34^ The transition states were optimized using the Sella package,^35^ with a force convergence criterion of fmax = 0.02 eV Å^–1^. Transition states were confirmed to have a single dominant imaginary frequency corresponding to motion along the reaction coordinate. Additional small imaginary frequencies (<30 cm^–1^), when present, were attributed to soft rotational or conformational modes and were not associated with the chemical transformation.

As explained in Section 3.1.2 and the subsequent sections, to quantitatively evaluate the oxidation resistance of phosphines, MD simulations and survival time analysis were conducted under quantitative-evaluation conditions. For each phosphine, *N*_0_ = 40 independent trajectories were conducted. In these simulations, each initial MD configuration consisted of one phosphine molecule and 80 oxygen molecules randomly packed with a minimum intermolecular distance of 5.0 Å in a cubic periodic cell at a density of 0.5 g cm^–3^. The simulations were conducted at 400 K with a time step of 0.5 fs, and oxidation events were assessed at every MD time step. Figure 2 shows the structural formulas of the 51 different phosphines used in this study.

**Figure 2:**
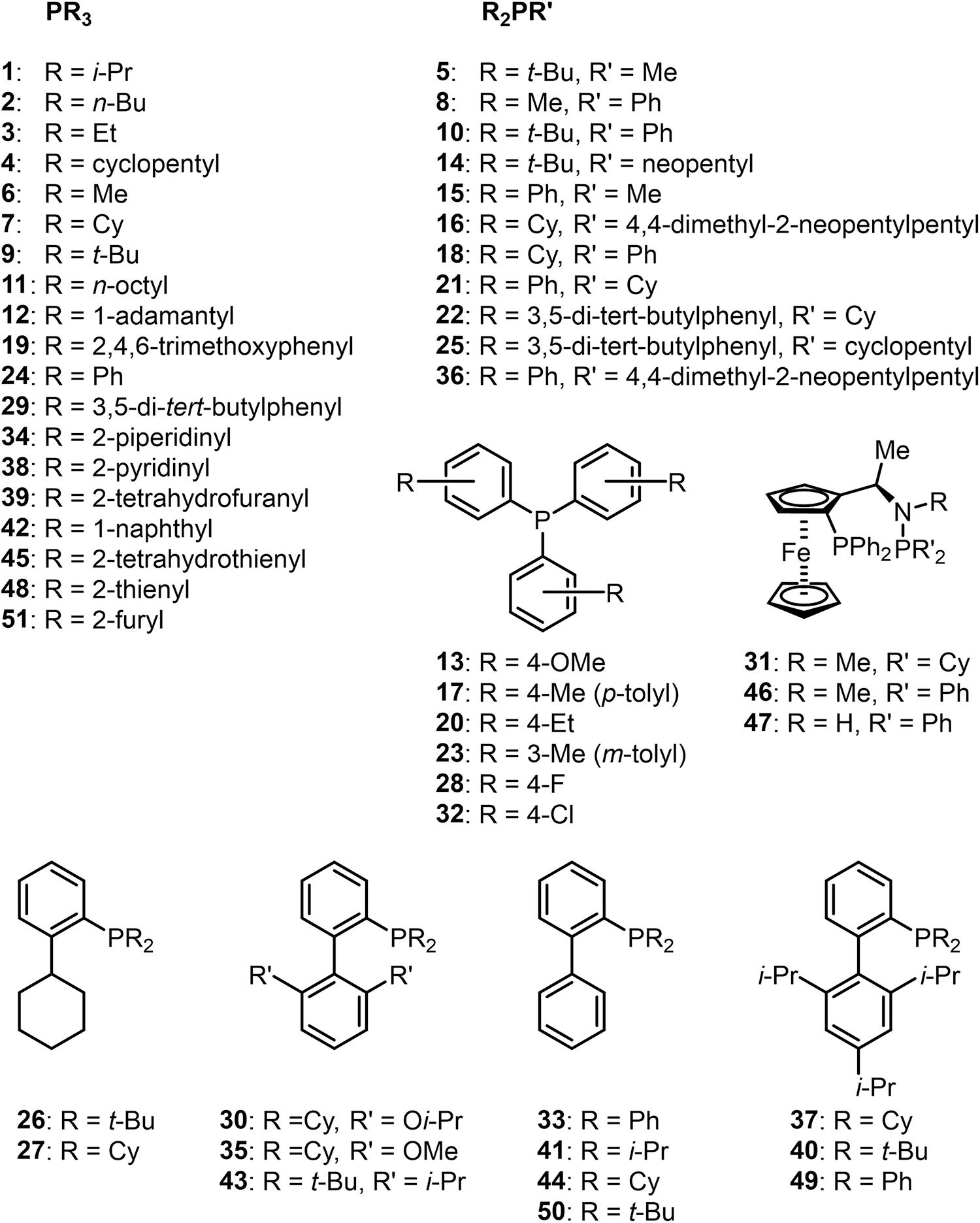
Structural formulas of 51 phosphines examined in this study. Abbreviations are as follows: Me = methyl; Et = ethyl; *n*-Bu = *n*-butyl; *i*-Pr = isopropyl; *t*-Bu = *tert*-butyl; Cy = cyclohexyl; Ph = phenyl.

To statistically characterize the chemical factors governing τ across all 51 phosphines and to assess the chemical validity of τ, as described in Section 3.3, three descriptors independent of MD simulations were evaluated: the percent buried volume (%*V*_bur_) and the Mulliken charge on the phosphorus atom (*Q*_P_), both obtained for the neutral, most stable conformer, and the singly occupied molecular orbital (SOMO) energy of the corresponding phosphine radical cation. The %*V*_bur_ was used because it provides a quantitative measure of steric congestion in the approach direction to the phosphorus donor site (lone-pair axis), which is expected to sterically hinder the approach of molecular oxygen, thus impede oxidation. Operationally, %*V*_bur_ is defined as the fraction (in %) of a reference sphere of radius 3.5 Å centered at the metal atom that is occupied by the ligand’s volume.^36,37^ For each phosphine, a “virtual metal center” was placed along the P–lone-pair (donor) axis at a distance of 2.28 Å from the P atom, and the 3.5 Å sphere was centered at this virtual metal position. Thus

10 $$\%V_{\mathrm{bur}}=\frac{V_{\mathrm{occ}}}{V_{\mathrm{sphere}}}\times 100$$

where *V*_sphere_ is the volume of the 3.5 Å sphere and *V*_occ_ is the occupied volume of the ligand within that sphere. The *Q*_P_ was chosen as a simple, widely used electronic descriptor reflecting the electron density localized at phosphorus center, because the availability and donation propensity of the phosphorus lone pair govern interaction/reactivity with oxygen-derived species. A more negative (electron-richer) *Q*_P_ is generally expected to correlate with higher intrinsic reactivity toward molecular oxygen, whereas a less negative *Q*_P_ suggests diminished electronic driving force for oxidation. SOMO energy was additionally evaluated because it directly reflects the energy level of the electron available for donation upon oxidation, and has been reported to correlate with the air stability of phosphines.^38^ Taken together, %*V*_bur_ captures steric shielding in the donor direction, *Q*_P_ reflects the static electron density localized at phosphorus, and SOMO energy reflects the overall molecular environment beyond the local phosphorus center, together providing complementary perspectives on the chemical factors governing τ. To ensure consistent descriptor evaluation across the full phosphine set, a common conformational-search protocol was employed. For each phosphine, 50 conformers were generated using RDKit and subsequently geometry optimizations were conducted with PFP using the L-BFGS algorithm with a force convergence threshold of fmax = 0.001 eV Å^–1^. The most stable conformer was then identified. The %*V*_bur_ and *Q*_P_ were calculated for the most stable conformer. For SOMO energy calculations, the most stable conformer was converted to the corresponding radical cation and subsequently reoptimized, and the SOMO energy was evaluated for the optimized radical-cation structure. The %*V*_bur_ was calculated using the MORFEUS package.^39^ *Q*_P_ and SOMO energy were calculated using the Gaussian 16 program with the B3LYP-D3­(BJ) functional.^30,40,41^ The basis set was 6–311+G­(d) for iron atoms and 6–31G­(d,p) for all other atoms. The strength of the linear relationship between each descriptor and log_10_τ, as discussed in Section 3.3.1, was quantified using the Pearson correlation coefficient (*r*).

To evaluate the combined predictive performance of *Q*_P_, %*V*_bur_, and SOMO energy for τ, as described in Section 3.3.2, several regression algorithms, including linear regression, ridge regression,^42^ lasso regression,^43^ elastic net regression,^44^ support vector regression with an RBF kernel,^45^ random forest (RF) regression,^46^ and gradient boosting regression,^47^ were compared using these three standardized descriptors as inputs. Three phosphines for which no oxidation event was observed within the simulation time window (Compounds **49**–**51**, τ = ∞) were excluded from this regression analysis, yielding a data set of 48 phosphines. To prevent information leakage, hyperparameters were optimized within each fold of leave-one-out cross-validation (LOO–CV) by an inner 5-fold CV on the 47-compound training set before predicting the held-out compound (nested LOO–CV). Predictive performance was evaluated using *Q* ^2^, the cross-validated coefficient of determination, which reflects performance on unseen compounds rather than the goodness of fit to the training data.

## 3 Results and Discussion

### 3.1 Validation of the Proposed Method: The Case of Triphenylphosphine Oxidation

It is proposed that the oxidation mechanism of phosphines in air is sensitive to substituent structure and involves multiple pathways.^48–50^ For example, in the case of sterically hindered phosphines, oxidation was proposed to proceed without the formation of a radical-cation intermediate and instead via a phosphadioxirane-type species as a key intermediate. As mentioned in Section 2.2, as a preliminary step, MD simulations were conducted for PPh_3_, a typical tertiary phosphine widely used as a ligand,^51^ in the presence of molecular oxygen under mechanistic-analysis conditions. The trajectories were used to identify the reaction pathways of phosphine oxidation and monitor dynamic events.

#### 3.1.1 Identification of Oxidation Reaction Pathways

The reaction pathways of PPh_3_ oxidation were investigated by MD simulations at 300 and 900 K. The initial configuration contains 5 PPh_3_ molecules and 100 oxygen molecules (summarized in Figure 3). At both simulation temperatures, no decomposition of the PPh_3_ molecular framework was observed throughout the simulations, and PPh_3_ rapidly formed phosphadioxirane (I), in which two oxygen atoms are bound to the phosphorus center (Figure 3a, Step 1). Subsequently, phosphadioxirane (I) reacted with an unreacted PPh_3_ molecule, yielding two molecules of phosphine oxide (II) (Figure 3b, Step 2). Only at 900 K, in addition to Steps 1 and 2, an intramolecular reaction pathway was observed: the phosphadioxirane (I) further rearranged to form a phosphinate species (III) (Figure 3c, Step 3). These reaction pathways are consistent with a previously reported mechanism for phosphine oxidation,^52^ indicating that the present simulations accurately reproduce the known reactivity landscape of PPh_3_.

**Figure 3:**
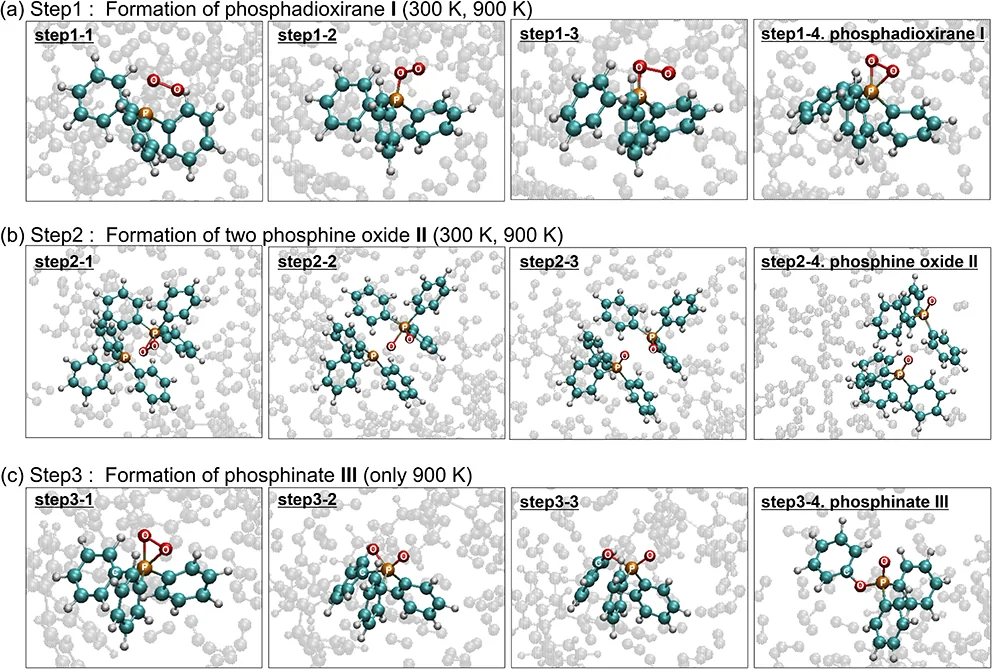
Reaction pathways for PPh_3_ oxidation obtained from MD simulations at 300 and 900 K. (a) Step 1: Rapid formation of phosphadioxirane (I), in which two oxygen atoms are bound to phosphorus. (b) Step 2: Subsequent reaction of phosphadioxirane (I) with unreacted PPh_3_ molecule to produce two molecules of phosphine oxide (II). (c) Step 3 (900 K only): Intramolecular rearrangement of phosphadioxirane (I) to yield phosphinate species (III). Hydrogen atoms are shown in white and carbon atoms in cyan.

To further analyze the pathways, potential energy diagrams for Steps 1–3 were calculated through geometry optimization and transition state search, and the results are shown in Figure 4. The activation energy for the formation of phosphadioxirane (I) in Step 1 was relatively small (Ea_1_ = 1.9 kcal/mol). The activation energy increased in Step 2, where the formation of phosphine oxide (II) required Ea_2_ = 11.8 kcal/mol, and it became even larger in Step 3, with the formation of phosphinate (III) requiring Ea_3_ = 13.7 kcal/mol. This progressively higher activation barrier is consistent with the observation that Step 3 occurred only in the high-temperature (900 K) MD simulations.

**Figure 4:**
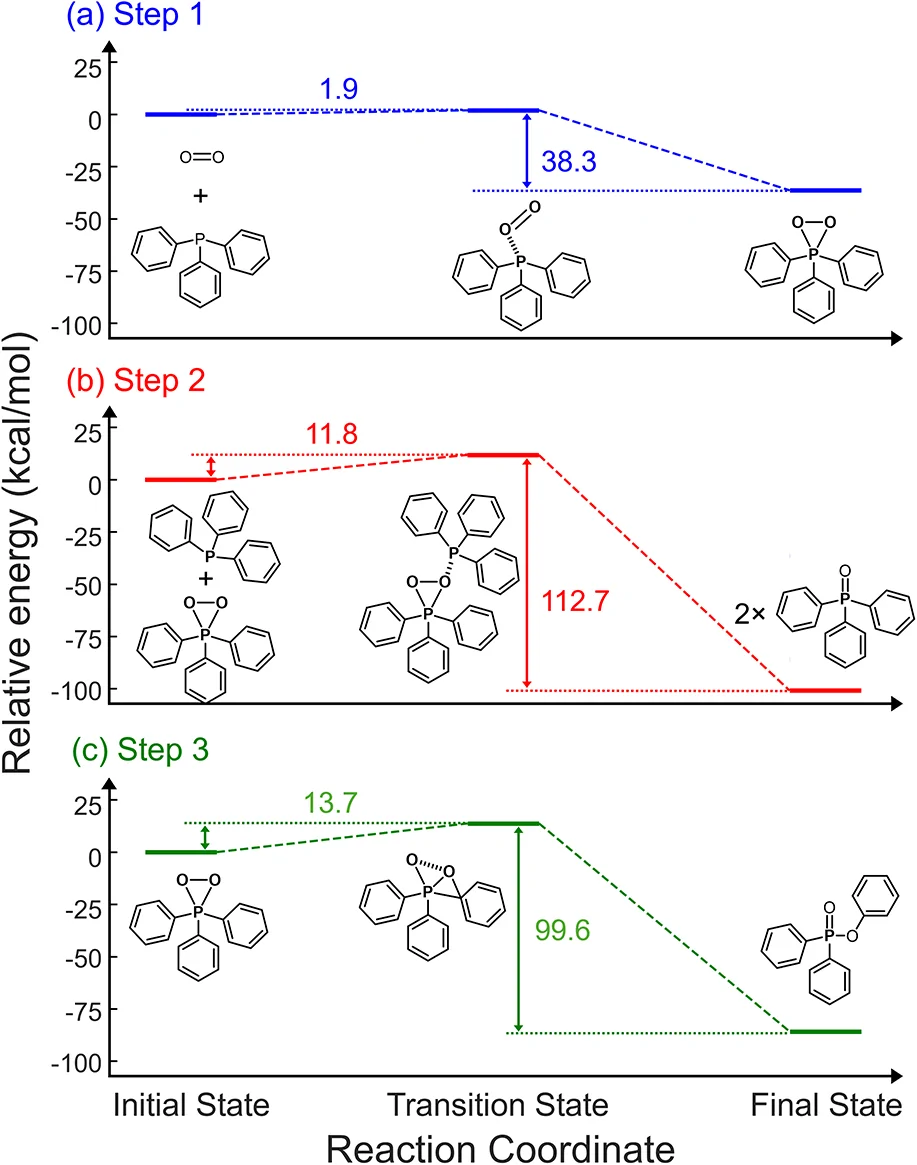
Potential energy diagrams for the PPh_3_ oxidation pathways, corresponding to Steps 1–3 defined in Figure 3. Only the reacting species were included, and other oxygen molecules present in the MD simulations were not considered. The highest activation energy for Step 3 explains why this step was observed only in high-temperature (900 K) MD trajectories.

To further examine the validity of this barrier ordering, we compared it with independent kinetic evidence. Previous kinetic studies on the ortho isomer of tris­(methoxyphenyl)­phosphine have reported that, under photosensitized conditions, the oxide-forming pathway corresponding to Step 2 is kinetically favored over the competing phosphinate-forming pathway (Step 3), with an experimentally measured rate constant approximately 25 times larger.^52^ Because the cited study and the present calculations differ in several important respects, including reaction conditions, phase, and the factors governing reactive encounters, direct quantitative comparison between the two systems is difficult. The qualitative kinetic preference is nevertheless consistent between the two studies, as the present calculations predict a lower activation barrier for Step 2 than for Step 3, indicating that the oxide-forming pathway is kinetically favored over the competing phosphinate-forming pathway. Experimentally observed reactivity can also be influenced by reactive encounter probabilities, entropic contributions, and environmental effects that are not captured by static activation energies alone. In particular, Step 2 is bimolecular whereas Step 3 is unimolecular, so differences in activation entropy and pre-exponential factors are expected even when comparing pathways originating from the same intermediate.

The calculated energy profiles also provide insight into the reversibility of the oxidation process. The activation energies for the reverse reactions of Steps 1–3 were estimated to be 38.3, 112.7, and 99.6 kcal/mol, respectively, indicating that phosphine oxidation proceeds effectively irreversibly under the present conditions.

Beyond these observations, we also assessed the extent to which activation barriers can distinguish phosphines with substantially different air stability. The forward activation barrier of Step 1 is only 1.9 kcal/mol even for PPh_3_, which exhibits moderate oxidation resistance. Accordingly, we additionally calculated the Step 1 activation energy for triethylphosphine (PEt_3_), which is known to be significantly less air-stable than PPh_3_.^53^ Despite their markedly different oxidation resistance, the calculated activation energies were found to be very similar (see Supporting Information), suggesting that static activation barriers alone may provide insufficient discriminatory power for reliably ranking phosphines with substantially different oxidation resistance.

Furthermore, transition-state calculations with a fixed geometry for the initial state do not account for the probability that reactive species reach a productive reactive conformation. MD naturally captures this through conformational sampling with Boltzmann weighting, which can be as important as the intrinsic barrier in governing the observed reaction rate and branching behavior. These results together demonstrate that PFP-MD can reliably describe both the kinetic preference and the effectively irreversible nature of phosphine oxidation while explicitly accounting for conformational accessibility that is not captured by transition-state calculations alone.

#### 3.1.2 Dynamically Track Time of Oxidation Events and Estimation of Mean Survival Time

As described in Section 2.2, MD simulations described in this section onward were conducted under quantitative-evaluation conditions to efficiently evaluate oxidation resistance for 51 different phosphines. In this section, the oxidation behavior of PPh_3_ is first examined as a representative example to evaluate oxidation resistance.

Dynamic tracking of MD trajectories revealed the approach of oxygen molecules toward PPh_3_. As shown in Figure 5a, an oxygen molecule that was initially distant approached the phosphorus atom and formed phosphadioxirane (I) at around 80 ps. For the reacting oxygen molecule (O_1_–O_2_), the temporal progression of the P–O_1_ and P–O_2_ distances throughout the trajectory is shown in Figure 5b. After the oxidation event at around 80 ps, both P–O distances remained at approximately 1.7 Å, indicating that once the oxygen molecule became bound to the phosphorus atom, the oxidized configuration was stably maintained. Figure 5c presents the time-dependent nearest P–O distance among all oxygen molecules in the system. Prior to the oxidation event, the nearest P–O distance never fell below 2 Å. In summary, an oxygen molecule could not approach within 2 Å before the oxidation event; once it came within 2 Å, a P–O bond was formed, and no dissociation is observed thereafter. This behavior was also consistently observed for the five different phosphines (Compounds **7**, **12**, **22**, **24,** and **36**), for which 40 MD trajectories were conducted for each phosphine under the quantitative-evaluation conditions (see Supporting Information). The observation that no P–O bond dissociation occurred was also consistent with the high reverse activation energy of 38.3 kcal/mol obtained for Step 1 from the energy diagram in Figure 4. On the basis of these observations, the time of an oxidation event was operationally defined as the moment when the nearest P–O distance first reached 2.0 Å. For subsequent simulations with other phosphines, the MD calculation was terminated 1 ps after the nearest P–O distance dropped below 2 Å to reduce computational cost.

**Figure 5:**
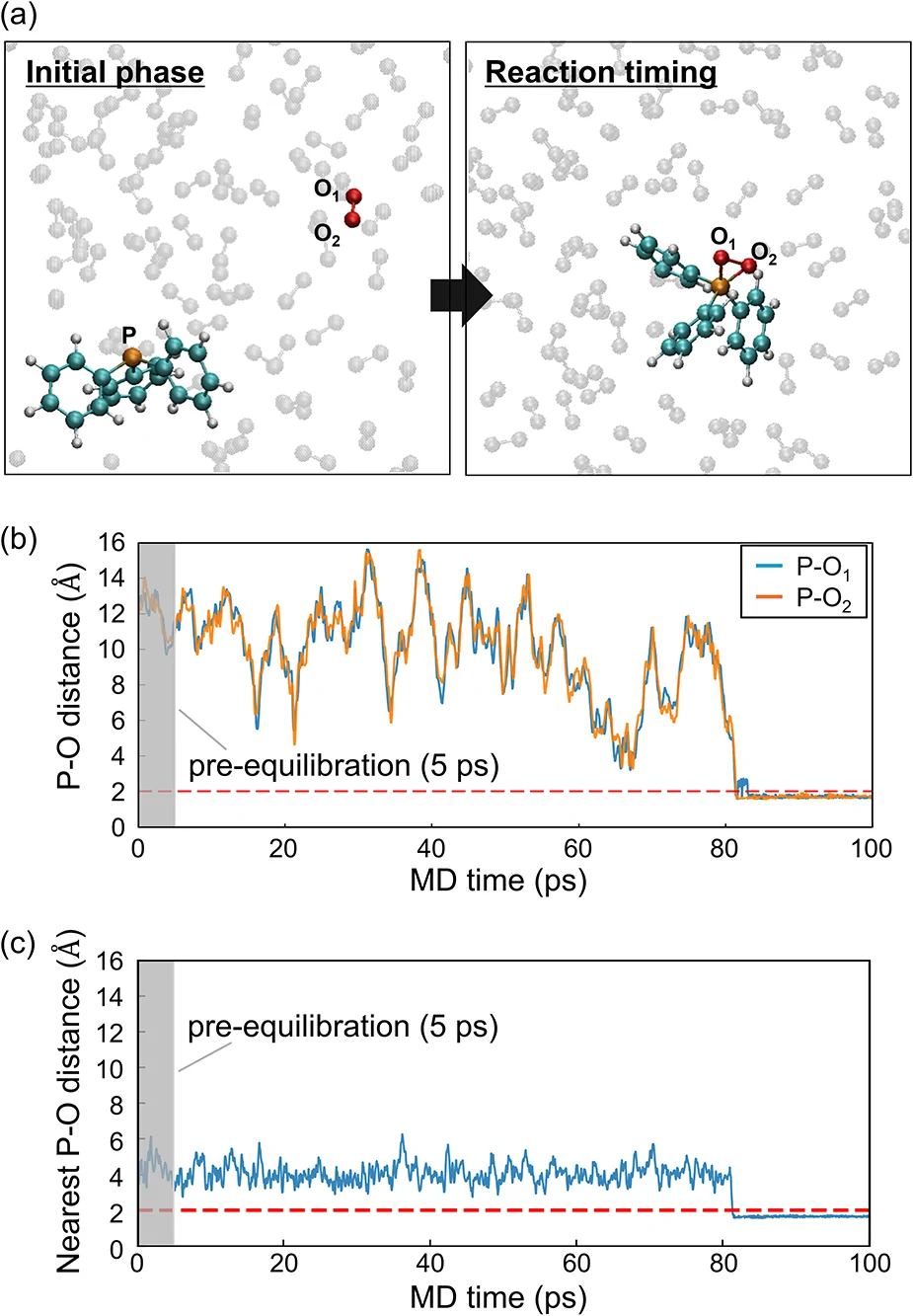
Representative MD results for PPh_3_ illustrating oxygen molecule approach and P–O formation under quantitative-evaluation conditions. (a) Time evolution of oxygen molecule approaching PPh_3_, leading to formation of phosphadioxirane (I) at around 80 ps. (b) Temporal profiles of P–O_1_ and P–O_2_ distances for reacting oxygen molecule, showing that both P–O bonds stabilized at ∼ 1.7 Å after oxidation event. (c) Time-dependent nearest P–O distance among all oxygen molecules, indicating that no P–O distance fell below 2.0 Å prior to oxidation, whereas entry within 2.0 Å resulted in irreversible bond formation.

Finally, the τ for PPh_3_ was evaluated. From all 40 MD trajectories, the total observation time aggregated over all trajectories before oxidation events or right-censoring was 1630.8 ps, and a total of 32 oxidation events were observed. Accordingly, τ was calculated using eq 4 as τ = 1630.8 ps/32, giving τ = 51.0 ps.

### 3.2 Assessment of Consistency with Experimental and Theoretical Observations

The practical usefulness of τ as a lifetime descriptor depends on its ability to reproduce oxidation-resistance trends that have been established experimentally and theoretically. In this section, τ is compared with experimentally reported oxidation resistance and with literature-based theoretical predictions across a diverse set of phosphines to assess its chemical validity.

#### 3.2.1 Comparison with Experimental Observations for Air-Sensitive Phosphines

The percentage of unoxidized phosphine remaining after 0.5-h exposure to air under neat conditions was reported for five alkyl phosphines, including tributylphosphine (Compound **2**), triethylphosphine (Compound **3**), di-*tert*-butylmethylphosphine (Compound **5**), tricyclohexylphosphine (Compound **7**), and trioctylphosphine (Compound **11**), as well as for PPh_3_ (Compound **24**), based on ^31^P NMR spectra before and after exposure.^53^ Accordingly, τ was calculated for these phosphines, and its trend was compared with the residual percentage. The results are shown in Figure 6. For the phosphines reported to exhibit residual percentage ≤ 20% (Compounds **2**, **3**, and **5**), τ were 6.6, 6.7, and 7.8 ps, respectively, all below 8 ps. In contrast, PPh_3_ (Compound **24**), for which a 100% residual percentage (no detectable oxidation) was reported, exhibited the largest τ (51.0 ps) among the phosphines examined. Although the 95% CIs of Compounds **2**, **3**, **5**, and **7** overlap substantially with one another, τ still increases with increasing residual percentage, and PPh_3_ remains clearly distinguished from the other alkylphosphines examined by its substantially higher τ. Overall, the reported trend in residual percentage was well reproduced by τ.

**Figure 6:**
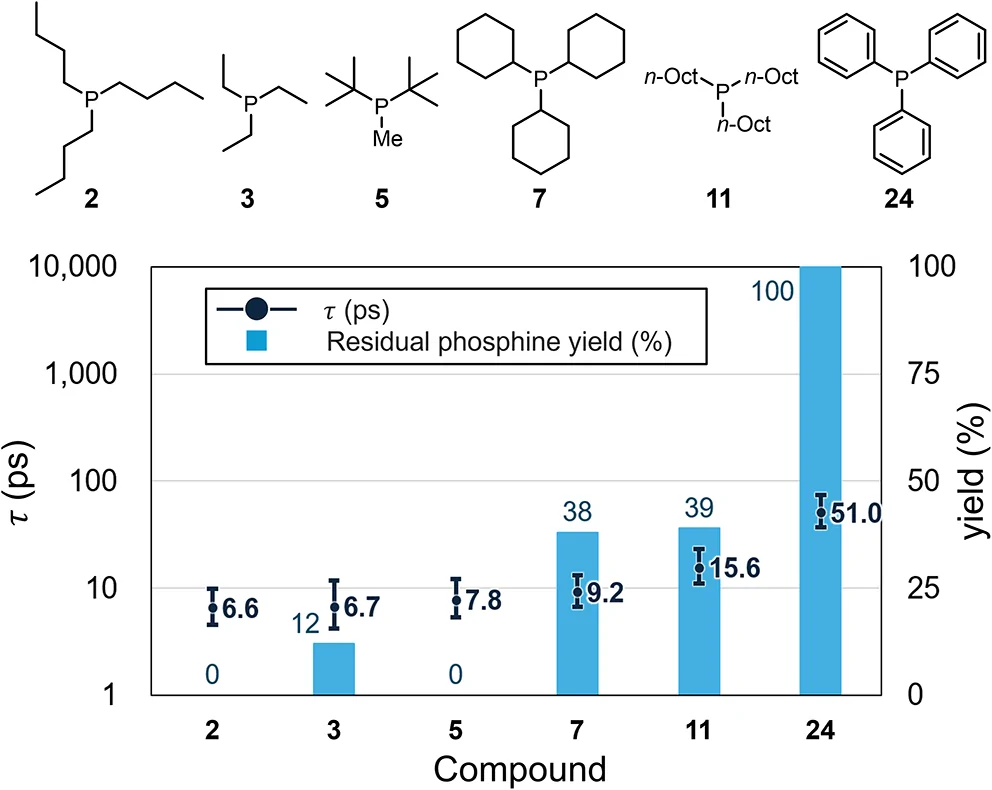
Comparison between τ and experimentally reported residual percentages of unoxidized phosphine after 0.5-h exposure to air under neat conditions (^31^P NMR spectra reported by Hoefler et al.^53^). Blue bars indicate the reported residual phosphine yield, and black circles with error bars represent τ and its 95% confidence intervals (CIs). Phosphines reported with residual percentage ≤ 20% (Compounds **2**, **3**, **5**) correspond to τ < 8 ps, whereas Compound **24** (100% residual) shows the largest τ (51.0 ps).

#### 3.2.2 Comparison with Observations for Air-Stable Tertiary Phosphines at Higher Temperature

The oxidation resistance of tertiary phosphines was experimentally evaluated by Barder et al., exposing each phosphine to three conditions: (a) oxygen atmosphere at 100 °C, (b) air at 100 °C, and (c) oxygen atmosphere at room temperature.^9^ After 65 h, the resulting phosphine oxide was quantified by ^31^P NMR spectroscopy. We compared the experimentally reported phosphine oxide percentages under conditions (a) and (b) with our τ (Figure 7), while excluding condition (c) because most phosphines were reported to remain essentially unoxidized and no meaningful trend was observed. The extreme condition (a) is generally suitable for comparing highly oxidation-resistant phosphines, whereas the milder condition (b) enables discussion among less stable ones. As shown in Figure 7, the phosphines we examined can be classified into three groups: (i) phosphines lacking either a biaryl backbone or a 2-cyclohexylphenyl substituent (Compounds **7**, **18**, **24**), (ii) phosphines bearing a 2-cyclohexylphenyl substituent (Compounds **26**, **27**), and (iii) biaryl phosphines (Compounds **30**, **33**, **35**, **37**, **40**, **41**, **43**, **44**, **49**, **50**).

**Figure 7:**
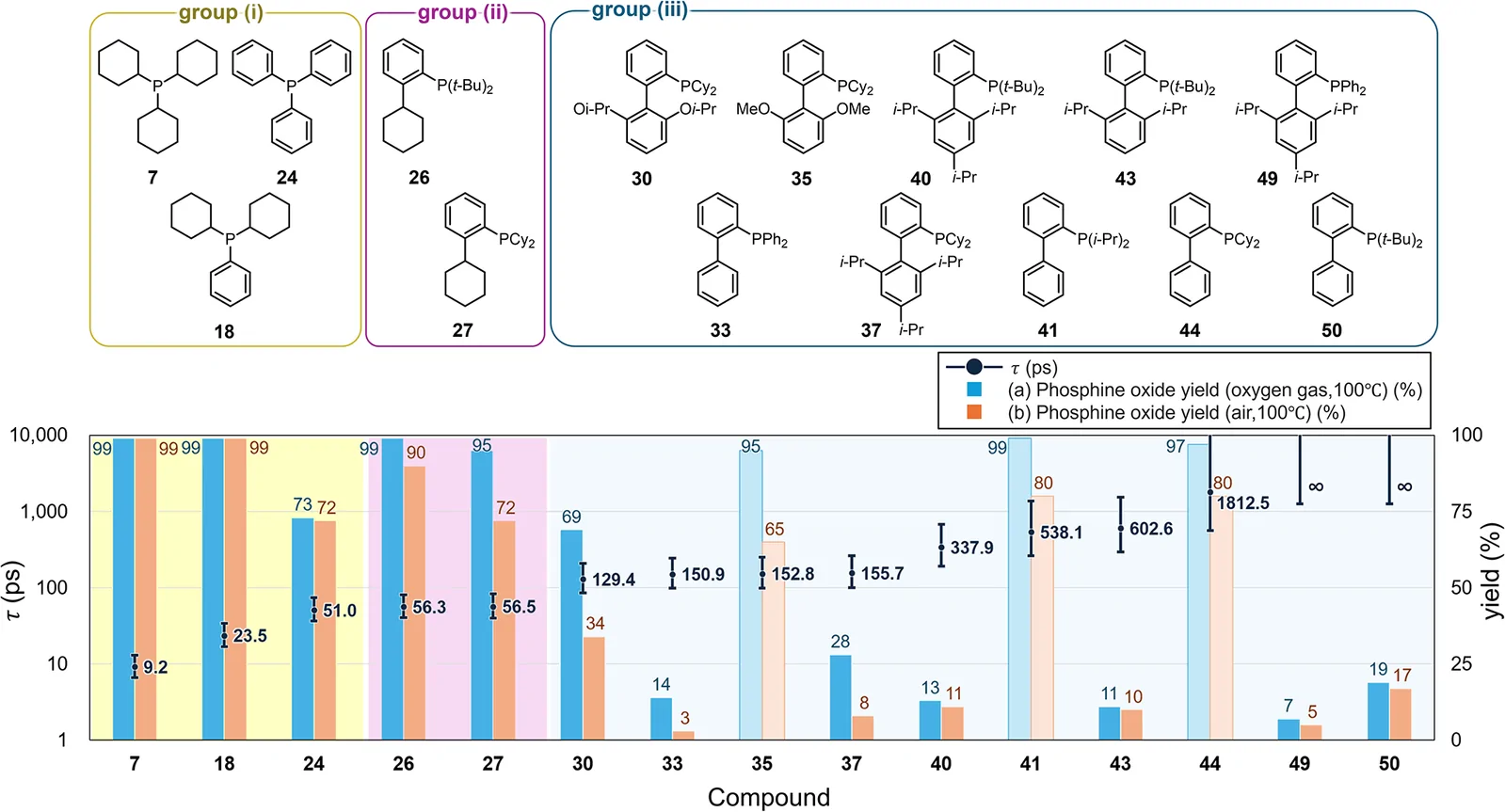
Comparison of τ with experimentally reported phosphine-oxide percentages (^31^P NMR spectra) by Barder et al.^9^ under two conditions, each for 65 h: (a) oxygen gas at 100 °C (blue bars) and (b) air at 100 °C (orange bars). Black circles with error bars represent τ and its 95% CIs. Phosphines were grouped as (i) phosphines lacking either biaryl backbone or 2-cyclohexylphenyl substituent (Compounds **7**, **18**, **24**), (ii) phosphines bearing 2-cyclohexylphenyl substituent (Compounds **26**, **27**), and (iii) biaryl phosphines (Compounds **30**, **33**, **35**, **37**, **40**, **41**, **43**, **44**, **49**, **50**). Compounds **35**, **41**, and **44** (lighter-shaded bars) are discussed separately in Figure 8 because their τ values show notable deviations from the experimental trend.

For group (i), tricyclohexylphosphine (Compound **7**), dicyclohexylphenylphosphine (Compound **18**), and PPh_3_ (Compound **24**), τ increased with the number of phenyl substituents (9.2, 23.5, and 51.0 ps), indicating enhanced oxidation resistance. Experimentally, both conditions (a) and (b) showed the same order (Compounds **7**, **18** < Compound **24**), and under condition (b) the oxide percentages were 99, 99, and 72%, respectively, confirming that PPh_3_ (Compound **24**) is the greatest oxidation resistant among them. These experimental results are fully consistent with the τ trends, which are further supported by the relatively narrow 95% CIs for Compounds **7**, **18**, and **24**, confirming that the rank order among these three compounds is statistically well-defined.

For group (ii), di-*tert*-butyl-(2-cyclohexylphenyl)­phosphine (Compound **26**) and dicyclohexyl-(2-cyclohexylphenyl) phosphine (Compound **27**) exhibited τ of 56.3 and 56.5 ps, respectively. These values are comparable to that of PPh_3_ (Compound **24**, τ = 51.0 ps), which belongs to group (i) and shows the largest τ within that group. Experimentally, under condition (a), Compounds **24**, **26**, and **27** were almost completely oxidized (73, 99, and 95%), making comparison with the less oxidation-resistant phosphines in group (i) difficult. Under condition (b), however, the oxide percentages were 72, 90, and 72%, all corresponding to approximately 70*–*90% oxidation. This indicates that group (ii) exhibits greater oxidation resistance than Compound **7** and **18**, which belong to group (i). Thus, the τ results correctly reproduced both the comparable oxidation resistance among these three phosphines and the trend that group (ii) tends to exhibit oxidation resistance comparable to, or greater than that observed for group (i). This comparability is further reflected in the overlapping 95% CIs of Compounds **26** and **27** with that of PPh_3_ (Compound **24**), statistically confirming that these three phosphines exhibit comparable oxidation resistance.

For group (iii), excluding Compounds **35**, **41**, and **44**, all biaryl phosphines exhibited larger τ than PPh_3_ under both conditions (a) and (b). Experimentally, their oxide percentages under condition (b) were lower than that of PPh_3_, indicating higher oxidation resistance. This experimental trend is consistent with the larger τ for these phosphines. Since oxidation proceeds only to a limited extent under condition (b), we used condition (a) for a more detailed comparison and found a clear trend. For Compounds **30**, **33**, **37**, **40**, **43**, **49**, and **50**, the oxide percentages were respectively 69, 14, 28, 13, 11, 7, and 19%, with corresponding τ of 129.4, 150.9, 155.7, 337.9, 602.6, ∞, and ∞ ps, respectively. For Compounds **49** and **50**, which yielded no oxidation events in any trajectory, the 95% CI reduces to a one-sided lower bound of τ > 1268 ps, still substantially exceeding τ of PPh_3_ (Compound 24, 51.0 ps). Accordingly, the oxide percentages decreased with increasing τ, aside from a minor increase at Compound **50**, demonstrating a negative correlation. These results indicate that τ successfully reproduced both the magnitude and rank order of oxidation resistance within the biaryl phosphine series.

Although Compounds **35**, **41**, and **44** exhibited poor oxidation resistance experimentally, their τ were larger than that of PPh_3_ (Compound **24**), indicating a divergence between the experimental trend and τ. This observed mismatch can be explained by the ligands’ intrinsic preference for the oxidation-susceptible A-away conformation: the relatively small dialkyl groups within the diaryl framework promote adoption of A-away, and the small substituents at the 2′ and 6′ positions on the distal phenyl ring likewise bias the ligands toward this conformation (Scheme 1).^9^ The transition from the A structure to A-away structure was reported to require activation energies of 12.1, 13.6, and 14.1 kcal/mol, respectively. Furthermore, the free energy of the A-away conformation relative to the A conformation (Δ*G*) was reported to be +2.7, + 3.0, and +5.4 kcal/mol for Compounds **35**, **41**, and **44**, respectively.^9^ In contrast, for the *tert*-butyl-substituted biaryl phosphines (Compounds **40**, **43**, and **50**), the corresponding free-energy differences are 12.6, 13.1, and 8.7 kcal/mol, respectively, while the activation barriers for the A-to-A-away transition rise to 24.6, 24.8, and 26.3 kcal/mol.^9^ These results indicate that both the thermodynamic penalty for adopting the oxidation-susceptible A-away conformation and the kinetic barrier for accessing it are substantially lower for Compounds **35**, **41**, and **44**. As a result, interconversion to the A-away conformation is expected to be significantly more feasible than for the *tert*-butyl-substituted biaryl phosphines. Nevertheless, the reported activation barriers of 12.1–14.1 kcal/mol remain sufficiently large that such transitions are still expected to be rare on the MD time scale employed in the present study. Therefore, conformational accessibility may contribute to the discrepancy between the experimentally observed oxidation resistance and the τ obtained from simulations initiated from the A conformation.

**Scheme 1:**
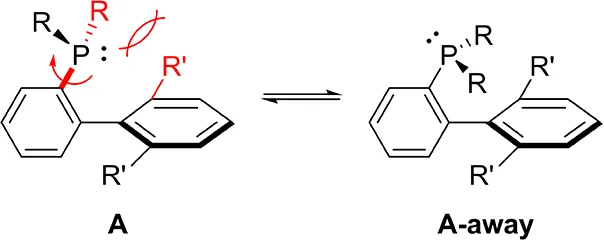
Interconversion of Biaryl-phosphine from A Structure to A-away Structure

To directly assess the impact of this conformational preference on oxidation resistance, we performed additional MD simulations for Compounds **35**, **41**, and **44** using A-away conformations as the initial structures (denoted Compounds **35′**, **41′**, and **44′**, respectively). As shown in Figure 8, the resulting τ were 42.4, 38.9, and 34.4 ps for Compounds **35′**, **41′**, and **44′**, respectively. These values are all substantially smaller than τ of PPh_3_ (Compound 24, 51.0 ps), consistent with the experimentally observed high susceptibility to oxidation of these ligands. These results confirm that when simulations are initialized from the A-away conformer, τ successfully captures the experimentally observed poor oxidation resistance of Compounds **35**, **41**, and **44**, while standard simulations initialized from the A conformation cannot sample the interconversion from the A to the A-away conformer within the MD time scale and therefore overestimate the oxidation resistance of these ligands. Modestly increasing the simulation temperature or using enhanced-sampling techniques may render these transitions observable, enabling a more faithful evaluation.

**Figure 8:**
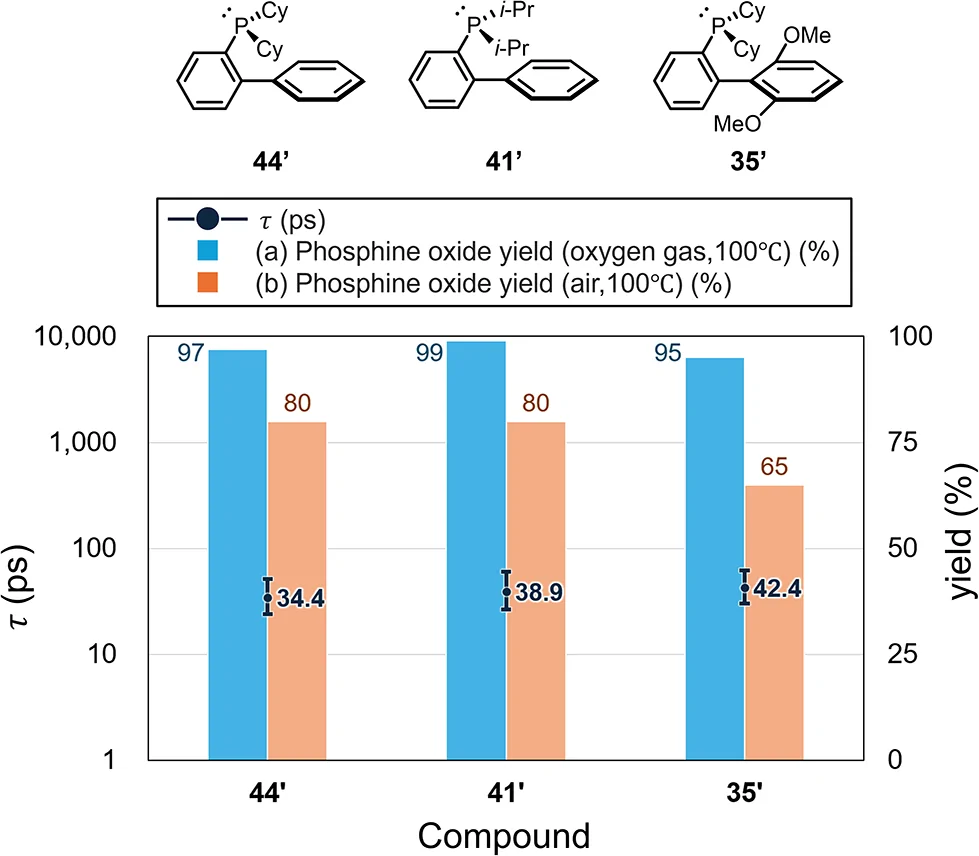
Comparison of τ for Compounds **35′**, **41′**, and **44′**, in which the initial MD structure was set to the A-away conformation of Compounds **35**, **41**, and **44**, with phosphine-oxide yields experimentally reported by Barder et al.^9^ under two conditions, oxygen gas at 100 °C (blue bars) and air at 100 °C (orange bars). Black circles with error bars represent τ and its 95% CIs.

Overall, τ successfully reproduces the experimentally observed oxidation resistance trends and rank order across a wide range of phosphine structures, including differences among groups (i), (ii), and (iii), except for the rare cases where reactions with high activation energies, not captured within practical MD sampling, impact the oxidation resistance.

#### 3.2.3 Comparison with DFT-Based Predictions

It has been reported that the SOMO energy of the phosphine radical cation is an important descriptor governing their air stability.^38^ To assess the reproduction of this reported relationship by τ, τ was compared with the reported SOMO energies for five tertiary phosphines, including triethylphosphine (Compound **3**), trimethylphosphine (Compound **6**), dimethyl­(phenyl)­phosphine (Compound **8**), methyldiphenylphosphine (Compound **15**), and PPh_3_ (Compound **24**), as summarized in Figure 9.^38^ The results indicate that phosphines with higher SOMO energies tend to exhibit larger τ. The nonaromatic and highly pyrophoric alkylphosphines triethylphosphine (Compound **3**) and trimethylphosphine (Compound **6**) exhibited smaller τ. In contrast, PPh_3_ (Compound **24**), which is known to be stable in air, exhibited the largest τ among the five. These results are consistent with the previous study,^38^ which indicated that phosphines with SOMO energies above −10 eV, such as PPh_3_, and aromatic phosphines exhibit high oxidation resistance. Although the 95% CIs of adjacent compounds partially overlap, the overall trend of increasing τ with SOMO energy is consistently maintained, and PPh_3_ (Compound **24**) is clearly distinguished from the other four phosphines by its substantially higher τ.

**Figure 9:**
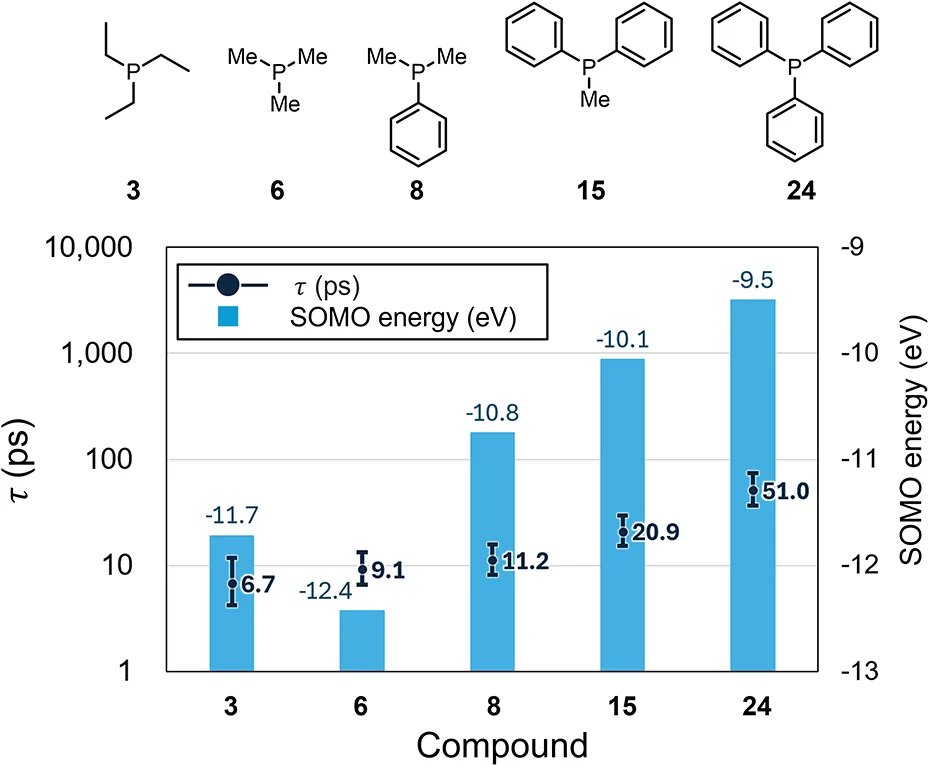
Comparison of τ with SOMO energies of phosphine radical cations (DFT) reported by Stewart et al.^38^ for five tertiary phosphines. Black circles with error bars represent τ and its 95% CIs. Blue bars represent the reported SOMO energy. Phosphines with higher SOMO energies exhibited larger τ, with aromatic PPh_3_ (Compound **24**) showing largest τ and alkylphosphines (Compounds **3** and **6**) showing smallest.

#### 3.2.4 Experimental Consistency in Oxidation-Resistant Phosphines Bearing Specialized Frameworks

In addition to the comparisons between τ and previously reported trends presented in Sections 3.2.1–3.2.3, the applicability of τ to experimentally reported oxidation-resistant phosphines with typical structural frameworks were evaluated. The ability of τ to identify, among such specialized frameworks, those exhibiting particularly pronounced oxidation resistance was examined.

The bidentate ligands featuring an alkylated ferrocene (BoPhoz) framework, Compounds **31**, **46**, and **47**, have been reported to afford excellent oxidation resistance.^10^ To evaluate the experimentally reported oxidation resistance of these ligands using τ, τ were calculated for these ligands, yielding 141.9, 1850.3, and 1867.9 ps for Compounds **31**, **46**, and **47**, respectively (Figure 10a). These values all exceed that of PPh_3_ (Compound **24**, τ = 51.0 ps), which exhibits moderate oxidation resistance, indicating that the BoPhoz ligands exhibit greater oxidation resistance than PPh_3_. Particularly for Compounds **46** and **47**, the lower bounds of their 95% confidence intervals exceed the upper bound of the 95% confidence interval of PPh_3_, indicating statistically greater oxidation resistance than PPh_3_ within the present simulations. Furthermore, even Compound **31**, which bears a Cy substituent, exceeded air-stable PPh_3_, consistent with the previous reports that the BoPhoz framework enhances oxidation resistance.^10^ Hence, τ successfully reproduces the experimentally reported oxidation-resistance trend even for BoPhoz ligands with distinctive architectures.

**Figure 10:**
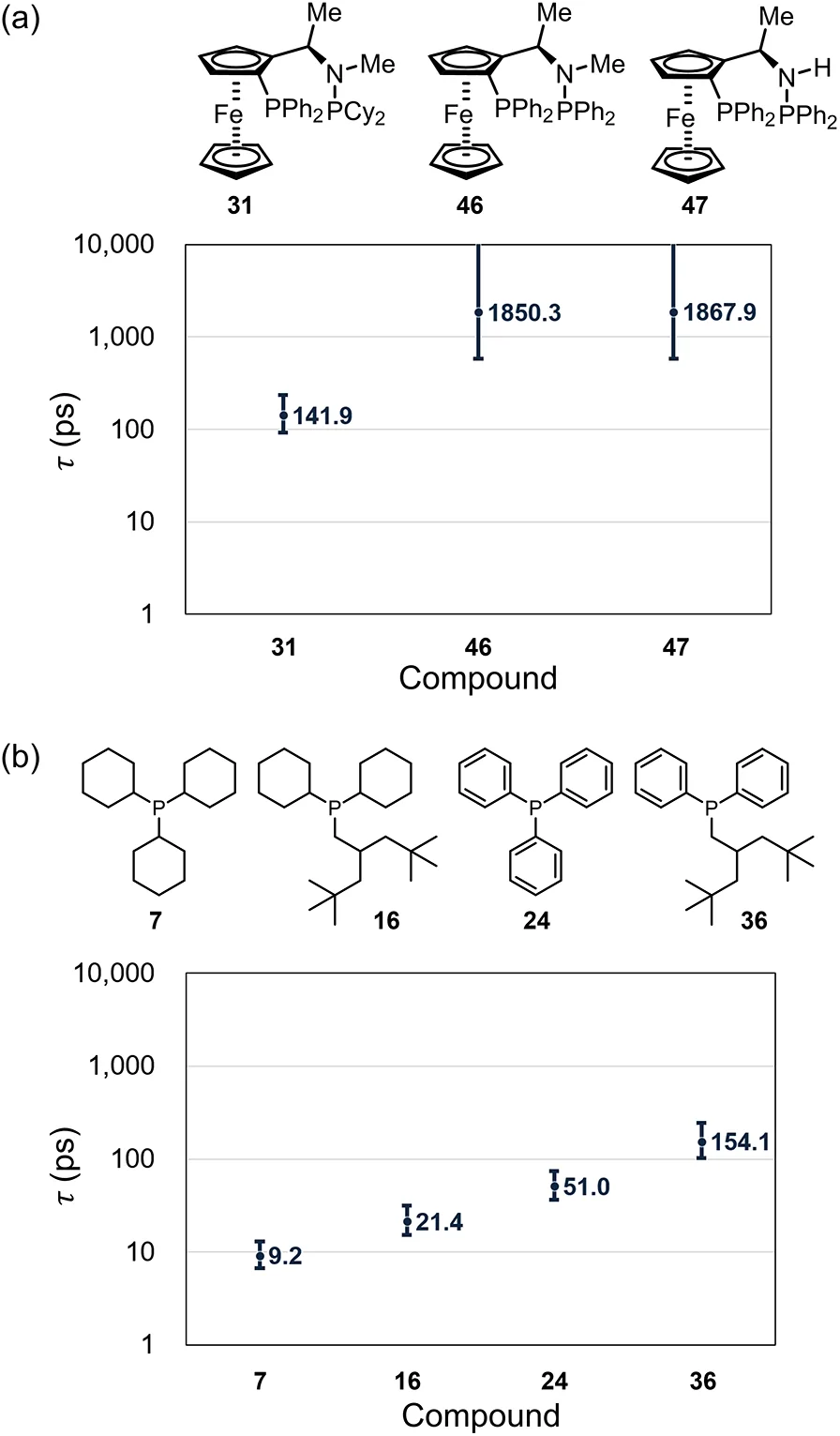
τ for phosphines bearing experimentally reported oxidation-resistant frameworks. Black circles with error bars represent τ and its 95% CIs. (a) Bidentate ligands with alkylated ferrocene (BoPhoz) framework (Compounds **31**, **46**, **47**), reported by Boaz et al.^10^ (b) Phosphines incorporating TIB frameworks (Compounds **16**, **36**), compared with tricyclohexylphosphine (Compound **7**) and PPh_3_ (Compound **24**), reported by Matsubara et al.^11^

As another illustrative case, ligands incorporating the bulky yet flexible TIB framework were evaluated. Experimentally, TIB-PCy_2_ (Compound **16**) was reported to be fully oxidized within 24 h in toluene at room temperature under air, whereas TIB-PPh_2_ (Compound **36**) remained unoxidized after 1 week.^11^ As shown in Figure 10b, the calculated τ were 21.4 and 154.1 ps, respectively, indicating higher oxidation resistance for TIB-PPh_2_. In addition, TIB-PPh_2_ exhibited a larger τ than PPh_3_ (Compound **24**, τ = 51.0 ps), whereas TIB-PCy_2_ exhibited a larger τ than PCy_3_ (Compound **7**, τ = 9.2 ps). These results suggest that the TIB framework may beneficially contribute to oxidation resistance. It should be noted, however, that a direct experimental comparison between TIB-PPh_2_ and PPh_3_, and between TIB-PCy_2_ and PCy_3_, under matched oxidation conditions has not been reported. Therefore, the larger τ predicted for the TIB-substituted phosphines relative to the corresponding non-TIB phosphines illustrate the potential of the present workflow for pre-experimental prediction, while awaiting experimental verification. Nonetheless, within the TIB framework itself, the relative order of τ (TIB-PCy_2_ < TIB-PPh_2_) is consistent with the experimentally observed difference in air stability. These results suggest that the present workflow correctly captures the experimentally established trend within the TIB series and that the TIB framework may favorably contribute to oxidation resistance.

Taken together, comparison of τ with reported experimental and theoretical observations shows that τ reproduces the experimentally observed oxidation resistance for a broad range of phosphines, including simple alkyl, aryl, aryl–cyclohexyl, and biaryl, and distinctive frameworks such as BoPhoz and TIB, regardless of their structural framework. This finding indicates a key advantage of our workflow: by using PFP-MD, large-scale MD sampling combined with statistical analysis yields a robust predictive metric, enabling prediction of oxidation resistance prior to synthesis.

### 3.3 Global Quantitative Analysis of Chemical Factors Governing Oxidation Resistance

This section extends the validation by providing further support for the chemical validity of τ and statistically characterizes the factors governing τ through a global analysis of all 51 phosphines. Unlike the comparisons in Section 3.2, which relied on available experimental observations and theoretical predictions, all 51 phosphines are systematically examined, including compounds for which no direct oxidation-resistance data have been reported.

#### 3.3.1 Descriptor Correlation Analysis

To statistically characterize the factors governing τ across all 51 phosphines, three descriptors were considered, namely the Mulliken charge on the phosphorus atom (*Q*_P_), the percent buried volume (%*V*_bur_), and the SOMO energy. For the bidentate ligands featuring an alkylated ferrocene (BoPhoz) framework (Compounds **31**, **46**, and **47**), which contain two inequivalent phosphorus atoms, *Q*_P_ and %*V*_bur_ were calculated for the phosphorus atom distal to the ferrocene unit, as molecular oxygen reacted predominantly at that site in the MD simulations.

Scatter plots of log_10_τ against each descriptor, colored by structural category, are shown in Figure 11. For interpretive clarity, the phosphines were classified into five categories: electronic-effects group (PPh_3_ derivatives; Compounds **13**, **17**, **20**, **23**, **24**, **28**, and **32**), steric-effects group (branched/cyclic trialkylphosphines; Compounds **1**, **4**, **7**, **9**, and **12**), Cy → Ph group (Compounds **7**, **18**, **21**, and **24**), heterocyclic group (Compounds **34**, **38**, **39**, **45**, **48** and **51**), and others. The three phosphines for which no oxidation event was observed within the simulation time (τ = ∞; Compounds **49**–**51**) are shown separately above the broken *y*-axis in Figure 11. PCy_3_ (Compound **7**) belongs to both the steric-effects group and the Cy → Ph group, and PPh_3_ (Compound **24**) belongs to both the electronic-effects group and the Cy → Ph group. The predictive power of each descriptor was first assessed across the full data set, and trends within individual structural groups were then examined.

**Figure 11:**
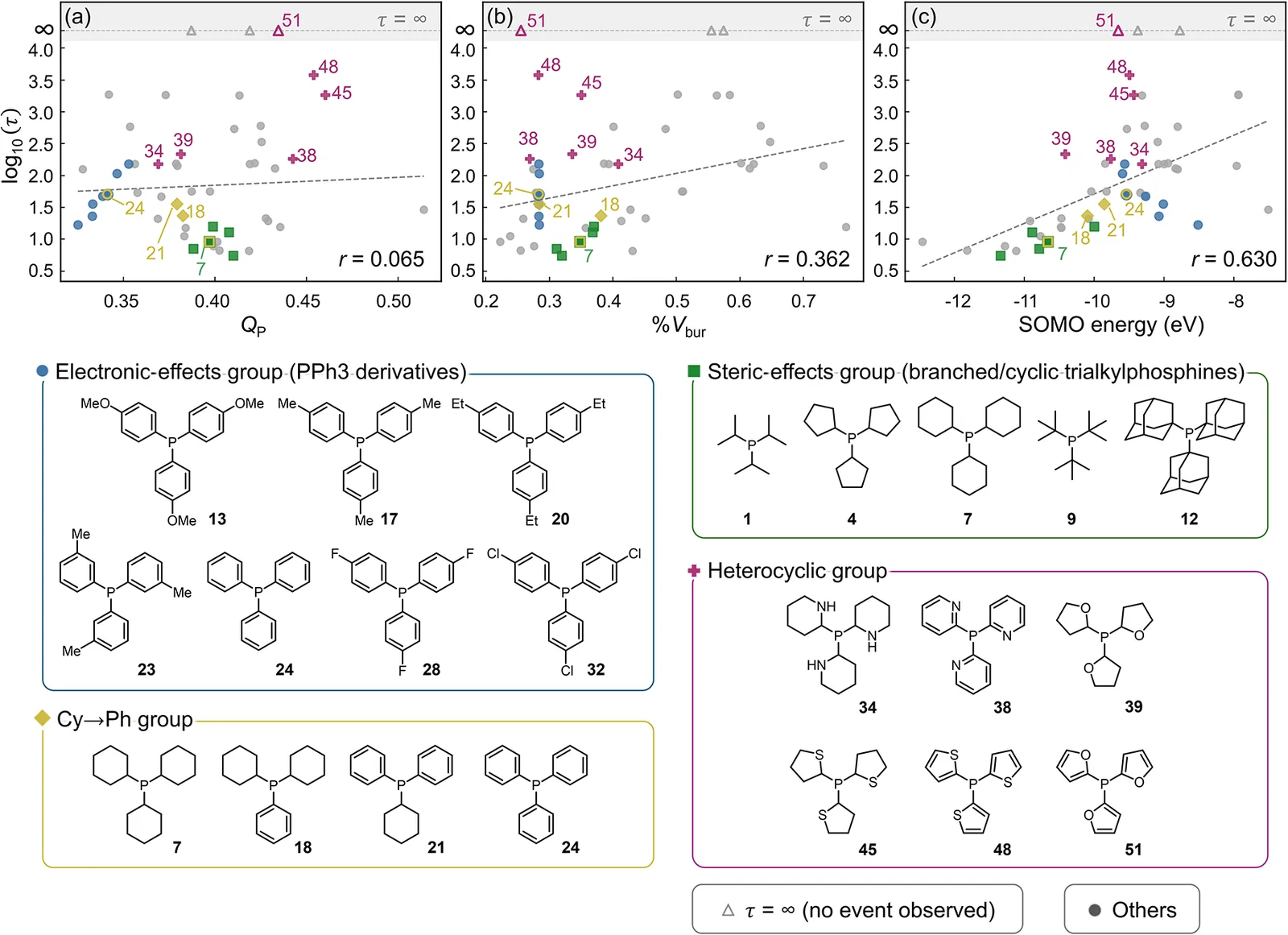
Scatter plots of log_10_τ against (a) *Q*_P_, (b) %*V*_bur_, and (c) SOMO energy, colored by structural category. Blue circles, electronic-effects group (PPh_3_ derivatives; Compounds **13**, **17**, **20, 23**, **24**, **28**, and **32**). Green squares, steric-effects group (branched/cyclic trialkylphosphines; Compounds **1**, **4**, **7**, **9**, and **12**). Yellow diamonds, Cy → Ph group (Compounds **7**, **18**, **21**, and **24**). Magenta crosses, heterocyclic group (Compounds **34**, **38**, **39**, **45**, **48**, and **51**). Gray circles, others. Open triangles above the broken *y*-axis indicate the three phosphines for which no oxidation event was observed within the simulation time (Compounds **49**–**51**, τ = ∞). These three compounds were excluded from the correlation analysis. Dashed lines indicate the linear regression fit, and *r* denotes the Pearson correlation coefficient (*n* = 48).

As shown in Figure 11a, *Q*_P_ showed a near-zero Pearson correlation with log_10_τ across the full data set (*r* = 0.065), indicating that the Mulliken charge on phosphorus alone cannot discriminate the differences in τ among structurally unrelated phosphines. As shown in Figure 11b, %*V*_bur_ showed a moderate correlation with log_10_τ (*r* = 0.362). In contrast, as clearly seen in Figure 11c, SOMO energy showed the strongest linear correlation with log_10_τ (*r* = 0.630). Phosphines with SOMO energies < – 10 eV all cluster at small log_10_τ, whereas phosphines with SOMO energy > – 10 eV show longer τ. This transition near – 10 eV coincides with the air-stability threshold proposed by Stewart et al.,^38^ demonstrating that τ reproduces the qualitative stability classification of the prior study within a quantitative framework. However, substantial scatter remains among phosphines with SOMO energies above this threshold, suggesting that additional descriptor−τ relationships may be masked in the global analysis.

Trends within each structural category are examined next. In the electronic-effects group, namely tris­(4-methoxyphenyl)­phosphine (Compound **13**), tri­(*p*-tolyl)­phosphine (Compound **17**), tris­(4-ethylphenyl)­phosphine (Compound **20**), tri­(*m*-tolyl)­phosphine (Compound **23**), PPh_3_ (Compound **24**), tris­(4-fluorophenyl)­phosphine (Compound **28**), and tris­(4-chlorophenyl)­phosphine (Compound **32**), all compounds bear three phenyl rings, so the extent of π-conjugation is essentially constant, and the dominant substituent effect is the inductive effect acting on the phosphorus atom. Over this group, %*V*_bur_ remains essentially constant at 28.3–28.4%, as shown in Figure 11b, confirming that steric effects do not materially impact τ within this group. In contrast, *Q*_P_ increased monotonically with τ, as shown in Figure 11a. This trend is consistent with the inductive effect of electron-withdrawing substituents. This effect decreases the electron density at the phosphorus atom, reflected in an increase in *Q*_P_, and thereby lowers the nucleophilicity toward molecular oxygen. Therefore, τ correctly reflects this electronic trend.

SOMO energy, by contrast, showed a trend opposite to *Q*_P_ within this group and correlates negatively with τ, as shown in Figure 11c. This is because the same electron-withdrawing group also lowers the π-orbital energy of the phenyl rings, decreasing SOMO energy. Using SOMO energy alone to evaluate oxidation resistance within this group would therefore give the wrong result, predicting that derivatives bearing electron-withdrawing groups are more susceptible to oxidation. This discrepancy arises not from a change in the extent of π-conjugation but from a shift in the orbital energy level itself.

In the steric-effects group, namely triisopropylphosphine (Compound **1**), tricyclopentylphosphine (Compound **4**), tricyclohexylphosphine (Compound **7**), tri-*tert*-butylphosphine (Compound **9**), and tri­(1-adamantyl)­phosphine (Compound **12**), *Q*_P_ remains nearly constant within a narrow range (0.388–0.410), as shown in Figure 11a, indicating that electronic effects do not account for the differences in τ within this group. In contrast, %*V*_bur_ exhibited an increasing trend with τ, as shown in Figure 11b. This indicates that greater steric bulkiness around phosphorus center hinders the approach of molecular oxygen, thus increasing τ. SOMO energy also showed an overall trend similar to that of %*V*_bur_, rising together with τ, as shown in Figure 11c. This suggests that steric factors also influence the SOMO energy, allowing SOMO energy to partially capture this steric contribution as well.

In the Cy → Ph group, the cyclohexyl groups of tricyclohexylphosphine (Compound **7**, PCy_3_) are sequentially replaced by phenyl to give dicyclohexylphenylphosphine (Compound **18**, PCy_2_Ph), diphenylcyclohexylphosphine (Compound **21**, PCyPh_2_), and PPh_3_ (Compound **24**). Across this group (PCy_3_ → PCy_2_Ph → PCyPh_2_ → PPh_3_), τ increased markedly with the number of phenyl substituents (τ = 9.2, 23.5, 36.0, and 51.0 ps, respectively). Aromatic frameworks are generally known to provide electronic stabilization,^54^ and SOMO energy likewise increased monotonically along this series, as shown in Figure 11c, reflecting the stepwise expansion of electronic delocalization into the aromatic π system. As a result, both SOMO energy and τ reflect the aromatic substituent effect. The observed trend is also in line with previous reports that aromatic phosphines exhibit high oxidation resistance and elevated SOMO energies.^38^ By contrast, when oxidation resistance is assessed on the basis of *Q*_P_ and %*V*_bur_ alone, as shown in Figures 11a and 11b, PPh_3_, which has the highest electron density at the phosphorus atom and the smallest steric bulk among the four compounds, would be predicted to be the most reactive toward molecular oxygen. This prediction is inconsistent with the observation that triarylphosphines such as PPh_3_ exhibit higher oxidation resistance than trialkylphosphines,^9^ indicating that these simple electronic and steric descriptors fail to capture the aromatic substituent effect. The concurrent decrease in %*V*_bur_ along this series, as bulkier cyclohexyl groups are replaced by flatter phenyl groups, further indicates that the stabilizing effect of aromatic substitution outweighs the reduction in steric shielding. Taken together with the positive %*V*_bur_–τ correlation observed within the steric-effects group, these results support that τ accurately captures electronic and steric contributions to oxidation resistance independently.

In the heterocyclic group, namely tri­(piperidin-2-yl)­phosphine (Compound **34**), tri­(pyridin-2-yl)­phosphine (Compound **38**), tris­(tetrahydro-2-furanyl)­phosphine (Compound **39**), tris­(tetrahydro-2-thienyl)­phosphine (Compound **45**), tri­(2-thienyl)­phosphine (Compound **48**), and tri­(2-furyl)­phosphine (Compound **51**), the ring heteroatom adjacent to phosphorus is expected to lower the electron density at the phosphorus center, increasing *Q*_P_. The resulting decrease in electron density at the phosphorus atom is expected to reduce its nucleophilicity toward molecular oxygen, and hence increase τ. Indeed, all of these compounds show both higher *Q*_P_ and larger τ relative to PPh_3_ (Compound **24**), as shown in Figure 11a. Thus, although *Q*_P_ does not correlate with τ across the full data set, every compound in the heterocyclic group shows both a higher *Q*_P_ and a correspondingly larger τ than PPh_3_. Furthermore, comparing each aromatic heterocycle with its corresponding nonaromatic (hydrogenated) analogue shows that the aromatic species consistently exhibits the larger τ for every heteroatom examined, with Compound **48** (τ = 3768.3 ps) exceeding Compound **45** (τ = 1830.3 ps) in the sulfur pair, Compound **51** (τ = ∞) exceeding Compound **39** (τ = 218.8 ps) in the oxygen pair, and Compound **38** (τ = 183.2 ps) exceeding Compound **34** (τ = 152.5 ps) in the nitrogen pair. This pattern, consistent across all three ring systems containing sulfur, oxygen, and nitrogen, suggests that aromatic substituents enhance oxidation resistance through electronic stabilization via the π-electron system. It further indicates that the aromatic-stabilization effect observed in the Cy → Ph group also holds for the heterocyclic compounds. The higher *Q*_P_ and larger τ observed for these compounds relative to PPh_3_ are further supported by independent experimental evidence comparing three of them with PPh_3_. Crystallographic and NMR studies of Hg­(II) complexes have reported that Compound **51** and Compound **48**, judged from the ^1^J­(^199^Hg–^31^P) coupling constants and P–Hg–P bond angles of their Hg­(II) complexes, are substantially weaker σ-donors than PPh_3_.^55,56^ A related experimental and theoretical study of Compound **38** has similarly reported reduced donor strength at phosphorus associated with its electron-withdrawing pyridyl substituents.^57^ These reports are consistent with the increase in *Q*_P_ calculated in the present study, supporting the chemical interpretation underlying τ. SOMO energy, by contrast, does not correlate with τ within this group, as shown in Figure 11c, with some compounds showing a higher SOMO energy than PPh_3_ and others a lower SOMO energy, irrespective of the trend in τ.

The group-by-group analysis above demonstrates that τ correctly reflects chemically distinct governing factors, namely inductive electronic effect of nucleophilicity (electronic-effects group), steric shielding (steric-effects group), aromatic stabilization (Cy → Ph group), and electron-withdrawing effects within the heterocyclic group. This supports the chemical validity of τ across all structural categories. At the same time, the finding that no single descriptor fully accounts for τ across all structural categories motivates the construction of a multidescriptor quantitative model in the following section.

#### 3.3.2 Machine Learning Model Evaluation

Building on the descriptor-level analysis in Section 3.3.1, we next constructed a multidescriptor regression model combining *Q*_P_, %*V*_bur_, and SOMO energy. Among the regression algorithms compared (Section 2.2), RF regression gave the best predictive performance. As shown in Figure 12, the RF model achieved *Q* ^2^ = 0.587 under LOO–CV. This value indicates that the combination of the three descriptors accounts for roughly half of the variance in τ across this chemically diverse phosphine set. The model captures the broad trends within the electronic-effects, steric-effects, and Cy → Ph groups, which are reproduced with small residuals. The heterocyclic group, by contrast, is less well captured. The model substantially underestimates the oxidation resistance of Compounds **39**, **45**, and **48**, whereas Compounds **34** and **38** are reproduced reasonably well. As shown in Section 3.3.1, SOMO energy does not vary consistently with τ within the heterocyclic group.

**Figure 12:**
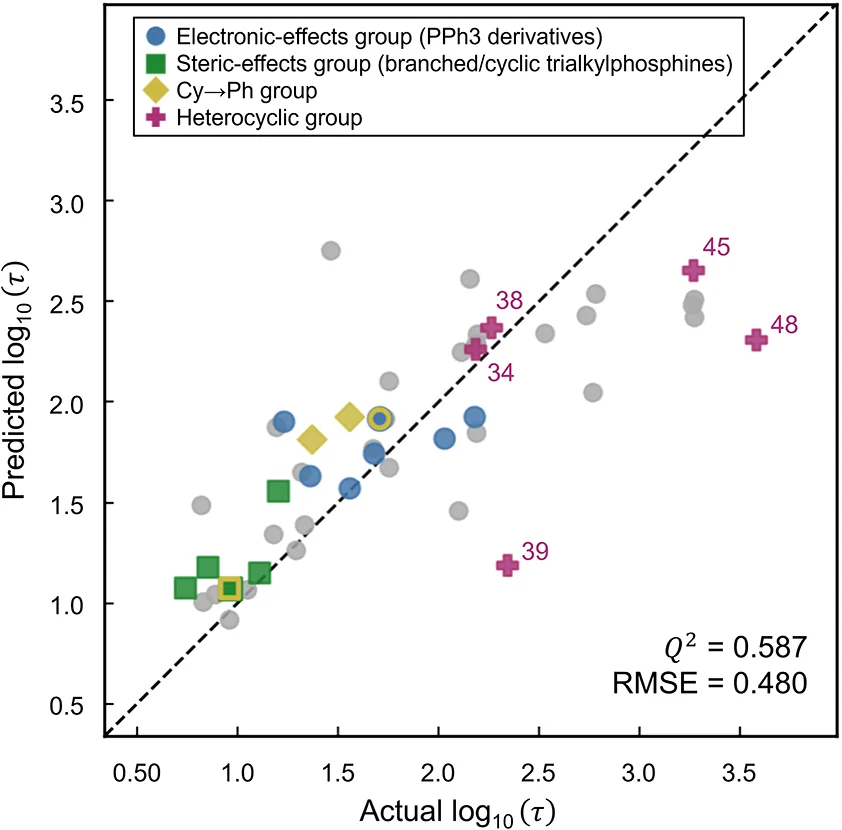
LOO–CV parity plot of predicted versus actual log_10_τ for the random forest (RF) regression model using *Q*_P_, %*V*_bur_, and SOMO energy as descriptors, colored by structural category as defined in Figure 11 (electronic-effects group, blue circles; steric-effects group, green squares; Cy → Ph group, yellow diamonds; heterocyclic group, magenta crosses; others, gray circles). The dashed line represents the parity line (predicted = actual). *Q* ^2^ and Root Mean Square Error (RMSE) denote the cross-validated coefficient of determination and root-mean-square error, respectively (*n* = 48).

To further assess the relative contribution of each descriptor to the RF model, feature importance was evaluated using the Mean Decrease Impurity (MDI).^58^ As shown in Figure 13, SOMO energy contributes more than 60% of the RF model importance, whereas *Q*_P_ contributes approximately 20% and %*V*_bur_ less than 15%. Thus, SOMO energy is the dominant descriptor governing the RF predictions. Notably, *Q*_P_ contributes more to the model than %*V*_bur_ despite exhibiting a near-zero Pearson correlation with τ across the full data set (Figure 11). This suggests that, although *Q*_P_ alone cannot explain the overall trend in τ, it remains locally informative within specific structural groups, particularly the electronic-effects and heterocyclic groups. The relatively large contribution of *Q*_P_ therefore reflects the ability of the RF model to exploit group-specific relationships that are not apparent from global correlation analysis alone. Conversely, because the RF model relies predominantly on SOMO energy, larger prediction errors are expected in structural categories where τ is not well aligned with SOMO energy, such as the heterocyclic group.

**Figure 13:**
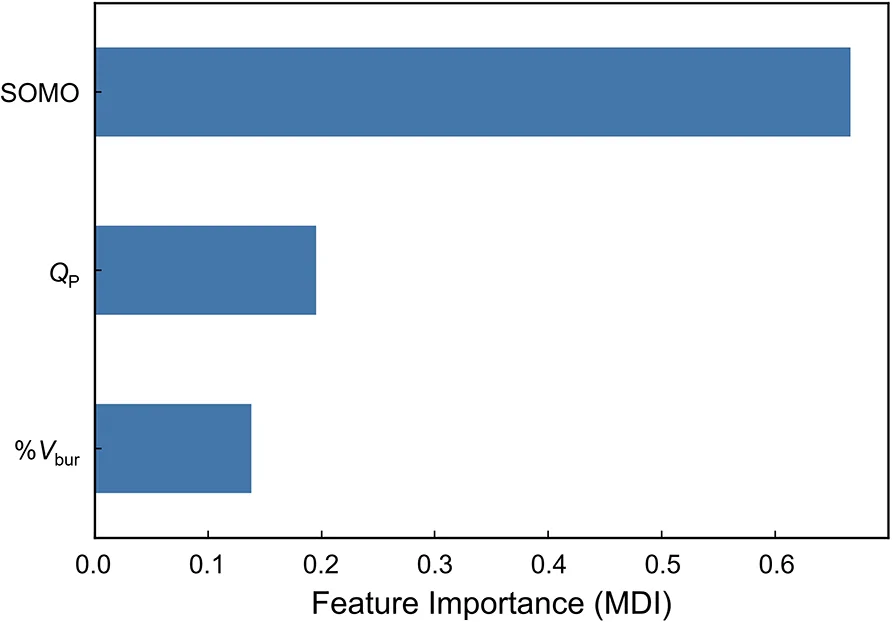
Feature importance of *Q*_P_, %*V*_bur_, and SOMO energy in the RF regression model, evaluated using the Mean Decrease Impurity (MDI).

Taken together, the combination of SOMO energy, %*V*_bur_, and *Q*_P_ reproduces the broad trends of the principal chemical factors governing τ, namely aromatic stabilization, steric shielding, and inductive electronic effect of nucleophilicity, and is therefore useful as a supporting indicator of the chemical validity of τ. At the same time, the *Q* ^2^ of 0.587 also shows that these three descriptors alone cannot fully account for the variance in τ. Descriptors are therefore best regarded as a supporting tool for understanding the chemical factors governing oxidation resistance and for verifying the chemical validity of τ. Although the predictive performance could potentially be improved by incorporating additional descriptors tailored to specific structural frameworks, such extensions would require framework-specific descriptor selection on a case-by-case basis. In contrast, τ obtained from PFP-MD inherently incorporates all effects present in the simulation and therefore provides a more general measure of oxidation resistance across diverse phosphine structures.

### 3.4 Toward Design of New Ligands

Across Sections 3.1–3.3, τ was demonstrated to be chemically valid and to serve as a lifetime descriptor for evaluating oxidation resistance. Electronic, steric, and aromatic factors were appropriately reflected, which is consistent with experimental and theoretical observations of oxidation resistance. To this end, a systematic comparison of τ across all 51 tertiary phosphines was conducted, as shown in Figure 14. This analysis enabled the identification of structural features that promote or diminish oxidation resistance across this chemically diverse set.

**Figure 14:**
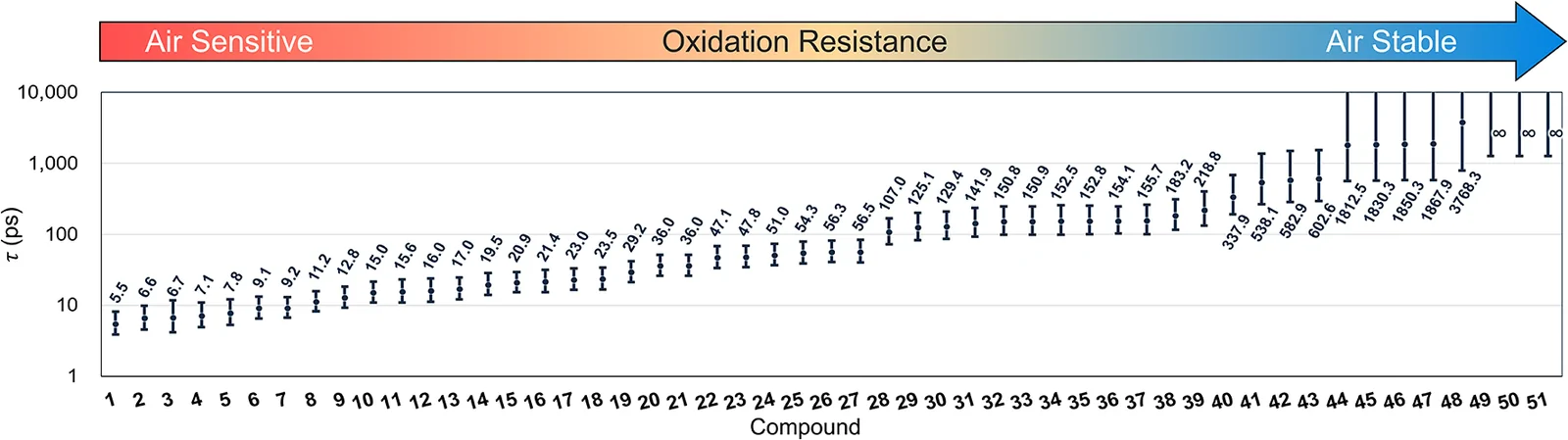
Systematic comparison of τ across 51 tertiary phosphines, allowing identification of structural features that promote or diminish oxidation resistance across a chemically diverse ligand set.

To further characterize the structural origins of large τ, the five ligands with the largest τ (Compounds **47**–**51**) were first examined, and their common features were extracted. Among these top-performing ligands, τ reflect several features: (i) lower electron-donating character (e.g., tri­(2-thienyl)­phosphine, Compound **48**; tri­(2-furyl)­phosphine, Compound **51**),^59^ (ii) greater steric bulk (e.g., the BoPhoz framework, Compound **47**; biaryls bearing *t*-Bu/*i*-Pr, Compounds **49** and **50**), and (iii) the presence of aromatic substituents. Ligands satisfying either (i) or (ii), in combination with (iii), tended to exhibit the largest τ. These observations are consistent with a theoretical understanding in which decreased electron density at phosphorus atom and enhanced steric hindrance suppress reaction with molecular oxygen, thus increasing τ.

By contrast, alkyl phosphines lacking aromatic substituents (Compounds **1–7**, **9**, **11**, **12**, **14**, **16**) generally exhibited smaller τ. Even within alkyl series, however, TIB-PCy_2_ (Compound **16**) exhibited the highest oxidation resistance within its series. This result further underscores the effectiveness of TIB-based phosphines in providing oxidation resistance, indicating that τ can provide design guidance even for phosphines with low oxidation resistance.

The τ provides a comprehensive and consistent scale to evaluate oxidation resistance for both highly and weakly resistant phosphines, exhibiting predictive power that is consistent with experimental and theoretical observations. Accordingly, the proposed workflow provides clear guidance for new ligand design.

It should be noted, however, that the statistical uncertainty associated with τ increases for highly oxidation-resistant phosphines because oxidation events become increasingly rare within the accessible MD time scale. Consequently, compounds exhibiting very large τ tend to show broader 95% confidence intervals than phosphines with moderate oxidation resistance. Nevertheless, for the most oxidation-resistant phosphines identified in this study, the lower bounds of the 95% confidence intervals remain substantially larger than the upper bound of the 95% confidence interval of PPh_3_, indicating that the ranking of highly oxidation-resistant phosphines remains statistically meaningful despite the larger uncertainty.

As a practical extension, for systems where oxidation events are extremely rare under current molecular dynamics settings, the ranking accuracy can be improved with modest adjustments to temperature or pressure or by employing enhanced sampling strategies. Alternatively, increasing the number of independent trajectories offers a direct means of narrowing the 95% CIs, enabling more precise statistical discrimination even between compounds with similar τ. The proposed workflow is readily extensible beyond tertiary phosphines to primary and secondary phosphines, other organic molecules, and metal complexes, and adaptable to other key properties (e.g., water tolerance, basicity, and reducing ability).

## 4 Conclusions

We proposed a practical framework for the a priori assessment of the oxidation resistance of tertiary phosphines. By integrating reactivity sampling via PFP-MD with statistical estimation of τ, this study presented two longstanding challenges in assessing the oxidation resistance of tertiary phosphines: (i) reliance on heuristic trends confined to congeneric series, and (ii) the trade-off between accuracy and cost in conventional computations. Specifically, 40 trajectories per ligand were conducted for 51 tertiary phosphines, enabling statistical analysis at a sampling scale that is effectively infeasible for ab initio MD, with 95% confidence intervals providing a statistically grounded measure of the reliability of each estimated τ. τ appropriately reflects electronic, steric, and aromatic contributions and shows strong consistency with both experimental and theoretical observations. For example, the stabilizing effect of aromatic substitution and the joint role of electron density and steric hindrance in determining rank order were reproduced on a unified τ scale. A global statistical analysis across all 51 phosphines further confirmed the chemical validity of τ, showing that the Mulliken charge on phosphorus, the percent buried volume, and SOMO energy are each associated with τ through distinct, chemically interpretable mechanisms, and a machine learning model combining these descriptors reproduced the broad trend in τ. At the same time, this analysis showed that these descriptors alone cannot fully account for the variance in τ, underscoring that explicit PFP-MD simulation remains necessary to obtain a reliable τ for individual phosphines, particularly for specialized structural frameworks or conformational effects that static calculations cannot resolve. A key strength of the proposed workflow is that it extends to multifactorial reaction systems, allowing cross-series prediction of oxidation resistance with the single lifetime descriptor τ even when multiple factors are intertwined. Accordingly, τ provides a generalizable, predictive tool for pre-experimental design for new ligands.

In summary, τ provides a unified and chemically meaningful scale for evaluating oxidation resistance across structurally diverse phosphines, reproducing experimentally and theoretically recognized trends within a single computational framework. Overall, our workflow advances both the accuracy and throughput of presynthesis computational design and provides a foundation for accelerating catalyst and ligand discovery.

## Supplementary Material







## Notes

Structures (ZIP)
Detailed descriptions of τ calculations, PFP-based molecular dynamics simulation conditions, event-tracking and survival analysis procedures, validation of accuracy, conformer generation, and calculations of molecular electronic and steric descriptors (PDF)
Numerical data underlying the figures and tables, corresponding to Table S8 (XLSX)

## Notes

Toshiya Sato, Matlantis Corporation, 1-6-1 Otemachi, Chiyoda-ku, Tokyo 100-0004, Japan
