# Supplementary material for: Comprehensive Evaluation of Oxidation Resistance for Tertiary Phosphines Using Universal Machine Learning Interatomic Potential-Aided Molecular Dynamics Simulation
Source: JACS Au. 2026 Aug 23;6(9):5102–17. doi: 10.1021/jacsau.6c00783 (PMC13625735; doi:10.1021/jacsau.6c00783)
Supplement: Supplementary file 1 [file au6c00783_si_002.pdf]

Supporting Information for

Comprehensive Evaluation of Oxidation Resistance for Tertiary Phosphines Using Universal Machine Learning Interatomic Potential-Aided Molecular Dynamics Simulation

Toshiya Sato[1,*], Yuri Asai[2], Uika Koshimizu[2], Yuji Hakozaki[2], Kenshin Takei[3], Kazuki Mori[3], Shunsaku Yasumura[4], Tatsushi Ikeda[3], and Yoshihiro Yayama[2,*]

[1]Central Technical Research Laboratory, ENEOS Corporation, 8 Chidoricho, Naka-ku, Yokohama, Kanagawa 231-0815, Japan

[2]AI Innovation Department, ENEOS Holdings, Inc., 8, Chidoricho, Naka-ku, Yokohama, Kanagawa 231-0815, Japan

[3]Department of Chemical System Engineering, The University of Tokyo, Tokyo 113-8656, Japan

[4]Institute of Industrial Science, The University of Tokyo, Komaba 4-6-1, Meguro, Tokyo, Japan 153-8505

*Corresponding authors: sato.toshiya.233@eneos.com, yayama.yoshihiro@eneos.com

Supporting Information Content

S1. Parameters for Conducting the Lifetime Descriptors τ 3

S2. PFP-MD Simulation Details 6

S3. Event-tracking Analysis for 5 different Phosphines 8

S4. Cartesian coordinates for the potential energy diagrams 29

S5. Comparison of Calculated Activation Energies with Experimental Kinetic Data for Representative Phosphines 38

S6. MD Simulations of Biaryl Phosphines Initialized from A-away Conformations 50

S7. Comparison of Regression Models 52

S8. Accuracy Validation 54

S9. Conformer Generation and Descriptor Calculations 56

S1. Parameters for Conducting the Lifetime Descriptors τ

All 51 phosphines investigated in this study, together with their SMILES representations, are shown in Table S1. The τ, τ_{low} and τ_{up} together with the raw data used for its estimation ($\sum_{i=1}^{M} \Delta t_i$ and $\sum_{i=1}^{M} \Delta n_i$) are summarized in Table S2.

Table S1. SMILES representations for all 51 phosphines.

Compound	SMILES
1	CC(C)P(C(C)C)C(C)C
2	CCCCP(CCCC)CCCC
3	CCP(CC)CC
4	C1CCC(P(C2CCCC2)C2CCCC2)C1
5	CP(C(C)(C)C)C(C)(C)C
6	CP(C)C
7	C1CCC(P(C2CCCCC2)C2CCCCC2)CC1
8	CP(C)c1ccccc1
9	CC(C)(C)P(C(C)(C)C)C(C)(C)C
10	CC(C)(C)P(c1ccccc1)C(C)(C)C
11	CCCCCCCCP(CCCCCCCC)CCCCCCCC
12	C1[C@H]2C[C@H]3C[C@@H]1C[C@@](P([C@]14C[C@H]5C[C@H]\ (C[C@H](C5)C1)C4)[C@]14C[C@H]5C[C@H](C[C@H](C5)C1)C4)(C2)C3
13	COc1ccc(P(c2ccc(OC)cc2)c2ccc(OC)cc2)cc1
14	CC(C)(C)CP(C(C)(C)C)C(C)(C)C
15	CP(c1ccccc1)c1ccccc1
16	CC(C)(C)CC(CP(C1CCCCC1)C1CCCCC1)CC(C)(C)C
17	Cc1ccc(P(c2ccc(C)cc2)c2ccc(C)cc2)cc1
18	c1ccc(P(C2CCCCC2)C2CCCCC2)cc1
19	COc1cc(OC)c(P(c2c(OC)cc(OC)cc2OC)c2c(OC)cc(OC)cc2OC)c(OC)c1
20	CCc1ccc(P(c2ccc(CC)cc2)c2ccc(CC)cc2)cc1
21	c1ccc(P(c2ccccc2)C2CCCCC2)cc1
22	CC(C)(C)c1cc(P(c2cc(C(C)(C)C)cc(C(C)(C)C)c2)C2CCCCC2)cc\ (C(C)(C)C)c1
23	Cc1cccc(P(c2cccc(C)c2)c2cccc(C)c2)c1
24	c1ccc(P(c2ccccc2)c2ccccc2)cc1
25	CC(C)(C)c1cc(P(c2cc(C(C)(C)C)cc(C(C)(C)C)c2)C2CCCC2)cc(C(C)(C)C)c1
26	CC(C)(C)P(c1ccccc1C1CCCCC1)C(C)(C)C
27	c1ccc(P(C2CCCCC2)C2CCCCC2)c(C2CCCCC2)c1
28	Fc1ccc(P(c2ccc(F)cc2)c2ccc(F)cc2)cc1

29	CC(C)(C)c1cc(P(c2cc(C(C)(C)C)cc(C(C)(C)C)c2)c2cc(C(C)(C)C)cc\ (C(C)(C)C)c2)cc(C(C)(C)C)c1
30	CC(C)Oc1cccc(OC(C)C)c1-c1ccccc1P(C1CCCCC1)C1CCCCC1
31	CC(N(C)P(C1CCCCC1)C1CCCCC1)C12C3C4C5C1\ (P(c1ccccc1)c1ccccc1)[Fe]43521678C2C1C6C7C28
32	Clc1ccc(P(c2ccc(Cl)cc2)c2ccc(Cl)cc2)cc1
33	c1ccc(-c2ccccc2P(c2ccccc2)c2ccccc2)cc1
34	C1CCC(P(C2CCCCN2)C2CCCCN2)NC1
35	COc1cccc(OC)c1-c1ccccc1P(C1CCCCC1)C1CCCCC1
36	CC(C)(C)CC(CP(c1ccccc1)c1ccccc1)CC(C)(C)C
37	CC(C)c1cc(C(C)C)c(-c2ccccc2P(C2CCCCC2)C2CCCCC2)c(C(C)C)c1
38	c1ccc(P(c2ccccn2)c2ccccn2)nc1
39	C1COC(P(C2CCCO2)C2CCCO2)C1
40	CC(C)c1cc(C(C)C)c(-c2ccccc2P(C(C)(C)C)C(C)(C)C)c(C(C)C)c1
41	CC(C)P(c1ccccc1-c1ccccc1)C(C)C
42	c1ccc2c(P(c3cccc4ccccc34)c3cccc4ccccc34)cccc2c1
43	CC(C)c1cccc(C(C)C)c1-c1ccccc1P(C(C)(C)C)C(C)(C)C
44	c1ccc(-c2ccccc2P(C2CCCCC2)C2CCCCC2)cc1
45	C1CSC(P(C2CCCS2)C2CCCS2)C1
46	CC(N(C)P(c1ccccc1)c1ccccc1)C12C3C4C5C1(P(c1ccccc1)c1ccccc1)\ [Fe]43521678C2C1C6C7C28
47	CC(NP(c1ccccc1)c1ccccc1)C12C3C4C5C1(P(c1ccccc1)c1ccccc1)\ [Fe]43521678C2C1C6C7C28
48	c1csc(P(c2cccs2)c2cccs2)c1
49	CC(C)c1cc(C(C)C)c(-c2ccccc2P(c2ccccc2)c2ccccc2)c(C(C)C)c1
50	CC(C)(C)P(c1ccccc1-c1ccccc1)C(C)(C)C
51	c1coc(P(c2ccco2)c2ccco2)c1

Table S2. τ, τ_{low}, τ_{up}, $\sum_{i=1}^{M}\Delta t_i$ and $\sum_{i=1}^{M}\Delta n_i$ for all 51 phosphines.

Compound	τ (ps)	τ_{low} (ps)	τ_{up} (ps)	$\sum_{i=1}^{M}\Delta t_i$	$\sum_{i=1}^{M}\Delta n_i$
1	5.5	3.9	8.2	153.9	28
2	6.6	4.6	9.9	170.8	26
3	6.7	4.2	11.7	101.0	15
4	7.1	4.9	10.9	178.3	25
5	7.8	5.3	12.1	179.0	23
6	9.1	6.5	13.3	283.0	31

7	9.2	6.7	13.1	311.6	34
8	11.2	8.2	15.8	402.5	36
9	12.8	9.3	18.4	436.9	34
10	15.0	10.9	21.6	496.0	33
11	15.6	11.0	23.1	436.2	28
12	16.0	11.2	23.7	446.8	28
13	17.0	12.2	24.7	526.9	31
14	19.5	14.0	28.3	603.8	31
15	20.9	15.4	29.4	772.8	37
16	21.4	15.3	31.4	643.3	30
17	23.0	16.6	33.0	758.6	33
18	23.5	16.9	34.2	729.7	31
19	29.2	21.2	41.7	992.2	34
20	36.0	26.0	51.7	1186.8	33
21	36.0	26.1	51.8	1189.4	33
22	47.1	33.8	68.6	1461.4	31
23	47.8	34.5	69.2	1531.1	32
24	51.0	36.7	73.7	1630.8	32
25	54.3	39.1	78.5	1736.4	32
26	56.3	40.7	80.9	1857.2	33
27	56.5	40.1	83.4	1639.5	29
28	107.0	72.3	168.0	2354.8	22
29	125.1	83.0	201.1	2501.2	20
30	129.4	85.9	208.1	2588.1	20
31	141.9	92.3	234.7	2554.8	18
32	150.8	99.1	245.7	2865.0	19
33	150.9	99.2	245.9	2867.3	19
34	152.5	98.0	256.3	2593.3	17
35	152.8	99.3	252.7	2751.2	18
36	154.1	103.2	244.7	3236.0	21
37	155.7	100.0	261.6	2647.0	17
38	183.2	116.2	313.3	2931.8	16
39	218.8	132.5	399.4	2844.5	13
40	337.9	191.9	678.7	3379.3	10
41	538.1	263.3	1373.7	3228.6	6
42	582.9	285.2	1488.1	3497.3	6

43	602.6	294.9	1538.4	3615.7	6
44	1812.5	566.3	11944.1	3625.1	2
45	1830.3	571.9	12061.4	3660.7	2
46	1850.3	578.1	12193.2	3700.7	2
47	1867.9	583.6	12309.2	3735.9	2
48	3768.3	790.8	88951.9	3768.3	1
49	∞	1268.5	∞	3800.0	0
50	∞	1268.5	∞	3800.0	0
51	∞	1268.5	∞	3800.0	0

S2. PFP-MD Simulation Details

This section summarizes the full MD protocols used in this study, including
(1) mechanistic-analysis MD conditions (used for PPh_3 in Section 3.1.1), and
(2) quantitative-evaluation MD conditions (used for estimating τ across 51 phosphines).
All MD simulations and geometry optimizations were conducted using the PFP model available through the Matlantis platform.[1,2] Initial configurations were constructed using Packmol.[3]

S2.1 General MD Settings

All MD simulations were conducted using:

- PFP version: 5.0.0
- PFP mode: PBE_PLUS_D3
- Ensemble: NVT
- Thermostat: Nosé–Hoover, time constant = 20.0 fs
- Boundary condition: periodic
- Oxidant: molecular oxygen, treated as intact molecules
- Event tracking: nearest P–O distance tracked at every MD step

S2.2. MD Protocol for Mechanistic Analysis (Section 3.1.1)

These simulations were designed to probe the reaction pathway and intermediates at the MD conditions in this work.

Simulation parameters

- Temperatures: 300 K and 900 K
- Timestep: 0.1 fs
- Simulation length: 20 ps

Initial configuration and velocity

- Phosphine: 5 PPh_3 molecules
- Oxygen: 100 molecules
- Packing: random packing with minimum intermolecular distance 2.0 Å
- Cell density: 1.0 g cm^{-3} (cubic periodic box)

A single initial-velocity set was assigned by Maxwell–Boltzmann distribution at the target temperature using a random seed (seed = 1).

Initial configuration archive

Initial MD structures (ZIP): Initial configurations used for PFP-MD simulations (MD_initial_structures.zip). For the mechanistic-analysis conditions, the initial configurations were provided as compressed archives in the directory:

/si_structure/md_mechanistic_section3_1_1/.

S2.3. MD Protocol for Quantitative Evaluation of Oxidation Resistance (*τ* estimation)

These simulations provided the event-time data used to calculate τ for all 51 phosphines. Trajectories with no reaction by the terminal observation window (95 ps) were treated as right-censored at 95 ps. As a representative example, the 40 sequential trajectories for PPh_3, each consisting of 194 atoms (one PPh_3 molecule and 80 oxygen molecules), required a total wall time of approximately 3 days and 9 hours.

Simulation parameters

- Temperature: 400 K
- Timestep: 0.5 fs
- Simulation length: 100 ps

Initial configuration and velocity

- Phosphine: one phosphine molecule
- Oxygen: 80 O_2 molecules
- Packing: random packing with minimum intermolecular distance 5.0 Å
- Cell density: 0.5 g cm^{-3} (cubic periodic box)

For all phosphines, N_0 = 40 trajectories were generated using 4 different initial configurations and 10 distinct initial velocities. Initial velocities were assigned from the Maxwell–Boltzmann

distribution at the 400 K using random seeds (seed = 1–10) to produce 10 distinct initial-velocity sets per configuration. For a subset of phosphines, some of the 4 initial configurations were identical to one another rather than mutually distinct. Reaction times nonetheless showed no clustering by shared initial configuration, confirming that the resulting trajectories remain mutually independent and that τ and its 95% CIs are unaffected.

Initial configuration archive

Initial MD structures (ZIP): Initial configurations used for PFP-MD simulations (MD_initial_structures.zip). For the quantitative-evaluation conditions, the initial configurations were provided as compressed archives in the directory:

/si_structure/md_quantitative_section3_1_2_onward/.

S3. Event-tracking Analysis for 5 different Phosphines

This section summarizes the event-tracking analysis conducted for 5 different phosphines (Compound **7**, **12**, **22**, **24**, and **36**) to evaluate whether the reaction behavior observed in the main text—oxygen approach within 2.0 Å leading to irreversible P–O bond formation—is consistently reproduced across phosphines. For each phosphine, 40 trajectories were conducted under the quantitative-evaluation conditions. The full set of trajectories are listed in Figure S1-5.

Across all phosphines, once the nearest P–O distance first reached 2.0 Å, a P–O bond was formed and no dissociation of this bond was observed for the remainder of the simulation. This persistence, irreversible retention of the oxidized state was consistently observed in all reactive trajectory.

- Tricyclohexylphosphine (Compound **7**)

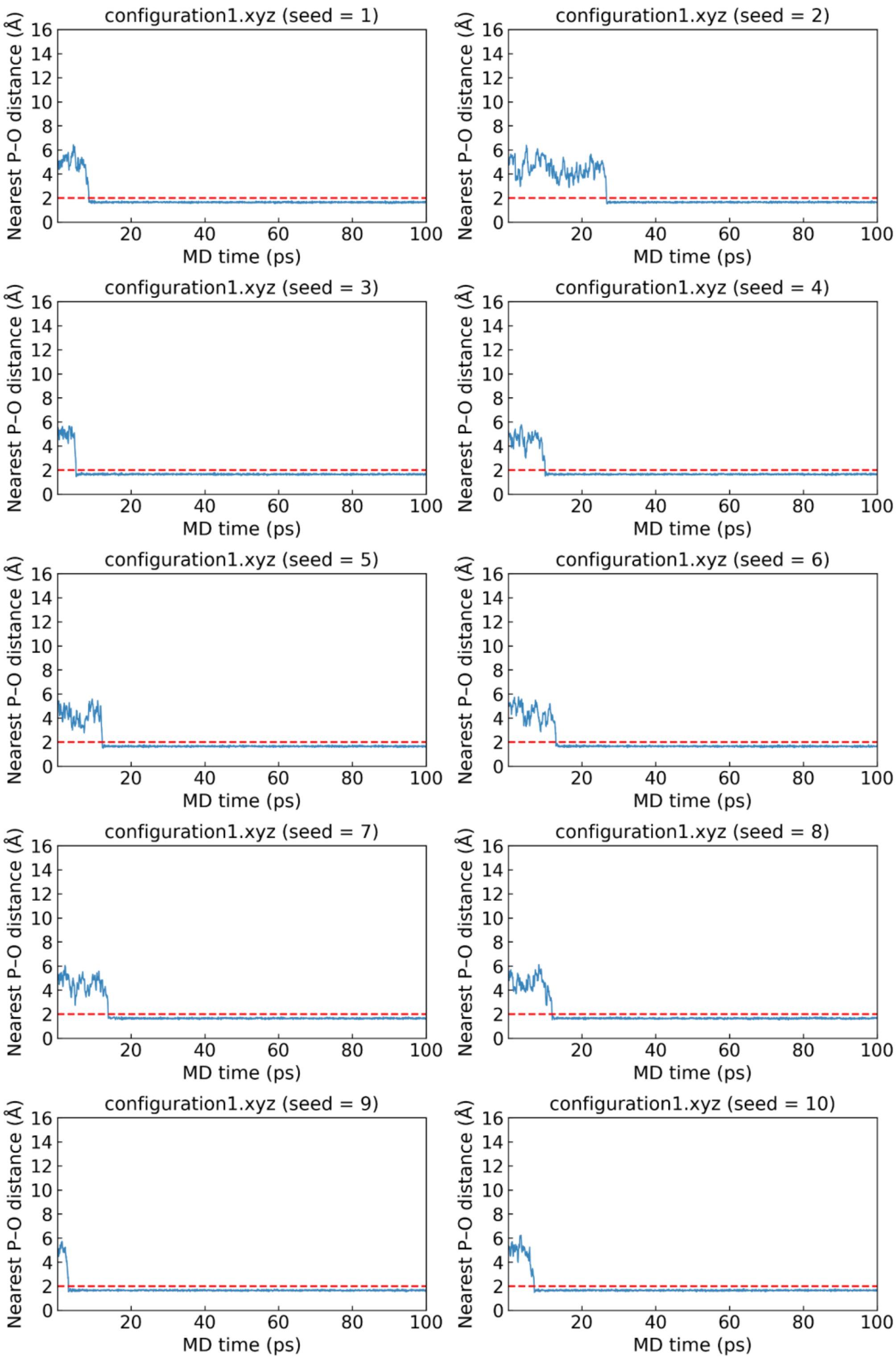

Figure S1. Time-dependent P–O distances for Tricyclohexylphosphine (Compound **7**) under quantitative-evaluation conditions (40 trajectories).

- Tricyclohexylphosphine (Compound **7**)

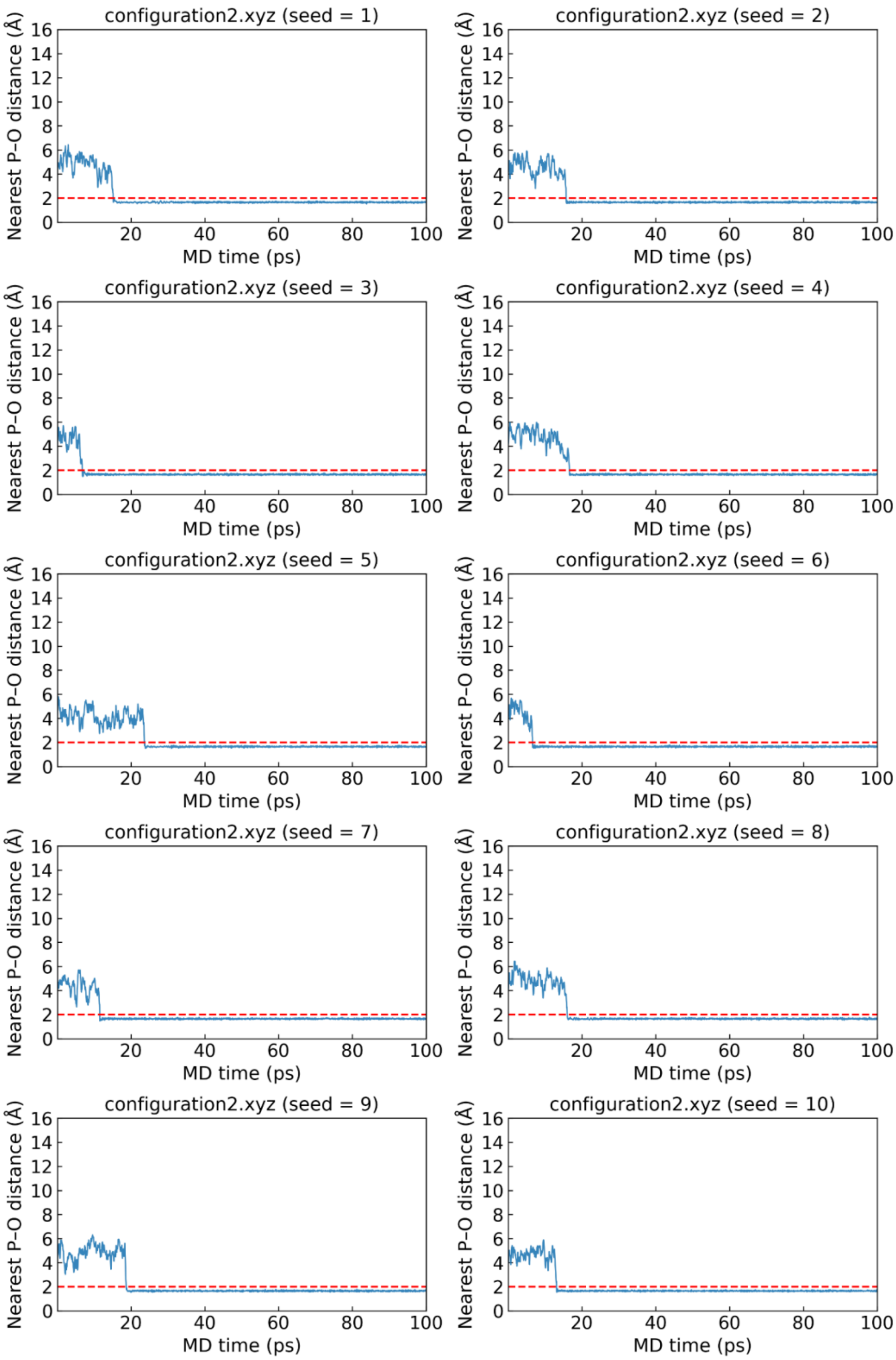

Figure S1 (continued).

- Tricyclohexylphosphine (Compound **7**)

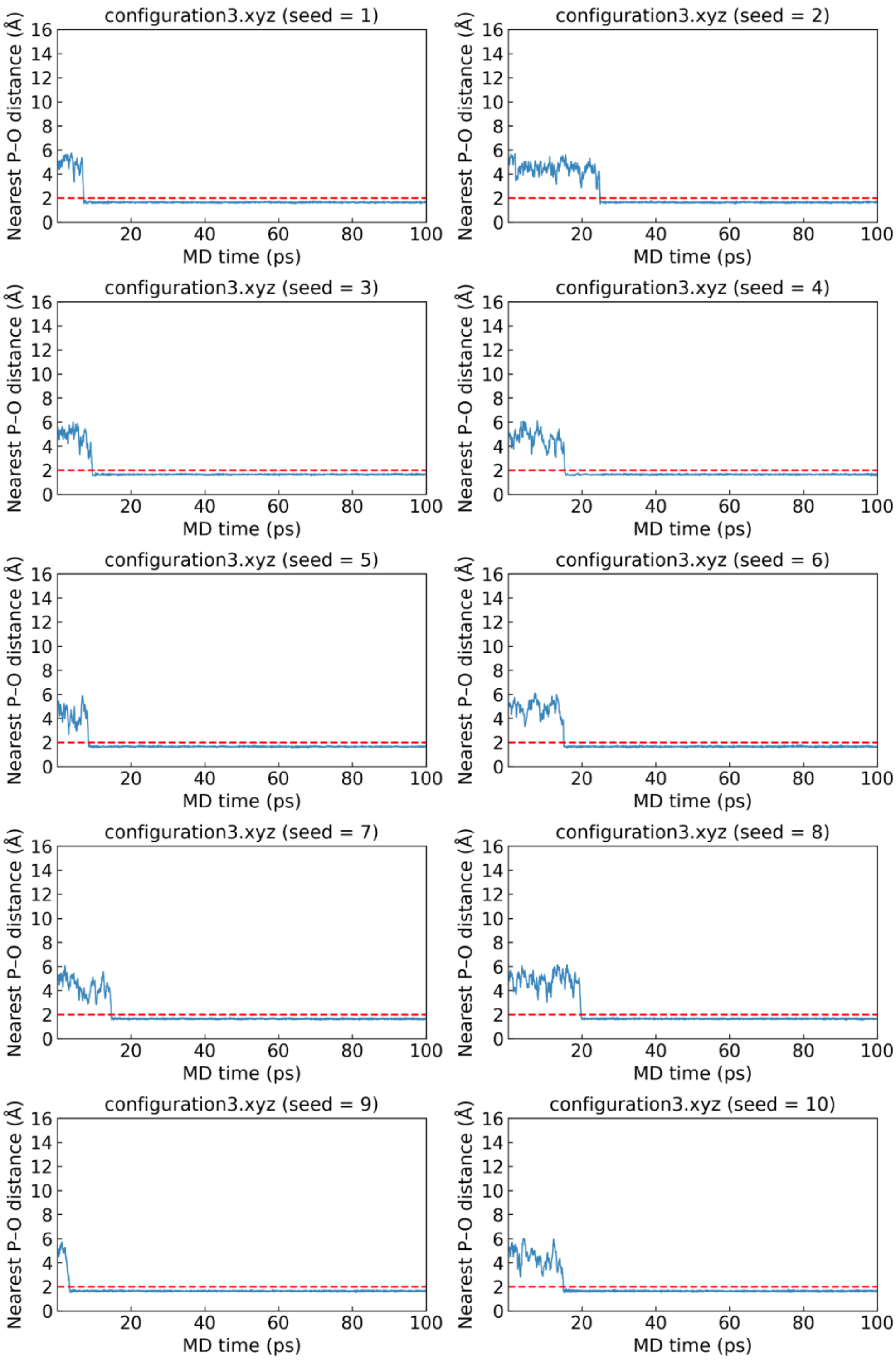

Figure S1 (continued).

- Tricyclohexylphosphine (Compound **7**)

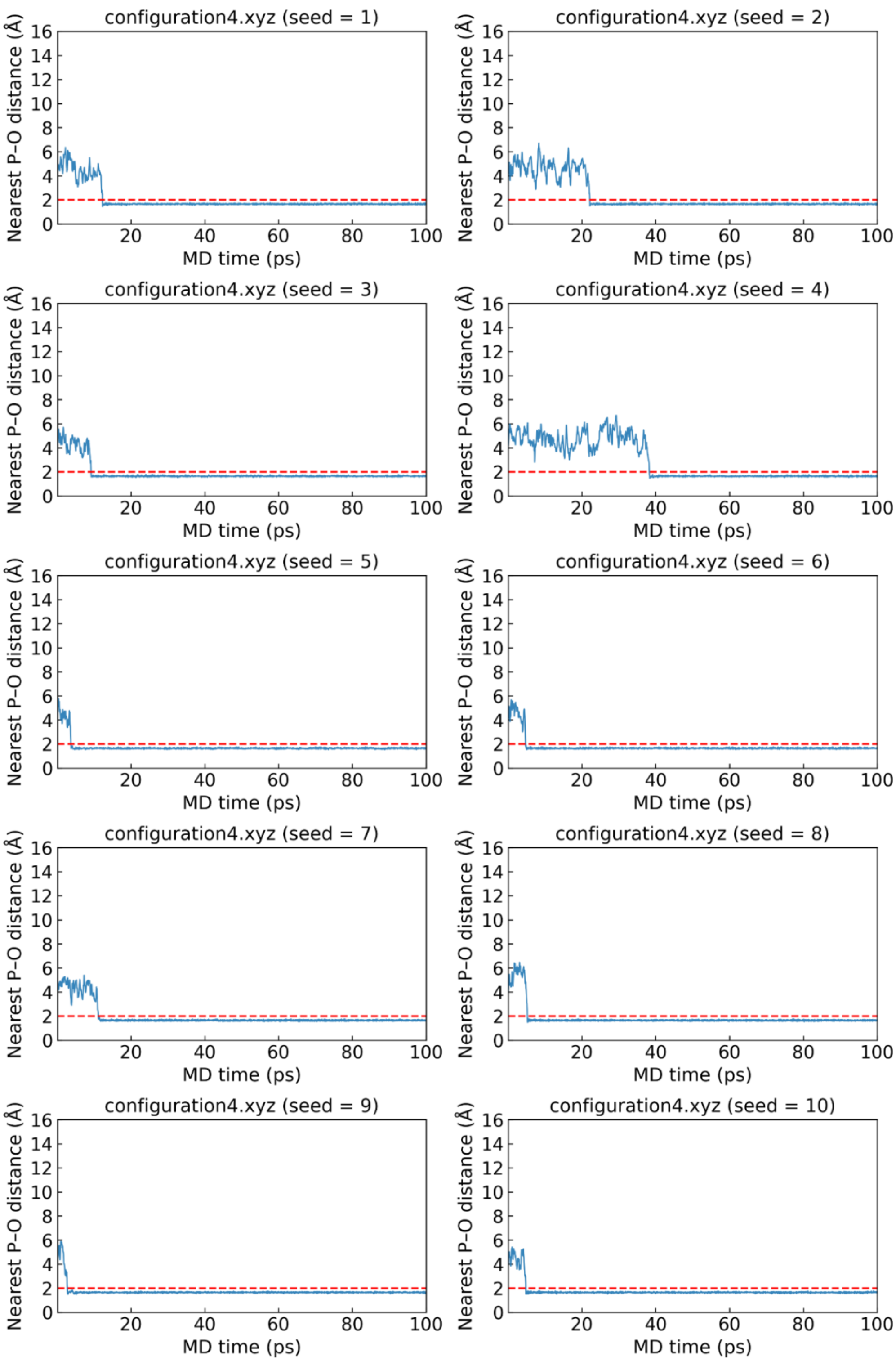

Figure S1 (continued).

- Tri(1-adamantyl)phosphine (Compound **12**)

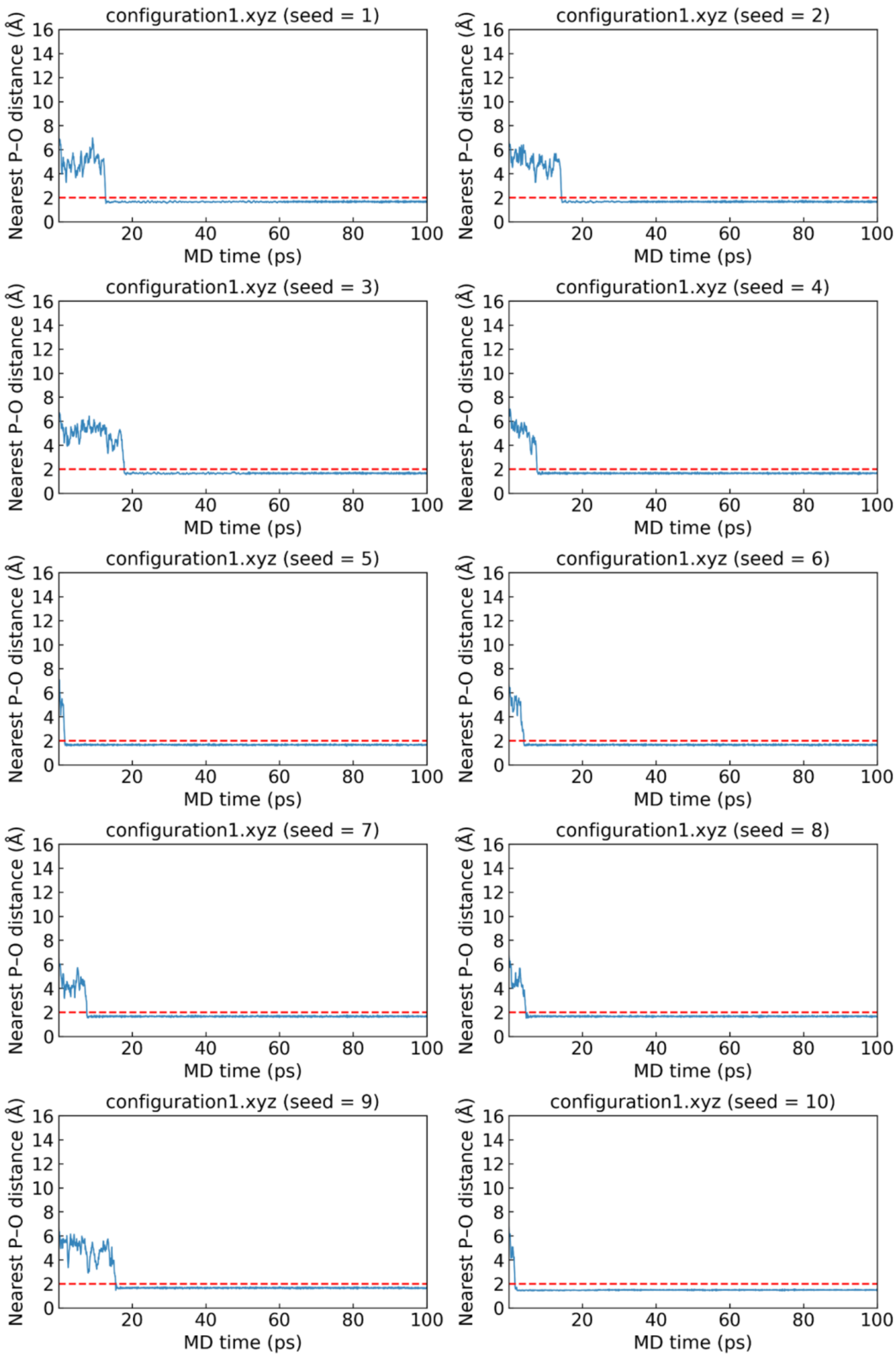

Figure S2. Time-dependent P–O distances for Tri(1-adamantyl)phosphine (Compound **12**) under quantitative-evaluation conditions (40 trajectories).

- Tri(1-adamantyl)phosphine (Compound **12**)

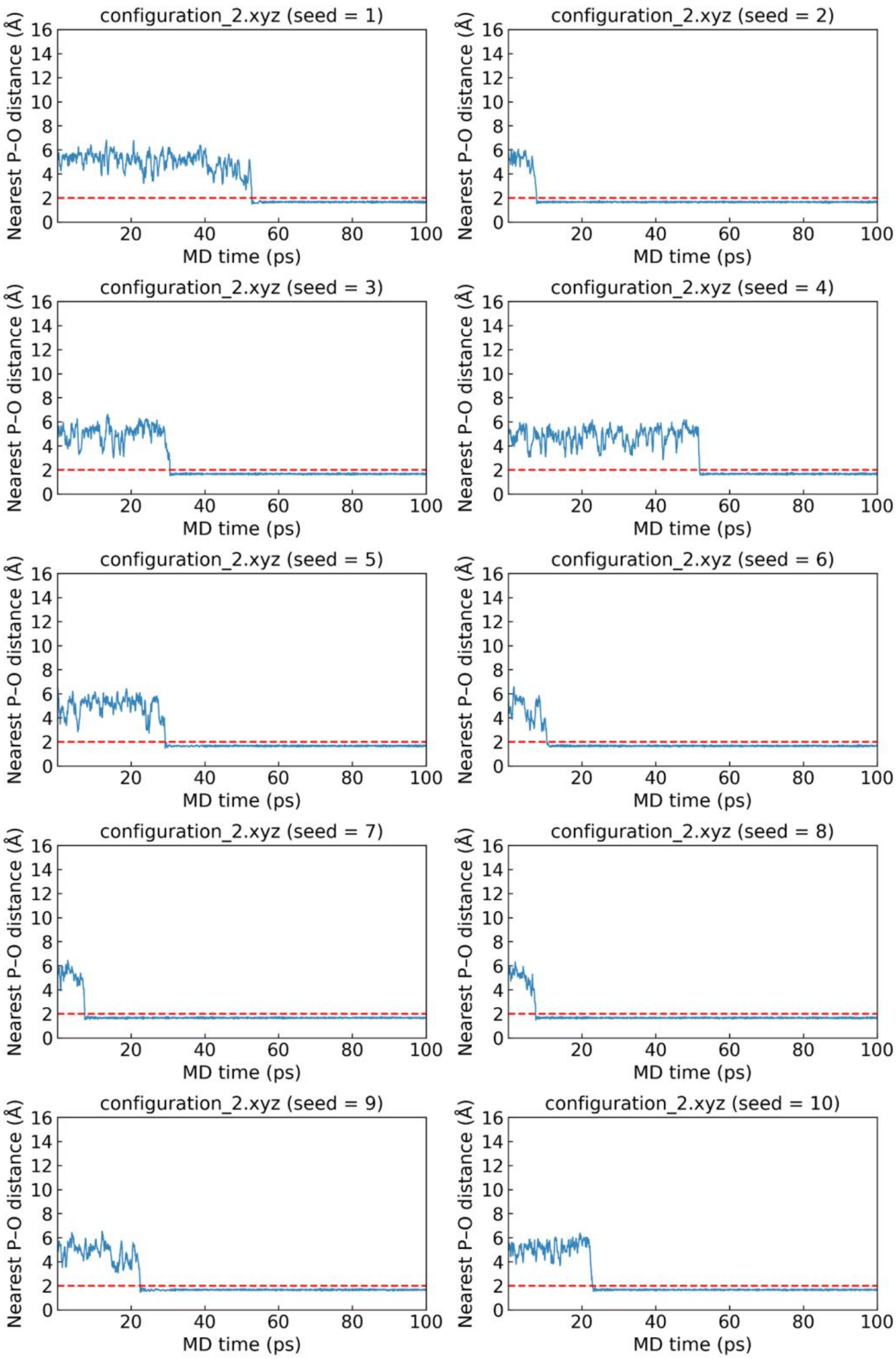

Figure S2 (continued).

- Tri(1-adamantyl)phosphine (Compound **12**)

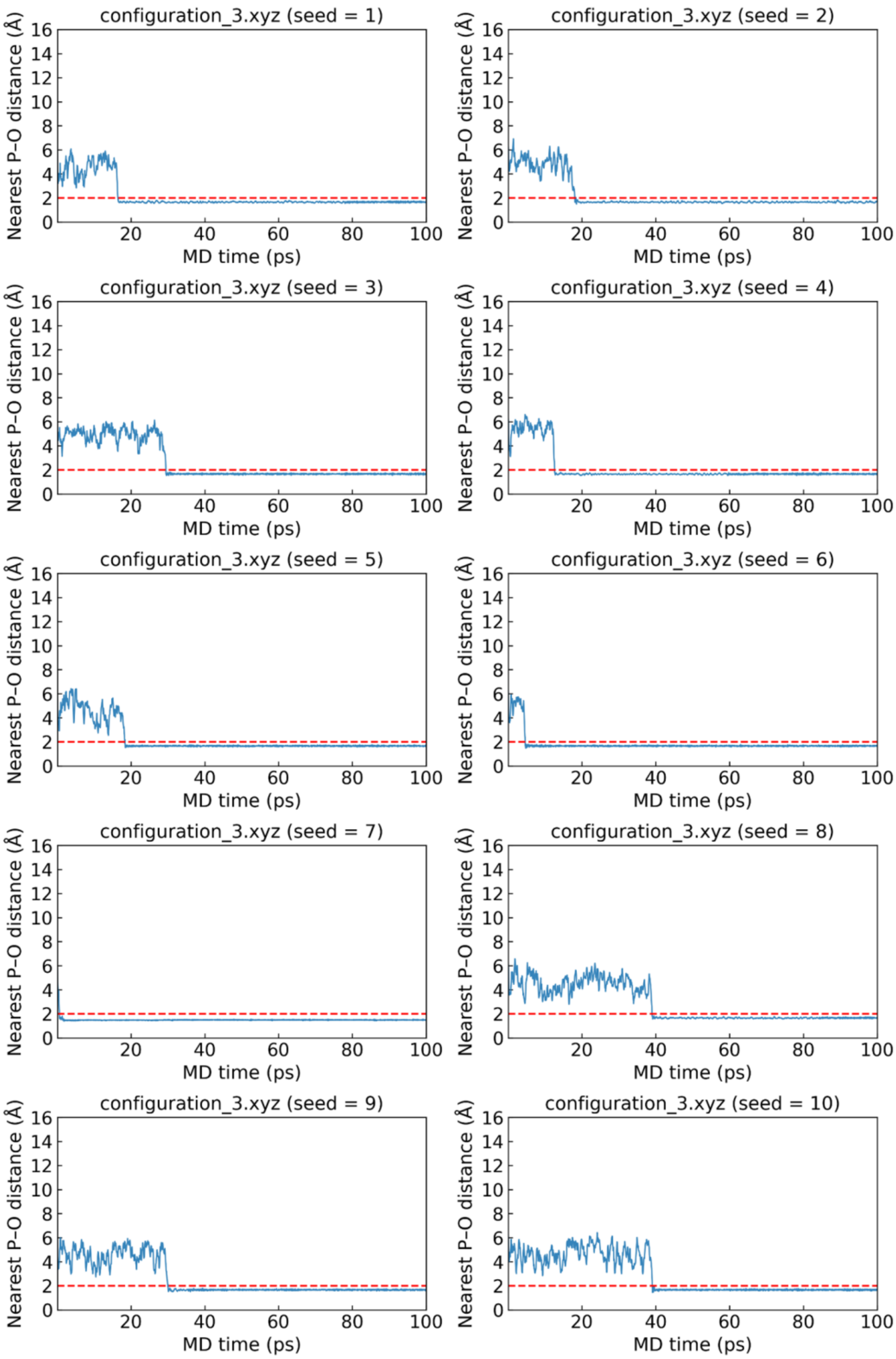

Figure S2 (continued).

- Tri(1-adamantyl)phosphine (Compound **12**)

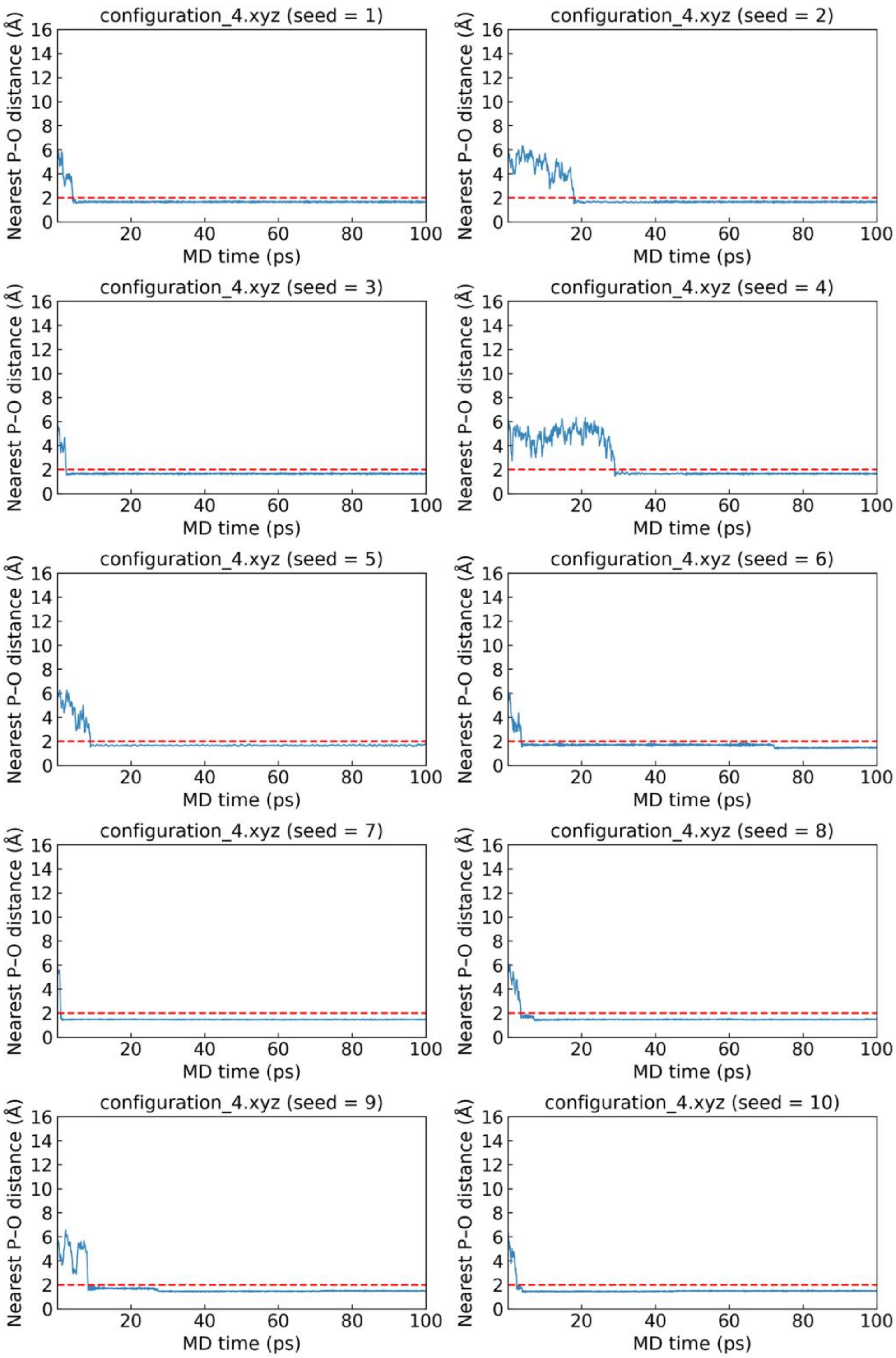

Figure S2 (continued).

- CyTyrannoPhos (Compound **22**)

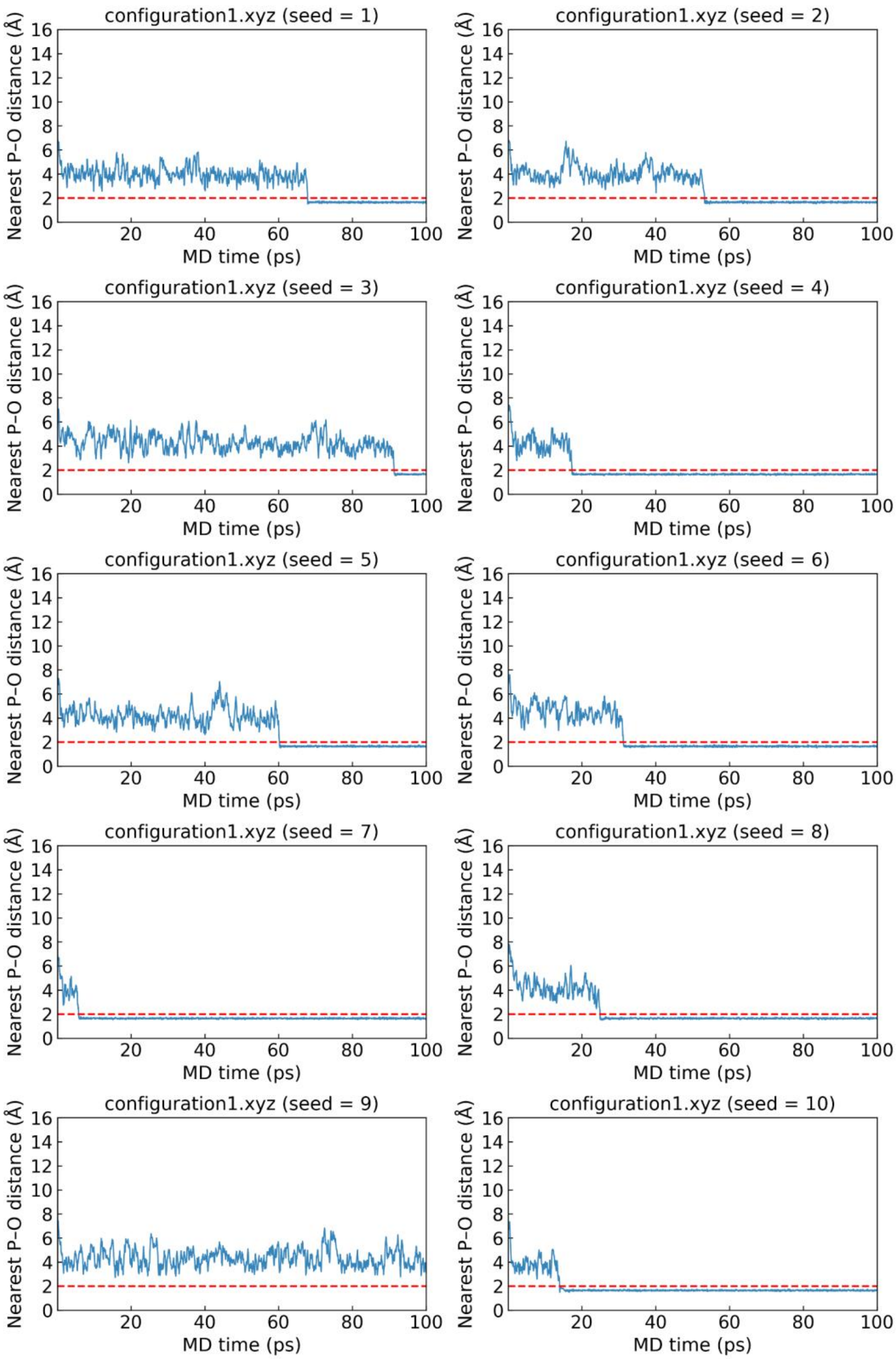

Figure S3. Time-dependent P–O distances for CyTyrannoPhos (Compound **22**) under quantitative-evaluation conditions (40 trajectories).

- CyTyrannoPhos (Compound **22**)

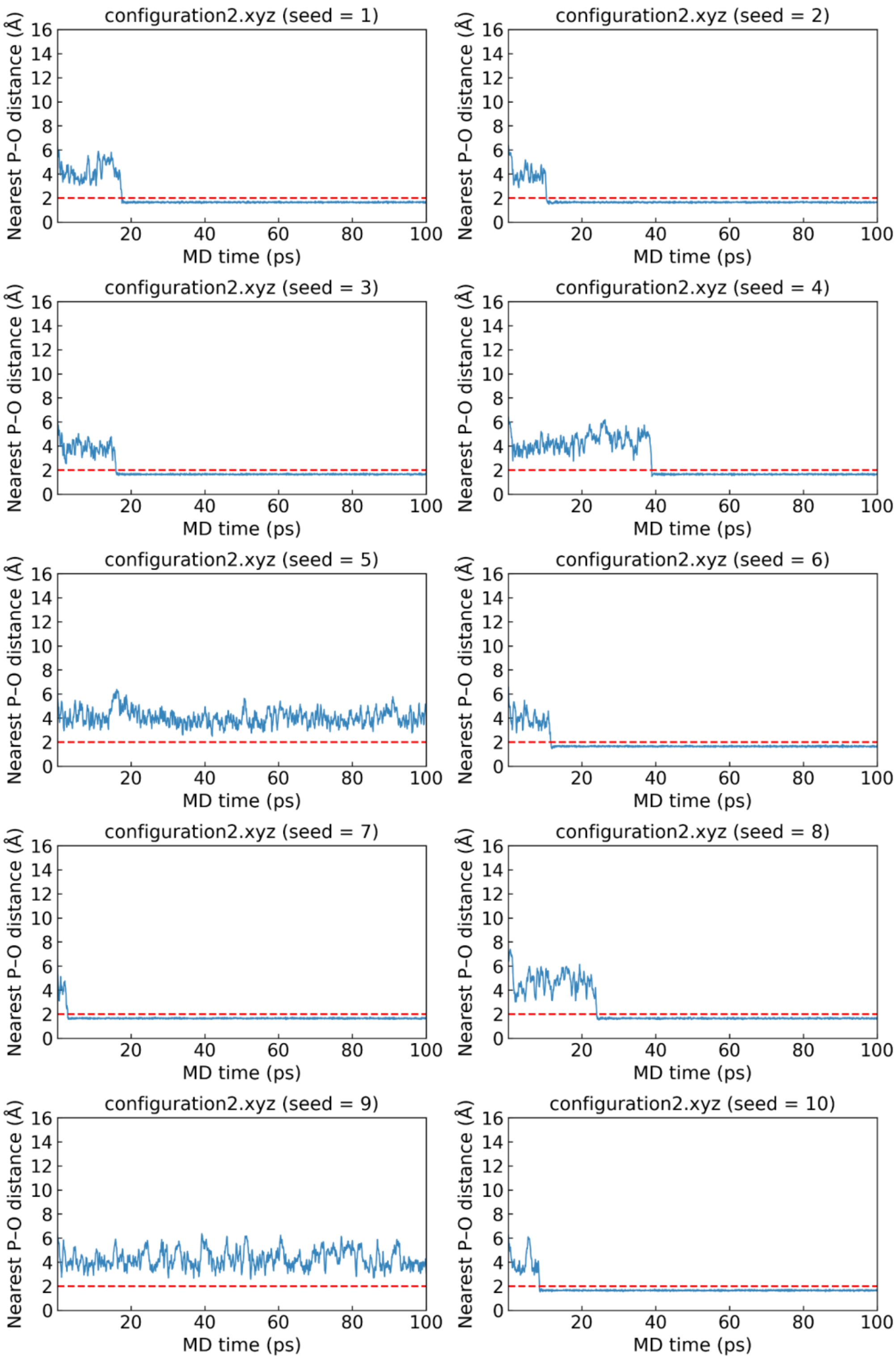

Figure S3 (continued).

- CyTyrannoPhos (Compound **22**)

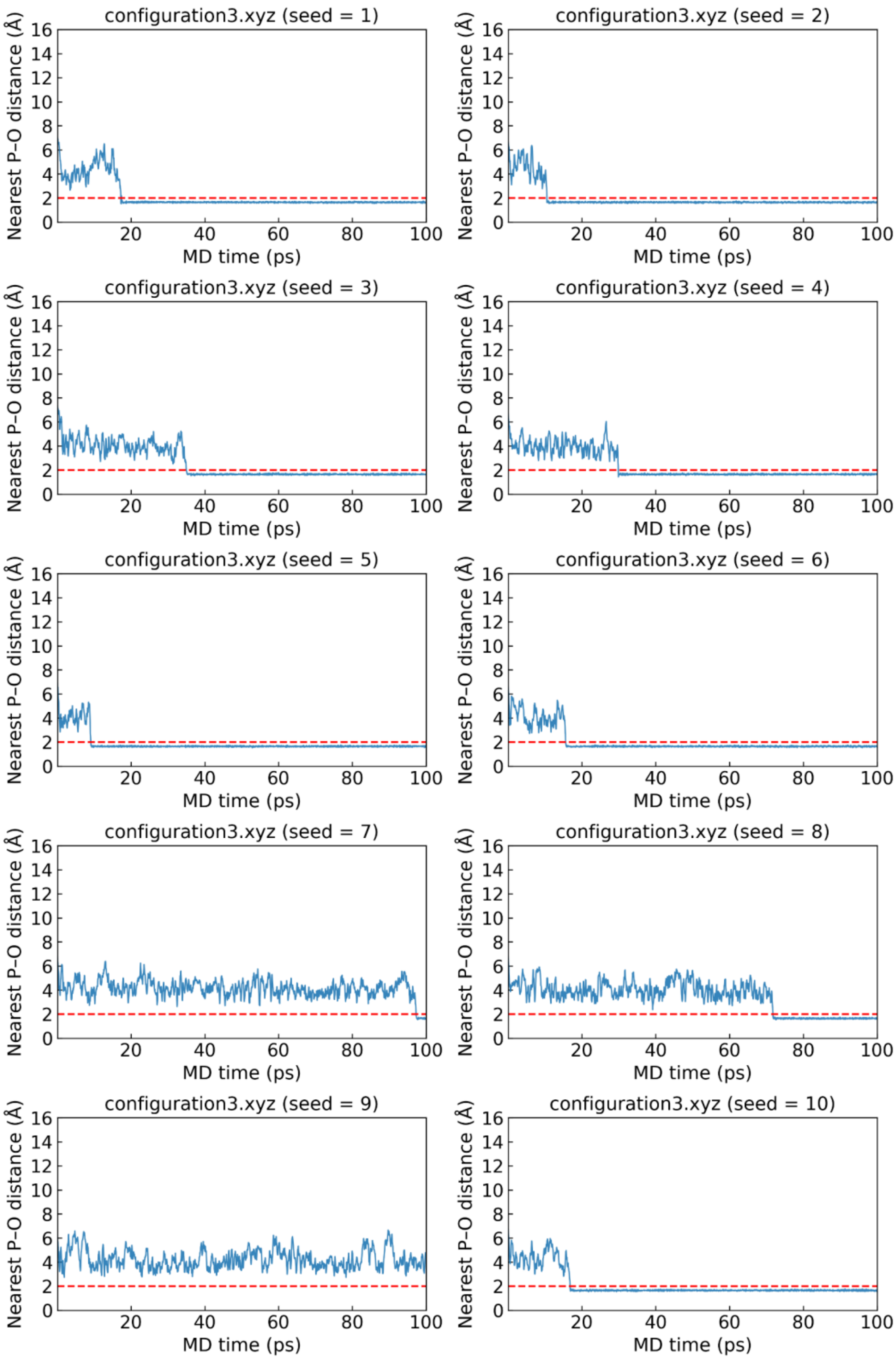

Figure S3 (continued).

● CyTyrannoPhos (Compound **22**)

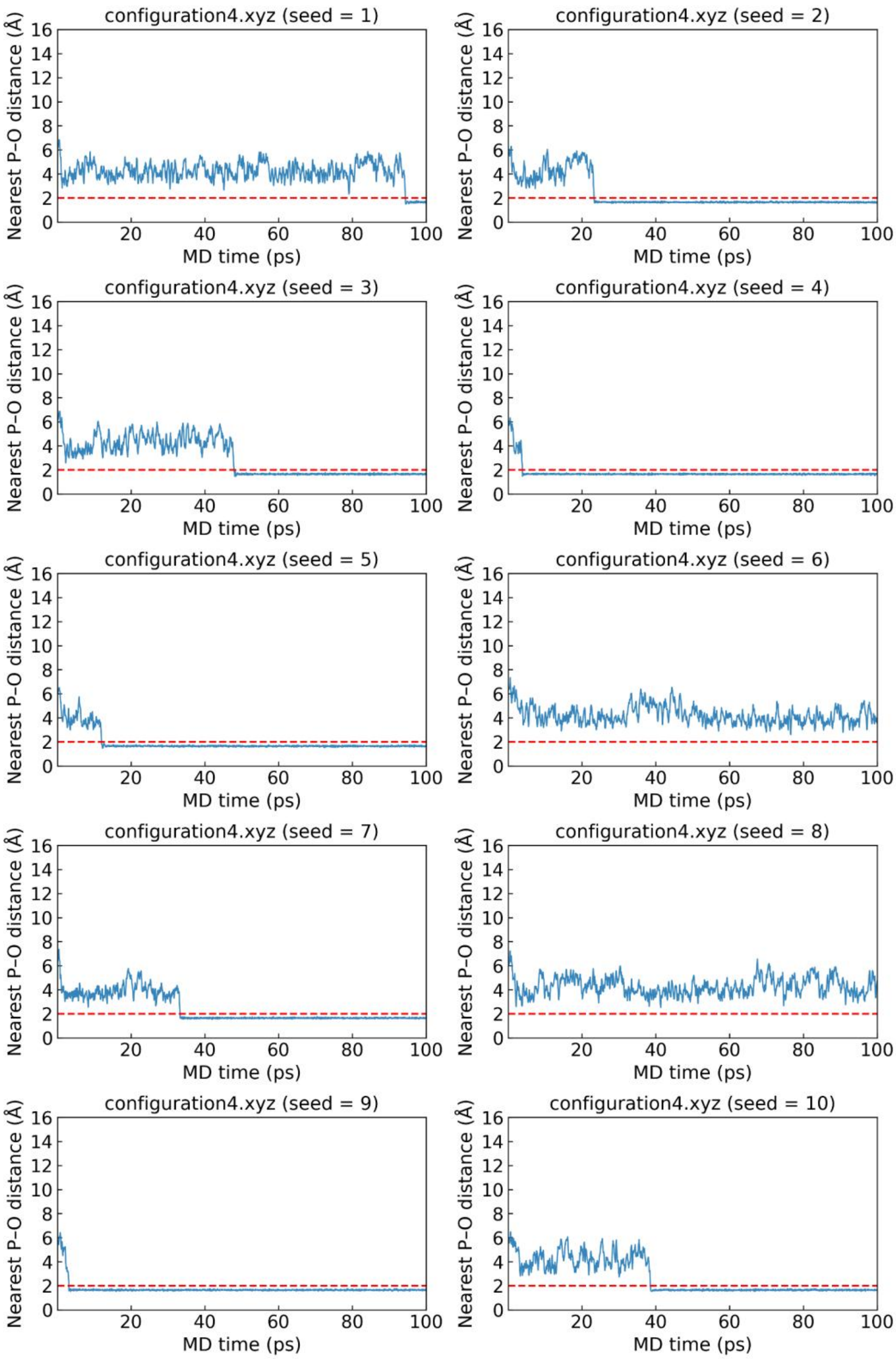

Figure S3 (continued).

- PPh_3 (Compound **24**)

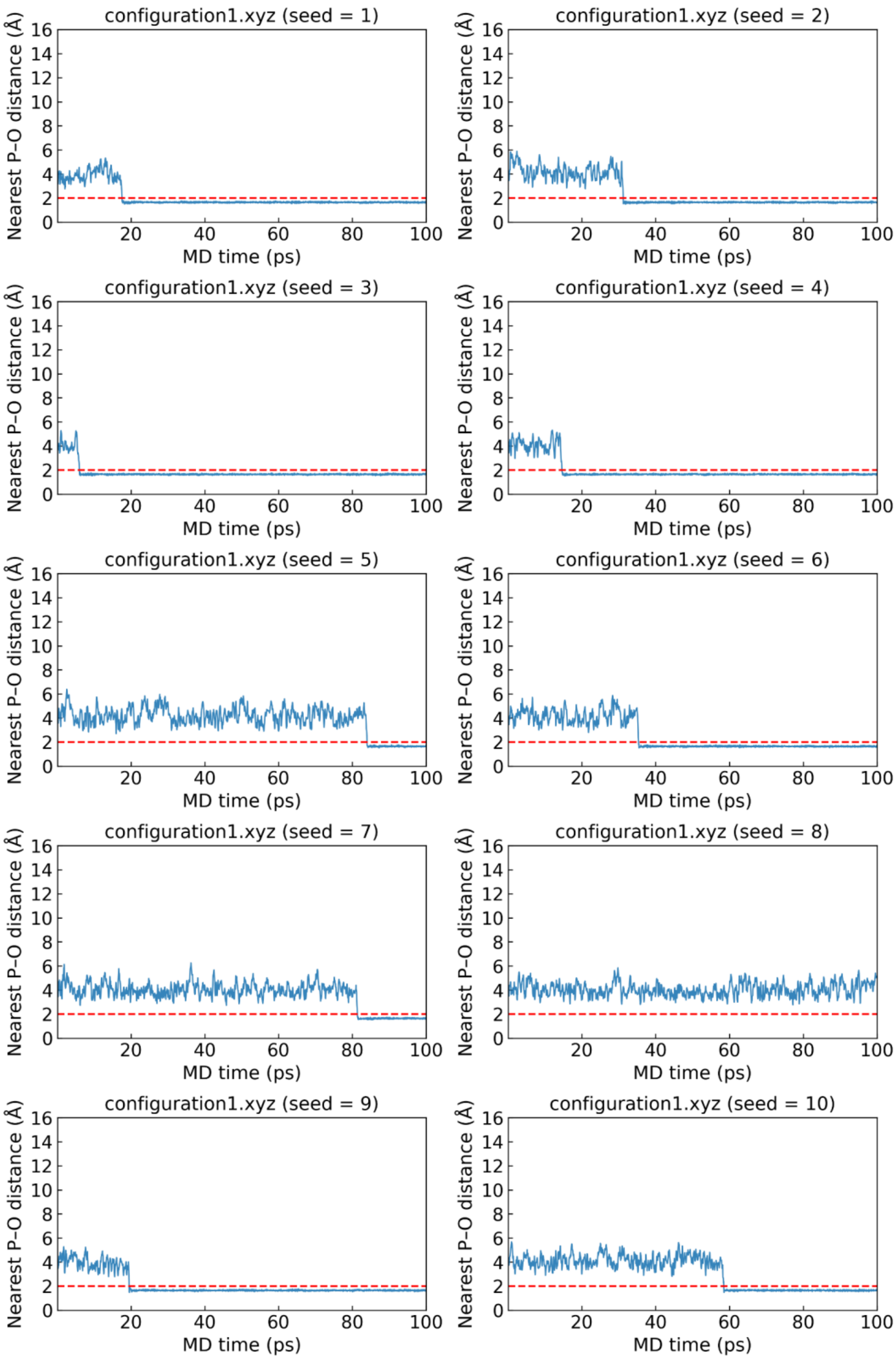

Figure S4. Time-dependent P–O distances for PPh_3 (Compound **24**) under quantitative-evaluation conditions (40 trajectories).

- PPh_3 (Compound **24**)

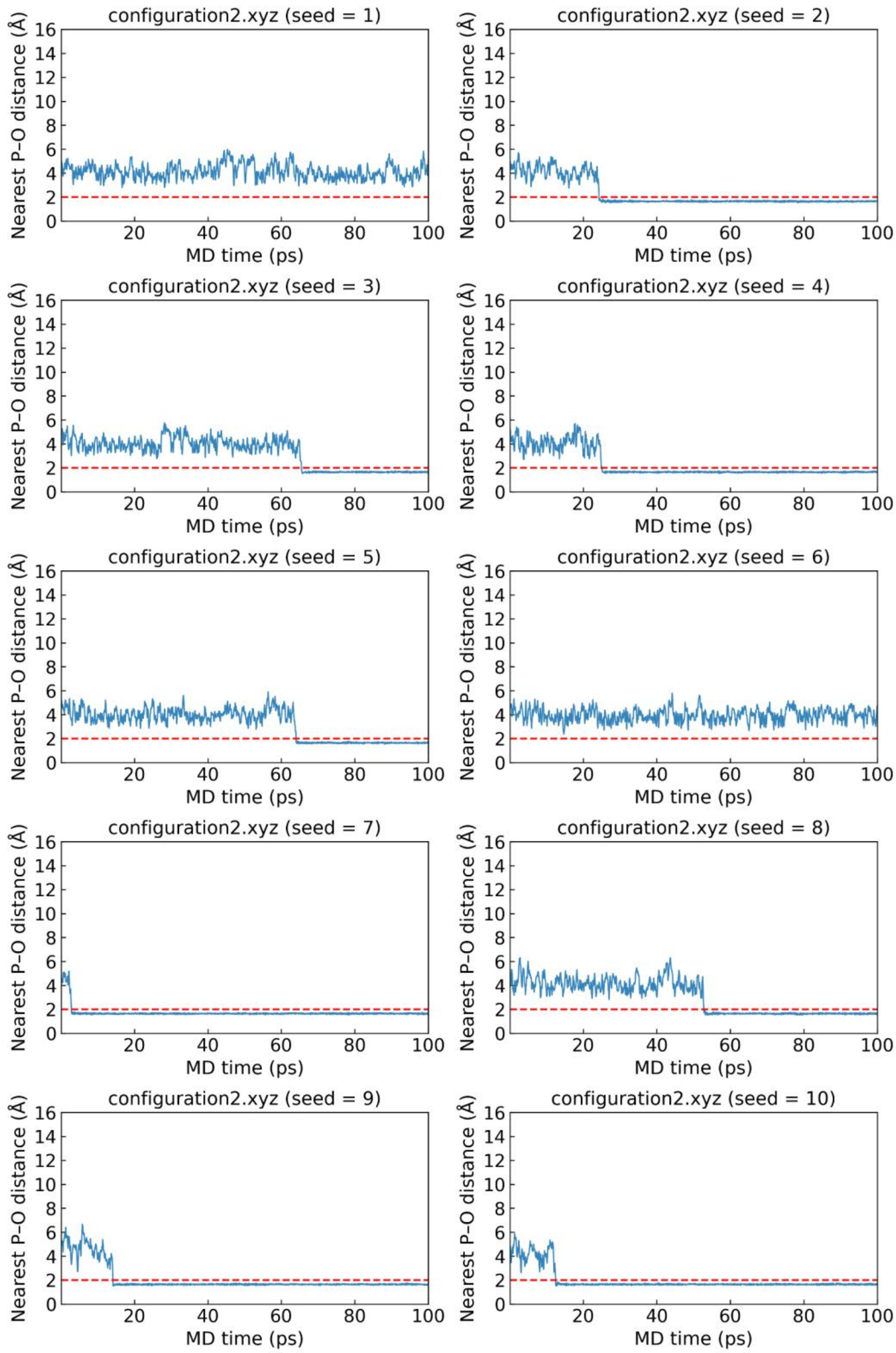

Figure S4 (continued).

- PPh_3 (Compound **24**)

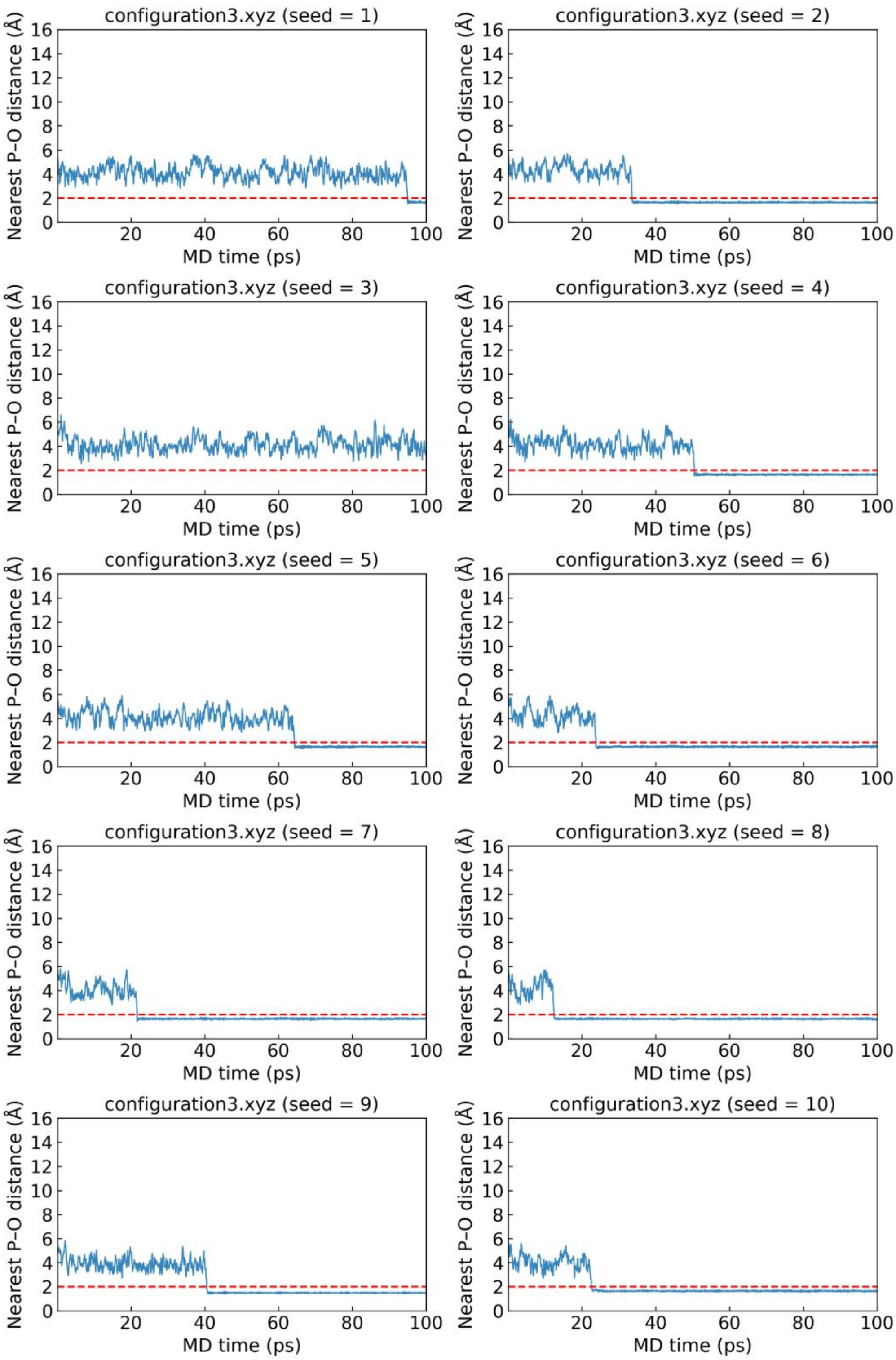

Figure S4 (continued).

- PPh_3 (Compound **24**)

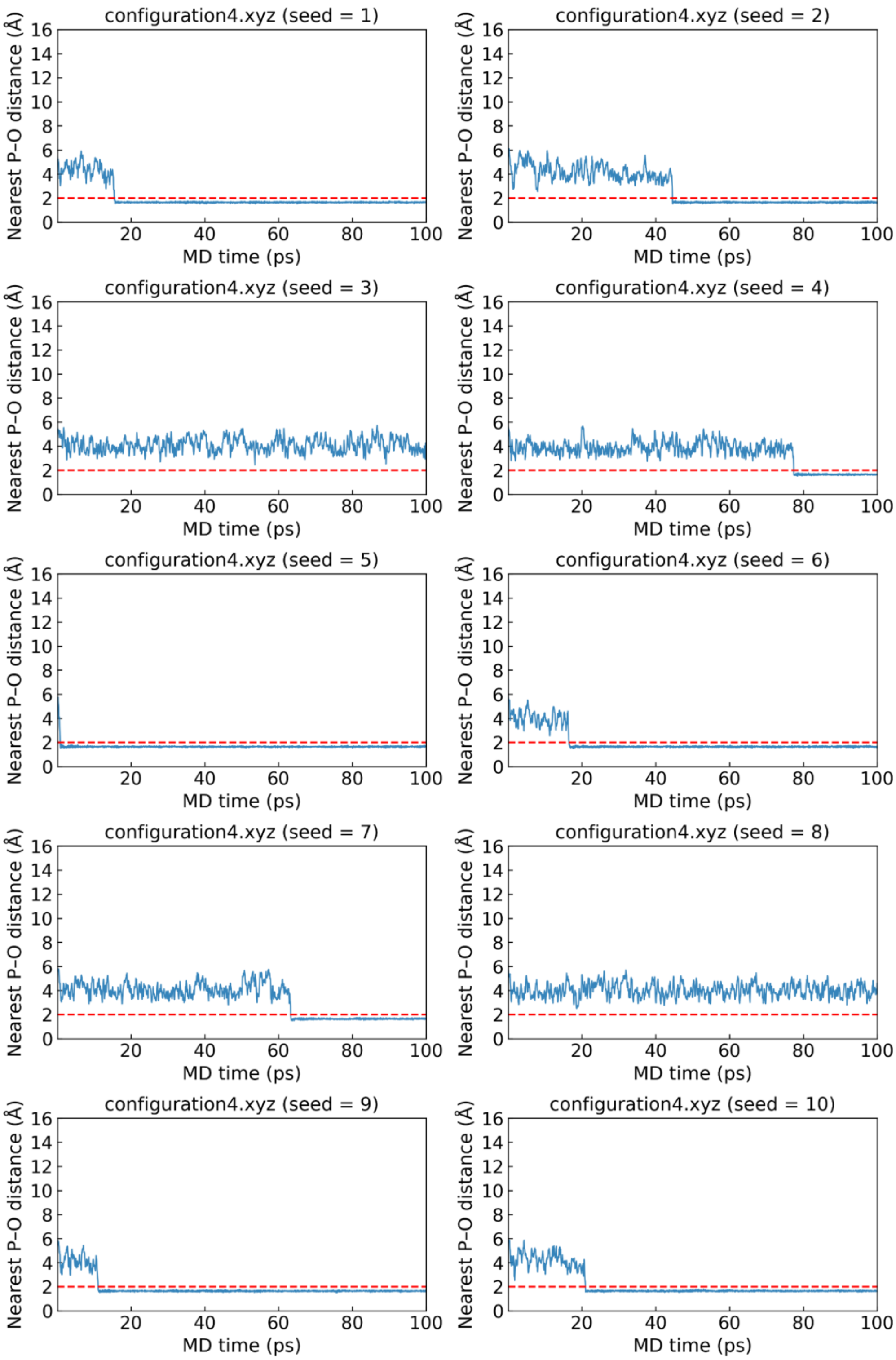

Figure S4 (continued).

- TIBPPh$_2$ (Compound **36**)

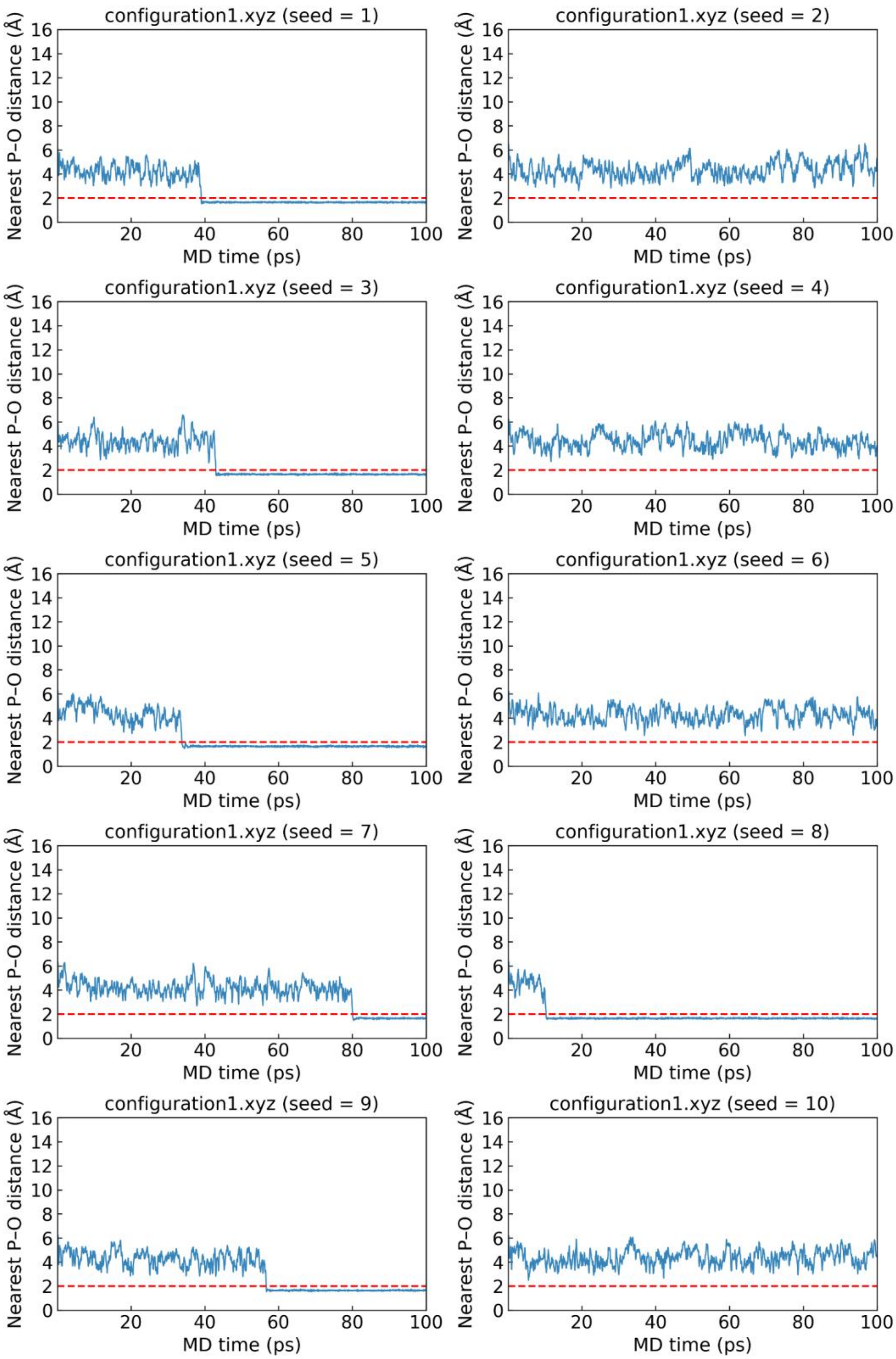

Figure S5. Time-dependent P–O distances for TIBPPh$_2$ (Compound **36**) under quantitative-evaluation conditions (40 trajectories).

- TIBPPh$_2$ (Compound **36**)

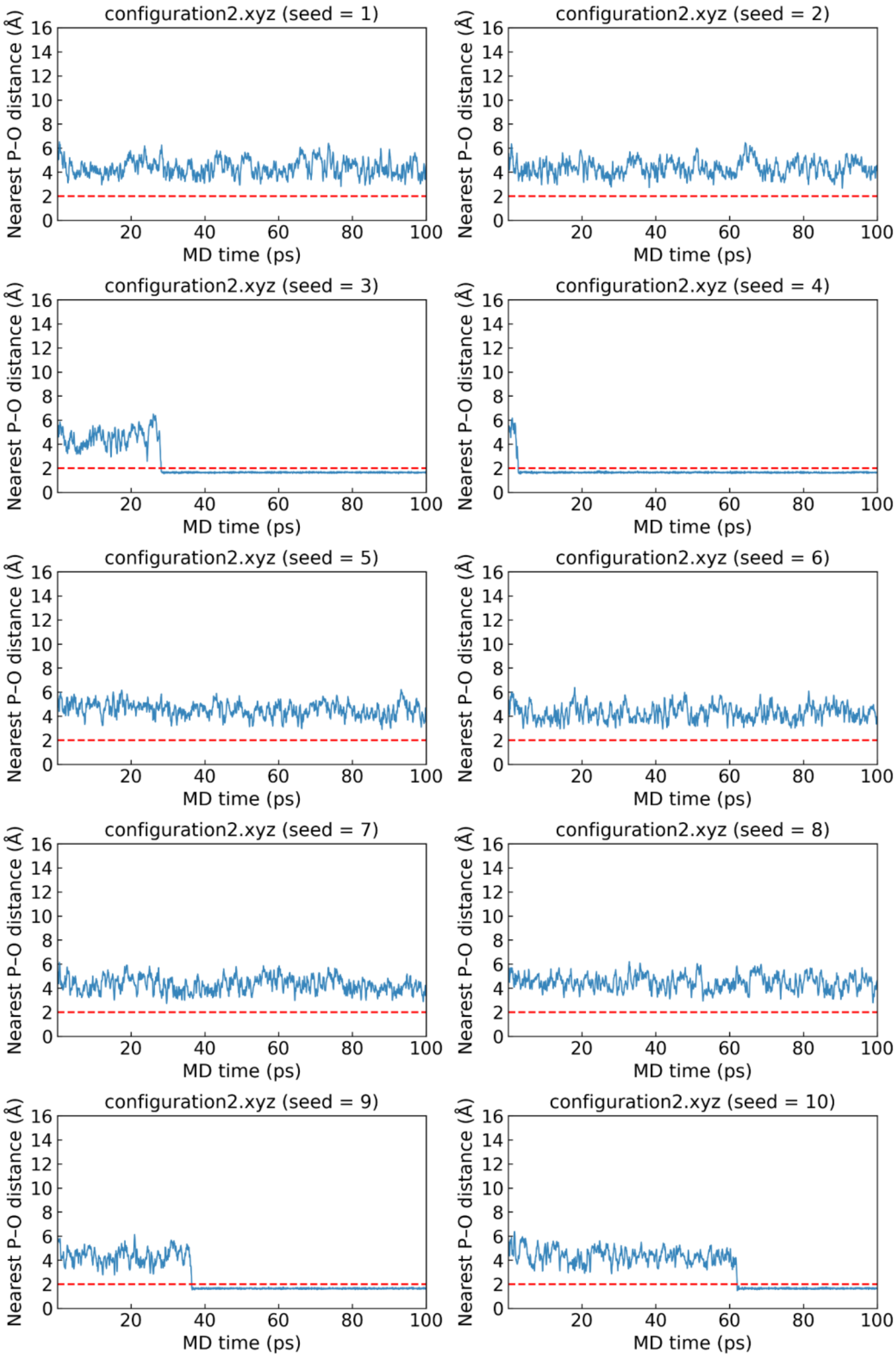

Figure S5 (continued).

- $TIBPPh_2$ (Compound **36**)

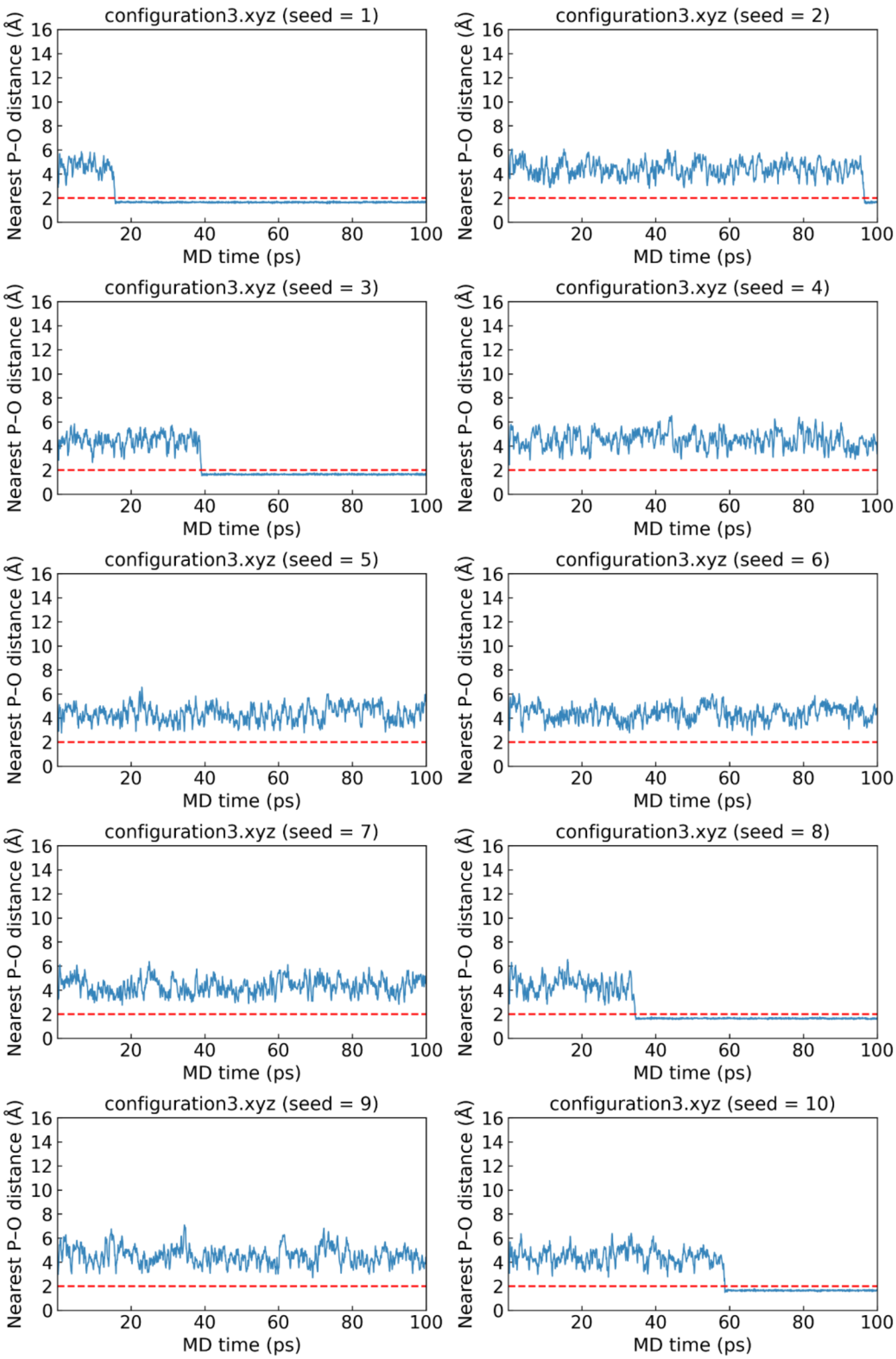

Figure S5 (continued).

- TIBPPh$_2$ (Compound **36**)

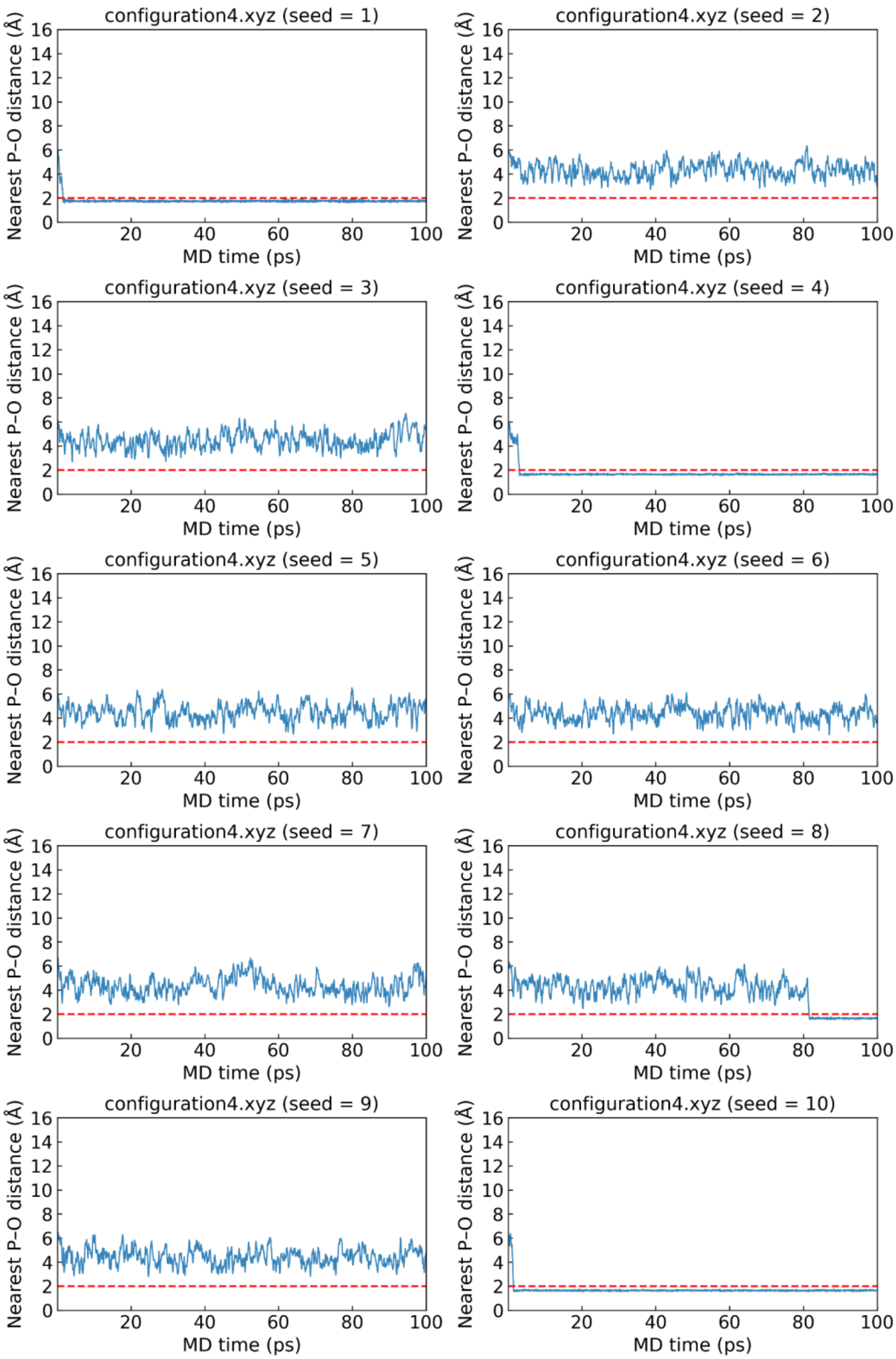

Figure S5 (continued).

S4. Cartesian coordinates for the potential energy diagrams

The Cartesian coordinates of the optimized initial states (IS), transition states (TS), and final states (FS) corresponding to the potential energy diagrams shown in Figure 4 (Steps 1–3) are provided.

Step 1 (IS)
Energy: -185.79787795784227 eV

Atom	X (Å)	Y (Å)	Z (Å)
C	11.89947963	5.25813410	9.62064946
C	12.42646397	6.54343284	9.47395447
C	11.66932312	7.66365831	9.82597088
C	10.37123488	7.51020875	10.33665878
C	9.85531664	6.21231375	10.49548701
C	10.60901671	5.09548637	10.13209518
P	9.29349129	8.90257786	10.85925401
C	10.42257293	10.34282255	10.70377128
C	10.43360985	11.22488385	9.61234461
C	11.31607435	12.30786495	9.58879003
C	12.20094585	12.52324641	10.64795609
C	12.19376784	11.65262606	11.74124046
C	11.30547345	10.57682677	11.77225970
C	8.19823964	9.11144394	9.39983132
C	8.34632372	8.40998694	8.19343568
C	7.45018270	8.61721759	7.14171764
C	6.39977072	9.52817735	7.27571837
C	6.24205187	10.22822093	8.47503489
C	7.12880644	10.01426619	9.53105321
H	12.49274614	4.38669760	9.34089457
H	13.43458023	6.67772846	9.07835583
H	12.09032577	8.66218142	9.70241834
H	8.85218751	6.07800948	10.90649614
H	10.19071767	4.09517187	10.25488804
H	9.75059337	11.06476556	8.77722432
H	11.31387493	12.98563150	8.73352909
H	12.89098112	13.36757332	10.62346456
H	12.87763513	11.81602057	12.57566498
H	11.29640563	9.90699214	12.63509432
H	9.16510105	7.69927482	8.07479796
H	7.57745240	8.06456621	6.20938621
H	5.70498935	9.68976998	6.45063609
H	5.42157747	10.93819569	8.59067140
H	6.99259725	10.55528868	10.47015611
O	8.17307888	10.61497024	21.62550995
O	8.26447377	9.38683945	21.50432800

Step 1 (TS)
Energy: -185.71341649732352 eV

Atom	X (Å)	Y (Å)	Z (Å)
C	12.00031173	5.20174319	9.69074391
C	12.59995206	6.46198399	9.75365038
C	11.83259216	7.59757090	10.01686322
C	10.44638439	7.47521153	10.21180903
C	9.85066229	6.20004648	10.17903242
C	10.62358889	5.07403335	9.90486813
P	9.42003099	8.90699876	10.64318999
C	10.48913537	10.36089965	10.62386827
C	10.52741769	11.24050966	9.52830331
C	11.36489432	12.35546500	9.55657342
C	12.17060089	12.60217128	10.67194441
C	12.13231635	11.73427673	11.76711161
C	11.29185592	10.62175450	11.74990887
C	8.19209681	9.12030672	9.33462027
C	8.16211164	8.30969248	8.18675521
C	7.17401386	8.50550981	7.22076726
C	6.21334714	9.50607080	7.38719350
C	6.23906237	10.31324022	8.52882535
C	7.21658053	10.12242197	9.50373747
H	12.60569673	4.31831149	9.48495943
H	13.67468794	6.56465622	9.59860970
H	12.30269021	8.57924463	10.06646889
H	8.78012244	6.09758472	10.36747448
H	10.15296827	4.09068321	9.87195563
H	9.89585361	11.04685225	8.66070752
H	11.38770598	13.03508543	8.70361020
H	12.82547422	13.47428685	10.68941109
H	12.75317269	11.92929969	12.64234463
H	11.25188237	9.95451373	12.61257299
H	8.91729608	7.53656491	8.04694907
H	7.15981794	7.87483551	6.33093837
H	5.44478563	9.65671246	6.62814838
H	5.48879226	11.09359486	8.66314807
H	7.23101110	10.74835647	10.39879000
O	8.16874958	10.64131938	12.63984204
O	8.26380103	9.36126364	12.51504234

Step 1 (FS)
Energy: -187.37511110083864 eV

Atom	X (Å)	Y (Å)	Z (Å)
C	11.78067072	5.37532947	9.59565691
C	12.42508170	6.60142358	9.75893384
C	11.69546152	7.73719321	10.12990900
C	10.31475413	7.65820076	10.33138522

C	9.67661999	6.41804740	10.17936250
C	10.40182842	5.28471620	9.80986097
P	9.29808122	9.11473725	10.87977523
C	10.49160879	10.47175607	10.71459016
C	10.84468290	10.98274408	9.46182669
C	11.81052231	11.98752842	9.36640752
C	12.42698711	12.47522347	10.52168731
C	12.07113609	11.96712065	11.77425136
C	11.09953108	10.96861107	11.87391847
C	8.06738075	9.19185924	9.53838435
C	8.36205872	8.68039181	8.26377072
C	7.44339991	8.81353471	7.22219701
C	6.21736774	9.44664882	7.44653223
C	5.91491751	9.94914655	8.71379377
C	6.83548403	9.82576885	9.75692467
H	12.35020574	4.48963872	9.31037923
H	13.50179953	6.68150311	9.60337521
H	12.21278379	8.68730595	10.26210987
H	8.60072878	6.34007194	10.34933139
H	9.88927534	4.32876581	9.69436578
H	10.36222265	10.60269761	8.56004183
H	12.08092691	12.38913475	8.38913192
H	13.18365376	13.25730169	10.44562957
H	12.54692625	12.35305794	12.67675826
H	10.79818401	10.57346267	12.84462603
H	9.30999017	8.17336796	8.08403000
H	7.68344316	8.41562933	6.23553024
H	5.49778175	9.54570215	6.63241892
H	4.95713282	10.43848860	8.89507784
H	6.60717728	10.21073994	10.74998407
O	8.31470766	9.86396714	12.10021077
O	9.07658655	8.52227001	12.38179124

Step 2 (IS)

Energy: -367.2350606833844 eV

Atom	X (Å)	Y (Å)	Z (Å)
C	12.83207173	6.30247901	9.28742191
C	12.60623841	6.08076095	10.64909099
C	12.36774220	4.78801446	11.11637186
C	12.34018351	3.70494851	10.22232088
C	12.56745025	3.93408949	8.85741239
C	12.81450630	5.22866463	8.39489439
P	11.93156035	2.02777670	10.80552511
C	12.43354550	2.15345993	12.59114550
C	11.54963584	1.89438535	13.64221548
C	11.98641148	1.96324393	14.97058043
C	13.31333312	2.28266100	15.25774211
C	14.20760487	2.52873186	14.21130475
C	13.77001224	2.45988192	12.88820660
C	10.13296206	1.83441318	10.94873145

C	9.58613349	0.55535044	10.78914008
C	8.21353606	0.36151798	10.96043577
C	7.38812007	1.44341391	11.27971270
C	7.93305971	2.72173377	11.42579977
C	9.30673513	2.91754286	11.26380383
H	13.02363568	7.31325661	8.92406794
H	12.62235804	6.91497930	11.35145017
H	12.20606725	4.62177916	12.18134084
H	12.55623499	3.08940718	8.16972568
H	12.99590703	5.39611972	7.33249135
H	10.51198081	1.63599600	13.43161290
H	11.28253056	1.76055704	15.77892588
H	13.65412586	2.33268979	16.29288256
H	15.24979591	2.77039400	14.42416053
H	14.47373814	2.64971459	12.07505817
H	10.24141098	-0.27492678	10.52357506
H	7.78709841	-0.63507214	10.83890248
H	6.31590259	1.29108086	11.41047248
H	7.28899413	3.56837696	11.66623199
H	9.73229969	3.91580719	11.37649702
O	12.09466354	1.23335711	9.26932155
O	12.78918978	0.66460584	10.55540333
C	17.96943576	-4.03271109	3.70403775
C	16.79977048	-4.41846587	3.04499130
C	15.59474505	-4.52351363	3.74425178
C	15.54441689	-4.23740890	5.11718912
C	16.72319599	-3.83467470	5.76846721
C	17.92794749	-3.74174677	5.07038083
P	14.01772041	-4.31519900	6.13523248
C	12.72647258	-4.63860611	4.86965872
C	12.19191357	-5.90592792	4.59097991
C	11.19554205	-6.05443757	3.62286266
C	10.72303659	-4.94529392	2.91728089
C	11.24680473	-3.67876809	3.19081080
C	12.23386265	-3.52628932	4.16543537
C	14.17987888	-5.95406550	6.94819759
C	15.20167413	-6.87562255	6.67252241
C	15.26006955	-8.09132523	7.35868525
C	14.29996949	-8.40677107	8.32292746
C	13.28092614	-7.49335608	8.60694335
C	13.22740581	-6.27347174	7.93148914
H	18.90870546	-3.95590456	3.15497185
H	16.82416680	-4.64406594	1.97757341
H	14.68922157	-4.83019160	3.21930532
H	16.69480411	-3.59413797	6.83361480
H	18.83556344	-3.43501042	5.59267196
H	12.55571987	-6.78028696	5.13206996
H	10.78883921	-7.04586997	3.41665963
H	9.94774206	-5.06656140	2.15979142
H	10.88048585	-2.80585793	2.64813795
H	12.63043640	-2.53208095	4.38333670

H	15.95465963	-6.64303745	5.91859194
H	16.06053770	-8.79821656	7.13408321
H	14.34708689	-9.35869242	8.85322388
H	12.52930363	-7.72899030	9.36204170
H	12.43581185	-5.55859437	8.16717904

Step2 (TS)

Energy: -366.7238886146596 eV

Atom	X (Å)	Y (Å)	Z (Å)
C	13.42992319	4.44932563	7.94133413
C	13.24966782	4.56548536	9.32265620
C	13.01598081	3.42606654	10.09290247
C	12.96023797	2.16173318	9.47898539
C	13.14244164	2.04593637	8.09002970
C	13.37805663	3.19296739	7.33023079
P	12.49789114	0.72451164	10.46578404
C	12.85909732	1.11431572	12.19143729
C	11.86584259	1.04460597	13.17890713
C	12.18942280	1.29579234	14.51419768
C	13.50243704	1.61304708	14.86990246
C	14.49761834	1.68065361	13.88924554
C	14.17992760	1.43321962	12.55476260
C	10.72738977	0.46003417	10.31518903
C	10.21294699	-0.84262266	10.24351824
C	8.83047054	-1.03328520	10.20239414
C	7.96653798	0.06601776	10.22622770
C	8.48157270	1.36480712	10.28015487
C	9.86147263	1.56431059	10.32358727
H	13.60858864	5.34226576	7.34044346
H	13.29078343	5.54394117	9.80275647
H	12.87643375	3.51738606	11.17079586
H	13.09617274	1.05144367	7.64415665
H	13.51864810	3.10650328	6.25200495
H	10.84380678	0.78668809	12.90097808
H	11.41314005	1.23606089	15.27782469
H	13.75409250	1.80093852	15.91445443
H	15.52527216	1.91898326	14.16555023
H	14.95547294	1.47469946	11.78824788
H	10.90387587	-1.68381135	10.19115665
H	8.42408867	-2.04378808	10.14467007
H	6.88707921	-0.08799706	10.19693945
H	7.80900336	2.22334222	10.28710694
H	10.26588841	2.57725746	10.36013483
O	12.97184764	-0.82620583	8.81540738
O	13.34403432	-0.59238442	10.27126150
C	17.45660936	-2.61562935	4.67766605
C	16.33875127	-3.03633652	3.95448214
C	15.08387404	-3.09775541	4.56503520
C	14.93521418	-2.73405303	5.91264188
C	16.05926977	-2.28233447	6.62700537

C	17.31188309	-2.23502477	6.01547739
P	13.35487414	-2.83629032	6.83227438
C	12.16117657	-3.32973165	5.53537681
C	11.72743519	-4.64794089	5.32367031
C	10.80255751	-4.92924203	4.31580980
C	10.29935728	-3.90543372	3.51014235
C	10.71675000	-2.58852910	3.72391884
C	11.63341180	-2.30157136	4.73481302
C	13.57915664	-4.39352357	7.77119221
C	14.62798719	-5.29814281	7.53561066
C	14.71566215	-6.48429590	8.26702315
C	13.75949615	-6.78682621	9.23895355
C	12.72056400	-5.88592786	9.48936572
C	12.63612547	-4.69423111	8.76843441
H	18.43725855	-2.58368799	4.20119500
H	16.44331047	-3.33118498	2.90902076
H	14.22108408	-3.44675684	3.99626237
H	15.94456202	-1.97567177	7.66877163
H	18.18100942	-1.89967819	6.58391199
H	12.11760964	-5.45569974	5.94397391
H	10.47655402	-5.95855087	4.15785305
H	9.58117602	-4.13164482	2.72120877
H	10.32109306	-1.78138235	3.10546947
H	11.94678770	-1.27020638	4.91088605
H	15.37463625	-5.07857667	6.77166424
H	15.53303729	-7.17964847	8.06845675
H	13.82484286	-7.72003508	9.79989379
H	11.97456338	-6.11278029	10.25282069
H	11.83332780	-3.98464080	8.97693441

Step 2 (FS)

Energy: -371.6103906359719 eV

Atom	X (Å)	Y (Å)	Z (Å)
C	13.26409667	14.69386315	8.10363789
C	12.97418196	14.78585315	9.46724534
C	12.73440084	13.62921755	10.21248182
C	12.78088588	12.37263097	9.59170476
C	13.08457279	12.28376197	8.22498368
C	13.32391707	13.44225733	7.48454417
P	12.44959330	10.81038090	10.46412353
C	12.84118136	11.12323942	12.21362038
C	11.92468770	11.66703715	13.12522614
C	12.30894950	11.89992175	14.44760621
C	13.60502217	11.58872525	14.86633244
C	14.51712667	11.03502107	13.96366453
C	14.13789034	10.80097779	12.64109329
C	10.64657720	10.57465948	10.39144232
C	10.17520610	9.25564130	10.46765079
C	8.80331151	9.00035255	10.44265233
C	7.89582804	10.05820997	10.33939411

C	8.36171796	11.37245940	10.25114568
C	9.73360870	11.63279135	10.27488181
H	13.45201497	15.59904804	7.52449918
H	12.94123796	15.76110695	9.95501561
H	12.52659769	13.70413164	11.28119137
H	13.14828492	11.29821682	7.76036628
H	13.56264631	13.36922057	6.42255082
H	10.90561760	11.89366594	12.80738469
H	11.59126142	12.31873885	15.15447196
H	13.90160891	11.77012506	15.90037512
H	15.52526676	10.77995429	14.29338096
H	14.83209245	10.34918492	11.93030728
H	10.89681168	8.43890057	10.52670268
H	8.44036848	7.97308072	10.49853384
H	6.82366151	9.85785608	10.31912045
H	7.65526782	12.19828284	10.15608902
H	10.09395763	12.65907733	10.18623929
O	13.18640667	-1.85743171	7.55554397
O	13.20964134	9.65511824	9.89615990
C	17.57773516	-2.61671001	4.59900293
C	16.58293095	-3.38798722	3.99281030
C	15.33751573	-3.53430317	4.60757525
C	15.08444461	-2.90985990	5.83749964
C	16.08186555	-2.12653937	6.43703127
C	17.32466355	-1.98260396	5.81848535
P	13.50785473	-3.06387137	6.73332265
C	12.24898064	-3.41265354	5.46626947
C	11.95135746	-4.70437404	5.00855133
C	10.98867271	-4.88861446	4.01362147
C	10.31788821	-3.78789341	3.47475186
C	10.60285041	-2.50029911	3.93764443
C	11.56430543	-2.31123480	4.93139506
C	13.64570715	-4.58192907	7.72751304
C	14.48829336	-5.65138666	7.39154364
C	14.51929423	-6.79493922	8.19277794
C	13.71433383	-6.87505890	9.33185507
C	12.88275625	-5.80595254	9.67661613
C	12.84806726	-4.66137415	8.87884261
H	18.54898681	-2.50291145	4.11548226
H	16.77382834	-3.87183574	3.03388300
H	14.55686774	-4.12167742	4.12130525
H	15.86207243	-1.62025994	7.37863556
H	18.09676036	-1.36970468	6.28573106
H	12.45953634	-5.56832333	5.44011652
H	10.75700299	-5.89568328	3.66389625
H	9.56559303	-3.93501420	2.69858103
H	10.06999495	-1.64181466	3.52631273
H	11.78243482	-1.31413384	5.31797153
H	15.13224580	-5.58545022	6.51301188
H	15.17957675	-7.62263916	7.93002704
H	13.74167139	-7.76910432	9.95638907

H	12.26346976	-5.86286758	10.57292319
H	12.21958901	-3.81023688	9.14683342

Step 3 (IS)
Energy: -187.37511425973616 eV

Atom	X (Å)	Y (Å)	Z (Å)
C	11.51184135	9.30549747	12.78568460
C	10.83348453	9.99864214	11.77838505
C	9.70411578	9.43614459	11.18258228
C	9.23109950	8.17947050	11.58912802
C	9.92332082	7.48674349	12.58619976
C	11.05646902	8.04936966	13.18567428
P	7.75507165	7.46455432	10.71382473
C	7.41688251	5.99161815	11.71856576
C	7.81255035	4.74296899	11.22373842
C	7.62619679	3.59782532	12.00149621
C	7.03708489	3.69791157	13.26507484
C	6.63113913	4.94289998	13.75267167
C	6.82410637	6.09135541	12.98104068
C	6.50939450	8.75251451	11.04493688
C	5.39220917	8.87538353	10.20590717
C	4.39890059	9.81443842	10.49282435
C	4.50965550	10.63284131	11.61883818
C	5.62078491	10.51552300	12.45903275
C	6.61955181	9.58489794	12.17101752
H	12.39726581	9.74175754	13.25030751
H	11.18639305	10.97753211	11.45116546
H	9.18095647	9.98051730	10.39362026
H	9.58315065	6.50068787	12.90213960
H	11.58343310	7.49596615	13.96412493
H	8.25506970	4.68003662	10.22903266
H	7.93620927	2.62497300	11.61770001
H	6.88933002	2.80229562	13.87008290
H	6.16361882	5.02133872	14.73488995
H	6.50613835	7.06344813	13.36098685
H	5.31692746	8.23544557	9.32768302
H	3.53687229	9.90652130	9.83095044
H	3.73181716	11.36451978	11.84250117
H	5.71474752	11.15474103	13.33770053
H	7.48929665	9.50933578	12.82352342
O	7.04469258	6.86885479	9.24470870
O	8.57072881	7.22227972	9.32379253

Step 3 (TS)
Energy: -186.7805191557403 eV

Atom	X (Å)	Y (Å)	Z (Å)
C	11.46663169	9.21419307	12.75167898
C	10.96940473	9.75884696	11.56507255

C	9.84321287	9.20720278	10.95203560
C	9.18744508	8.11280127	11.53279481
C	9.69232952	7.56472585	12.71410628
C	10.82974954	8.11237786	13.32056575
P	7.64204357	7.47030655	10.60276998
C	7.41727327	5.94314056	11.63174277
C	8.24163183	4.83137712	11.39103413
C	8.11497074	3.70080752	12.19105811
C	7.16632884	3.66361471	13.22057439
C	6.34133023	4.76740603	13.44710119
C	6.46097923	5.91108384	12.65572344
C	6.47313907	8.79342331	11.09254145
C	5.28594886	8.92408178	10.35287634
C	4.35482274	9.90545961	10.69257989
C	4.58927462	10.75749725	11.77408087
C	5.76722582	10.63196252	12.51394288
C	6.70700041	9.65977288	12.17099847
H	12.34963027	9.64436446	13.22636675
H	11.46543579	10.61573809	11.10641702
H	9.47115488	9.62280015	10.01419803
H	9.20693280	6.70421496	13.17484487
H	11.21398344	7.66962322	14.24090215
H	8.96977407	4.86402155	10.57911334
H	8.75843145	2.83795911	12.01295565
H	7.06953108	2.77290946	13.84233111
H	5.59974849	4.74098251	14.24728944
H	5.81843370	6.77449680	12.83249447
H	5.10443725	8.24152330	9.52256203
H	3.43534201	10.00042412	10.11288413
H	3.85453175	11.51888743	12.04089369
H	5.95732992	11.29504847	13.35905531
H	7.62677809	9.57264200	12.74875026
O	6.63538658	6.58433334	9.61554569
O	8.35174430	7.58433049	9.19352598

Step 3 (FS)

Energy: -191.10151288321987 eV

Atom	X (Å)	Y (Å)	Z (Å)
C	11.55448660	8.47572945	12.85901269
C	11.60617148	8.82573090	11.50755408
C	10.44841879	8.77926938	10.72853264
C	9.23176972	8.38028445	11.30185083
C	9.18379211	8.02989753	12.66192230
C	10.34358448	8.07960275	13.43524276
P	7.79648886	8.29932274	10.20072035
C	7.38193748	5.83955387	11.18929138
C	8.47904817	4.98978364	11.34970360
C	8.49189696	4.07873930	12.40638444
C	7.41900739	4.01878716	13.30079662
C	6.32536268	4.87038717	13.12669341

C	6.29904983	5.78404220	12.06966120
C	6.40148976	9.09534571	11.03754420
C	5.10067135	8.81728586	10.58356337
C	4.00425433	9.46211104	11.15506415
C	4.19523449	10.39629779	12.17780214
C	5.48622877	10.68983731	12.62122077
C	6.58681400	10.04396039	12.05400059
H	12.45971057	8.51098809	13.46687130
H	12.55111773	9.13533834	11.05922813
H	10.46582019	9.04937746	9.67136120
H	8.24326159	7.71498703	13.11618017
H	10.30262430	7.80528653	14.49002094
H	9.31060641	5.05854791	10.64849924
H	9.34829251	3.41558689	12.53380511
H	7.43407849	3.30866622	14.12792450
H	5.48234599	4.82655801	13.81749746
H	5.45151274	6.45288033	11.92546133
H	4.95339982	8.09716000	9.77710177
H	2.99747695	9.23961209	10.79880186
H	3.33707447	10.90169564	12.62268784
H	5.63964437	11.42638482	13.41102318
H	7.59329445	10.27992939	12.40075044
O	7.37498911	6.72022374	10.11213860
O	8.05572135	8.81102365	8.83616966

S5. Comparison of Calculated Activation Energies with Experimental Kinetic Data for Representative Phosphines

To demonstrate that differences in the Step 1 activation energy between phosphines are too small to compare reliably using DFT or MLIP calculations, the Step 1 activation energy was calculated for PEt_3 (Compound **3**) and compared with that of PPh_3 (Compound **24**). These two phosphines were selected because PEt_3 is known to be far less stable than PPh_3 in air (main text ref 53), making them a suitable pair to test whether static barrier heights can discriminate between phosphines of contrasting oxidation resistance.

S5.1. Step 1 Activation Energies and Mean Survival Times for PEt_3 and PPh_3

The Step 1 activation energies and τ for PPh_3 and PEt_3 are summarized in Table S3. Although PEt_3 is far less stable than PPh_3 in air, its Step 1 activation energy differs from that of PPh_3 by only 0.38 kcal/mol, which is too small to compare reliably using DFT or MLIP calculations. In contrast, the corresponding τ are 6.7 and 51.0 ps, respectively, and their confidence intervals do not overlap (4.2–11.7 ps versus 36.7–73.7 ps), indicating that this difference is statistically significant. This demonstrates that differences in oxidation resistance between phosphines cannot be reliably resolved from barrier heights alone, and that the differences captured by τ arise from factors such as the probability that oxygen molecules reach a reactive conformation.

Table S3. Step 1 activation energies and mean survival times for PPh_3 and PEt_3.

Compound	E_{a1} (kcal/mol)	τ (ps)	τ_{low} (ps)	τ_{up} (ps)
3	1.52	6.7	4.2	11.7
24	1.90	51.0	36.7	73.7

S5.2. Step 2 and Step 3 Activation Energies for Tris(*o*-methoxyphenyl)phosphine

To examine whether static activation energies can quantitatively reproduce experimentally observed branching between competing oxidation pathways, the Step 2 and Step 3 activation energies were calculated for tris(*o*-methoxyphenyl)phosphine using the same computational protocol as for PPh_3 (Section 3.1.1). This phosphine was selected because a rate ratio of the oxide-forming pathway (Step 2 analog) to the phosphinate-forming pathway (Step 3 analog) of approximately 25 has been reported experimentally under photosensitized conditions in solution (main text ref 52).

The calculated activation energies for the Step 2 and Step 3 analogs were 4.87 and 11.2 kcal/mol, respectively (Table S4). Assuming comparable pre-exponential factors for the two pathways, this barrier difference corresponds to a predicted rate ratio on the order of 10^4, substantially larger than the experimentally reported value of 25. This discrepancy is attributed to differences between the two systems rather than to a deficiency of the present calculations. The literature study was conducted under photosensitized conditions in solution, whereas the present calculations are performed in the gas phase. In addition, Step 2 is a bimolecular process and Step 3 is unimolecular, so their activation entropies, and therefore their pre-exponential factors, are expected to differ substantially, an effect not captured by comparison of electronic activation energies alone. This result further illustrates that static activation energies alone cannot reliably reproduce experimentally observed branching ratios between competing reaction pathways.

Table S4. Step 2 and Step 3 activation energies for tris(*o*-methoxyphenyl)phosphine.

Step	E_a (kcal/mol)
Step 2	4.87
Step 3	11.2

S5.3 Cartesian Coordinates

S5.3.1 Cartesian Coordinates of PEt_3 (Step 1)

Step 1 (IS)
Energy: -94.81853582309758 eV

Atom	X (Å)	Y (Å)	Z (Å)
C	0.19281651	-3.12731711	3.53573221
C	-0.67209176	-2.56839159	2.40425091

P	0.28687858	-2.27013676	0.83931581
C	-1.02678214	-1.40387121	-0.15136893
C	-0.50375990	-0.84800407	-1.47730803
C	1.38322741	-0.84659650	1.33606221
C	0.73810039	0.37956702	1.98239687
H	0.94728841	-2.39763164	3.86331238
H	-0.41799078	-3.38413880	4.41309279
H	0.72626851	-4.03520660	3.21885415
H	-1.45856070	-3.29214578	2.13675439
H	-1.18787007	-1.64843004	2.72544807
H	-1.80409312	-2.16166145	-0.33962667
H	-1.50194864	-0.60780860	0.44532283
H	0.23108777	-0.04623961	-1.31565298
H	-1.32034059	-0.42780336	-2.08182086
H	-0.01270703	-1.63174815	-2.07210254
H	2.14569282	-1.27890987	2.00337059
H	1.92886010	-0.56004253	0.42297473
H	1.49590655	1.13890748	2.22693382
H	0.00432294	0.85309462	1.31515798
H	0.21966575	0.12237418	2.91673521
O	2.82185719	-10.20672199	-3.44822348
O	3.92987353	-9.66204678	-3.36469940

Step 1 (TS)
Energy: -94.75269703992707 eV

Atom	X (Å)	Y (Å)	Z (Å)
C	0.39710730	-3.05527601	3.54184799
C	-0.52068411	-2.65571909	2.38413570
P	0.38831640	-2.16723469	0.87738580
C	-0.87554496	-1.46798452	-0.24070915
C	-0.28928532	-0.78865295	-1.48059325
C	1.42684084	-0.71938243	1.39066939
C	0.63216123	0.42630897	2.01889828
H	0.97482959	-2.19973711	3.92022177
H	-0.19122669	-3.45065638	4.38089682
H	1.10762582	-3.83343894	3.23202147
H	-1.13144526	-3.51128942	2.04216622
H	-1.22040647	-1.85783657	2.67690061
H	-1.48108935	-2.35046379	-0.51666035
H	-1.53508217	-0.78787529	0.32011307
H	0.29255281	0.10753467	-1.22129560
H	-1.09264050	-0.47223174	-2.15972140
H	0.36827381	-1.47235114	-2.03462102
H	2.18947714	-1.10210558	2.08444869
H	1.97233752	-0.38457766	0.49627592
H	1.30641391	1.24848201	2.29930191
H	-0.11466664	0.82944601	1.32256533
H	0.10706001	0.10142344	2.92647278
O	-0.80991688	-4.52589176	-0.02342940
O	0.43025391	-4.07274457	0.01100656

Step 1 (FS)
Energy: -96.64389563169449 eV

Atom	X (Å)	Y (Å)	Z (Å)
C	0.24962674	-3.40402061	3.44446821
C	-0.62189476	-2.52109875	2.54870215
P	0.17606814	-2.13241374	0.96010000
C	-1.02918963	-1.32996741	-0.14113159
C	-0.44840602	-0.98667371	-1.51366636
C	1.28764094	-0.71840515	1.42671564
C	0.64381470	0.58250038	1.89968514
H	1.17096629	-2.89015400	3.75167477
H	-0.29440683	-3.68191998	4.35776420
H	0.52983506	-4.32369822	2.91503603
H	-1.55811433	-3.03281901	2.28788732
H	-0.87502984	-1.57191864	3.04435355
H	-1.86204739	-2.03950158	-0.22974994
H	-1.39809147	-0.42973801	0.37327518
H	0.36054397	-0.24639954	-1.43989399
H	-1.22452727	-0.56428372	-2.16673256
H	-0.04595464	-1.88571686	-1.99803384
H	1.95724775	-1.11930611	2.20416964
H	1.92685754	-0.53441866	0.54954537
H	1.41056513	1.30098054	2.22393259
H	0.06547100	1.06911066	1.10164095
H	-0.03149593	0.42839116	2.75397959
O	0.00154956	-3.73685952	0.27779160
O	1.39130057	-3.05436634	0.38223109

S5.3.2 Cartesian Coordinates of Tris(*o*-methoxyphenyl)phosphine (Step 2 and Step 3)

Step 2 (IS): Tris(*o*-methoxyphenyl)phosphine
Energy: -470.82228656040536 eV

Atom	X (Å)	Y (Å)	Z (Å)
C	13.09573406	13.30686909	18.22006301
C	11.98260133	13.43125214	19.05522327
C	11.28014304	12.28827637	19.45459782
C	11.69651274	11.01545382	19.01497532
C	12.80974082	10.91452685	18.17455677
C	13.51310940	12.05315130	17.77361123
P	10.98303985	9.46269667	19.63974927
C	11.41367773	9.69705199	21.45311536
C	10.60272061	10.19073127	22.50559053
C	11.14037360	10.36283332	23.79336939
C	12.47505404	10.06069917	24.05482313
C	13.29321182	9.58540226	23.03153108
C	12.75367633	9.41296177	21.75645592
C	9.18162811	9.28700508	19.62689676

C	8.60886597	8.36455964	20.50660235
C	7.23496099	8.12067140	20.50768554
C	6.42346849	8.81330393	19.60832842
C	6.97637238	9.71795635	18.69980850
C	8.35924002	9.94619163	18.68944817
H	13.63424475	14.20556864	17.91554520
H	11.66326637	14.41905295	19.38343512
H	13.10191351	9.92210377	17.82976031
H	14.37531788	11.95941159	17.11350551
H	10.51109454	10.73639534	24.59905062
H	12.86935985	10.20283377	25.06208722
H	14.34020762	9.34691832	23.21748370
H	13.39417458	9.03743364	20.95627458
H	9.25728528	7.84300953	21.21285126
H	6.80444787	7.40218702	21.20471012
H	5.34576369	8.64299348	19.59844233
H	6.32831489	10.23385267	17.99330113
O	11.18296091	8.57406597	18.13383110
O	11.89481218	8.12372340	19.45541955
C	17.58392101	-1.30037819	5.53071226
C	16.87505021	-2.08968259	4.62000219
C	15.60113856	-2.57810597	4.93979992
C	15.01892784	-2.26525890	6.19298037
C	15.74878827	-1.45704961	7.07457288
C	17.02530052	-0.97993130	6.76544517
P	13.36855987	-2.74887242	6.84852826
C	12.34634359	-3.19614075	5.38491818
C	12.16101053	-4.46786469	4.78847990
C	11.31556468	-4.60254026	3.67908592
C	10.65628880	-3.49002802	3.14749328
C	10.82371674	-2.23332103	3.72362784
C	11.65645498	-2.10591742	4.83853471
C	13.62864424	-4.30980763	7.78820444
C	14.69576117	-5.23646519	7.68869257
C	14.72016090	-6.36545113	8.51842311
C	13.69483523	-6.59248046	9.44152228
C	12.64019954	-5.69020302	9.55528630
C	12.62719494	-4.55820336	8.73606526
H	18.57600545	-0.93591769	5.26052439
H	17.32763970	-2.32483006	3.65785681
H	15.29218796	-1.20210591	8.03444054
H	17.56812840	-0.35870091	7.47792985
H	11.16725538	-5.57732792	3.21685747
H	10.00709673	-3.61954371	2.28040254
H	10.30721302	-1.36235316	3.32007695
H	11.78667661	-1.12810760	5.30943469
H	15.53824156	-7.08079432	8.44845391
H	13.73369418	-7.48114890	10.07305063
H	11.84095890	-5.85592480	10.27776220
H	11.81292498	-3.83428217	8.82397674
O	12.84425965	-5.51744496	5.32627240

C	12.69700781	-6.79937497	4.73102828
H	13.04426247	-6.80194929	3.68388121
H	11.65115907	-7.14668191	4.76782494
H	13.32210881	-7.47505960	5.32558464
O	14.88259408	-3.37138766	4.09592046
C	15.45298905	-3.71996236	2.84193627
H	16.38683014	-4.29384651	2.96723559
H	15.65446579	-2.82830322	2.22540478
H	14.70476532	-4.34353192	2.33982421
O	15.65809253	-4.98551003	6.75669683
C	16.72759955	-5.91103398	6.61946390
H	16.36396045	-6.90888399	6.32085322
H	17.30952525	-6.00039447	7.55173692
H	17.37082565	-5.50478433	5.83078433
O	10.16961027	12.31952231	20.23942797
C	9.76968729	13.56237882	20.80315793
H	9.48480970	14.28641113	20.02216218
H	10.56826348	13.99364400	21.42871996
H	8.89656027	13.33745586	21.42508312
O	9.30176674	10.48958521	22.23036869
C	8.44254072	10.86429148	23.30012815
H	8.75084059	11.82111335	23.75455595
H	8.40449058	10.08760200	24.08144544
H	7.44875606	10.97572476	22.85203277
O	8.97707402	10.77895879	17.80996932
C	8.18019489	11.46341281	16.84785607
H	7.44663035	12.12721743	17.33408170
H	7.65413478	10.75612650	16.18671623
H	8.88078413	12.06282116	16.25682838

Step 2 (TS) : Tris(*o*-methoxyphenyl)phosphine
Energy: -470.6110377719635 eV

Atom	X (Å)	Y (Å)	Z (Å)
C	14.42771152	4.16340590	8.10840625
C	13.63655676	4.45977125	9.22186765
C	13.02378581	3.42811144	9.94139936
C	13.21096643	2.08293906	9.53603344
C	14.00184436	1.81022857	8.41020839
C	14.61202901	2.84339598	7.69720677
P	12.51542748	0.69745075	10.46588515
C	12.79178139	1.06276074	12.22556042
C	11.81351150	1.47809213	13.15611941
C	12.17269439	1.67950228	14.49702843
C	13.49111984	1.48684168	14.91320106
C	14.47156002	1.09592342	14.00117377
C	14.11348808	0.88767709	12.67082638
C	10.78681083	0.30598121	10.15515046
C	10.35978475	-0.94921571	10.61198805
C	9.03462519	-1.35453792	10.47060761
C	8.12148787	-0.49504877	9.85650118

C	8.52810832	0.75179079	9.38010582
C	9.86183047	1.16275476	9.52227541
H	14.89897260	4.98224971	7.56203434
H	13.50177920	5.49683680	9.52491802
H	14.10234730	0.76513758	8.11032665
H	15.22525922	2.61337555	6.82585917
H	11.42430453	1.98896468	15.22401906
H	13.74673517	1.64531321	15.96166679
H	15.50224220	0.94319657	14.31986863
H	14.86366115	0.55917056	11.94993421
H	11.09836131	-1.61581846	11.05994881
H	8.72013152	-2.33527800	10.82644073
H	7.07980357	-0.79599940	9.73450953
H	7.80269727	1.40112428	8.89303037
O	13.02882587	-0.86792468	8.92646047
O	13.39807281	-0.61586782	10.38634287
C	17.49979609	-1.14525022	5.64670950
C	16.83871899	-1.93006313	4.69719906
C	15.58960436	-2.49474345	4.98752838
C	14.98381594	-2.25658809	6.24706495
C	15.66715926	-1.45802711	7.17646305
C	16.91921380	-0.90969929	6.89170653
P	13.33708356	-2.81333980	6.83029318
C	12.40289942	-3.27577284	5.32645452
C	12.13603723	-4.56102857	4.79411844
C	11.36934689	-4.68856968	3.62736532
C	10.86310656	-3.55766817	2.98175606
C	11.10180448	-2.28663358	3.50189729
C	11.85327558	-2.16316524	4.67094521
C	13.64376292	-4.32725831	7.81189543
C	14.66687798	-5.29188571	7.62352881
C	14.75592943	-6.39437107	8.48320679
C	13.83886815	-6.55388219	9.52640453
C	12.83707668	-5.60818274	9.73332216
C	12.75747236	-4.50237545	8.88296017
H	18.47256994	-0.71766160	5.39948815
H	17.30833684	-2.10298782	3.73028884
H	15.16727035	-1.25973519	8.12961895
H	17.42774582	-0.29256629	7.63300319
H	11.16232059	-5.67339521	3.21221143
H	10.27241978	-3.68173210	2.07327048
H	10.69767700	-1.40014195	3.01307821
H	12.03456929	-1.17440758	5.10006645
H	15.53851134	-7.13919376	8.34470805
H	13.92312276	-7.42338315	10.18011676
H	12.12989184	-5.72139777	10.55540775
H	12.00152514	-3.73149198	9.04882897
O	12.66482265	-5.63359968	5.44715246
C	12.43376902	-6.93392979	4.92012754
H	12.85879943	-7.04115379	3.90801923
H	11.35826090	-7.17405436	4.89075714

H	12.93903760	-7.62365801	5.60540637
O	14.92060903	-3.29630642	4.11015670
C	15.49528137	-3.53146284	2.83157803
H	16.46367335	-4.05376432	2.91172308
H	15.63466739	-2.59212626	2.27148280
H	14.77930789	-4.16705852	2.29879020
O	15.51943193	-5.10028666	6.57913663
C	16.53664905	-6.06672501	6.34875792
H	16.10882900	-7.05955151	6.12961774
H	17.22253406	-6.14757838	7.20797064
H	17.09337111	-5.70869413	5.47565884
O	12.22360441	3.64534965	11.02162473
C	11.98039818	4.98516919	11.43471146
H	11.48394688	5.56613136	10.63996878
H	12.91216148	5.49265834	11.73234064
H	11.31462383	4.91109857	12.30146571
O	10.54905566	1.67945804	12.68952500
C	9.53415894	2.03936894	13.62083851
H	9.74638129	3.01386628	14.09171735
H	9.41753683	1.27402947	14.40498415
H	8.60902930	2.10757729	13.03870138
O	10.32103141	2.36301184	9.07599793
C	9.40938744	3.24648128	8.43012932
H	8.58793610	3.54144135	9.10354988
H	8.99024886	2.79414590	7.51669602
H	9.99651613	4.13117056	8.15981471

Step 2 (FS) : Tris(*o*-methoxyphenyl)phosphine
Energy: -475.4094195354462 eV

Atom	X (Å)	Y (Å)	Z (Å)
C	13.16662040	10.14645928	14.38544748
C	12.59724467	10.51222760	15.60743205
C	12.12944645	9.52828321	16.48921863
C	12.23427356	8.16362777	16.14005641
C	12.82724762	7.82466237	14.91639436
C	13.28856271	8.80296628	14.03395929
P	11.69275475	6.72521711	17.12228990
C	11.79397582	7.12744137	18.89857524
C	10.77923411	7.67908170	19.71164866
C	11.03007826	7.92896133	21.06782410
C	12.28048049	7.63443153	21.61633792
C	13.28688110	7.07781183	20.82850263
C	13.03250460	6.82379285	19.47970082
C	9.97181800	6.30305693	16.68936820
C	9.67116422	4.94081844	16.82263721
C	8.39606943	4.44506233	16.54530900
C	7.40431071	5.32486064	16.11434270
C	7.68172380	6.68442379	15.95353334
C	8.96119829	7.18078542	16.23837647
H	13.52388501	10.92668959	13.71160838

H	12.52425030	11.56668525	15.86845278
H	12.93598704	6.76327025	14.68469482
H	13.74607571	8.51743988	13.08677864
H	10.25359859	8.35052821	21.70396428
H	12.45768953	7.83780138	22.67354125
H	14.25779231	6.83496383	21.26011012
H	13.78899887	6.36006992	18.84353300
H	10.47789443	4.27349092	17.13235754
H	8.18337194	3.38187218	16.65651152
H	6.40189432	4.95734970	15.88948955
H	6.89661906	7.35185184	15.60213806
O	12.80899741	-1.75981196	7.63871905
O	12.58203691	5.55597346	16.82511294
C	17.56442259	-1.19739941	5.53422325
C	16.93507021	-2.07977500	4.65300684
C	15.67934418	-2.61575572	4.96990236
C	15.04945693	-2.26175037	6.18360457
C	15.69502688	-1.35955548	7.03983501
C	16.94845565	-0.82933594	6.72942307
P	13.43030412	-2.83404361	6.79828487
C	12.33857036	-3.20541065	5.38501400
C	12.18463996	-4.44366462	4.72308187
C	11.29619740	-4.55277548	3.64450261
C	10.56390681	-3.44051193	3.22285159
C	10.69642202	-2.21562192	3.87513204
C	11.57707669	-2.11110956	4.95313624
C	13.65597881	-4.33746294	7.80629559
C	14.66820578	-5.31384537	7.67515851
C	14.69150269	-6.41572880	8.54113577
C	13.71643127	-6.55050127	9.53219305
C	12.71921790	-5.58784360	9.68112425
C	12.70199478	-4.48803454	8.82172510
H	18.54250751	-0.79181553	5.27095236
H	17.42738871	-2.34308105	3.71832336
H	15.17225910	-1.06738053	7.95274182
H	17.43331536	-0.12957912	7.41004407
H	11.16706429	-5.50358535	3.13011021
H	9.87849868	-3.54456252	2.38033422
H	10.11406131	-1.35165708	3.55509678
H	11.68068803	-1.17455502	5.50466204
H	15.46961678	-7.17185810	8.45103859
H	13.74992147	-7.41597761	10.19571161
H	11.96708776	-5.68572179	10.46383852
H	11.95344775	-3.70098281	8.93231036
O	12.92702823	-5.49018222	5.18155715
C	12.79757461	-6.74908771	4.53476359
H	13.09957235	-6.69225705	3.47533497
H	11.76666210	-7.13404113	4.59865510
H	13.47046672	-7.42804948	5.07031160
O	15.01428654	-3.48046579	4.15356693
C	15.62120828	-3.85722325	2.92491186

H	16.57813548	-4.38045411	3.09025990
H	15.79157373	-2.98540226	2.27223643
H	14.91200221	-4.53772522	2.44064325
O	15.58959588	-5.12554743	6.68920359
C	16.62373904	-6.08802504	6.53349706
H	16.21652058	-7.08245273	6.28425995
H	17.24493507	-6.16657891	7.44072594
H	17.24113956	-5.72775251	5.70304625
O	11.56531659	9.81994280	17.69470078
C	11.44316245	11.18392888	18.07493145
H	10.80034218	11.74300960	17.37417817
H	12.42686121	11.67730944	18.13826588
H	10.97928549	11.17434044	19.06757688
O	9.58217954	7.94450280	19.11741075
C	8.53957743	8.49863655	19.90851082
H	8.82134433	9.48422271	20.31600525
H	8.25996133	7.82879556	20.73807768
H	7.68436590	8.61368481	19.23323980
O	9.29477893	8.49472209	16.10157315
C	8.30090334	9.40408296	15.64862870
H	7.44850197	9.45238125	16.34711909
H	7.93334424	9.13644289	14.64447456
H	8.79253966	10.38231563	15.60435724

Step 3 (IS) : Tris(*o*-methoxyphenyl)phosphine
Energy: -239.17498307803206 eV

Atom	X (Å)	Y (Å)	Z (Å)
C	11.57837314	9.50082890	12.18007073
C	11.02317021	9.68157026	10.91456911
C	9.85224813	8.99879469	10.58374988
C	9.20180063	8.13013487	11.47289867
C	9.77652216	7.97078873	12.75868460
C	10.95870727	8.65327886	13.09588395
P	7.60003306	7.41796098	10.79822235
C	7.30532161	5.94482158	11.80790869
C	8.35324511	5.03141302	11.95088304
C	8.18313245	3.83699537	12.65195344
C	6.93883162	3.55468269	13.21689962
C	5.86891468	4.43849376	13.06321465
C	6.03890006	5.62841936	12.34231510
C	6.54429498	8.87307138	11.07814942
C	6.05028420	9.53151074	9.94756266
C	5.35974908	10.74041658	10.06428575
C	5.16248077	11.29553864	11.32849861
C	5.64229301	10.65169743	12.47188822
C	6.33319900	9.44032290	12.35114023
H	12.49479823	10.01970901	12.46500315
H	11.49273293	10.34320794	10.18698013

H	9.41997524	9.13778858	9.59108083
H	11.39897615	8.52207756	14.08259474
H	9.32336333	5.27594055	11.51459210
H	9.01293723	3.13873748	12.75826689
H	6.78698629	2.62961116	13.77520881
H	4.90103945	4.19089643	13.49588015
H	6.20677416	9.06431286	8.97474728
H	4.97495237	11.23872674	9.17457598
H	4.62504395	12.23890471	11.43628344
H	5.46776978	11.09315466	13.45173088
O	6.66628066	6.79791753	9.44106144
O	8.16768573	7.21856383	9.28292130
O	5.03675029	6.51864571	12.11425647
C	3.74275798	6.23406371	12.63747862
H	3.76073462	6.16787839	13.73767815
H	3.33813279	5.29823384	12.21964726
H	3.10940424	7.07361687	12.33176721
O	9.15200321	7.14536953	13.64534695
C	9.78070263	6.87613563	14.89246076
H	9.85300812	7.78293694	15.51665609
H	10.78775878	6.44921575	14.75562921
H	9.13997847	6.13957824	15.39026135
O	6.80667547	8.73150291	13.41111290
C	6.71704963	9.30646094	14.70871111
H	5.66766199	9.45072330	15.01416142
H	7.24843618	10.27098410	14.75673893
H	7.19595559	8.58890559	15.38361918

Step 3 (TS) : Tris(*o*-methoxyphenyl)phosphine
Energy: -238.6876237862457 eV

Atom	X (Å)	Y (Å)	Z (Å)
C	11.47718714	9.37352295	12.60148606
C	10.99815957	9.73715063	11.34541997
C	9.83883960	9.13358166	10.85119026
C	9.12676496	8.17635241	11.58114657
C	9.60638653	7.84425003	12.86936920
C	10.78368440	8.43259949	13.36109091
P	7.60551502	7.44734267	10.62584743
C	7.37297102	5.92556060	11.62056713
C	8.34344694	4.93013035	11.47141947
C	8.25946425	3.73674505	12.18584046
C	7.18664236	3.53082030	13.05729909
C	6.18440512	4.49516428	13.18924893
C	6.25978299	5.68474048	12.45450512
C	6.54714106	8.93379851	10.87100997
C	5.96665430	9.45525140	9.70919686
C	5.16798194	10.59857477	9.75051099
C	4.93573357	11.23081424	10.97134060
C	5.50614721	10.73303781	12.14467141
C	6.31557865	9.59144392	12.09868556

H	12.38848211	9.82027725	13.00090272
H	11.52653399	10.47523218	10.74150374
H	9.48357440	9.39352085	9.85239042
H	11.15535229	8.16721737	14.35000236
H	9.17629374	5.11644808	10.79050817
H	9.02808783	2.97282928	12.07002391
H	7.11476444	2.60531840	13.63094487
H	5.34394841	4.30842026	13.85672019
H	6.13595747	8.92593389	8.77104100
H	4.71745746	10.98365053	8.83544938
H	4.29958414	12.11593730	11.02100409
H	5.31299765	11.23669302	13.09092379
O	6.57903801	6.73110812	9.50989061
O	8.45851789	7.34695597	9.29809577
O	5.30808826	6.65343495	12.48314953
C	4.20213586	6.49287876	13.36602801
H	4.53484817	6.43244102	14.41528117
H	3.61522955	5.59532703	13.11463259
H	3.58196778	7.38485384	13.22794679
O	8.88301490	6.96353900	13.62084821
C	9.45229297	6.46192838	14.82555175
H	9.55164735	7.25164845	15.58963700
H	10.43874405	6.00461783	14.64562481
H	8.75554220	5.69432654	15.18071436
O	6.88576616	9.03879113	13.20330528
C	6.69152505	9.66531230	14.46496270
H	5.62768002	9.66198862	14.75539781
H	7.07010360	10.70023398	14.46584485
H	7.26677520	9.06665588	15.17967171

Step 3 (FS) : Tris(*o*-methoxyphenyl)phosphine
Energy: -243.09299938152478 eV

Atom	X (Å)	Y (Å)	Z (Å)
C	11.68971823	8.78672697	12.74210461
C	11.47520757	9.09057611	11.39748719
C	10.22382638	8.84023073	10.83783374
C	9.16931503	8.30322834	11.59460242
C	9.40127786	8.00299678	12.95937595
C	10.66671585	8.24173007	13.51774686
P	7.65139595	8.03445272	10.63463725
C	7.04148728	5.69551064	11.74916467
C	7.95861885	4.96698634	10.99739790
C	8.25443313	3.64401287	11.34569286
C	7.62293751	3.05433462	12.43715692
C	6.68920155	3.77878081	13.18703208
C	6.38831707	5.10383768	12.85416447
C	6.61989924	9.51850833	10.64464001
C	6.23271419	9.99692188	9.38581765
C	5.41996697	11.12496292	9.26151135
C	4.99481528	11.78764406	10.41324835

C	5.37588433	11.33825491	11.67984810
C	6.19308670	10.20622229	11.80276736
H	12.66386576	8.96765056	13.19869472
H	12.27642760	9.50770185	10.78790051
H	10.04471031	9.03624343	9.77959092
H	10.85769112	8.00376837	14.56269305
H	8.42169890	5.44435982	10.13262090
H	8.97303255	3.08013192	10.75134949
H	7.84483134	2.02316130	12.71312215
H	6.19579671	3.30489532	14.03381777
H	6.58682950	9.45103700	8.50975302
H	5.12258623	11.48220196	8.27588456
H	4.35904846	12.67085393	10.33397116
H	5.03732286	11.87435043	12.56481232
O	6.70533164	7.00720862	11.46218946
O	7.98205018	7.62519123	9.24280634
O	5.50430787	5.89581474	13.53345292
C	4.78878174	5.29737207	14.60801026
H	5.46624756	4.96198410	15.41222374
H	4.18769231	4.43888249	14.26548989
H	4.12396687	6.07726847	14.99468863
O	8.37184329	7.47438880	13.67194526
C	8.61899322	7.02203575	14.99782263
H	8.90632510	7.85292798	15.66373392
H	9.40175795	6.24636758	15.01941740
H	7.67006552	6.59309522	15.33718476
O	6.61937555	9.70821736	12.99289410
C	6.15385647	10.31709359	14.19145254
H	5.05467966	10.27508583	14.25947087
H	6.48758670	11.36483706	14.26878451
H	6.59262556	9.73248491	15.00714072

S6. MD Simulations of Biaryl Phosphines Initialized from A-away Conformations

To examine whether the trend observed for Compounds **35'**, **41'**, and **44'** extends to other biaryl phosphines, MD simulations were additionally performed for Compounds **30'**, **33'**, **37'**, **40'**, **43'**, **49'**, and **50'** using A-away geometries as initial structures. For completeness, the corresponding results for Compounds **35'**, **41'**, and **44'** are also included in Table S5. As summarized in Table S5, the resulting τ were substantially smaller than those from standard A-conformation-initialized simulations in all cases, consistent with the results described in Section 3.2.2.

Table S5. τ and 95% confidence intervals (τ_{low}, τ_{up}) from MD simulations initialized from A-away conformations for representative biaryl phosphines.

Compound	τ (ps)	τ_{low} (ps)	τ_{up} (ps)
30'	25.1	18.6	34.9
33'	42.5	31.0	60.4

35'	42.4	30.5	61.3
37'	35.5	24.9	53.2
40'	71.1	52.8	99.2
41'	38.9	26.9	59.2
43'	73.9	55.0	102.5
44'	34.4	24.4	50.7
49'	88.8	65.6	124.3
50'	328.7	181.5	689.3

S7. Comparison of Regression Models

To justify the selection of the random forest (RF) model used in Section 3.3.2, six additional regression models were also evaluated: linear regression, ridge, regression lasso regression, elastic net regression, support vector regression with an RBF kernel (SVR-RBF), and gradient boosting (GB). The raw LOO-CV prediction results for all seven models, including the RF model, are provided in Table S6.

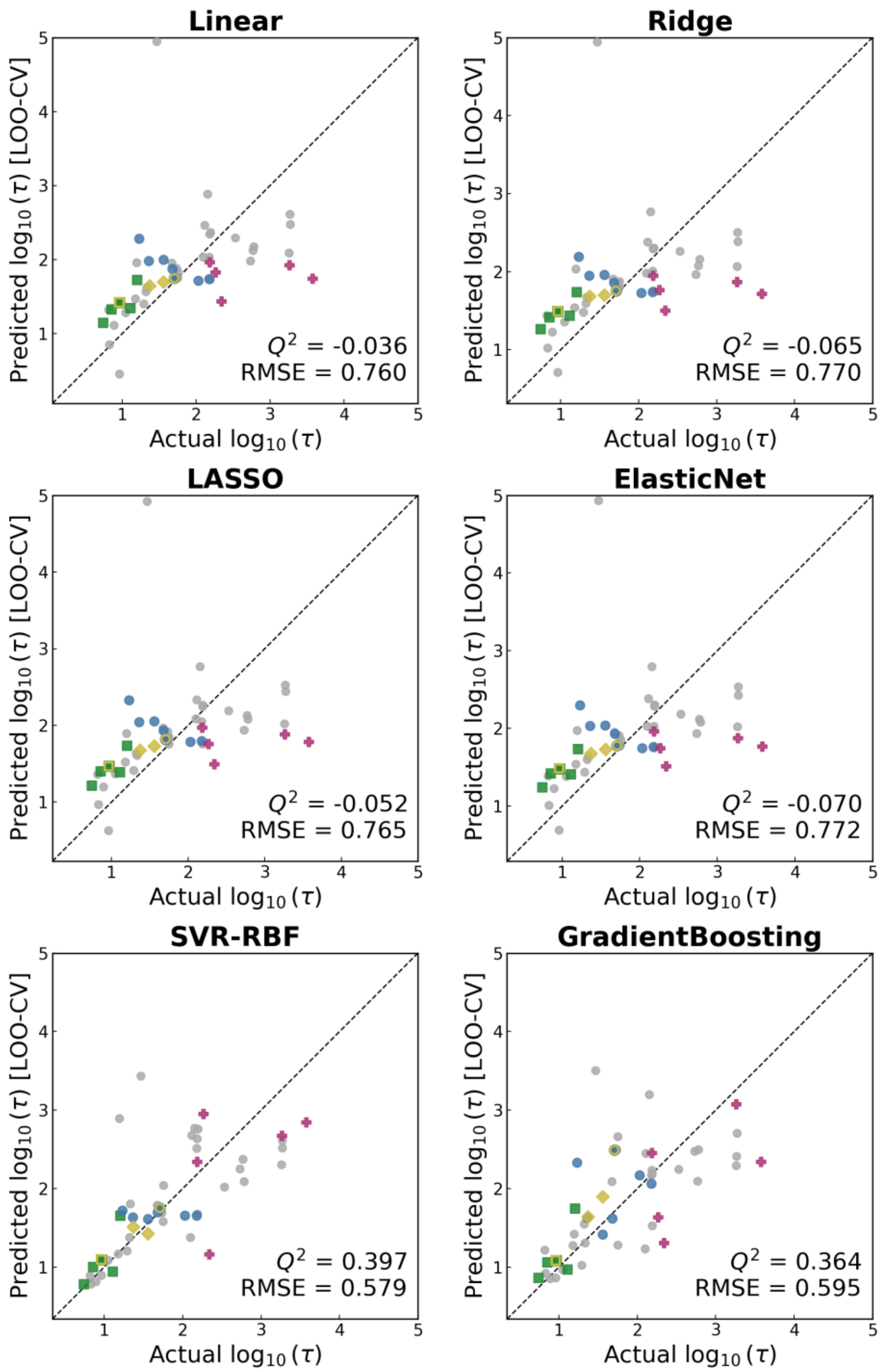

Figure S6. Cross-validated Q^2 for six regression models of τ.

Table S6. LOO-CV prediction results.

Compound	Actual ($\log_{10}\tau$)	Linear	Ridge	LASSO	Elastic Net	SVR-RBF	RF	GB
1	0.74	1.15	1.26	1.22	1.24	0.79	1.08	0.86
2	0.82	1.33	1.44	1.36	1.39	0.89	1.49	1.22
3	0.83	0.85	1.02	0.97	1.01	0.78	1.01	0.93
4	0.85	1.33	1.41	1.40	1.42	1.00	1.18	1.06
5	0.89	1.11	1.23	1.20	1.22	0.82	1.05	0.85
6	0.96	0.46	0.71	0.63	0.69	0.90	0.92	0.86
7	0.96	1.42	1.49	1.47	1.48	1.10	1.07	1.08
8	1.05	1.29	1.36	1.37	1.38	1.09	1.07	0.97
9	1.11	1.35	1.43	1.39	1.41	0.94	1.15	0.97
10	1.18	1.47	1.54	1.52	1.54	1.17	1.34	1.28
11	1.19	1.96	2.03	1.90	1.97	2.90	1.88	1.42
12	1.20	1.73	1.74	1.74	1.74	1.66	1.56	1.75
13	1.23	2.29	2.19	2.33	2.30	1.72	1.90	2.33
14	1.29	1.40	1.48	1.41	1.44	1.21	1.26	1.03
15	1.32	1.57	1.59	1.63	1.60	1.38	1.65	1.55
16	1.33	1.63	1.67	1.61	1.64	1.81	1.39	1.31
17	1.36	1.98	1.95	2.04	2.03	1.63	1.64	1.63
18	1.37	1.65	1.68	1.68	1.68	1.51	1.82	1.64
19	1.47	4.95	4.95	4.92	4.93	3.44	2.76	3.51
20	1.56	2.00	1.96	2.05	2.04	1.61	1.57	1.42
21	1.56	1.70	1.70	1.73	1.73	1.43	1.93	1.90
22	1.67	1.96	1.90	1.97	1.95	1.79	1.77	2.09
23	1.68	1.88	1.86	1.94	1.93	1.70	1.74	1.62
24	1.71	1.75	1.76	1.82	1.78	1.75	1.92	2.49
25	1.73	1.88	1.85	1.92	1.91	1.69	1.92	2.50
26	1.75	1.76	1.80	1.76	1.78	1.59	1.68	1.28
27	1.75	1.84	1.87	1.84	1.86	2.04	2.11	2.66
28	2.03	1.72	1.73	1.78	1.75	1.66	1.82	2.17
29	2.10	2.03	1.98	2.09	2.03	1.38	1.46	1.23
30	2.11	2.47	2.38	2.34	2.38	2.68	2.25	2.45
31	2.15	2.89	2.77	2.77	2.80	2.77	2.61	3.20
32	2.18	1.74	1.74	1.80	1.76	1.66	1.93	2.07
33	2.18	2.03	2.01	2.05	2.03	2.51	2.27	2.17

34	2.18	1.97	1.95	1.97	1.96	2.34	2.26	2.46
35	2.18	2.35	2.29	2.25	2.29	2.64	2.29	2.24
36	2.19	1.73	1.75	1.76	1.75	1.68	1.85	1.53
37	2.19	2.37	2.31	2.27	2.30	2.76	2.34	2.19
38	2.26	1.83	1.77	1.76	1.75	2.95	2.37	1.63
39	2.34	1.44	1.50	1.50	1.51	1.17	1.19	1.31
40	2.53	2.30	2.26	2.19	2.19	2.02	2.34	2.25
41	2.73	1.98	1.97	1.94	1.94	2.25	2.43	2.48
42	2.77	2.13	2.08	2.13	2.12	2.37	2.05	2.10
43	2.78	2.18	2.16	2.08	2.08	2.09	2.54	2.50
44	3.26	2.09	2.07	2.02	2.02	2.31	2.48	2.29
45	3.26	1.93	1.87	1.89	1.87	2.67	2.65	3.08
46	3.27	2.61	2.51	2.52	2.54	2.62	2.51	2.41
47	3.27	2.48	2.39	2.44	2.43	2.52	2.42	2.71
48	3.58	1.74	1.72	1.79	1.77	2.84	2.31	2.34

S8. Accuracy Validation

The applicability of PFP to phosphines was validated using the Kraken database.[4] Kraken contains 31,175 phosphine structures with their associated electronic/steric descriptors by DFT calculations. We compared PFP with DFT for 31,175 phosphine structures listed in Kraken along two respects:

(a) validation of single-point energies through comparison of formation energies, and
(b) validation of structure optimization through comparison of structure-optimised by the percent buried volume (%V_{bur}),[5,6] which is the steric descriptors.

S6.1. Methods

All PFP calculations were carried out with PFP version 5.0.0 and PBE_PLUS_D3 modes using the L-BFGS algorithm with a force convergence threshold of fmax = 0.001 eV Å^{-1}.[1,2] The DFT calculations were conducted at the B3LYP-D3/6-31G(d,p) (SDD for Ni), using the Gaussian 16 program package.[7]

The formation energy (E_{form}) was defined as

$$E_{\text{form}} = E_{\text{pot}} - E_{\text{shift}} = E_{\text{pot}} - \sum_i N_{i,\text{atom}}\, E_{i,\text{atom}}\,, \tag{S1}$$

where E_{pot} is potential energy, $N_{i,\mathrm{atom}}$ is the number of atoms of type (i), and $E_{i,\mathrm{atom}}$ is the shift value for element i. For example, in the case of PMe_3,

$$E_{\mathrm{form,PMe_3}} = E_{\mathrm{pot,PMe_3}} - (E_{\mathrm{P}} \times 1 + E_{\mathrm{C}} \times 3 + E_{\mathrm{H}} \times 9). \qquad \text{(S2)}$$

For the DFT reference, $\%V_{\mathrm{bur}}$ was conducted directly on the DFT-optimized geometries provided in Kraken. For the PFP comparison, we took the same Kraken DFT-optimized geometries as initial coordinates and re-optimized them with PFP, after which $\%V_{\mathrm{bur}}$ was conducted. This protocol isolates the effect of the optimizer (PFP vs DFT).

S6.2. Results

The $E_{i,\mathrm{atom}}$ of each element for DFT is shown in Table S7. These values were used to obtain E_{pot}. For PFP, however, the $E_{i,\mathrm{atom}}$ of all elements was zero because each element energy was standardized to zero.

Table S7. Potential energies of individual atoms as DFT shift values.

Element	Shift Value /eV
H	-13.7888753830889
B	-670.523278867987
C	-1029.36337326082
N	-1484.79049157295
O	-2042.19372775746
F	-2713.35629551101
Si	-7873.70249203826
P	-9285.29119333512
S	-10832.3381054273
Cl	-12520.6607968260
Se	-65288.5390876611
Br	-69978.4093998688

Validation results are summarized in Figure S7. The formation energy comparison gives an MAE of 0.13 eV and RMSE of 0.22 eV. From Figure S7(b), $\%V_{\mathrm{bur}}$ shows an MAE of 0.92% and RMSE of 1.63% between PFP and DFT. These results indicate that, for phosphines, PFP can accurately reproduce DFT energies and optimized structures, supporting its use for large-scale screening and descriptor generation.

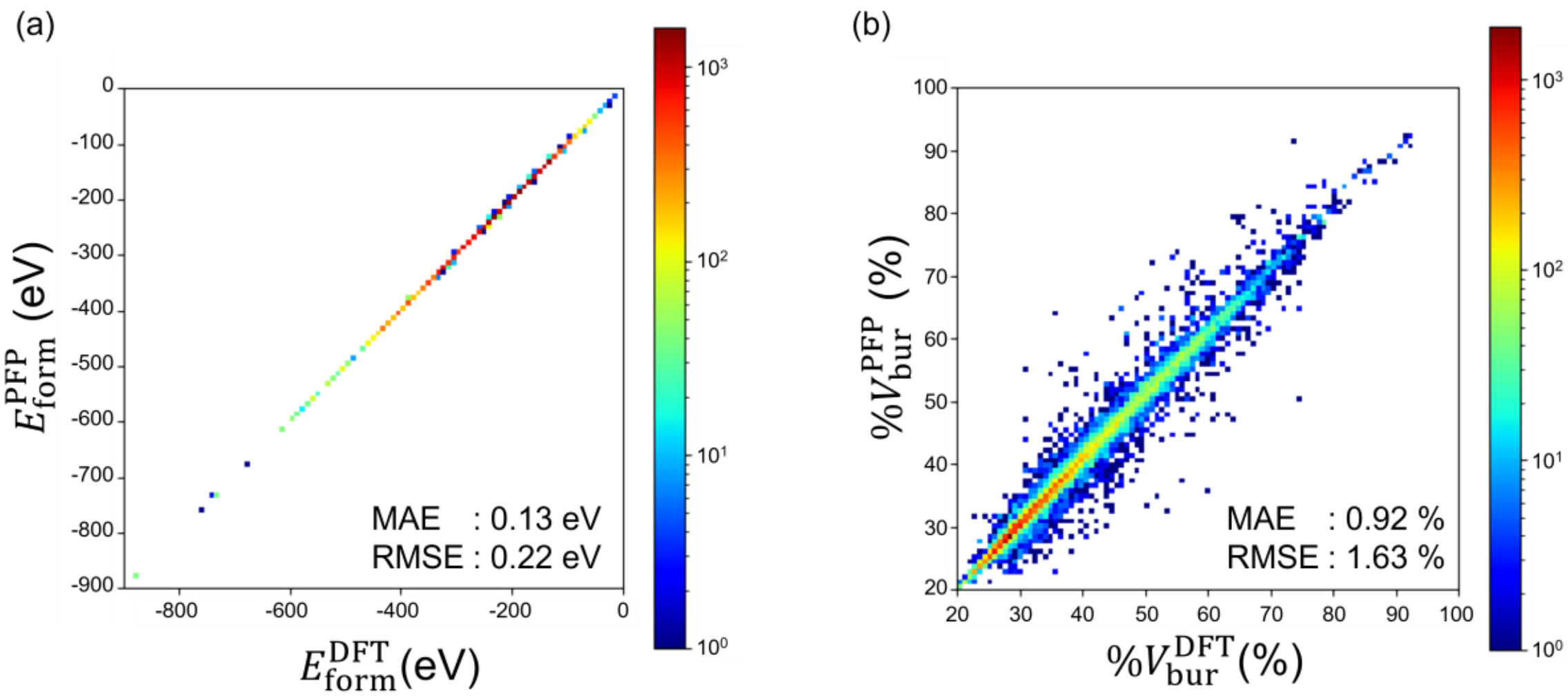

Figure S7. Validation results from comparison of NNP and DFT: (a) formation energy and (b) $\%V_{bur}$.

The underlying numerical data used for these analyses, including E_{form}, $\%V_{bur}$, and related intermediate quantities for a total of 31 175 phosphines, are provided separately in the accompanying XLSX Supporting Information file (corresponding to Table S8).

S9. Conformer Generation and Descriptor Calculations

For each of the 51 phosphines, we generated a conformer ensemble, conducted geometry optimizations, and selected the most stable conformer, and conducted electronic/steric descriptors used in Section 3.3 to assess the chemical validity of τ: the percent buried volume ($\%V_{bur}$) and the Mulliken charge on phosphorus (Q_P). In addition, the SOMO energy of the corresponding phosphine radical cation was calculated as a third descriptor used in Section 3.3.

S9.1 Conformer Selection

Initial conformers were generated with RDKit (rdkit.Chem.AllChem.EmbedMultipleConfs) under the following settings:[8]

- Number of conformers: 50
- pruneRmsThresh: 0.1 Å
- randomSeed: 1234
- useExpTorsionAnglePrefs = True
- useBasicKnowledge = True
- useRandomCoords = True

The 50 generated conformers for each phosphine were ranked by PFP (version 5.0.0, PBE_PLUS_D3) energies using the L-BFGS algorithm with a force convergence threshold of fmax

= 0.001 eV Å^{-1}.[2,3] The lowest-energy conformer was then adopted as the representative structure for subsequent descriptor calculations.

S9.2. Result of Descriptor Calculation for 51 Phosphines

The steric and electronic descriptors calculated for the most stable conformer of each of the 51 target phosphines are summarized in Table S9, together with the SOMO energy calculated for the corresponding phosphine radical cation. For phosphines bearing two phosphorus atoms, two sets of descriptor values are listed, corresponding to the individual phosphorus atoms within the molecule.

Table S9. Calculated descriptors (%V_{bur}, Q_P, and SOMO energy).

Compound	%V_{bur}	Q_P	SOMO energy (eV)
1	0.410	0.320	-11.34
2	0.418	0.431	-11.11
3	0.402	0.255	-11.82
4	0.388	0.311	-10.78
5	0.399	0.276	-11.28
6	0.401	0.222	-12.46
7	0.397	0.348	-10.66
8	0.383	0.238	-10.77
9	0.408	0.368	-10.89
10	0.384	0.357	-10.47
11	0.436	0.767	-10.48
12	0.399	0.370	-10.00
13	0.325	0.284	-8.52
14	0.429	0.413	-10.92
15	0.369	0.259	-10.08
16	0.428	0.451	-10.47
17	0.333	0.283	-9.08
18	0.382	0.381	-10.10
19	0.514	0.428	-7.51
20	0.333	0.283	-9.01
21	0.379	0.284	-9.86
22	0.371	0.281	-9.26
23	0.339	0.283	-9.27
24	0.341	0.283	-9.54

25	0.358	0.279	-9.34
26	0.397	0.509	-10.05
27	0.387	0.510	-9.82
28	0.346	0.284	-9.59
29	0.328	0.274	-8.81
30	0.433	0.615	-8.84
31	0.769, 0.379	0.836, 0.731	-7.96
32	0.353	0.284	-9.57
33	0.356	0.393	-9.08
34	0.369	0.408	-9.32
35	0.419	0.622	-9.00
36	0.379	0.386	-9.85
37	0.422	0.602	-8.94
38	0.442	0.269	-9.77
39	0.381	0.336	-10.42
40	0.425	0.647	-9.09
41	0.410	0.483	-9.54
42	0.353	0.400	-8.81
43	0.425	0.632	-9.28
44	0.413	0.563	-9.32
45	0.460	0.350	-9.43
46	0.746, 0.373	0.711, 0.583	-7.94
47	0.696, 0.342	0.727, 0.502	-7.94
48	0.454	0.283	-9.50
49	0.387	0.574	-8.77
50	0.419	0.555	-9.37
51	0.435	0.255	-9.66

S9.3 Cartesian Coordinates of Most Stable Conformers

Compound **1**
Energy: -127.7486272160473 eV

Atom	X (Å)	Y (Å)	Z (Å)
C	1.17352628	-1.88979343	-0.22026982
C	-0.10994818	-1.39038940	-0.89116871
C	-0.04096624	-1.58474197	-2.41375307
P	-0.67581139	0.36700397	-0.50712616

C	-1.63845482	0.14861743	1.08834876
C	-0.94352325	-0.58425464	2.23748644
C	-3.00122305	-0.48138989	0.77014207
C	0.88280192	1.23151379	0.07183217
C	1.77745214	1.50598164	-1.14443179
C	0.52858539	2.54400173	0.78040353
H	2.05647019	-1.34865086	-0.58936694
H	1.15123396	-1.78710569	0.87163979
H	1.32833894	-2.95551366	-0.45282808
H	-0.94941420	-2.00487202	-0.52006765
H	-0.98069747	-1.28651920	-2.89816479
H	0.76394790	-0.97979728	-2.85717518
H	0.16020346	-2.63846086	-2.66296664
H	-1.82617751	1.18967599	1.40217462
H	-0.78954647	-1.64575156	1.99495957
H	-1.56335482	-0.54514394	3.14763174
H	0.03530725	-0.14874151	2.48242006
H	-2.89407171	-1.52820806	0.44672862
H	-3.51989035	0.06421406	-0.03037481
H	-3.64598389	-0.48114157	1.66250299
H	1.43043994	0.58452391	0.77656420
H	1.25255639	2.13976686	-1.87507648
H	2.69497633	2.03128288	-0.83661338
H	2.07776128	0.58468174	-1.66145729
H	1.44272384	3.11754008	0.99862577
H	0.00763765	2.37651573	1.73254210
H	-0.11649877	3.17272497	0.14721234

Compound **2**
Energy: -166.63355150912963 eV

Atom	X (Å)	Y (Å)	Z (Å)
C	-4.09009507	1.15176303	-0.08057509
C	-2.81013684	0.44141512	-0.51604569
C	-1.70598315	1.41294838	-0.93822209
C	-0.40132212	0.72920877	-1.36153763
P	0.43947848	-0.20036189	0.01606677
C	1.27942047	1.23284630	0.85657952
C	2.06269742	0.84627591	2.11808773
C	1.23613641	0.14686620	3.20547605
C	0.05991910	0.97529721	3.72329514
C	1.89255265	-0.87521867	-0.93293648
C	1.57629487	-2.14872502	-1.72429544
C	1.22471980	-3.35277764	-0.84791099
C	0.90621079	-4.60566182	-1.66111492
H	-4.87134671	0.43621372	0.21183825
H	-4.49595391	1.77729711	-0.89008887
H	-3.90245210	1.81011104	0.78136312
H	-2.43554587	-0.19599244	0.30118482
H	-3.02619031	-0.24098444	-1.35581011
H	-1.50723666	2.11673336	-0.11109356

H	-2.07198963	2.03428108	-1.77382541
H	0.30750368	1.46457385	-1.78028870
H	-0.61129356	-0.00150328	-2.16052552
H	0.49065720	1.95756739	1.11299968
H	1.94406209	1.73540636	0.13245537
H	2.90771666	0.19562652	1.83875349
H	2.51349406	1.76052744	2.54176028
H	0.85996808	-0.81094629	2.81134240
H	1.90647738	-0.10219388	4.04360263
H	-0.44489392	0.46877656	4.55777320
H	0.39290739	1.96110085	4.08354286
H	-0.69411940	1.14117761	2.94017005
H	2.68558203	-1.08821818	-0.19644422
H	2.28059332	-0.08544566	-1.59944262
H	0.74579772	-1.95622007	-2.42582506
H	2.44522196	-2.40299661	-2.35551306
H	2.06625110	-3.55376963	-0.16300385
H	0.36841396	-3.09175255	-0.20523571
H	0.66203994	-5.45852237	-1.01237501
H	1.75809457	-4.89966753	-2.29295936
H	0.04587155	-4.43667232	-2.32640059

Compound **3**
Energy: -88.77093679922959 eV

Atom	X (Å)	Y (Å)	Z (Å)
C	0.19152697	-3.09968803	3.53287581
C	-0.67435127	-2.53060154	2.40721814
P	0.28009028	-2.23209490	0.83963193
C	-1.03463718	-1.36099105	-0.14530438
C	-0.51283024	-0.79855773	-1.46896878
C	1.38191290	-0.81212535	1.33446942
C	0.74204736	0.41632151	1.98167929
H	0.95218101	-2.37628622	3.86006810
H	-0.41726023	-3.35596143	4.41180840
H	0.71757691	-4.00980753	3.21010745
H	-1.46629231	-3.24838818	2.13977807
H	-1.18319175	-1.60881710	2.73424568
H	-1.81223943	-2.11774001	-0.33652731
H	-1.50910383	-0.56775419	0.45568367
H	0.22113940	0.00331104	-1.30387113
H	-1.33011509	-0.37647805	-2.07121781
H	-0.02113771	-1.57897115	-2.06765473
H	2.14427068	-1.24679645	2.00040871
H	1.92670837	-0.52732451	0.42033829
H	1.50266379	1.17392293	2.22282022
H	0.00719915	0.89105434	1.31647840
H	0.22614926	0.16152773	2.91804801

Compound **4**
Energy: -189.16654468242268 eV

Atom	X (Å)	Y (Å)	Z (Å)
C	-0.91530884	3.75316200	-0.85269331
C	0.50111131	3.77228529	-0.23661901
C	1.03727012	2.35317260	-0.46523928
C	-0.19694657	1.47051207	-0.20497503
P	-0.00159468	-0.24606035	-0.88041822
C	-1.57546144	-1.12330341	-0.36982121
C	-2.39095548	-0.67441327	0.88362563
C	-3.86805202	-0.59857397	0.42956357
C	-3.93245011	-1.46429719	-0.83602766
C	-2.60311429	-1.14955644	-1.52814128
C	1.25670827	-0.94434515	0.30481689
C	1.65543450	-2.39051177	-0.10519248
C	1.90018282	-3.13226441	1.21676013
C	0.85206752	-2.52253191	2.15529512
C	0.90279646	-1.02193537	1.82085033
C	-1.34411570	2.25836272	-0.87911429
H	-1.61676984	4.37390477	-0.27881257
H	-0.89726996	4.15945940	-1.87392830
H	0.44145797	3.96784475	0.84567990
H	1.14419260	4.55027616	-0.67097857
H	1.88848227	2.10588273	0.18454431
H	1.36909129	2.22919873	-1.51026542
H	-0.36764201	1.44170202	0.88567161
H	-1.24275680	-2.16248719	-0.20778995
H	-2.26896587	-1.41047041	1.69136033
H	-2.05484841	0.28893195	1.28907506
H	-4.12694287	0.44023840	0.16956031
H	-4.56832367	-0.91958993	1.21325992
H	-4.81028622	-1.25104402	-1.46233579
H	-3.97026656	-2.53231817	-0.56391938
H	-2.66381656	-0.15900054	-2.00784499
H	-2.32421386	-1.86858963	-2.31063866
H	2.12678720	-0.28320053	0.16859874
H	2.52076405	-2.40535921	-0.78143303
H	0.82766704	-2.87694654	-0.64798593
H	1.81830799	-4.22389069	1.11711128
H	2.91115429	-2.91014528	1.59662983
H	-0.14303795	-2.93097641	1.91235394
H	1.04054796	-2.73012822	3.21801968
H	-0.03189825	-0.50554597	2.07357277
H	1.69892930	-0.54022965	2.40965944
H	-1.46368307	1.91813286	-1.91964864
H	-2.30640152	2.08603975	-0.37789733

Compound **5**
Energy: -127.76825346645813 eV

Atom	X (Å)	Y (Å)	Z (Å)
C	0.64243979	2.54797041	-0.06267894

P	-0.70017933	1.52436202	0.69485475
C	-1.00151230	-0.27475105	-1.75644955
C	-1.57980274	0.74554860	-0.75550495
C	-2.14157769	-0.81344303	-2.63126686
C	0.11325038	0.29039281	-2.63855077
C	0.24880634	0.14408784	1.56923877
C	0.75200679	0.76662888	2.88486497
C	1.44567712	-0.43999937	0.81078065
C	-0.76686620	-0.96134793	1.89659331
H	0.16951516	3.31814282	-0.68798267
H	1.17387853	3.06687269	0.74728838
H	1.37565495	2.00582715	-0.67197327
H	-0.59521043	-1.12810062	-1.18760688
H	-1.96592584	1.61644048	-1.31786658
H	-2.47553960	0.29868514	-0.29128345
H	-2.59672021	0.00017893	-3.21828902
H	-2.93523757	-1.26836634	-2.02074884
H	-1.77640492	-1.57274857	-3.33830731
H	-0.23009289	1.19563054	-3.16373102
H	1.00796107	0.55619543	-2.06347609
H	0.41454434	-0.44330135	-3.40039278
H	1.26133088	0.00043441	3.49179481
H	-0.07867900	1.17865653	3.47526245
H	1.47285555	1.57711832	2.70165718
H	2.20994882	0.32401883	0.61195786
H	1.15867604	-0.89132503	-0.14689852
H	1.92067732	-1.22784151	1.41922993
H	-0.30346040	-1.71450918	2.55383317
H	-1.11689713	-1.47866239	0.99238001
H	-1.64579498	-0.55438618	2.41913145

Compound **6**

Energy: -50.020143133349016 eV

Atom	X (Å)	Y (Å)	Z (Å)
C	1.40891811	1.43064025	-0.40520228
P	0.07465479	0.87322996	-1.55885047
C	-1.39254708	1.11784019	-0.45920194
C	0.27517163	-0.95756922	-1.38644736
H	1.34340717	0.93913273	0.57809968
H	1.34082741	2.51824680	-0.26446666
H	2.39086808	1.21240327	-0.84731335
H	-2.28465451	0.69060925	-0.93768520
H	-1.25775302	0.64808410	0.52767080
H	-1.57123317	2.19301509	-0.31995938
H	-0.55089849	-1.46683853	-1.90181277
H	1.21208127	-1.27082580	-1.86748144
H	0.29032435	-1.27882425	-0.33320473

Compound **7**

Energy: -228.3548396730654 eV

Atom	X (Å)	Y (Å)	Z (Å)
C	-4.19079619	-0.59210590	-2.51919960
C	-2.94554776	0.31375253	-2.59994916
C	-2.52490190	0.80206368	-1.21279733
C	-2.07185702	-0.37759376	-0.31453416
P	-0.23328166	-0.65385982	-0.52026793
C	0.06296582	-2.10223158	0.62909841
C	-0.33417161	-1.92980305	2.10153280
C	-0.09395026	-3.21578909	2.90331365
C	1.36175187	-3.67904733	2.78650506
C	1.77271064	-3.85048669	1.32073556
C	1.52337348	-2.57090209	0.51325204
C	0.56256499	0.77153159	0.41681703
C	-0.30658768	1.53577403	1.42834089
C	0.49988686	2.61731333	2.15936827
C	1.15700762	3.58769000	1.17306898
C	2.02469098	2.83732088	0.15802408
C	1.22597731	1.75080460	-0.57027305
C	-2.83254464	-1.67468920	-0.67561753
C	-4.24256142	-1.36687839	-1.18720723
H	-4.18539982	-1.29773378	-3.36427185
H	-5.10590690	0.01046505	-2.62801451
H	-3.13531396	1.16678955	-3.26771766
H	-2.10155346	-0.24165993	-3.04005331
H	-1.72141292	1.54899832	-1.30343657
H	-3.37086128	1.32230399	-0.73271707
H	-2.29321087	-0.12249900	0.73381720
H	-0.56813894	-2.90174771	0.19793608
H	-1.38770199	-1.62140896	2.18703944
H	0.26793325	-1.11879768	2.54636380
H	-0.75944094	-4.00981785	2.52073859
H	-0.36491344	-3.06004323	3.95933321
H	2.01757699	-2.92681830	3.25967728
H	1.51232768	-4.61881622	3.33999774
H	1.18970706	-4.67578509	0.87522197
H	2.83242160	-4.14175890	1.24991424
H	1.78421254	-2.72389612	-0.54518991
H	2.19296974	-1.77528166	0.88661690
H	1.38117903	0.28422891	0.98090350
H	-1.14322406	2.01930643	0.89673493
H	-0.75713762	0.84678692	2.15700238
H	-0.15195044	3.16268309	2.85987705
H	1.28354469	2.13442565	2.76980735
H	0.36873529	4.14260963	0.63402334
H	1.75619639	4.33780091	1.71217105
H	2.87357057	2.36702264	0.68556838
H	2.45919170	3.53912625	-0.57105696
H	1.87222853	1.19521374	-1.26661839
H	0.44625679	2.22767713	-1.19046674
H	-2.28257519	-2.22848364	-1.45739599
H	-2.87474797	-2.33888336	0.20011595

H	-4.81906649	-2.29617393	-1.30788333
H	-4.77427379	-0.77165706	-0.42649157

Compound **8**
Energy: -93.26432353485951 eV

Atom	X (Å)	Y (Å)	Z (Å)
C	1.80674742	-1.13791673	-1.79849832
P	1.33361358	-0.85023820	-0.02943853
C	2.41063189	0.60436516	0.33682606
C	-0.31027686	-0.07040791	-0.26392357
C	-1.35689059	-0.87943657	-0.74468651
C	-2.64362147	-0.36727689	-0.90809356
C	-2.91808447	0.96249284	-0.57436611
C	-1.89381266	1.77055215	-0.07788281
C	-0.60122299	1.26052511	0.07573607
H	2.83635946	-1.52047751	-1.83329599
H	1.14873894	-1.90147428	-2.23342609
H	1.73675958	-0.22203820	-2.40274414
H	2.31122589	1.41455057	-0.40077449
H	3.45295549	0.25691044	0.34195205
H	2.18986135	0.99483794	1.33941072
H	-1.16213897	-1.92657688	-0.98847983
H	-3.43865400	-1.01105197	-1.28805035
H	-3.92515576	1.36337587	-0.69487859
H	-2.09847064	2.80786470	0.19262427
H	0.18181182	1.91288811	0.46292458

Compound **9**
Energy: -166.27879712267423 eV

Atom	X (Å)	Y (Å)	Z (Å)
C	-1.14030964	1.14431789	-1.53412505
C	-0.03581638	1.75221336	-0.65893785
C	-0.66522646	2.70734264	0.38065363
C	0.84963322	2.65045548	-1.54361039
P	1.09676051	0.54973862	0.32009586
C	-0.01722872	-0.85016532	1.01741566
C	-1.23258600	-0.20928878	1.71434168
C	0.78578659	-1.52676232	2.15166930
C	-0.52453036	-1.91785038	0.03862131
C	2.30844294	-0.25082399	-0.93609816
C	1.75187535	-0.66638119	-2.30464306
C	3.46613951	0.75460215	-1.13082623
C	2.95524171	-1.48406736	-0.27715139
H	-1.88586693	0.59291811	-0.94851654
H	-1.67473744	1.95512134	-2.05777485
H	-0.74062202	0.46470848	-2.29688333
H	0.11164771	3.15799579	1.01451686
H	-1.39373664	2.21957064	1.03452170

H	-1.18757409	3.52036638	-0.14989165
H	1.67028409	3.09977153	-0.96701880
H	0.23015341	3.47249190	-1.93723751
H	1.27375365	2.12236391	-2.40509483
H	-1.95954073	0.21210942	1.01087313
H	-0.92494209	0.57809836	2.41662235
H	-1.75343645	-0.98810007	2.29451320
H	0.12353126	-2.22123608	2.69432640
H	1.15409185	-0.77784762	2.86719374
H	1.64393117	-2.10249166	1.79341167
H	-1.11200418	-1.48605512	-0.78104262
H	-1.17681435	-2.62427916	0.57966698
H	0.28968327	-2.50506587	-0.40302850
H	2.55585432	-1.13918166	-2.89399072
H	1.38515514	0.18740289	-2.88720401
H	0.93384037	-1.39226564	-2.21857881
H	3.17088349	1.65845730	-1.67123358
H	3.88264215	1.05999149	-0.16041658
H	4.26886197	0.26909619	-1.70981530
H	3.79814697	-1.81426884	-0.90555553
H	2.26795888	-2.33253977	-0.18406065
H	3.35638434	-1.24501076	0.71768697

Compound **10**

Energy: -171.00129938870776 eV

Atom	X (Å)	Y (Å)	Z (Å)
C	0.21577415	0.68484103	-2.29504741
C	-1.24236139	0.53580127	-1.84776237
C	-1.90053177	1.92402144	-1.75713588
C	-2.00797612	-0.27986756	-2.91019427
P	-1.60992345	-0.31722623	-0.18688226
C	-1.17578375	0.93048771	1.10342274
C	-0.01147748	1.71884374	1.16033956
C	0.18617551	2.63722859	2.19296342
C	-0.77208031	2.78582449	3.19966795
C	-1.93279644	2.01047653	3.16194811
C	-2.13217405	1.09905520	2.12248177
C	-0.35633944	-1.70212318	0.16048268
C	1.10358794	-1.29897789	0.39267561
C	-0.44389557	-2.72244957	-0.98509678
C	-0.89142908	-2.35842710	1.44861091
H	0.81545972	1.28151741	-1.59655631
H	0.70863590	-0.28724457	-2.42806190
H	0.24241873	1.20039880	-3.26990557
H	-1.36335779	2.58977972	-1.06967356
H	-1.90734197	2.39075350	-2.75507122
H	-2.94243445	1.85050961	-1.41092907
H	-1.59225157	-1.28668781	-3.04341611
H	-1.95139630	0.23768518	-3.88175833
H	-3.06729846	-0.38495138	-2.63753459

H	0.75744718	1.61680832	0.39521805
H	1.09676526	3.23874068	2.21354589
H	-0.61381802	3.50223997	4.00689737
H	-2.68906231	2.11782097	3.94137302
H	-3.04561955	0.50101900	2.09037626
H	1.54852155	-0.79610010	-0.47455484
H	1.70121206	-2.20453954	0.59191512
H	1.20352373	-0.63726922	1.26238497
H	-1.48672073	-2.99813944	-1.19971315
H	0.09748247	-3.63983170	-0.70232018
H	0.01074827	-2.34541018	-1.91102389
H	-0.27393692	-3.23591132	1.70020140
H	-0.85510714	-1.66268322	2.29894332
H	-1.93159837	-2.69219299	1.32529927

Compound **11**

Energy: -322.63023149047433 eV

Atom	X (Å)	Y (Å)	Z (Å)
C	-2.96881198	-1.20197021	-0.03484840
C	-2.20059335	-1.40974173	-1.33882692
C	-2.29567130	-2.84748630	-1.85361870
C	-1.62850157	-3.08385500	-3.21507511
C	-0.13089007	-2.75395729	-3.27699902
C	0.71409141	-3.52645471	-2.26192037
C	2.22664128	-3.35810377	-2.44638173
C	2.74419798	-1.91505002	-2.38519404
P	2.21823607	-0.92362583	-0.89787001
C	3.19011814	-1.76201013	0.45422486
C	2.45613010	-2.91458501	1.15304555
C	1.19367419	-2.50517563	1.92120293
C	1.47184586	-1.66650569	3.17221271
C	0.23004182	-1.39408527	4.02970263
C	-0.76854603	-0.41581400	3.40906544
C	-2.02861766	-0.22309183	4.25440395
C	-3.01100922	0.78378027	3.65699616
C	3.34721941	0.52760438	-1.20704086
C	3.29139077	1.68892955	-0.20559580
C	1.95800041	2.45335355	-0.15621079
C	0.93564877	1.88256335	0.83000658
C	-0.42700533	2.58079332	0.79843629
C	-1.20909457	2.36608385	-0.49955288
C	-2.64202752	2.89625519	-0.43664190
C	-3.42648387	2.66228183	-1.72698564
H	-2.88950796	-0.16574275	0.32248056
H	-4.03787231	-1.43261161	-0.15960068
H	-2.58059361	-1.85725165	0.75989949
H	-2.58680009	-0.72222078	-2.11155640
H	-1.14374896	-1.13555809	-1.18745390
H	-1.86183579	-3.52645215	-1.09999840
H	-3.35951689	-3.12968051	-1.93076584

H	-2.15472679	-2.48631867	-3.97862472
H	-1.77319904	-4.13967903	-3.50059237
H	0.23617676	-2.97311416	-4.29458170
H	0.01425380	-1.67218382	-3.12684562
H	0.43599700	-3.21188009	-1.24284452
H	0.47031217	-4.60049335	-2.32922687
H	2.75011228	-3.96335251	-1.68912029
H	2.51817294	-3.78481542	-3.42191217
H	2.38602324	-1.34798740	-3.25967882
H	3.84672410	-1.91621745	-2.44140213
H	3.43841786	-0.97913484	1.18857785
H	4.14939920	-2.11301151	0.03638489
H	2.19009474	-3.68198662	0.40990054
H	3.15583602	-3.40390063	1.85314724
H	0.65711590	-3.41943750	2.22687693
H	0.51673629	-1.96373075	1.24008646
H	1.93125886	-0.70393179	2.88946551
H	2.22230842	-2.19506163	3.78492140
H	-0.27861172	-2.35236772	4.23868076
H	0.54569622	-0.99984363	5.01043790
H	-0.27258484	0.55929619	3.26505091
H	-1.05827057	-0.75640837	2.40135275
H	-1.73756404	0.10076236	5.26840275
H	-2.52966897	-1.19799857	4.38074351
H	-2.54751462	1.77658423	3.55337412
H	-3.34145711	0.46840221	2.65618493
H	-3.90686768	0.89837283	4.28366381
H	3.08400517	0.90614027	-2.20938907
H	4.38164665	0.14733653	-1.27890424
H	3.54729304	1.33152889	0.80670250
H	4.09573872	2.39073012	-0.48001256
H	2.15311506	3.50362488	0.11792541
H	1.52858744	2.47989100	-1.17153551
H	0.78872141	0.80859366	0.62912415
H	1.35837338	1.95202492	1.84713577
H	-0.29719388	3.66348400	0.97636528
H	-1.03547552	2.20556486	1.63781351
H	-0.68655663	2.84571273	-1.34427222
H	-1.22772471	1.28794244	-0.73243029
H	-3.16608066	2.41666578	0.40815290
H	-2.62084893	3.97472015	-0.20403756
H	-3.49797636	1.58829946	-1.95587924
H	-2.93542964	3.14920153	-2.58312077
H	-4.44979525	3.05796607	-1.65932863

Compound **12**
Energy: -353.27474025217975 eV

Atom	X (Å)	Y (Å)	Z (Å)
C	-3.88491733	0.02123067	2.20696038
C	-2.77392248	-0.83549903	2.83243113

C	-3.08944297	-2.32151516	2.62359402
C	-3.14044495	-2.60278141	1.11689050
C	-4.24913755	-1.75625078	0.47094246
C	-3.95378700	-0.26502784	0.69945832
C	-2.60948744	0.09906570	0.03985818
C	-1.45918266	-0.73824912	0.64172785
P	0.30561593	-0.48633657	-0.08299541
C	0.13682316	-0.30824703	-1.99165300
C	-0.38969879	1.03491209	-2.54397405
C	-0.41164801	1.02155334	-4.08477626
C	1.01192954	0.78671910	-4.61540005
C	1.52495802	-0.57515667	-4.12021969
C	0.60344879	-1.68953209	-4.63104452
C	-0.80585588	-1.45063302	-4.07582366
C	-1.33711319	-0.10013917	-4.57938049
C	-0.76840132	-1.44393272	-2.53567222
C	1.54887789	-0.59252657	-2.58147386
C	0.99087467	1.17572808	0.60395345
C	2.21241551	1.61737904	-0.24336379
C	2.90402966	2.85444821	0.36068076
C	1.89993899	4.01443207	0.43680974
C	0.71374161	3.59705273	1.31882772
C	1.21774880	3.24595689	2.72805537
C	2.23515506	2.09724042	2.63664954
C	3.42218465	2.52825968	1.76655897
C	1.56533275	0.85870436	2.01541734
C	0.01518501	2.36942389	0.70260047
C	-1.42635797	-0.48765622	2.17183262
C	-1.78295777	-2.25227252	0.48208930
H	-3.68218171	1.09171749	2.37995318
H	-4.85294634	-0.20605589	2.68397024
H	-2.69964007	-0.61779273	3.91056177
H	-4.05139681	-2.57882019	3.09783936
H	-2.31466415	-2.94566915	3.09927296
H	-3.34448416	-3.67225724	0.94456425
H	-5.22795109	-2.02038353	0.90517790
H	-4.30741787	-1.96880379	-0.61001131
H	-4.75045922	0.34436924	0.24217666
H	-2.41028368	1.17310294	0.17167568
H	-2.69177212	-0.07333513	-1.04237182
H	-1.40111095	1.23581835	-2.16024155
H	0.24318513	1.86863351	-2.20929998
H	-0.78433805	1.99511311	-4.44322279
H	1.01543437	0.81404112	-5.71796049
H	1.68177899	1.59245449	-4.27025426
H	2.54993892	-0.74070173	-4.49055402
H	0.98023654	-2.67261201	-4.30284084
H	0.58359706	-1.69994662	-5.73375608
H	-1.47847127	-2.26000126	-4.40425531
H	-2.36355214	0.06590101	-4.21078801
H	-1.38471456	-0.09855020	-5.68121262

H	-1.79850584	-1.33214250	-2.17240353
H	-0.39461680	-2.41560865	-2.17392234
H	1.89088362	-1.57600063	-2.22004828
H	2.28378718	0.14078412	-2.23104801
H	2.93482114	0.78730521	-0.30542274
H	1.90878606	1.86609063	-1.26879908
H	3.74701404	3.13530724	-0.29182914
H	1.54571688	4.27759371	-0.57425744
H	2.38819107	4.91178122	0.85233925
H	-0.01172965	4.42470016	1.38075046
H	1.68529962	4.12899303	3.19498996
H	0.37034965	2.95171616	3.37021980
H	2.58973350	1.83656261	3.64730218
H	3.92281773	3.40844667	2.20366849
H	4.16984725	1.71907685	1.71843351
H	2.29747550	0.04004313	1.92273188
H	0.78326055	0.50165184	2.69475731
H	-0.85402201	2.11191039	1.32375587
H	-0.37648078	2.62624103	-0.29302199
H	-0.62760140	-1.09640820	2.62584806
H	-1.20293306	0.56456061	2.39196984
H	-0.98028637	-2.83713879	0.96006442
H	-1.79880008	-2.54891783	-0.57260207

Compound **13**
Energy: -232.0542457069005 eV

Atom	X (Å)	Y (Å)	Z (Å)
C	-2.59044262	-3.40108837	4.55785532
O	-3.41690450	-2.85526638	3.53134024
C	-2.92766629	-1.79442647	2.81989274
C	-1.67308362	-1.20764247	3.03175619
C	-1.26795195	-0.12603033	2.24287633
C	-2.09589270	0.39119521	1.23944301
P	-1.67050773	1.82162855	0.17999053
C	-1.10946203	1.01308708	-1.36308897
C	-0.95090547	-0.36882868	-1.52119170
C	-0.54238381	-0.91727918	-2.74129011
C	-0.28049522	-0.07664052	-3.83122985
O	0.12662003	-0.50944209	-5.06324810
C	0.29543063	-1.91365413	-5.24868738
C	-0.43889816	1.31174770	-3.68901792
C	-0.85615424	1.84100672	-2.47475917
C	-0.09652670	2.39847777	0.91432935
C	1.17224781	2.10372763	0.40130904
C	2.33200787	2.60266360	1.00324438
C	2.23376871	3.41076278	2.14366602
O	3.30268716	3.94516360	2.80904245
C	4.60619581	3.65687650	2.30661001
C	0.96741429	3.71849946	2.66767610
C	-0.17582105	3.22500354	2.05274795

C	-3.36000624	-0.20223146	1.05158290
C	-3.77113307	-1.28386396	1.81947965
H	-2.37201575	-2.65288736	5.33713563
H	-3.16244009	-4.22655682	4.99430081
H	-1.64306992	-3.78690878	4.14744609
H	-1.00309364	-1.58466490	3.80307097
H	-0.28716282	0.31820410	2.41850905
H	-1.14828238	-1.03636137	-0.68120558
H	-0.42895247	-1.99685934	-2.82851368
H	1.06533753	-2.31815415	-4.57161028
H	-0.65087547	-2.45576898	-5.08933555
H	0.61881867	-2.04107921	-6.28708943
H	-0.23710160	1.95282053	-4.54788445
H	-0.98724020	2.92151490	-2.38277444
H	1.26556122	1.47383780	-0.48450844
H	3.30270543	2.35268017	0.57752243
H	5.30436482	4.17199582	2.97467243
H	4.73293842	4.03582947	1.27940746
H	4.81217710	2.57425116	2.32352138
H	0.90672293	4.35327150	3.55239588
H	-1.15427722	3.48279141	2.46426670
H	-4.03195496	0.19209895	0.28596370
H	-4.74697143	-1.74724815	1.67008922

Compound **14**
Energy: -179.71613337224412 eV

Atom	X (Å)	Y (Å)	Z (Å)
C	-3.35382657	-1.26986228	1.27571888
C	-2.85510844	-0.53223370	0.02625009
C	-3.83301347	0.60753404	-0.30581174
C	-2.81708972	-1.51749618	-1.15074454
C	-1.45344924	0.09916631	0.24889357
P	-0.07296605	-1.08467643	0.70579784
C	0.53109419	-0.44640328	2.39655258
C	-0.70178872	-0.40921409	3.31791719
C	1.49075015	-1.50225414	2.97650164
C	1.19886109	0.93196776	2.41020102
C	1.23588777	-0.67731058	-0.61912694
C	2.57316476	-1.31515725	-0.21359865
C	1.46654081	0.79987515	-0.96659410
C	0.72933445	-1.39745248	-1.88488698
H	-2.68230347	-2.09986463	1.53715327
H	-4.35592594	-1.68734238	1.09621990
H	-3.41987950	-0.59564434	2.14140272
H	-3.51177929	1.15584098	-1.20449966
H	-3.89931788	1.32657460	0.52480462
H	-4.84299490	0.21289560	-0.49310097
H	-2.51441698	-1.01406195	-2.08115521
H	-3.81131804	-1.95879401	-1.31578093
H	-2.10763367	-2.33462015	-0.95747950

H	-1.53310810	0.89119175	1.01043729
H	-1.17232404	0.61145185	-0.68322411
H	-1.40978498	0.38254203	3.04056549
H	-0.37138414	-0.21065413	4.35033553
H	-1.24031602	-1.36675301	3.31441882
H	2.43257795	-1.57158441	2.42096186
H	1.02418059	-2.49771303	2.97832687
H	1.73687354	-1.23932086	4.01834727
H	0.56515018	1.70238104	1.94773142
H	2.16647891	0.92682388	1.89200519
H	1.38890290	1.23926691	3.45247378
H	3.06903489	-0.75874125	0.59243861
H	3.25503222	-1.31427105	-1.07955482
H	2.44345150	-2.35751254	0.11187483
H	0.56240171	1.28184935	-1.36220165
H	1.81646667	1.38971677	-0.11258242
H	2.23666055	0.86672636	-1.75353429
H	-0.21919773	-0.97698831	-2.24471823
H	0.57286929	-2.46965861	-1.69986080
H	1.47245528	-1.28952886	-2.69193575

Compound **15**

Energy: -136.54865264983442 eV

Atom	X (Å)	Y (Å)	Z (Å)
C	1.87040889	0.41174359	-2.25690761
P	0.18876847	0.77563419	-1.57202900
C	0.65904934	1.93126269	-0.21942432
C	1.23437025	1.51898154	0.99433275
C	1.59752732	2.45847131	1.96019862
C	1.39541176	3.82293576	1.72951987
C	0.82174216	4.24310111	0.52774703
C	0.45212211	3.30275871	-0.43720023
C	-0.18296881	-0.75104606	-0.63000092
C	0.42286226	-1.98770946	-0.90211624
C	0.03469014	-3.13900290	-0.21048079
C	-0.96414791	-3.07474988	0.76244284
C	-1.57881814	-1.84920044	1.03783762
C	-1.19825370	-0.70180880	0.34312944
H	2.26417252	1.34777068	-2.67477089
H	1.79207763	-0.31339746	-3.07858281
H	2.57095964	0.03662247	-1.49792362
H	1.39033542	0.45573826	1.18586055
H	2.04154310	2.12457377	2.89951918
H	1.68267105	4.55453719	2.48582251
H	0.65678576	5.30567081	0.34199590
H	-0.00520622	3.63252047	-1.37267779
H	1.20725920	-2.06343909	-1.65583315
H	0.51894417	-4.09079424	-0.43511834
H	-1.26522489	-3.97403496	1.30098694
H	-2.36236259	-1.78878140	1.79489530

H	-1.68559580	0.24977229	0.56573517

Compound **16**

Energy: -311.7376613980146 eV

Atom	X (Å)	Y (Å)	Z (Å)
C	3.87255306	1.69877039	2.23916298
C	2.47871303	1.35425379	2.78204936
C	1.88760869	2.61863491	3.43141905
C	2.60655701	0.26529837	3.85899109
C	1.49038870	0.91714792	1.67234292
C	1.73322763	-0.39628830	0.89551911
C	0.37136073	-1.00880135	0.47735184
P	-0.74280057	0.13557339	-0.51049400
C	-1.14813696	-0.93117290	-2.00751362
C	0.04576339	-1.00152359	-2.97808895
C	0.32705483	0.33670198	-3.66931675
C	-0.91570529	0.86484121	-4.39350477
C	-2.11819992	0.94292042	-3.44653257
C	-2.37911815	-0.40730870	-2.76933795
C	-2.36276264	0.02513503	0.43372292
C	-2.26479771	0.77868292	1.77010152
C	-3.61883834	0.84235637	2.48616353
C	-4.19877976	-0.55911173	2.70001707
C	-4.29931300	-1.32246633	1.37596002
C	-2.94821038	-1.37849297	0.64939489
C	2.68376760	-0.21946802	-0.30749576
C	3.65194862	-1.38477016	-0.65058893
C	4.28344912	-1.07786343	-2.01825854
C	4.77284569	-1.47469917	0.39789712
C	2.95147960	-2.75057480	-0.72368287
H	4.50570747	2.10375680	3.04336840
H	4.38183997	0.81722663	1.83218454
H	3.81258244	2.45928481	1.44591933
H	0.89401746	2.41569337	3.85938023
H	2.53741393	2.98608211	4.23982310
H	1.77704608	3.42647188	2.69224307
H	3.08958681	-0.64171042	3.46828983
H	1.61909817	-0.01914759	4.25356258
H	3.21568259	0.62495446	4.70169145
H	0.50690268	0.84012521	2.16298078
H	1.38493856	1.74129799	0.94629736
H	2.18721122	-1.12130069	1.59306434
H	-0.17137605	-1.29739032	1.39153134
H	0.52465836	-1.93385503	-0.09541441
H	-1.35919083	-1.95154386	-1.64433788
H	0.94638093	-1.35913760	-2.45915449
H	-0.18830450	-1.76066894	-3.74646002
H	0.64572930	1.07433911	-2.91369101
H	1.16394377	0.22304063	-4.37622330
H	-0.71068606	1.85149639	-4.83663523

H	-1.16107965	0.18814423	-5.23145809
H	-1.92298280	1.70714324	-2.67484853
H	-3.01728326	1.26606318	-3.99366726
H	-3.25860599	-0.34585463	-2.11159109
H	-2.63156321	-1.15137821	-3.54673708
H	-3.06638831	0.59489109	-0.20161393
H	-1.86213167	1.78978547	1.60402771
H	-1.54442725	0.25982958	2.42706558
H	-4.32205752	1.43868877	1.87855395
H	-3.51291322	1.36695850	3.44872337
H	-3.54330733	-1.11872280	3.39069606
H	-5.18552903	-0.49810921	3.18439384
H	-5.03633327	-0.82030510	0.72459496
H	-4.67709983	-2.34257062	1.54726008
H	-3.06652345	-1.90278953	-0.30999579
H	-2.24316137	-1.98085712	1.24838073
H	3.30904516	0.67249521	-0.15090342
H	2.07776921	0.01237328	-1.19899264
H	5.02360079	-1.84471422	-2.29260325
H	4.79468009	-0.10320509	-2.00534269
H	3.51800944	-1.04591918	-2.80834093
H	5.36983704	-0.55111055	0.41843021
H	4.37206789	-1.64297234	1.40841281
H	5.45272205	-2.30886141	0.16777687
H	2.51326976	-3.03601378	0.24326134
H	2.15278689	-2.76243760	-1.47887102
H	3.67758081	-3.52999193	-0.99977285

Compound **17**
Energy: -219.22064954409535 eV

Atom	X (Å)	Y (Å)	Z (Å)
C	-2.46727810	-2.06497469	-5.15977311
C	-2.11723552	-1.74419672	-3.73153987
C	-3.08215706	-1.22284208	-2.85455436
C	-2.76610465	-0.92821305	-1.53048632
C	-1.46392246	-1.12737022	-1.03760153
P	-1.16301792	-0.72077953	0.72496721
C	0.54621174	-1.33398488	0.98008020
C	0.70458071	-2.70754991	1.23744839
C	1.96778246	-3.25357362	1.45219403
C	3.11795996	-2.44816845	1.43670133
C	4.47834283	-3.04304371	1.68320205
C	2.95243272	-1.07856857	1.19429690
C	1.69031684	-0.52429439	0.96708306
C	-0.92189133	1.09620302	0.67087023
C	-0.88495121	1.85699964	-0.50602755
C	-0.72606878	3.24354556	-0.44791438
C	-0.59412843	3.91517415	0.77440714
C	-0.39767575	5.40625634	0.83173735
C	-0.63933803	3.14879714	1.94992383

C	-0.80870084	1.76695814	1.90168030
C	-0.49786674	-1.65241781	-1.90686382
C	-0.82652639	-1.95570074	-3.23046377
H	-2.80544647	-1.16642276	-5.69784346
H	-3.28695946	-2.79721939	-5.21361789
H	-1.60701894	-2.47913312	-5.70088310
H	-4.09916109	-1.05268790	-3.21615382
H	-3.53967209	-0.53485879	-0.86692453
H	-0.17436735	-3.35566729	1.26672880
H	2.06613278	-4.32507069	1.64248583
H	4.68709275	-3.86505437	0.98234096
H	4.54961552	-3.46250883	2.69845856
H	5.27127315	-2.29234814	1.57298209
H	3.83176038	-0.43017576	1.17803021
H	1.59761212	0.54585614	0.77707932
H	-0.98140753	1.36560584	-1.47505293
H	-0.70201270	3.81799821	-1.37706058
H	-0.60081245	5.87596959	-0.13929423
H	-1.05584833	5.86836890	1.58158521
H	0.63671641	5.65805995	1.11392420
H	-0.54877019	3.64677965	2.91829941
H	-0.85338849	1.19729333	2.83275379
H	0.51848372	-1.82577319	-1.55074686
H	-0.05837285	-2.36381406	-3.89176323

Compound **18**

Energy: -212.2626433417502 eV

Atom	X (Å)	Y (Å)	Z (Å)
C	2.06711261	1.17981438	-3.98600304
C	1.07021515	1.87867583	-3.29986318
C	0.33475149	1.25277711	-2.29142201
C	0.57661492	-0.09058210	-1.94910504
P	-0.31186827	-1.03916293	-0.64804363
C	-1.91641243	-0.09077458	-0.48148173
C	-2.70527054	-0.56554035	0.75437287
C	-3.23345330	-1.99587459	0.60340915
C	-4.08397219	-2.14228890	-0.66286660
C	-3.30998745	-1.69242615	-1.90652907
C	-2.77229288	-0.26547524	-1.75154925
C	0.62007984	-0.53509319	0.89926313
C	0.70315073	0.96650754	1.20176489
C	1.47871393	1.23985380	2.49748994
C	2.87445384	0.61048582	2.46143940
C	2.79319541	-0.89080508	2.16970742
C	2.02279978	-1.16521339	0.87327846
C	1.57526600	-0.78326435	-2.65969334
C	2.31728484	-0.15559504	-3.66170348
H	2.64216161	1.67309006	-4.77070590
H	0.86497022	2.92126970	-3.54913828
H	-0.43454241	1.82349534	-1.77005159

H	-1.69245302	0.98043441	-0.35063862
H	-3.56208324	0.11894446	0.88891295
H	-2.09412238	-0.47230945	1.66520587
H	-3.81787399	-2.27405209	1.49403193
H	-2.38198161	-2.69537437	0.55038128
H	-4.42232232	-3.18341085	-0.77853312
H	-4.99457637	-1.52546393	-0.55926152
H	-3.95096577	-1.75225602	-2.79968791
H	-2.46495160	-2.38060285	-2.07527533
H	-3.62447075	0.43467617	-1.68474511
H	-2.19648932	0.02632275	-2.64291393
H	0.05199839	-1.02555773	1.71285278
H	-0.30442113	1.40355317	1.27366298
H	1.20763833	1.47193647	0.36109632
H	0.91564664	0.82164023	3.35059111
H	1.54913669	2.32502807	2.67062787
H	3.47103257	1.10005257	1.67177958
H	3.40156345	0.78992092	3.41120254
H	3.80247574	-1.32678484	2.10724376
H	2.28374303	-1.39614506	3.00902631
H	1.94382627	-2.24847841	0.69165220
H	2.58527619	-0.74380211	0.02233420
H	1.76565094	-1.83185904	-2.42011517
H	3.08880170	-0.71325691	-4.19531503

Compound **19**

Energy: -335.6995082037173 eV

Atom	X (Å)	Y (Å)	Z (Å)
C	4.79300976	1.01481886	4.62581766
O	3.41383347	0.66033665	4.63069011
C	2.72792809	0.75008805	3.44639466
C	1.37810190	0.38991426	3.50492046
C	0.59380834	0.44125735	2.34929972
O	-0.72229856	0.07852940	2.36814979
C	-1.30426094	-0.29956395	3.60552348
C	1.12948968	0.86181033	1.10825098
P	0.28622394	1.08819133	-0.52316410
C	-1.51222966	0.86211252	-0.20499471
C	-2.38196184	-0.11004117	-0.73233819
O	-1.84339332	-1.04479672	-1.57222437
C	-2.64791741	-2.13292247	-1.99820315
C	-3.75399528	-0.12531142	-0.41899294
C	-4.27839823	0.87003359	0.40663658
O	-5.59709867	0.93412868	0.78030677
C	-6.47439606	-0.07518842	0.29123249
C	-3.46141089	1.89228214	0.90097672
C	-2.10704842	1.88712959	0.57172534
O	-1.24735872	2.86193362	1.00591703
C	-1.73936029	3.83182531	1.92026379
C	0.85987203	-0.41175722	-1.41614197

C	1.33448894	-0.22760603	-2.72737062
O	1.26415678	1.04906138	-3.22282663
C	1.71211180	1.28858616	-4.54922542
C	1.83850371	-1.28914584	-3.50066610
C	1.86753396	-2.56730629	-2.94146852
O	2.33431351	-3.67604921	-3.60071959
C	2.82428871	-3.50165570	-4.92623183
C	1.40233425	-2.79598773	-1.64086714
C	0.90223494	-1.72717201	-0.89543120
O	0.39433001	-1.89328758	0.35758657
C	0.37238132	-3.20046546	0.90987549
C	2.49152326	1.22463902	1.10062841
O	2.98832247	1.62775839	-0.11374384
C	4.36052388	1.98242900	-0.20513502
C	3.30382091	1.16610864	2.24506332
H	4.93762626	2.06719489	4.33034708
H	5.14116217	0.87463827	5.65476412
H	5.37379680	0.36443855	3.95090892
H	0.97672281	0.06723612	4.46225426
H	-0.83394619	-1.20877410	4.01901444
H	-1.23650568	0.50929342	4.35323450
H	-2.35894291	-0.50089464	3.38445115
H	-3.48407189	-1.80034852	-2.63703674
H	-3.04919966	-2.70192122	-1.14199468
H	-1.98112956	-2.77767519	-2.58269030
H	-4.39770817	-0.89741310	-0.82809144
H	-6.52959803	-0.06214475	-0.80990298
H	-6.16239009	-1.07804605	0.62716521
H	-7.46016226	0.15817006	0.70772045
H	-3.90956623	2.66668316	1.51796125
H	-0.87938563	4.46113996	2.17441096
H	-2.52827918	4.45574466	1.46737354
H	-2.13268161	3.35814108	2.83570321
H	2.78268145	1.04965024	-4.66901360
H	1.55953065	2.35960854	-4.72155471
H	1.12807445	0.71414611	-5.28842292
H	2.19390680	-1.11615546	-4.51085001
H	2.03748968	-3.12512305	-5.60083110
H	3.68413499	-2.81185142	-4.95030382
H	3.14457952	-4.49460789	-5.25940085
H	1.43902160	-3.81104406	-1.25316696
H	-0.08111703	-3.09374896	1.90177889
H	1.38871281	-3.61582320	1.01643997
H	-0.23832895	-3.88814488	0.29995918
H	4.60290280	2.84246612	0.44201173
H	5.01791343	1.13513601	0.05414223
H	4.52377941	2.26075679	-1.25203563
H	4.35088795	1.44565365	2.19435844

Compound **20**

Energy: -258.21452681355436 eV

Atom	X (Å)	Y (Å)	Z (Å)
C	6.02289517	-0.04188095	0.22737179
C	5.35766617	-1.12236498	1.09333709
C	3.85286763	-1.02412866	1.06854604
C	3.16352733	-0.23229995	1.99854618
C	1.77636108	-0.10307284	1.94482822
C	1.03109494	-0.74893761	0.94366876
P	-0.78740295	-0.51448025	0.97025066
C	-1.34914432	-1.67751271	-0.33156399
C	-1.68727014	-1.30024591	-1.64001557
C	-2.12336008	-2.25473450	-2.56012865
C	-2.23416786	-3.60873279	-2.21335673
C	-2.64474135	-4.64129741	-3.23311528
C	-1.44107050	-5.20672320	-4.00202284
C	-1.90522234	-3.97825180	-0.90098084
C	-1.47925508	-3.02980792	0.02792488
C	-0.98135105	1.11283913	0.14726227
C	-2.26673793	1.68083987	0.14317788
C	-2.50001209	2.91650610	-0.45889722
C	-1.45742191	3.63655038	-1.06025767
C	-1.71840008	4.94820588	-1.75747241
C	-2.06440282	4.75636865	-3.24180774
C	-0.17300010	3.07495771	-1.03407908
C	0.06768658	1.83357619	-0.44333679
C	1.71417367	-1.55134809	0.01728552
C	3.10188182	-1.68356959	0.08517734
H	5.69948859	-0.12673318	-0.81970276
H	7.11816530	-0.12886865	0.25552097
H	5.75000913	0.96323510	0.57874532
H	5.71547496	-1.03133437	2.13055182
H	5.66674216	-2.11730228	0.73722754
H	3.72385635	0.28296152	2.78276391
H	1.26300078	0.50943018	2.68962656
H	-1.60967738	-0.25487000	-1.94170312
H	-2.38352745	-1.94087685	-3.57432611
H	-3.17660245	-5.46377824	-2.73037150
H	-3.35472039	-4.19260645	-3.94501522
H	-0.72737467	-5.68299175	-3.31502319
H	-0.90678772	-4.40689237	-4.53394656
H	-1.75808103	-5.95653194	-4.74044138
H	-1.99442303	-5.02544877	-0.60122160
H	-1.24249089	-3.34253033	1.04752353
H	-3.09421235	1.14699476	0.61616846
H	-3.50868857	3.33719044	-0.45506198
H	-0.83086394	5.59378270	-1.66974822
H	-2.54455146	5.47430146	-1.25461545
H	-2.96296477	4.13373934	-3.35616673
H	-2.25138862	5.72110262	-3.73402288
H	-1.24303439	4.25467761	-3.77263589
H	0.65746808	3.62179745	-1.48803931
H	1.07822391	1.42278425	-0.44184061

H	1.15890977	-2.07566461	-0.76157520
H	3.61578533	-2.31402482	-0.64503413

Compound **21**

Energy: -196.0746005642775 eV

Atom	X (Å)	Y (Å)	Z (Å)
C	-0.80879600	0.78736038	-4.51722141
C	-0.33241069	1.85793899	-3.75773154
C	0.29750312	1.62149270	-2.53373014
C	0.45253798	0.31421006	-2.04114021
P	1.25167843	0.14147702	-0.39399680
C	1.95407251	-1.54817930	-0.46274693
C	2.71406752	-1.93161699	-1.58399217
C	3.36891164	-3.16291863	-1.61898794
C	3.29317612	-4.03293665	-0.52808805
C	2.56106271	-3.65554177	0.59989965
C	1.90281802	-2.42449479	0.63571096
C	-0.23101470	-0.04641711	0.73398785
C	-0.89746519	1.32894856	0.90984755
C	-2.04543255	1.26845801	1.92433110
C	-3.07569501	0.20096976	1.54257226
C	-2.41264971	-1.16813269	1.36057723
C	-1.26912865	-1.10441149	0.34037508
C	-0.02891267	-0.75522698	-2.81781104
C	-0.65062689	-0.51903461	-4.04452322
H	-1.30003585	0.96827637	-5.47420299
H	-0.44742486	2.88066203	-4.12048179
H	0.67787239	2.46054636	-1.94694362
H	2.78612903	-1.26008145	-2.44130475
H	3.94630679	-3.44204231	-2.50190060
H	3.80842018	-4.99378219	-0.55306096
H	2.50308524	-4.32201825	1.46208508
H	1.34473900	-2.15206481	1.53253420
H	0.20945244	-0.32276453	1.71105691
H	-1.29220988	1.66091281	-0.06606678
H	-0.14759177	2.07445144	1.21636674
H	-1.63373015	1.03490187	2.92194186
H	-2.52595275	2.25569326	2.00706742
H	-3.86984765	0.14608687	2.30298928
H	-3.56421823	0.49273913	0.59643860
H	-3.15556908	-1.91638087	1.04367150
H	-2.01569409	-1.51191585	2.33214484
H	-1.68223379	-0.84164589	-0.64749852
H	-0.79424136	-2.09059941	0.23038032
H	0.08587241	-1.77894268	-2.45917421
H	-1.01872985	-1.36044199	-4.63408775

Compound **22**

Energy: -404.6026269876805 eV

Atom	X (Å)	Y (Å)	Z (Å)
C	2.87381092	-0.46460433	-1.56241412
C	3.59881937	-0.40396095	-0.20973755
C	4.80762877	0.54205471	-0.35293439
C	4.09677694	-1.82301139	0.13334203
C	2.68494560	0.09397600	0.91896052
C	1.34296108	0.40945905	0.70601794
C	0.51766808	0.83384289	1.76480452
P	-1.25655802	1.28133163	1.57099549
C	-1.74192147	0.31940029	0.08738008
C	-1.37084087	-1.03701113	0.00262870
C	-1.76074784	-1.83062478	-1.07688350
C	-1.31689251	-3.29273235	-1.22483059
C	-0.42362902	-3.75082398	-0.06235729
C	-0.51734381	-3.45060050	-2.53453762
C	-2.55830979	-4.20562720	-1.27175085
C	-2.57424560	-1.24764259	-2.06780245
C	-2.99559627	0.08299308	-1.99918480
C	-3.87712654	0.72915558	-3.07739458
C	-5.18428239	1.23443050	-2.43414586
C	-3.12237802	1.92058413	-3.70235670
C	-4.24258190	-0.25106203	-4.20114524
C	-2.57118511	0.85406484	-0.90276889
C	-1.13054596	3.03526938	0.91657544
C	-0.73875411	3.97181537	2.07166741
C	-0.73724656	5.43990827	1.62900883
C	0.16528018	5.65781577	0.41045947
C	-0.22154176	4.72158727	-0.73905887
C	-0.21249490	3.25467425	-0.29209114
C	1.06304531	0.94636266	3.04664101
C	2.41697034	0.65842157	3.29753970
C	2.97418805	0.81847091	4.71834075
C	4.46524383	0.46349629	4.80805340
C	2.19820193	-0.10656046	5.67740618
C	2.79819573	2.28322451	5.16809161
C	3.20124771	0.23174312	2.22290387
H	2.01556281	-1.15145271	-1.53380969
H	3.56575710	-0.82560749	-2.33684399
H	2.50857049	0.52468273	-1.87366815
H	4.47805702	1.56068360	-0.60313681
H	5.47404793	0.18938982	-1.15419833
H	5.39455927	0.59606134	0.57416284
H	4.76254865	-2.19625810	-0.65921374
H	3.25113767	-2.51963556	0.22832716
H	4.65378583	-1.83872186	1.08021340
H	0.91205001	0.31792412	-0.28837164
H	-0.74026244	-1.45657855	0.78466121
H	0.49477086	-3.14948193	0.00646432
H	-0.94704130	-3.68887185	0.90243579
H	-0.12720555	-4.79845454	-0.21542833
H	-1.11600423	-3.17124060	-3.41234615

H	0.38021575	-2.81524227	-2.52304534
H	-0.19686535	-4.49560979	-2.66094377
H	-3.14177064	-4.11826468	-0.34411653
H	-3.21899272	-3.94861601	-2.11118022
H	-2.25410288	-5.25641687	-1.38998781
H	-2.88419113	-1.85730450	-2.91531615
H	-5.82203930	1.71308878	-3.19231495
H	-5.74598155	0.40175479	-1.98715699
H	-4.98841942	1.97110056	-1.64302588
H	-2.86833675	2.67982708	-2.94972054
H	-3.74204815	2.40235691	-4.47349644
H	-2.18637254	1.58585215	-4.17222964
H	-4.80560875	-1.11547928	-3.82046189
H	-3.34990086	-0.62260752	-4.72480904
H	-4.87514271	0.25783636	-4.94247279
H	-2.88715531	1.89566483	-0.83689980
H	-2.16794012	3.29047725	0.62710765
H	-1.42106196	3.82507984	2.92331810
H	0.27024341	3.69698323	2.42544997
H	-0.41958774	6.08454255	2.46339625
H	-1.76850642	5.74158622	1.37429804
H	0.12142154	6.70825960	0.08380668
H	1.21286047	5.45989679	0.69771532
H	0.46080322	4.85835592	-1.59249199
H	-1.23145714	4.98705683	-1.09963051
H	0.81462569	2.96915004	-0.01006351
H	-0.50493339	2.59529442	-1.12274212
H	0.41360623	1.26475615	3.86496218
H	4.81396223	0.59754386	5.84204069
H	4.65294167	-0.58294549	4.52713864
H	5.07794763	1.11054737	4.16374772
H	1.12412903	0.12383316	5.68175062
H	2.57390465	0.00866917	6.70517836
H	2.31594385	-1.15950252	5.38383575
H	1.74197540	2.58536549	5.15734981
H	3.17901596	2.41636500	6.19182003
H	3.35054758	2.96386358	4.50431862
H	4.24989883	-0.00616842	2.39661811

Compound **23**
Energy: -219.21338062996202 eV

Atom	X (Å)	Y (Å)	Z (Å)
C	-0.48418232	0.98535220	-4.14692625
C	-1.30036818	0.09977285	-3.24239615
C	-1.91227907	-1.06387738	-3.72587970
C	-2.65408754	-1.88574161	-2.87312265
C	-2.79624847	-1.55341872	-1.52632671
C	-2.18141529	-0.39691593	-1.01540049
P	-2.43736383	-0.03568672	0.76753590
C	-1.18984137	-1.12539096	1.56214315

C	-1.21870502	-1.21046094	2.96528993
C	-0.30807343	-2.02544593	3.63661576
C	0.62835707	-2.77423295	2.91846151
C	0.66820459	-2.71548569	1.51964263
C	1.67888857	-3.50814504	0.73298400
C	-0.24811344	-1.88432458	0.85601715
C	-1.66381037	1.62184132	0.93851021
C	-2.44247027	2.73069443	0.56377802
C	-1.92482495	4.02068922	0.67670325
C	-0.63493760	4.21976788	1.17595767
C	0.15571886	3.13110042	1.56626119
C	1.55460525	3.33037636	2.08778368
C	-0.37412929	1.83738199	1.44092586
C	-1.44449106	0.41910827	-1.88308977
H	-0.74823225	2.04413837	-4.01308532
H	-0.63088596	0.72514900	-5.20309998
H	0.59060212	0.89166680	-3.92617273
H	-1.81000713	-1.32523780	-4.78114091
H	-3.13010881	-2.78625177	-3.26491995
H	-3.38579541	-2.19239811	-0.86570156
H	-1.95603728	-0.63571448	3.52941613
H	-0.33105345	-2.08521349	4.72604913
H	1.33491433	-3.41582521	3.44899606
H	2.23458458	-4.20229559	1.37633803
H	1.19457162	-4.09151444	-0.06356350
H	2.40921184	-2.84401909	0.24551631
H	-0.22344885	-1.83162830	-0.23451596
H	-3.45499067	2.58015036	0.18374397
H	-2.53175715	4.87855027	0.38222855
H	-0.23744322	5.23253723	1.26899694
H	2.30190309	3.03441664	1.33491758
H	1.73866674	2.71736605	2.98148752
H	1.74144821	4.38066062	2.34616650
H	0.23481946	0.98307440	1.74412782
H	-0.97129365	1.32474890	-1.49778256

Compound **24**
Energy: -179.8505322074688 eV

Atom	X (Å)	Y (Å)	Z (Å)
C	2.28136454	3.62269510	-0.64118760
C	2.44000773	2.33331783	-1.15453370
C	1.42085848	1.38712939	-1.01983304
C	0.22268141	1.72308199	-0.37105039
P	-1.19577358	0.57595257	-0.15801326
C	-0.96551498	-0.00555250	1.56889205
C	0.14347484	0.31102609	2.36846536
C	0.22424809	-0.15912636	3.68167658
C	-0.79425168	-0.95386429	4.21298377
C	-1.90436763	-1.27096136	3.42531789
C	-1.99290074	-0.79272247	2.11733939

C	-0.65926574	-0.88568121	-1.13206566
C	-0.09750018	-2.04454731	-0.57455717
C	0.26161308	-3.11884699	-1.39252163
C	0.07128787	-3.05080851	-2.77469123
C	-0.49119090	-1.90219977	-3.33828541
C	-0.86176751	-0.83272669	-2.52208036
C	0.06594193	3.02768201	0.12837407
C	1.08984453	3.96746641	0.00280370
H	3.08116468	4.35692416	-0.74514823
H	3.36637446	2.05807327	-1.66139553
H	1.55870130	0.38231074	-1.42100545
H	0.94740484	0.92695266	1.96359008
H	1.09248281	0.09513453	4.29192158
H	-0.72513312	-1.32193294	5.23735801
H	-2.70712182	-1.88723021	3.83327231
H	-2.86903991	-1.03358430	1.51104847
H	0.06040679	-2.10799661	0.50277219
H	0.69699651	-4.01394737	-0.94515018
H	0.35684182	-3.89021812	-3.40988390
H	-0.64804097	-1.84193514	-4.41643253
H	-1.31258555	0.05707002	-2.96728999
H	-0.86774149	3.30694088	0.62184437
H	0.95495313	4.97369695	0.40270903

Compound **25**
Energy: -391.6124122870049 eV

Atom	X (Å)	Y (Å)	Z (Å)
C	2.00334671	2.92905934	-5.52135211
C	2.25454861	3.43756579	-4.08713260
C	1.46697596	4.74179015	-3.89639704
C	3.75452243	3.74814856	-3.91060863
C	1.83517255	2.35371527	-3.08570956
C	0.84527789	2.56238998	-2.12110928
C	0.46288644	1.54030528	-1.23311614
P	-0.90892084	1.91044764	-0.06003482
C	-0.63191374	0.63576211	1.22733132
C	0.62306349	0.61893259	1.86931417
C	0.91019099	-0.29770146	2.88105366
C	2.28076120	-0.36442710	3.56933523
C	3.27541433	0.64833707	2.98328201
C	2.87648012	-1.77625804	3.39441723
C	2.11047024	-0.06552927	5.07237615
C	-0.10641723	-1.19219121	3.27030912
C	-1.37088519	-1.18877270	2.67695913
C	-2.47854596	-2.17097310	3.08365126
C	-3.71245871	-1.38095984	3.56427116
C	-2.04111280	-3.11312213	4.21424170
C	-2.86716980	-3.02998217	1.86269660
C	-1.61831276	-0.25787526	1.65110555
C	-2.38131038	1.23939708	-0.99978817

C	-2.73941112	2.20062038	-2.17225056
C	-3.07679696	1.28886582	-3.36390395
C	-2.16126360	0.07691963	-3.15376662
C	-2.25167525	-0.17133427	-1.64114391
C	1.11085424	0.30409986	-1.30205680
C	2.12015337	0.06042268	-2.24660425
C	2.79259100	-1.31916461	-2.28306414
C	1.72553192	-2.39227046	-2.58229711
C	3.88536352	-1.41159065	-3.35689462
C	3.43420295	-1.60987216	-0.91087473
C	2.45859630	1.09211723	-3.12767836
H	2.30491319	3.69239354	-6.25415640
H	0.93763976	2.70608933	-5.67492334
H	2.57294961	2.01375611	-5.73283115
H	1.62546639	5.17061366	-2.89654390
H	0.38735271	4.58937261	-4.03932950
H	1.79964132	5.48508656	-4.63498300
H	4.37540423	2.85653862	-4.07350091
H	3.95871865	4.12077153	-2.89667251
H	4.07143324	4.51723849	-4.63083165
H	0.35202407	3.53091004	-2.04454324
H	1.38770626	1.32168456	1.54018485
H	3.44927673	0.47249713	1.91182944
H	2.92600082	1.68289676	3.11059968
H	4.24185989	0.55701477	3.49944897
H	3.85651294	-1.83803439	3.89078709
H	3.01623022	-2.01236317	2.32976256
H	2.22839246	-2.54908487	3.82990916
H	1.43289677	-0.78451261	5.55331520
H	3.08268946	-0.12064035	5.58474991
H	1.69710763	0.94166786	5.22500583
H	0.10465154	-1.91100792	4.06072129
H	-3.46389108	-0.76400782	4.43963177
H	-4.09706790	-0.71384185	2.78076413
H	-4.52071906	-2.07178293	3.84733727
H	-1.18243851	-3.73339791	3.91914317
H	-1.77133733	-2.55890169	5.12484674
H	-2.86823520	-3.79076422	4.46991159
H	-2.00448019	-3.60899609	1.50288095
H	-3.66770190	-3.73528344	2.13196275
H	-3.22729578	-2.41066658	1.02978878
H	-2.59576242	-0.25429045	1.16914452
H	-3.19787307	1.24967686	-0.26100681
H	-3.55625685	2.88556252	-1.90776322
H	-1.87221311	2.82721108	-2.43192201
H	-4.13084199	0.96967205	-3.31488238
H	-2.92919606	1.78760970	-4.33218726
H	-2.45649959	-0.80121893	-3.74518212
H	-1.12676869	0.33511306	-3.43075663
H	-1.40119926	-0.74252971	-1.24855844
H	-3.15854897	-0.75648742	-1.41741109

H	0.82599515	-0.48014360	-0.60059646
H	1.24855727	-2.20733935	-3.55552960
H	2.18557189	-3.39151529	-2.60749177
H	0.93705751	-2.40161307	-1.81729690
H	4.68707122	-0.67860760	-3.18606922
H	4.33928418	-2.41264719	-3.33631229
H	3.47981178	-1.25165830	-4.36645419
H	2.68781385	-1.59494680	-0.10482501
H	4.20255077	-0.86019259	-0.67227608
H	3.91019869	-2.60197573	-0.91409338
H	3.23316188	0.91706706	-3.87322881

Compound **26**
Energy: -243.69683297091711 eV

Atom	X (Å)	Y (Å)	Z (Å)
C	1.30139636	-2.08029336	2.09194905
C	1.63592168	-1.64181878	0.65820886
C	3.12882344	-1.32103791	0.53964869
C	1.30951000	-2.80290188	-0.30291659
P	0.40701767	-0.28097012	0.15540728
C	1.10501057	0.29068522	-1.46375476
C	2.27689090	1.06467259	-1.56348988
C	2.76641490	1.50996608	-2.78922256
C	2.08056631	1.18275632	-3.96074398
C	0.92252655	0.41189498	-3.88337654
C	0.41318627	-0.04723894	-2.65718375
C	-0.87622786	-0.84104574	-2.64178965
C	-0.93819388	-1.96419368	-3.68751920
C	-2.25216186	-2.74476476	-3.57986092
C	-3.46437503	-1.81729955	-3.71462023
C	-3.41103971	-0.68620015	-2.68288432
C	-2.09552525	0.09287930	-2.77077463
C	0.51805180	1.21531510	1.32828075
C	1.86908085	1.57915125	1.95497969
C	-0.03093151	2.41596339	0.53796593
C	-0.48047747	0.90613345	2.46486130
H	0.22655445	-2.28076110	2.21035388
H	1.84744288	-3.00760309	2.32992716
H	1.59509617	-1.32650007	2.83468872
H	3.40541609	-1.07934203	-0.49436269
H	3.43437166	-0.48495663	1.18052369
H	3.71430328	-2.20428074	0.84624886
H	1.92947704	-3.67741418	-0.04732896
H	0.25284834	-3.09912068	-0.23312106
H	1.52059897	-2.53083796	-1.34690873
H	2.82618228	1.32098077	-0.65870193
H	3.67853234	2.10749916	-2.82876535
H	2.44589523	1.52411133	-4.93042761
H	0.39309100	0.16235495	-4.80451711
H	-0.95479001	-1.31533888	-1.64846175

H	-0.85838109	-1.54298206	-4.70427555
H	-0.07586017	-2.63710470	-3.55934611
H	-2.29265178	-3.25334701	-2.60048603
H	-2.28651825	-3.53553532	-4.34534211
H	-4.39902579	-2.38942113	-3.60851374
H	-3.47488686	-1.38214040	-4.72952726
H	-4.26470813	-0.00318070	-2.81544410
H	-3.50597256	-1.11522783	-1.67000725
H	-2.03924749	0.62680098	-3.73575713
H	-2.04690745	0.86058124	-1.98252723
H	2.62748229	1.85410350	1.21190857
H	2.27176713	0.76406689	2.57058255
H	1.73240390	2.45069449	2.61701414
H	0.65702882	2.73695330	-0.25403960
H	-0.18944840	3.26300180	1.22451315
H	-0.99716119	2.17677393	0.06862853
H	-1.47637176	0.67428489	2.06245196
H	-0.15989271	0.05653892	3.08133087
H	-0.57009716	1.78499982	3.12413516

Compound **27**

Energy: -284.83338673375863 eV

Atom	X (Å)	Y (Å)	Z (Å)
C	0.82357523	-0.59732582	4.68578079
C	1.62518127	-1.50343908	3.98330829
C	1.52490530	-1.57111911	2.59695077
C	0.63661129	-0.74703500	1.87826297
P	0.50034710	-0.88572408	0.03761909
C	2.29869298	-0.95549372	-0.49075377
C	3.11655736	0.16018598	0.18298719
C	4.57253530	0.17215512	-0.29808557
C	4.66883169	0.29263117	-1.82109982
C	3.84714431	-0.80150239	-2.50777092
C	2.39377573	-0.79368630	-2.02109696
C	-0.03623058	-2.69202716	-0.02776981
C	-1.44392489	-2.82591974	0.58406060
C	-2.52530343	-2.19126340	-0.29802976
C	-2.49674899	-2.75775545	-1.72208696
C	-1.10038619	-2.63680701	-2.34381730
C	-0.04069602	-3.28262624	-1.44612605
C	-0.17452781	0.16863190	2.58742828
C	-1.16783286	1.07215431	1.89242591
C	-0.81130062	2.57033889	2.05086522
C	-2.03545551	3.48285928	1.83854258
C	-3.01816101	2.85984069	0.84492167
C	-3.60765688	1.54957659	1.40699473
C	-2.61759224	0.82065694	2.33811906
C	-0.06196312	0.21980232	3.98812902
H	0.88885529	-0.53121929	5.77270251
H	2.32410141	-2.15083141	4.51492082

H	2.15664367	-2.27757464	2.05401738
H	2.73066833	-1.93526352	-0.20877472
H	3.08151163	0.05476613	1.27557434
H	2.64453639	1.13136279	-0.05249876
H	5.11844941	0.99523686	0.18883513
H	5.06442195	-0.76264395	0.02391090
H	4.28392743	1.27994810	-2.13139383
H	5.71977159	0.24895313	-2.14622782
H	3.87639819	-0.67666704	-3.60151173
H	4.29733531	-1.78647762	-2.29141705
H	1.81783557	-1.57218911	-2.53989463
H	1.92137448	0.16619933	-2.29454991
H	0.67724241	-3.26264118	0.59043986
H	-1.46426589	-2.39372029	1.59572930
H	-1.66027570	-3.90335062	0.69863593
H	-3.51606298	-2.34742656	0.15662467
H	-2.36555793	-1.10043296	-0.34001316
H	-2.78550657	-3.82348722	-1.69254247
H	-3.24240271	-2.24850074	-2.35175169
H	-1.08365096	-3.10178692	-3.34172560
H	-0.85740043	-1.56972349	-2.48602705
H	0.95758598	-3.22025866	-1.90251713
H	-0.26260980	-4.36175218	-1.35482147
H	-1.11672507	0.82937675	0.81752285
H	-0.37904360	2.75160132	3.04705921
H	-0.02073170	2.81528107	1.32416450
H	-1.71035116	4.47649258	1.49597531
H	-2.55332073	3.64345124	2.79891002
H	-3.82567161	3.56518190	0.59936810
H	-2.48831562	2.65600134	-0.10033340
H	-3.88883289	0.88727734	0.57367431
H	-4.53636813	1.76160937	1.95919065
H	-2.74593722	1.16936402	3.37627418
H	-2.82700025	-0.25932314	2.34869481
H	-0.68901672	0.92066391	4.54378126

Compound **28**
Energy: -182.1619385011367 eV

Atom	X (Å)	Y (Å)	Z (Å)
F	5.40530984	-3.05628380	-1.30875740
C	4.14374779	-2.53557606	-1.27099575
C	3.10093949	-3.33787014	-0.82001256
C	1.81542496	-2.79684320	-0.79203218
C	1.57514384	-1.46974336	-1.18999112
P	-0.16187681	-0.88323029	-1.12134881
C	-0.33725716	-0.40619092	0.64147380
C	-1.62928954	-0.07381860	1.08591297
C	-1.86564638	0.29090066	2.41148358
C	-0.79058035	0.30044811	3.29393044
F	-1.01093579	0.64931258	4.59540791

C	0.49886697	-0.03684790	2.89866540
C	0.71956534	-0.38907466	1.56518757
C	-0.04997862	0.74871910	-1.95215561
C	-0.08999982	0.75186691	-3.35775134
C	-0.02284230	1.94363681	-4.07972335
C	0.06643158	3.13809473	-3.37271812
F	0.12619684	4.31140814	-4.06834924
C	0.09249330	3.17959621	-1.98338071
C	0.03516823	1.97622667	-1.27657749
C	2.65429714	-0.69515999	-1.64365838
C	3.94613960	-1.22436544	-1.68850792
H	3.29943271	-4.36309809	-0.50826885
H	0.98370765	-3.41817859	-0.45332393
H	-2.46532597	-0.09925282	0.38381582
H	-2.86172263	0.55628593	2.76512981
H	1.31074271	-0.01931116	3.62559916
H	1.72775061	-0.65382870	1.24473003
H	-0.17404221	-0.19350472	-3.89787128
H	-0.04693372	1.95717874	-5.16916842
H	0.15926400	4.13922669	-1.47101773
H	0.05642742	1.99794044	-0.18649862
H	2.48895239	0.33292937	-1.96749647
H	4.79250456	-0.63363123	-2.03852564

Compound **29**

Energy: -492.746773020542 eV

Atom	X (Å)	Y (Å)	Z (Å)
C	-0.15035767	4.83586856	-3.35289306
C	-1.33287343	3.88016360	-3.56663292
C	-1.14730151	3.18044422	-4.92885667
C	-2.62968917	4.71329863	-3.59189606
C	-1.42586943	2.81682023	-2.46442743
C	-0.50002416	2.73151159	-1.42113679
C	-0.58877059	1.71570731	-0.45291712
P	0.75382934	1.62692684	0.80265427
C	0.11259272	0.27663613	1.87591475
C	0.48606009	-1.05933959	1.72870558
C	-0.08893965	-2.06599226	2.52268685
C	0.33875329	-3.52307505	2.29987391
C	1.86142107	-3.64651659	2.51227847
C	-0.36385507	-4.49380705	3.25895206
C	-0.00654027	-3.93285843	0.85276556
C	-1.05036819	-1.69125847	3.46521434
C	-1.44574638	-0.35105775	3.64696931
C	-2.52365558	-0.01604674	4.68622446
C	-2.81569731	1.48973062	4.75495580
C	-2.05902753	-0.48299105	6.08001585
C	-3.83109662	-0.74402046	4.31182148
C	-0.84106863	0.62392453	2.84901652
C	2.03459052	0.71084586	-0.14983255

C	1.80145778	0.15933797	-1.40971187
C	2.78840263	-0.59502846	-2.06642475
C	2.46828082	-1.20184725	-3.43933219
C	3.65611086	-1.97295740	-4.03107584
C	1.27955737	-2.17500483	-3.29345844
C	2.08359596	-0.07472995	-4.41941129
C	4.01303232	-0.78098713	-1.41937802
C	4.28622746	-0.23223616	-0.15057832
C	5.64574250	-0.49686462	0.50964157
C	5.77021999	0.18963054	1.87733921
C	6.77280275	0.03012616	-0.40064412
C	5.82108962	-2.01566244	0.71483240
C	3.28555720	0.52470073	0.46469714
C	-1.63909380	0.79986770	-0.51512702
C	-2.59769582	0.86257629	-1.54062098
C	-3.71672494	-0.18724019	-1.57654834
C	-4.68699593	0.02959927	-2.74584771
C	-4.51873478	-0.12356599	-0.26063866
C	-3.08742504	-1.58895998	-1.71948576
C	-2.46830002	1.86942430	-2.50110847
H	-0.12840635	5.58215626	-4.15981591
H	-0.23230563	5.37678456	-2.39924950
H	0.81094862	4.30230585	-3.36586927
H	-0.22222345	2.58595147	-4.93939674
H	-1.98444663	2.50578375	-5.15512226
H	-1.08547130	3.92678498	-5.73497917
H	-2.78198058	5.22720035	-2.63208184
H	-3.51211596	4.08748621	-3.78310864
H	-2.57712007	5.47371279	-4.38528455
H	0.32256209	3.44410628	-1.36101093
H	1.22246032	-1.32264704	0.96971282
H	2.18600101	-4.68441663	2.34466922
H	2.42027422	-3.00147255	1.82042145
H	2.13657794	-3.36083635	3.53774115
H	-0.01713001	-5.51902707	3.06598319
H	-1.45522164	-4.48184864	3.12474387
H	-0.14203070	-4.25905833	4.31007458
H	0.29940770	-4.97356098	0.66815313
H	0.50355925	-3.29379369	0.11892294
H	-1.08870405	-3.85596330	0.67266316
H	-1.51542324	-2.46033270	4.08058244
H	-3.58743555	1.68177449	5.51394764
H	-3.18837358	1.87551829	3.79515554
H	-1.92143211	2.06489805	5.03456482
H	-2.82831512	-0.25565894	6.83299394
H	-1.87205935	-1.56526491	6.10529999
H	-1.12998950	0.02687115	6.37231700
H	-3.69417993	-1.83375157	4.28200867
H	-4.18812049	-0.42139954	3.32301287
H	-4.61574782	-0.52088475	5.05018410
H	-1.12299151	1.67114210	2.95809271

H	0.82838174	0.30312563	-1.87881047
H	3.95094386	-2.81974195	-3.39449195
H	4.53278233	-1.32419074	-4.17157476
H	3.37991446	-2.37810675	-5.01499564
H	0.38505237	-1.66559354	-2.90934299
H	1.52666589	-2.99194584	-2.59992132
H	1.02675473	-2.61693851	-4.26893547
H	2.91407682	0.63517797	-4.54289013
H	1.20820742	0.48759089	-4.06647980
H	1.84018065	-0.49565106	-5.40647046
H	4.78487528	-1.37376448	-1.90856669
H	5.66876163	1.28127144	1.79498678
H	6.75903180	-0.02268280	2.30828520
H	5.01236416	-0.17527344	2.58546455
H	6.67279202	1.11360679	-0.55756331
H	7.75370743	-0.16227692	0.05901338
H	6.76116584	-0.45580144	-1.38586694
H	6.79498094	-2.22683139	1.18131168
H	5.03279561	-2.41452837	1.36965552
H	5.77640678	-2.56117258	-0.23783260
H	3.45848540	0.96287587	1.44762636
H	-1.70017185	0.01237736	0.23572933
H	-4.17800603	-0.04554625	-3.71768802
H	-5.18020425	1.01073684	-2.68878672
H	-5.47175315	-0.73987925	-2.72202601
H	-3.87931500	-0.31324401	0.61242116
H	-5.31835413	-0.87940122	-0.26546275
H	-4.98072773	0.86593101	-0.13286194
H	-3.87308692	-2.35909297	-1.73943689
H	-2.50883104	-1.66420936	-2.65169256
H	-2.41122246	-1.81578413	-0.88378300
H	-3.19509713	1.92456744	-3.31051942

Compound **30**
Energy: -356.3814571345262 eV

Atom	X (Å)	Y (Å)	Z (Å)
C	0.93417045	-3.61269032	-2.51828167
C	0.35363410	-3.83054527	-1.13295317
C	-0.28228923	-5.20339238	-0.95228737
O	-0.65800009	-2.80073940	-0.91795807
C	-0.77362956	-2.36299554	0.38600574
C	-1.85975524	-2.79451138	1.15352698
C	-1.96756776	-2.37535107	2.47785716
C	-0.99332449	-1.55702524	3.05407599
C	0.08912902	-1.11908841	2.27740703
O	1.10349530	-0.32573073	2.74567253
C	1.17519424	-0.00338553	4.15286869
C	2.64554328	0.26829871	4.43376727
C	0.29316656	1.19462893	4.48540390
C	0.18672669	-1.47755423	0.91286498

C	1.29788084	-0.97196462	0.05998148
C	2.60643092	-1.40084421	0.33199861
C	3.67985700	-1.00830737	-0.46488587
C	3.45141398	-0.16430168	-1.55534272
C	2.16118516	0.29145051	-1.82098228
C	1.06300997	-0.08923687	-1.02483779
P	-0.65493977	0.51925640	-1.32388174
C	-0.61703906	2.22855174	-0.54644766
C	0.55109371	3.14984256	-0.91710150
C	0.42629220	4.52188364	-0.24049818
C	0.28515111	4.39085390	1.27931498
C	-0.88563062	3.47318572	1.64369167
C	-0.73874762	2.10019337	0.97974260
C	-0.62925868	0.94630663	-3.15038608
C	-1.93316724	1.66557298	-3.55120967
C	-3.15901405	0.74973754	-3.47338764
C	-2.96508948	-0.50618003	-4.32953229
C	-1.67876320	-1.24457406	-3.94583342
C	-0.45609059	-0.32285257	-4.00585212
H	0.13684447	-3.64639845	-3.27383718
H	1.43776710	-2.63979172	-2.57993018
H	1.66450841	-4.40096511	-2.74967559
H	1.14602447	-3.68896949	-0.37908602
H	-0.70607458	-5.30616809	0.05555390
H	-1.08581148	-5.35075144	-1.68816695
H	0.46752224	-5.99551148	-1.09245196
H	-2.59512301	-3.45669428	0.69873774
H	-2.81468255	-2.70111394	3.08273463
H	-1.09053805	-1.26029598	4.09572206
H	0.85287484	-0.88812542	4.72872823
H	2.99859006	1.10546501	3.81521196
H	3.25682061	-0.61389336	4.20429355
H	2.78907833	0.53129122	5.49060158
H	0.66389954	2.08852092	3.96489458
H	-0.74875619	1.02883589	4.18324818
H	0.31416721	1.39075110	5.56679891
H	2.77180301	-2.06086922	1.18511483
H	4.68707469	-1.36140150	-0.23955469
H	4.27950792	0.14891281	-2.19307878
H	2.00997772	0.96524853	-2.66464848
H	-1.55477414	2.68752439	-0.91417416
H	1.49305531	2.67123795	-0.60089766
H	0.61611462	3.27900771	-2.00853361
H	1.29701916	5.14649742	-0.49435443
H	-0.46140635	5.04309006	-0.64049438
H	0.15811961	5.38257627	1.74046840
H	1.21764938	3.96652911	1.69252139
H	-0.96376212	3.36187268	2.73634643
H	-1.82945065	3.93931230	1.30929470
H	-1.58639503	1.44592579	1.23582888
H	0.16513060	1.60245619	1.36753475

H	0.20957718	1.63048169	-3.35822595
H	-1.81459922	2.01557483	-4.59257904
H	-2.08560851	2.56977877	-2.94268952
H	-4.05748125	1.30005185	-3.79337618
H	-3.32326085	0.45070944	-2.42434187
H	-3.83333159	-1.17553036	-4.22931280
H	-2.91504682	-0.21222273	-5.39346208
H	-1.77350693	-1.64970956	-2.92579017
H	-1.52227524	-2.10653742	-4.61382437
H	-0.29238367	-0.00553029	-5.05180757
H	0.44706976	-0.86846081	-3.69725655

Compound **31**
Energy: -421.9699586730513 eV

Atom	X (Å)	Y (Å)	Z (Å)
C	2.51230757	-2.61784548	1.95528590
C	2.10734448	-1.27898228	1.32565175
N	0.81003183	-0.77596220	1.76821368
C	0.75496137	-0.23910497	3.11198145
P	-0.50876740	-0.96500824	0.70931020
C	-1.33574890	0.74626432	0.82433949
C	-1.75957535	1.28405760	-0.54485523
C	-2.09175926	2.77721982	-0.48058091
C	-3.18099681	3.06011091	0.55691321
C	-2.79149103	2.49328214	1.92485497
C	-2.45455190	0.99629762	1.84909727
C	-1.67428760	-2.00091129	1.80864463
C	-1.00207635	-3.24681432	2.40287624
C	-1.09078291	-4.47844214	1.50714171
C	-2.55612251	-4.81746586	1.22034336
C	-3.29863949	-3.64040073	0.57978794
C	-3.10790028	-2.31504784	1.33235041
C	2.13019279	-1.31070114	-0.17549344
C	2.39722547	-2.43379390	-1.01669476
C	2.50345511	-1.96396767	-2.34993975
C	2.26987069	-0.56685012	-2.35965103
C	2.04261852	-0.12833320	-1.01963066
P	1.85760820	1.55747557	-0.36451646
C	3.50452548	1.79551937	0.40581338
C	3.58362549	2.62799874	1.53435848
C	4.80332743	2.83030565	2.18654844
C	5.95634968	2.19321367	1.72215415
C	5.88519056	1.35552842	0.60328655
C	4.66944920	1.15835322	-0.05282718
C	1.96071530	2.58090332	-1.88436754
C	2.87405052	3.63995452	-1.99813208
C	2.84143792	4.49002553	-3.10732750
C	1.89696507	4.29572054	-4.11770545
C	0.97974382	3.24635804	-4.01103277
C	1.00481637	2.40284171	-2.90004206

Fe	0.41214147	-1.51468280	-1.39496452
C	-1.00745554	-3.05588526	-1.96567876
C	-0.13771227	-3.00386113	-3.08817716
C	-0.23121549	-1.71117969	-3.66833871
C	-1.16211055	-0.94950669	-2.91525769
C	-1.66593929	-1.78134565	-1.86565713
H	1.86804311	-3.43416841	1.60180573
H	3.55219658	-2.86234999	1.69549361
H	2.44262480	-2.56757991	3.05044351
H	2.85880269	-0.52755311	1.63820028
H	1.60750727	0.44364974	3.27426294
H	-0.16247320	0.34136008	3.26110940
H	0.78932965	-1.02047612	3.89421103
H	-0.46090095	1.35001652	1.12475920
H	-2.64004250	0.73102662	-0.91360839
H	-0.94886173	1.09970503	-1.26005383
H	-2.40084552	3.13515768	-1.47527865
H	-1.17531145	3.33361222	-0.21760996
H	-3.37469802	4.14102411	0.63109628
H	-4.12521467	2.59179261	0.22718903
H	-1.91141673	3.03977852	2.30664273
H	-3.60208685	2.65246549	2.65306102
H	-2.17880333	0.64414972	2.85462125
H	-3.36211121	0.44130908	1.56280296
H	-1.78562409	-1.29783774	2.65102524
H	0.03781495	-3.02329467	2.66876183
H	-1.52080686	-3.47606726	3.35175293
H	-0.54309114	-4.28737458	0.56922638
H	-0.59175562	-5.33120805	1.99356581
H	-3.04369351	-5.08375065	2.17471874
H	-2.63406554	-5.70491495	0.57332033
H	-2.97827342	-3.53447992	-0.46316555
H	-4.37677072	-3.86145883	0.53486982
H	-3.51823280	-1.49021165	0.72941829
H	-3.73473808	-2.34807646	2.24122090
H	2.50738113	-3.46363547	-0.69281170
H	2.63902864	-2.58249393	-3.23178078
H	2.23796321	0.06639787	-3.23982722
H	2.67967626	3.11790062	1.90420870
H	4.85103392	3.48160179	3.06055763
H	6.90890986	2.34536466	2.23126828
H	6.78324731	0.85264332	0.24070157
H	4.61563510	0.49568673	-0.91898759
H	3.61971048	3.79845117	-1.21786370
H	3.56078319	5.30712703	-3.18042715
H	1.87195711	4.96054849	-4.98188189
H	0.23601828	3.08650259	-4.79347972
H	0.28168110	1.58864541	-2.82730252
H	-1.17870855	-3.92898400	-1.34745014
H	0.53090032	-3.79779185	-3.40723937
H	0.36896810	-1.34365170	-4.49597648

H	-1.46703675	0.07149846	-3.12161104
H	-2.47341882	-1.52404353	-1.18842167

Compound **32**

Energy: -178.01017516397113 eV

Atom	X (Å)	Y (Å)	Z (Å)
Cl	-3.59627922	-5.21282597	-0.17529296
C	-2.49091868	-3.89470033	-0.38364336
C	-2.98159947	-2.59149343	-0.48991825
C	-2.08829356	-1.53141102	-0.65186711
C	-0.70504093	-1.75885230	-0.71242931
P	0.55191618	-0.44454986	-0.95046869
C	1.13195660	-0.15166848	0.76461995
C	2.24066538	0.69645290	0.92855280
C	2.75932890	0.97109333	2.19299972
C	2.17397136	0.37548380	3.31326555
Cl	2.81349090	0.70344570	4.89004222
C	1.08149528	-0.48355638	3.17558219
C	0.56536848	-0.74146518	1.90472468
C	-0.48955576	1.03273044	-1.26175292
C	-0.80932122	1.99560039	-0.29250570
C	-1.59109753	3.10543700	-0.61662537
C	-2.06334679	3.25683176	-1.92218328
Cl	-3.04003127	4.62978926	-2.32693832
C	-1.75410472	2.31489376	-2.90681161
C	-0.96348148	1.21661189	-2.57210647
C	-0.23771729	-3.08113123	-0.61942405
C	-1.11824885	-4.14796910	-0.44669593
H	-4.05523371	-2.41113312	-0.44250043
H	-2.47898984	-0.51626714	-0.73136625
H	2.70752394	1.15287697	0.05301164
H	3.61495662	1.63464129	2.31520323
H	0.63862599	-0.94295129	4.05893137
H	-0.28958787	-1.41130176	1.80564766
H	-0.44639368	1.88315874	0.72979759
H	-1.83793419	3.85096773	0.13884804
H	-2.12452336	2.44934141	-3.92270601
H	-0.71232769	0.48815644	-3.34616146
H	0.83399236	-3.28246422	-0.68110798
H	-0.75035895	-5.17062624	-0.36823370

Compound **33**

Energy: -236.32110993710234 eV

Atom	X (Å)	Y (Å)	Z (Å)
C	-4.59859679	-1.75202546	0.25155535
C	-3.98325553	-1.59286270	1.49530305
C	-2.62998141	-1.89554105	1.65484910
C	-1.86958631	-2.36083832	0.56929506

C	-0.42658502	-2.69128436	0.72005715
C	0.00208933	-3.98897617	0.40214876
C	1.33938720	-4.36380167	0.53700496
C	2.27508628	-3.42920233	0.98358777
C	1.87216619	-2.12518175	1.27678657
C	0.53044517	-1.73605552	1.14510536
P	-0.05320379	-0.02764912	1.51004621
C	-0.56952166	0.51148752	-0.16666858
C	-1.61855048	1.43837303	-0.25123337
C	-2.08391158	1.87671410	-1.49342542
C	-1.50872867	1.38691706	-2.66750100
C	-0.46742214	0.45619447	-2.59345357
C	0.00037097	0.02055141	-1.35326132
C	1.51173989	0.88832898	1.78274089
C	2.09800342	1.74810695	0.84091201
C	3.25969914	2.45623823	1.15818756
C	3.85219985	2.31826665	2.41531614
C	3.26864760	1.47455524	3.36468164
C	2.10227176	0.77459693	3.05437852
C	-2.50328135	-2.53528920	-0.67095088
C	-3.85498764	-2.22808554	-0.83119766
H	-5.65431827	-1.50785505	0.12684914
H	-4.55891004	-1.23079748	2.34838239
H	-2.15486062	-1.77525614	2.62833737
H	-0.73845909	-4.71702600	0.06658849
H	1.64588329	-5.38322660	0.29896329
H	3.32388479	-3.70891635	1.09326001
H	2.61529552	-1.39297556	1.59377136
H	-2.08101203	1.80773250	0.66634724
H	-2.90154432	2.59742336	-1.54386969
H	-1.87208240	1.72524581	-3.63878246
H	-0.01814171	0.06691560	-3.50860943
H	0.80439454	-0.71581448	-1.30438286
H	1.64273892	1.86836559	-0.14261470
H	3.70369325	3.12173667	0.41600629
H	4.76000035	2.87206734	2.65787396
H	3.71747291	1.36964510	4.35371015
H	1.63969252	0.13166399	3.80690931
H	-1.91796297	-2.88890213	-1.52115040
H	-4.32659318	-2.35216454	-1.80707890

Compound **34**
Energy: -211.62243559405496 eV

Atom	X (Å)	Y (Å)	Z (Å)
C	0.30136998	2.86593141	-3.50440961
C	-1.05681169	2.23103617	-3.16331267
C	-0.92969710	0.71310994	-3.00357100
C	0.07369041	0.32740303	-1.88044738
P	-0.75826309	-0.43955308	-0.37679033
C	0.77445702	-0.68429764	0.73975760

C	1.92798056	-1.40461885	0.02181043
C	1.59094214	-2.87173130	-0.26249741
C	1.19062685	-3.58810524	1.03095613
C	0.07687809	-2.82130272	1.74215528
N	0.49448435	-1.43324117	1.97258887
C	-1.50546485	1.13051957	0.42252335
C	-2.91472116	1.39710938	-0.13005887
C	-3.95847270	0.43676104	0.45031087
C	-3.93070320	0.46246834	1.98043637
C	-2.51386011	0.18065719	2.47657212
N	-1.58412462	1.14261979	1.88245615
N	0.91368877	1.49144483	-1.56120755
C	1.44074679	2.12634420	-2.77226415
H	0.29060057	3.92804272	-3.22055507
H	0.48275912	2.82731276	-4.58953567
H	-1.43651740	2.66886548	-2.23013219
H	-1.79479518	2.46459222	-3.94495751
H	-0.57600790	0.28637907	-3.95610652
H	-1.91000676	0.25101900	-2.81488343
H	0.69072147	-0.50562254	-2.28392942
H	1.11888030	0.31901161	1.04832615
H	2.20822467	-0.88565866	-0.90606181
H	2.80232226	-1.35794202	0.69242867
H	0.75624649	-2.92390565	-0.98258667
H	2.45048760	-3.37265468	-0.73296752
H	0.85753656	-4.61562787	0.82070394
H	2.06381109	-3.65306385	1.70053430
H	-0.84941404	-2.88683743	1.13228804
H	-0.13744599	-3.27333548	2.72194883
H	-0.20524619	-0.95146547	2.53381034
H	-0.82754511	1.94422929	0.12023393
H	-3.18389738	2.43297121	0.13679074
H	-2.91491321	1.33674985	-1.22634683
H	-4.95997522	0.70010643	0.07738034
H	-3.74341897	-0.58678540	0.09910530
H	-4.62544614	-0.28203896	2.39810511
H	-4.24551103	1.45431470	2.34323589
H	-2.26984161	-0.87695775	2.22992253
H	-2.45430418	0.28407080	3.57038722
H	-0.66446812	1.12090428	2.31798363
H	1.67480903	1.21958425	-0.94208965
H	1.90039697	1.38022672	-3.45655791
H	2.23714562	2.82594470	-2.48207936

Compound **35**
Energy: -303.7010227809207 eV

Atom	X (Å)	Y (Å)	Z (Å)
C	-1.97912641	-3.55545486	-0.95244906
O	-0.77366369	-2.99058381	-0.44818029
C	0.04250376	-2.34717587	-1.33872685

C	-0.27600369	-2.17250225	-2.69295885
C	0.63935139	-1.52961920	-3.52566721
C	1.86670897	-1.07627895	-3.04414853
C	2.17457549	-1.26191324	-1.68852599
O	3.36346923	-0.87538790	-1.12515929
C	4.27509727	-0.14863518	-1.94037862
C	1.25607330	-1.86574414	-0.80858618
C	1.59106969	-2.06983922	0.62944453
C	2.51989284	-3.06594077	0.96155926
C	2.85241440	-3.33228852	2.28835320
C	2.24737675	-2.59324983	3.30852205
C	1.33435467	-1.58957657	2.98953811
C	0.99257273	-1.29821673	1.65435923
P	-0.19690777	0.03033842	1.17261843
C	-1.35218746	0.06572788	2.64854412
C	-2.22177983	-1.20664995	2.65475911
C	-3.24966492	-1.21468449	1.51806214
C	-4.12361710	0.04310979	1.52696096
C	-3.26151904	1.30894126	1.48455678
C	-2.24665958	1.32006905	2.63175839
C	0.80507484	1.59674727	1.42259665
C	1.43595000	1.82473498	2.80164016
C	2.19908514	3.15519074	2.85816808
C	3.25916476	3.24157036	1.75561360
C	2.63599775	3.01502547	0.37467315
C	1.87148408	1.68831154	0.32038375
H	-1.77428748	-4.30768395	-1.73187432
H	-2.65043786	-2.78143421	-1.35916734
H	-2.46176249	-4.03696236	-0.09520498
H	-1.21901510	-2.53180363	-3.09752155
H	0.39254967	-1.38463446	-4.57801865
H	2.57054255	-0.59339346	-3.71788004
H	4.63380352	-0.75441434	-2.78879647
H	3.81929350	0.77984521	-2.32309957
H	5.12060189	0.10128549	-1.28998448
H	2.97933155	-3.64034617	0.15551178
H	3.57439887	-4.11498955	2.52531972
H	2.49102168	-2.79290309	4.35327270
H	0.88349946	-1.01977158	3.80244959
H	-0.75945056	0.09017680	3.57645183
H	-1.58228997	-2.10063541	2.59557978
H	-2.75204867	-1.25600764	3.62277859
H	-2.71206689	-1.26971740	0.55797403
H	-3.87562023	-2.11839150	1.58811801
H	-4.73851603	0.05543335	2.44452901
H	-4.82595192	0.02755114	0.67914527
H	-2.72453238	1.34842896	0.52127203
H	-3.89359901	2.20901452	1.53410789
H	-1.63614118	2.23532882	2.60073497
H	-2.79560157	1.35256151	3.59030771
H	0.06530525	2.40008428	1.24015122

H	0.66917491	1.80084164	3.59130197
H	2.13424954	0.99788574	3.01309564
H	2.66323312	3.28042865	3.84893148
H	1.48397603	3.98835433	2.73832549
H	3.77389466	4.21421970	1.79234138
H	4.02792598	2.46929028	1.93460701
H	1.94475965	3.84587420	0.14754673
H	3.41468015	3.03402389	-0.40492002
H	2.57939373	0.85353097	0.44684853
H	1.40324128	1.54700450	-0.66656394

Compound **36**
Energy: -279.49988014308695 eV

Atom	X (Å)	Y (Å)	Z (Å)
C	-2.42499855	-1.35804800	1.95377182
C	-2.04628671	-2.06302358	0.64143114
C	-2.41900512	-3.55052177	0.75934092
C	-2.85749226	-1.44081612	-0.50442262
C	-0.51617119	-1.98921701	0.39522910
C	0.13021326	-0.60412613	0.16658624
C	0.12064921	-0.23992237	-1.33330129
P	0.46307664	1.56780174	-1.61041461
C	-1.15602478	2.26700839	-1.08493361
C	-2.34030715	2.10834451	-1.82555388
C	-3.55138410	2.60424388	-1.34173385
C	-3.60135570	3.26501562	-0.10976534
C	-2.42957933	3.43746446	0.62967147
C	-1.21517063	2.94732842	0.14141675
C	0.32677620	1.71135418	-3.43140336
C	0.55711698	0.64041518	-4.31056089
C	0.52585463	0.83418133	-5.69440326
C	0.26744083	2.09904879	-6.22550171
C	0.04285871	3.17442815	-5.36059845
C	0.07955593	2.98395913	-3.97980732
C	1.58574648	-0.54287137	0.68295833
C	1.85796756	-0.15028450	2.15580993
C	3.38423948	-0.15124362	2.34826052
C	1.32878134	1.26380954	2.44206904
C	1.23789482	-1.13361661	3.15816573
H	-3.50985999	-1.42619155	2.12442548
H	-2.16281099	-0.29034070	1.93741741
H	-1.92165472	-1.82259648	2.81453753
H	-1.85980179	-4.03499860	1.57422833
H	-2.19281019	-4.08989626	-0.17289502
H	-3.49277518	-3.67093979	0.96735393
H	-2.69277945	-0.35793183	-0.58405395
H	-2.60489759	-1.90331491	-1.47064387
H	-3.93337102	-1.59633500	-0.33352285
H	-0.03230680	-2.46945446	1.25914928
H	-0.27522040	-2.63152110	-0.47113253

H	-0.46741391	0.15225569	0.70145829
H	0.92069545	-0.81119052	-1.83423056
H	-0.82727400	-0.51480371	-1.81416825
H	-2.31073197	1.59232323	-2.78721799
H	-4.46331842	2.47062639	-1.92602594
H	-4.55061353	3.64578030	0.26930349
H	-2.45836130	3.95694514	1.58889771
H	-0.30010529	3.08774949	0.71907021
H	0.75966847	-0.35867481	-3.92390086
H	0.70529423	-0.01184117	-6.35991407
H	0.24464316	2.24748429	-7.30568761
H	-0.15747811	4.16820785	-5.76431086
H	-0.09780300	3.83144121	-3.31438734
H	2.13256732	0.19253248	0.06794042
H	2.06779217	-1.51752201	0.48472219
H	3.86879485	0.55657776	1.65898315
H	3.65043920	0.13868761	3.37572395
H	3.80580573	-1.15004295	2.15859375
H	1.74143710	1.98929251	1.72489281
H	1.61502415	1.58448242	3.45496943
H	0.23240788	1.30906389	2.37949818
H	0.14164995	-1.12349044	3.11277993
H	1.58205459	-2.16255655	2.97377722
H	1.52842702	-0.86366089	4.18482689

Compound **37**
Energy: -385.7863052134426 eV

Atom	X (Å)	Y (Å)	Z (Å)
C	4.15722930	-1.80255611	-4.57613409
C	3.66889977	-0.54951756	-3.83541256
C	2.79692542	0.32560094	-4.74803289
C	2.94485198	-0.90198819	-2.54871727
C	1.78109339	-1.67706356	-2.55852299
C	1.09267078	-1.99626640	-1.38447989
C	-0.13933405	-2.88786284	-1.47034094
C	-1.16705231	-2.37674681	-2.48784364
C	0.26991337	-4.33814172	-1.77454016
C	1.58545288	-1.51944415	-0.14617171
C	0.87864757	-1.90676031	1.11832762
C	1.36072860	-3.00001506	1.85383294
C	0.71501474	-3.44651943	3.00722788
C	-0.44595263	-2.80024971	3.43686301
C	-0.94454581	-1.71766038	2.71263033
C	-0.29552767	-1.24332072	1.55744888
P	-0.94201013	0.11935954	0.50061175
C	0.01399858	1.59811396	1.14484915
C	-0.48723060	2.88802562	0.47404063
C	0.41885476	4.07828978	0.81030232
C	0.55906866	4.25966542	2.32518869
C	1.07621499	2.97957101	2.98990750

C	0.18722043	1.77287167	2.66019080
C	-2.73733592	0.28413413	1.02847060
C	-3.53938879	0.82796856	-0.17324527
C	-5.04911422	0.79544834	0.08647177
C	-5.41546797	1.54407286	1.37086273
C	-4.62541395	1.00201772	2.56537037
C	-3.11382532	1.05017770	2.30631275
C	2.76722539	-0.74539410	-0.11004325
C	3.36580266	-0.25249471	1.20221233
C	4.45925789	-1.21562703	1.69665216
C	3.93497135	1.16929228	1.11271629
C	3.41866416	-0.45043104	-1.31573104
H	4.80459172	-2.41602866	-3.93433298
H	3.30922061	-2.42816986	-4.89207341
H	4.72411030	-1.52782704	-5.47775424
H	4.55668616	0.04055468	-3.55233844
H	2.47691017	1.24107212	-4.23138260
H	1.89162089	-0.21567976	-5.06071591
H	3.34647827	0.61426390	-5.65607878
H	1.39517204	-2.04674157	-3.51068813
H	-0.62596153	-2.88344453	-0.48465340
H	-1.45575357	-1.34160155	-2.26046341
H	-0.77309420	-2.40461743	-3.51462694
H	-2.06992373	-3.00491531	-2.46549037
H	-0.61305401	-4.99376185	-1.80892712
H	0.95530580	-4.72465468	-1.00736720
H	0.78116256	-4.40596594	-2.74658497
H	2.24841484	-3.52162686	1.49192859
H	1.10897330	-4.30228615	3.55715854
H	-0.96952948	-3.14244792	4.33093631
H	-1.86056399	-1.23719887	3.05531372
H	1.01266483	1.38452085	0.71952693
H	-0.54781260	2.74211506	-0.61583164
H	-1.51070016	3.11318442	0.81843876
H	0.02457464	4.99567620	0.34577890
H	1.41751695	3.90671168	0.37123495
H	1.22688562	5.10568099	2.54970627
H	-0.42777220	4.51573875	2.74990163
H	1.13467146	3.11117659	4.08180476
H	2.10396408	2.78312966	2.64236150
H	0.60743279	0.85380266	3.09526909
H	-0.79409529	1.92109490	3.13412856
H	-3.03936455	-0.77290015	1.16128861
H	-3.29009051	0.24602067	-1.07345860
H	-3.22693931	1.86609763	-0.38033987
H	-5.37660955	-0.25564608	0.17425406
H	-5.58791651	1.22027283	-0.77480345
H	-6.49736943	1.47478652	1.56196012
H	-5.18605356	2.61680187	1.24330117
H	-4.92728111	-0.04306716	2.75668635
H	-4.86539530	1.57032612	3.47746844

H	-2.80953111	2.10482748	2.20105500
H	-2.57146533	0.66147664	3.17961890
H	2.55993746	-0.24798324	1.95337135
H	4.88085701	-0.86871294	2.65203304
H	5.27770294	-1.27323276	0.96346307
H	4.06728702	-2.22929130	1.84608381
H	3.21203294	1.87550072	0.68198136
H	4.21108766	1.52949686	2.11422175
H	4.84464462	1.20349094	0.49548127
H	4.32707109	0.15498319	-1.29986196

Compound **38**

Energy: -163.45514822124525 eV

Atom	X (Å)	Y (Å)	Z (Å)
C	-1.30715641	-3.70940217	2.92519957
C	-1.81462983	-3.33737259	1.67829186
C	-1.08664982	-2.45124398	0.88813877
C	0.13681605	-1.97053778	1.38146885
P	1.19804635	-0.74226181	0.49067625
C	0.08619862	0.72835865	0.70355113
C	0.53150177	1.75482617	1.54913409
C	-0.29798558	2.85407889	1.77653359
C	-1.54238096	2.89376954	1.15024761
C	-1.89983891	1.82588367	0.32057518
N	-1.11531235	0.76479843	0.09504650
C	0.84995353	-1.17262157	-1.26770169
C	0.72472415	-0.18510808	-2.25918759
C	0.60403076	-0.58020955	-3.58972382
C	0.61179244	-1.94208071	-3.89471700
C	0.75413288	-2.85082300	-2.84464844
N	0.88481124	-2.48722106	-1.56203833
N	0.63039105	-2.31973254	2.58071994
C	-0.08480549	-3.17201517	3.33034090
H	-1.84683512	-4.40007660	3.57381529
H	-2.76901108	-3.73276261	1.32655375
H	-1.45448178	-2.12662634	-0.08366773
H	1.51208738	1.67935665	2.02073951
H	0.02342740	3.66484016	2.43234248
H	-2.22689066	3.72933341	1.29788413
H	-2.86806309	1.82237969	-0.18884576
H	0.71002321	0.87108974	-1.99161609
H	0.50353393	0.16665025	-4.37891624
H	0.51784510	-2.29510712	-4.92187724
H	0.77144507	-3.92717516	-3.03998784
H	0.34666201	-3.43672724	4.29989758

Compound **39**

Energy: -163.36812200799858 eV

Atom	X (Å)	Y (Å)	Z (Å)
C	1.51107171	-3.66672578	1.24502674
C	1.99324240	-3.41022735	-0.17813796
O	0.90427454	-2.71412532	-0.80111124
C	0.28887205	-1.85716147	0.19227150
P	0.73252562	-0.05849221	-0.12548195
C	-0.33756174	0.24275814	-1.64842899
C	0.25249666	-0.36645102	-2.92165424
C	1.21225379	0.73669277	-3.38263281
C	0.44946925	2.00936772	-3.00997626
O	-0.42123570	1.66068536	-1.90570332
C	-0.38134468	0.69169766	1.18496280
C	-0.29524169	2.23094410	1.25366661
C	-1.73830885	2.64516757	1.57613926
C	-2.53431191	1.57037531	0.84388214
O	-1.78390851	0.35473892	1.03378314
C	0.79841706	-2.34258115	1.56974837
H	2.32770273	-3.89337225	1.94329581
H	0.80086714	-4.50607762	1.25454748
H	2.90665573	-2.78449676	-0.18058596
H	2.17978918	-4.31676796	-0.76849625
H	-0.80009360	-1.98603967	0.08878369
H	-1.35549756	-0.11331806	-1.42570854
H	0.74050390	-1.33187914	-2.74404381
H	-0.55244013	-0.51249266	-3.65928799
H	1.44473425	0.68667761	-4.45427441
H	2.15572046	0.67390886	-2.82137841
H	-0.18223275	2.36606235	-3.84163714
H	1.10851217	2.83200948	-2.69293961
H	-0.05340992	0.24482423	2.14047646
H	0.00278745	2.63666132	0.27792396
H	0.43101849	2.56191434	2.00696416
H	-1.97584036	3.65962635	1.22933137
H	-1.93255379	2.59121135	2.65794445
H	-2.61121108	1.80470168	-0.23334004
H	-3.54044985	1.39804088	1.25034453
H	1.51944735	-1.62523695	1.99201947
H	-0.02145466	-2.45623815	2.28988191

Compound **40**
Energy: -344.4228601235645 eV

Atom	X (Å)	Y (Å)	Z (Å)
C	-3.41765493	0.24452593	-3.56270751
C	-3.23515406	-1.13001954	-2.90189818
C	-4.55079020	-1.62345095	-2.28356402
C	-2.10596912	-1.10959984	-1.88786646
C	-2.18003024	-0.32812683	-0.73121842
C	-1.13660472	-0.27769642	0.19719803
C	-1.33822323	0.49968884	1.48945176

C	-2.20525602	-0.31542479	2.46407545
C	-1.93030467	1.89490001	1.26456500
C	0.04287192	-1.02258351	-0.04160469
C	1.16760099	-0.99438780	0.95361986
C	1.29249418	-2.09216725	1.82295933
C	2.32879052	-2.19109468	2.74907169
C	3.28034877	-1.17140826	2.81965610
C	3.15552550	-0.06064458	1.98905047
C	2.10573860	0.06982225	1.05759598
P	1.84412625	1.61166048	0.06293427
C	2.20322061	3.07006343	1.25469538
C	3.65969345	3.46269778	1.53561576
C	1.50040044	2.77323973	2.58919167
C	1.48856165	4.28108468	0.61589954
C	3.22501404	1.52439796	-1.24165695
C	3.24682390	2.85457698	-2.00957917
C	2.75158445	0.41687184	-2.19617004
C	4.63277354	1.16153917	-0.75740531
C	0.11547705	-1.86474121	-1.17835719
C	1.28833435	-2.80673377	-1.43494056
C	1.72014013	-2.86499690	-2.90721629
C	0.95029278	-4.23002242	-0.95438917
C	-0.95724141	-1.87687009	-2.08099552
H	-3.70486178	1.00141101	-2.81769255
H	-4.20619749	0.21000372	-4.32883719
H	-2.48664544	0.58160080	-4.03921848
H	-2.94516274	-1.84551692	-3.68955097
H	-5.34242326	-1.68217849	-3.04485861
H	-4.89657956	-0.93902065	-1.49469234
H	-4.42947070	-2.61853554	-1.83365668
H	-3.08215555	0.25566895	-0.54008212
H	-0.35285960	0.62329278	1.95902711
H	-1.74615094	-1.29012074	2.67983368
H	-2.33354633	0.22219559	3.41568004
H	-3.20308503	-0.49938592	2.03841363
H	-1.33066625	2.46346817	0.54085597
H	-2.96170103	1.84449927	0.88587318
H	-1.96200836	2.45584141	2.21053074
H	0.54472905	-2.88410683	1.76435068
H	2.39164925	-3.05854441	3.40769149
H	4.10867356	-1.23261698	3.52724704
H	3.89521010	0.73502371	2.06819864
H	3.66460685	4.33109393	2.21540567
H	4.23426292	2.66720004	2.02611675
H	4.19645063	3.76080938	0.62645536
H	1.56516179	3.66468560	3.23377812
H	1.95799006	1.93175387	3.12391008
H	0.43685233	2.54771048	2.44210355
H	0.42721225	4.06210132	0.43626580
H	1.55170191	5.14508865	1.29750773
H	1.93801055	4.57572818	-0.34066268

H	2.23796483	3.14170952	-2.34011137
H	3.66268107	3.67860237	-1.41569590
H	3.87747558	2.74521899	-2.90669525
H	2.76670398	-0.55734553	-1.69426398
H	1.72818613	0.59578361	-2.55505367
H	3.42648343	0.36218947	-3.06590309
H	5.05554295	1.90426974	-0.07130376
H	4.64068060	0.18566462	-0.25467366
H	5.30701608	1.09401717	-1.62790675
H	2.14364409	-2.44939847	-0.84053936
H	2.66403543	-3.42158984	-2.99958570
H	1.86697864	-1.86696841	-3.33735949
H	0.97606102	-3.39086746	-3.52298880
H	0.08543913	-4.62349725	-1.50916181
H	0.70271453	-4.25479172	0.11374159
H	1.80016347	-4.90835141	-1.12288034
H	-0.89987142	-2.50903113	-2.96932531

Compound **41**
Energy: -201.5680655260456 eV

Atom	X (Å)	Y (Å)	Z (Å)
C	-2.87523796	2.31274233	0.32788490
C	-1.50196530	2.61906154	-0.28034592
C	-1.64095017	3.19634035	-1.69458925
P	-0.51508529	1.03652196	-0.36996848
C	-0.19283701	0.64736730	1.40841020
C	-0.16555647	1.64073175	2.40603936
C	0.14160349	1.34414043	3.73276924
C	0.44568442	0.03016965	4.09571605
C	0.41649012	-0.97033887	3.12632579
C	0.08320615	-0.69196277	1.78917381
C	0.04498502	-1.83454154	0.83762331
C	1.14938283	-2.69545676	0.73007350
C	1.12550514	-3.78719779	-0.13925874
C	-0.01209965	-4.04625048	-0.90760852
C	-1.12462420	-3.20804718	-0.79529240
C	-1.09707421	-2.11240555	0.06797969
C	1.17446282	1.62937658	-0.93972775
C	1.78020350	2.80722184	-0.17715956
C	2.13326831	0.43342454	-0.97144863
H	-3.49636537	3.22111603	0.35681915
H	-2.79085319	1.92359742	1.35162214
H	-3.40690636	1.56088008	-0.27518173
H	-0.99078736	3.36780913	0.34657226
H	-2.06961283	2.45139573	-2.38231081
H	-2.30946541	4.07080852	-1.68691061
H	-0.67576684	3.51965492	-2.10796172
H	-0.37757097	2.67640932	2.13982800
H	0.15220517	2.14024004	4.47887776
H	0.69378323	-0.21662863	5.12890286

H	0.62766621	-2.00399308	3.40608825
H	2.04409645	-2.48768092	1.31957275
H	2.00074103	-4.43345866	-0.22147477
H	-0.03239996	-4.89751458	-1.58922710
H	-2.02081702	-3.40622208	-1.38495036
H	-1.96938261	-1.46511683	0.15438404
H	0.98531172	1.93903280	-1.98258826
H	1.13673607	3.69763568	-0.19792195
H	2.74889872	3.08885634	-0.61985479
H	1.95941146	2.54347865	0.87474519
H	3.07573370	0.71030394	-1.46777463
H	2.37693857	0.10361734	0.04870575
H	1.70008398	-0.42465651	-1.50353206

Compound **42**

Energy: -287.9484046216719 eV

Atom	X (Å)	Y (Å)	Z (Å)
C	-3.78524163	3.16936939	2.13729556
C	-2.51947662	3.49016368	1.59220501
C	-1.63571844	2.49188745	1.23737333
C	-1.96862924	1.12108385	1.40687864
C	-1.08407044	0.05108118	1.03415515
P	0.56336403	0.48766003	0.35434104
C	0.19268008	0.87952974	-1.39949549
C	-1.07474571	0.72764896	-1.93899779
C	-1.34612575	1.08440707	-3.28047045
C	-0.34715461	1.58628135	-4.08443368
C	0.96824818	1.76446178	-3.57956419
C	2.01179734	2.28325260	-4.39175925
C	3.28093528	2.46481454	-3.88684984
C	3.55702776	2.13834700	-2.53799005
C	2.56624772	1.63126441	-1.72268063
C	1.24822962	1.42236252	-2.21036434
C	1.40466371	-1.13909325	0.24256576
C	1.54773125	-1.81643804	-0.95791759
C	2.24378665	-3.04580606	-1.02549378
C	2.79089903	-3.60081324	0.10976174
C	2.67567066	-2.94546418	1.36442340
C	3.23621287	-3.49957158	2.54619009
C	3.12960688	-2.84808410	3.75565417
C	2.45861752	-1.60453843	3.83040373
C	1.90238829	-1.03923020	2.70143808
C	1.98522139	-1.68535861	1.43876017
C	-1.46905447	-1.26204337	1.25314337
C	-2.72559632	-1.57012886	1.82462687
C	-3.60000332	-0.56411036	2.17040187
C	-3.25271104	0.79831418	1.96997638
C	-4.13894045	1.85070782	2.32337617
H	-4.47532721	3.96770414	2.41343678
H	-2.23971418	4.53533963	1.45403470

H	-0.65910251	2.75402498	0.82560061
H	-1.87955490	0.32823717	-1.32124206
H	-2.35510337	0.95437493	-3.67444429
H	-0.55094494	1.85846059	-5.12198558
H	1.78558534	2.53839946	-5.42915617
H	4.07300627	2.86419017	-4.52162650
H	4.56077895	2.29129910	-2.13938766
H	2.79131072	1.39140329	-0.68160931
H	1.11905260	-1.39668911	-1.86835912
H	2.34003276	-3.55338707	-1.98632707
H	3.32505580	-4.55194203	0.06316150
H	3.75784315	-4.45631127	2.47531518
H	3.56493912	-3.28481641	4.65542581
H	2.38309490	-1.08789575	4.78808733
H	1.39489480	-0.07514821	2.77221811
H	-0.79360769	-2.07427881	0.98314617
H	-2.99786932	-2.61419304	1.98571784
H	-4.57297892	-0.79737647	2.60747907
H	-5.11027311	1.59160582	2.74978180

Compound **43**
Energy: -305.5906034240835 eV

Atom	X (Å)	Y (Å)	Z (Å)
C	2.48196050	0.49578188	-3.88756004
C	2.19698175	-0.60075190	-2.85115251
C	2.77543231	-1.93406758	-3.35978033
C	2.73888866	-0.27957545	-1.46168753
C	4.02172245	0.27637081	-1.35859746
C	4.60687109	0.52056052	-0.11930356
C	3.91824293	0.18584881	1.04350718
C	2.63372245	-0.36869781	0.98820493
C	1.97052406	-0.82598448	2.27762630
C	2.56744915	-2.16990823	2.72908779
C	2.05778109	0.21689474	3.39699891
C	2.02198935	-0.57149648	-0.27215565
C	0.65557165	-1.19143567	-0.35188949
C	0.58516930	-2.55575058	-0.68367143
C	-0.63008864	-3.21956437	-0.83702652
C	-1.82075113	-2.51190711	-0.65946359
C	-1.77090539	-1.16800054	-0.29810044
C	-0.55393546	-0.47990303	-0.11899745
P	-0.47957474	1.25860778	0.51926166
C	-1.05470717	2.29284601	-0.96951212
C	-2.29718173	1.81250245	-1.72694241
C	-1.23692477	3.74553133	-0.50450808
C	0.14003668	2.23550854	-1.93442438
C	-1.80605041	1.36365873	1.90008037
C	-3.27900477	1.53792032	1.50710449
C	-1.67016695	0.11211793	2.78213130
C	-1.38490067	2.57879496	2.75531951

H	1.92601283	0.28979554	-4.81367237
H	3.54838643	0.52823032	-4.15409772
H	2.19482231	1.49173504	-3.52946875
H	1.10555952	-0.72118496	-2.76890258
H	2.53665654	-2.76810774	-2.68898748
H	3.87085153	-1.86844928	-3.43871803
H	2.37612805	-2.17374126	-4.35667637
H	4.57508053	0.51951965	-2.26622662
H	5.60446524	0.95855417	-0.06059389
H	4.39106689	0.34933767	2.01268203
H	0.90796431	-1.00047360	2.06068485
H	2.07422609	-2.52657662	3.64582666
H	3.64316000	-2.06981742	2.93710066
H	2.44404567	-2.93727819	1.95245278
H	1.48271621	-0.11375012	4.27473156
H	3.09408265	0.37175483	3.73085250
H	1.66028151	1.18504392	3.06350647
H	1.51903660	-3.10293965	-0.81912657
H	-0.64491310	-4.27947252	-1.09478813
H	-2.78495437	-3.00634008	-0.78728992
H	-2.70819684	-0.63487810	-0.14444029
H	-2.47182028	2.47668037	-2.59025925
H	-2.15791363	0.79468173	-2.11396565
H	-3.20549174	1.82507672	-1.11385748
H	-0.36368962	4.09537630	0.06514674
H	-2.13119020	3.88129860	0.11710988
H	-1.34763211	4.39727720	-1.38620856
H	-0.04094051	2.90642859	-2.78997453
H	1.07686697	2.53533711	-1.44398154
H	0.27296189	1.21968958	-2.32376470
H	-3.88258714	1.60489529	2.42765231
H	-3.67295414	0.69563405	0.92505915
H	-3.45510106	2.45874483	0.93741906
H	-1.96636354	-0.80378116	2.25605252
H	-2.31921175	0.22479826	3.66550159
H	-0.64179125	-0.01663883	3.14182727
H	-0.34631346	2.47777544	3.09855742
H	-2.03600015	2.64551805	3.64228605
H	-1.46788960	3.52563943	2.20725009

Compound **44**

Energy: -268.53751784915073 eV

Atom	X (Å)	Y (Å)	Z (Å)
C	-4.13851493	-0.51027956	1.67859077
C	-4.18380160	-0.80637964	0.31403242
C	-3.04457333	-1.28190411	-0.33750633
C	-1.83682471	-1.45659622	0.35735369
C	-0.64109864	-1.98888287	-0.34915563
C	-0.78229993	-3.20421550	-1.04121222
C	0.29192605	-3.79670165	-1.70176745

C	1.53909220	-3.16801212	-1.68184217
C	1.68903710	-1.94945806	-1.02268752
C	0.61534777	-1.32791777	-0.35524222
P	0.76467716	0.32492349	0.45365757
C	2.59115963	0.43072392	0.84042009
C	2.95558288	1.85291815	1.31054740
C	2.34436854	2.19967005	2.67206799
C	2.73515101	1.16709266	3.73472434
C	2.36045391	-0.25163578	3.29351840
C	2.96315219	-0.59679536	1.92727338
C	0.58678459	1.48949325	-1.00513385
C	-0.86204236	1.43766675	-1.51687076
C	-1.10951179	2.47230585	-2.61949333
C	-0.11825332	2.30126333	-3.77460022
C	1.32770404	2.35330202	-3.27178734
C	1.57770405	1.32134366	-2.16353843
C	-1.80742540	-1.16701949	1.73185509
C	-2.94768429	-0.69883413	2.38526832
H	-5.02677663	-0.13629846	2.18920376
H	-5.10778348	-0.66071282	-0.24764888
H	-3.07869233	-1.50052404	-1.40629240
H	-1.75441753	-3.70030213	-1.02989069
H	0.15858299	-4.74743303	-2.21959427
H	2.39361149	-3.62043449	-2.18737206
H	2.66600307	-1.46595690	-1.03947272
H	3.17848883	0.21559215	-0.06675137
H	4.05619745	1.90859625	1.38809266
H	2.66449287	2.59810792	0.55450489
H	2.66350042	3.20758698	2.97894913
H	1.24521847	2.22708927	2.58114372
H	3.82554079	1.21755753	3.90402080
H	2.25721467	1.40739957	4.69674669
H	2.69175585	-0.98760822	4.04226617
H	1.26161127	-0.33064780	3.23567400
H	4.06426027	-0.61496602	2.01532131
H	2.65655655	-1.60696682	1.61633255
H	0.74362384	2.48738883	-0.55214794
H	-1.56435323	1.57833488	-0.68130485
H	-1.05771555	0.42900355	-1.91880426
H	-1.00296822	3.48697884	-2.19695998
H	-2.14527011	2.39214515	-2.98490082
H	-0.29605654	1.32599394	-4.26092677
H	-0.28539101	3.07157927	-4.54324599
H	2.03111817	2.18894521	-4.10290032
H	1.53708227	3.36358585	-2.87785740
H	1.46911274	0.30589501	-2.57998327
H	2.61466231	1.40601531	-1.80428353
H	-0.88338244	-1.31880670	2.28952012
H	-2.90401048	-0.47894138	3.45291007

Compound **45**
Energy: -159.33079514586186 eV

Atom	X (Å)	Y (Å)	Z (Å)
C	2.71318531	2.12547815	1.62103284
C	1.90263563	3.09212179	0.75834309
S	0.24244980	2.34084052	0.59865722
C	0.81214901	0.64889727	1.06074999
P	0.21563921	-0.75538814	-0.01317095
C	0.55775233	-0.10790810	-1.73357754
C	0.73747414	-1.26612620	-2.73469376
C	0.47178653	-0.71879272	-4.13700580
C	-0.82031218	0.08660721	-4.02786493
S	-0.66685850	1.05104352	-2.48148147
C	-1.62703944	-0.53758771	0.20204023
C	-2.11765551	-1.15159535	1.51983792
C	-2.27278817	-2.65387945	1.27888726
C	-3.06120208	-2.80774378	-0.02182519
S	-2.51934035	-1.43550599	-1.11915511
C	2.34539466	0.71985420	1.14411293
H	2.43251841	2.24786174	2.67848414
H	3.79277076	2.32063211	1.53348728
H	2.35008056	3.20840305	-0.23954455
H	1.79673523	4.08573295	1.21217305
H	0.40576768	0.43765360	2.06301784
H	1.49852605	0.45659798	-1.63988909
H	1.74113465	-1.70483406	-2.63576618
H	0.00412649	-2.05349363	-2.50363369
H	1.29542833	-0.05965297	-4.45281677
H	0.38010925	-1.52735303	-4.87850894
H	-0.96186701	0.79108616	-4.85792528
H	-1.69695708	-0.57399743	-3.96549742
H	-1.89294284	0.52538228	0.10984230
H	-1.42857772	-0.93895578	2.35184278
H	-3.09123354	-0.70634477	1.77886453
H	-1.27803398	-3.10998783	1.16557595
H	-2.78276701	-3.15502528	2.11477912
H	-2.86983601	-3.76588335	-0.52100343
H	-4.14265766	-2.71238822	0.15087696
H	2.78352049	0.54393851	0.14775966
H	2.73047649	-0.06605035	1.80979361

Compound **46**
Energy: -387.2616120199702 eV

Atom	X (Å)	Y (Å)	Z (Å)
C	2.16719725	-2.95626115	2.07870994
C	1.78199510	-1.76874164	1.18686402
N	1.33285577	-0.59141049	1.94165780
C	2.26947622	-0.03255982	2.90119985

P	-0.34862739	-0.51187335	2.08614484
C	-0.61207577	1.26909849	2.42577884
C	-1.61353795	1.72692825	3.29574828
C	-1.86484206	3.09448120	3.42672320
C	-1.12098293	4.01963426	2.68882317
C	-0.12441379	3.57119055	1.81633537
C	0.12617387	2.20478457	1.68015094
C	-0.77788386	-1.32877127	3.68506629
C	-1.41680630	-2.57737526	3.63738109
C	-1.69054607	-3.28114035	4.81274564
C	-1.32119410	-2.74606064	6.04925006
C	-0.68011994	-1.50478733	6.10679116
C	-0.40681228	-0.79967870	4.93235920
C	0.71942931	-2.11943505	0.17381983
C	-0.10334798	-3.29136467	0.14856215
C	-0.86626991	-3.26059519	-1.04625101
C	-0.55404305	-2.06609520	-1.75071338
C	0.42403582	-1.32930387	-1.00476388
P	1.28089194	0.22772436	-1.40254251
C	2.54006597	-0.36758310	-2.59891962
C	2.88224831	-1.72042839	-2.74763182
C	3.91566633	-2.09773040	-3.60968116
C	4.61854608	-1.13268248	-4.33474597
C	4.28679106	0.21784874	-4.19076539
C	3.26240959	0.59749514	-3.32290831
C	0.05382225	1.01632281	-2.52117184
C	-0.72845576	2.05028352	-1.98672118
C	-1.72001544	2.66580377	-2.75477586
C	-1.93628858	2.25762646	-4.07296706
C	-1.14955956	1.23823403	-4.62045618
C	-0.15937239	0.62268796	-3.85212743
Fe	-1.32885641	-1.41112387	0.20914832
C	-2.97077722	-0.44723286	-0.99620156
C	-2.76248196	0.19536591	0.25644636
C	-3.08769740	-0.75462829	1.29031659
C	-3.47683001	-1.97569957	0.66031361
C	-3.41736312	-1.77521252	-0.74404861
H	1.32084505	-3.27041120	2.70536526
H	2.48639877	-3.81004501	1.46521756
H	3.00065061	-2.69011089	2.74125709
H	2.68128215	-1.45799383	0.62055263
H	3.23922063	0.12937170	2.40358222
H	1.90219042	0.93939273	3.25180604
H	2.43202302	-0.67981912	3.78114789
H	-2.20098493	1.01408267	3.87609655
H	-2.64592456	3.43672305	4.10679586
H	-1.32032913	5.08712878	2.79006082
H	0.45768933	4.28728169	1.23417622
H	0.89192267	1.84526541	0.98889369
H	-1.69593695	-2.98874095	2.66454823
H	-2.18891488	-4.25023643	4.76110688

H	-1.52936364	-3.29608734	6.96786592
H	-0.38999168	-1.08333999	7.07033913
H	0.08716850	0.17197022	4.98729075
H	-0.12698070	-4.07114911	0.90383829
H	-1.61975803	-3.98716943	-1.33507328
H	-1.01601598	-1.73488895	-2.67372406
H	2.33114369	-2.48523978	-2.19744101
H	4.16873040	-3.15378941	-3.71696238
H	5.42355994	-1.43076637	-5.00755472
H	4.83228953	0.97819992	-4.75180661
H	3.01434030	1.65570154	-3.21196703
H	-0.57099424	2.36273372	-0.95289252
H	-2.32788682	3.46049285	-2.31985502
H	-2.71284702	2.73210864	-4.67409176
H	-1.30942712	0.91969239	-5.65179109
H	0.45565436	-0.16765541	-4.28594851
H	-2.77429923	-0.01358201	-1.97152794
H	-2.44749189	1.22242313	0.40774274
H	-3.09587654	-0.57010729	2.36055602
H	-3.76503820	-2.89437773	1.16427688
H	-3.60021143	-2.53184893	-1.50107351

Compound **47**

Energy: -376.7096701749864 eV

Atom	X (Å)	Y (Å)	Z (Å)
C	-1.18697943	-1.76090785	-3.20052458
C	-1.05449193	-0.89589541	-1.94487320
N	-1.48516004	0.47775351	-2.20653211
P	-0.74877217	1.67116013	-1.26850540
C	-1.99153623	2.15938779	-0.00647461
C	-2.15926590	3.47644181	0.44806599
C	-3.01372966	3.75256299	1.51816019
C	-3.70664684	2.71663471	2.15130846
C	-3.54163952	1.40032901	1.70939152
C	-2.68754180	1.12379618	0.64049446
C	-0.71217354	3.09349086	-2.42932993
C	-1.85689585	3.83858031	-2.75513968
C	-1.78226091	4.86455754	-3.70027992
C	-0.56755781	5.15470686	-4.32866010
C	0.57338363	4.41239556	-4.01407800
C	0.50057544	3.38455008	-3.07118024
C	0.36114319	-0.83900416	-1.43486208
C	1.49637402	-0.32975744	-2.16054459
C	2.64618625	-0.53947209	-1.35063598
C	2.22890780	-1.13390475	-0.12679214
C	0.81109756	-1.32608661	-0.15901231
P	-0.33765746	-2.06149386	1.04721507
C	0.39031694	-1.54280962	2.64791310
C	-0.26134994	-0.50529771	3.33138192
C	0.24897446	-0.01440171	4.53549702

C	1.41626048	-0.56193513	5.07398833
C	2.06589120	-1.60584016	4.40597011
C	1.55691918	-2.09521600	3.20088142
C	0.15661661	-3.82736587	0.96308614
C	-0.24864614	-4.68516921	2.00079300
C	0.01381296	-6.05408439	1.93496887
C	0.66946796	-6.58990730	0.82251009
C	1.06363138	-5.74707210	-0.21955992
C	0.80940114	-4.37470741	-0.15216743
Fe	1.20986347	0.85740694	-0.38076496
C	2.14366446	1.30396833	1.62633983
C	0.99251633	2.10388457	1.38337888
C	1.28047116	2.94306902	0.25074445
C	2.59663709	2.63045075	-0.20992670
C	3.12671754	1.63180400	0.65056601
H	-2.22914932	-1.76641400	-3.55364291
H	-0.89923172	-2.79903686	-2.98223530
H	-0.55035283	-1.37850392	-4.00953263
H	-1.65938322	-1.35767993	-1.14023707
H	-2.43880216	0.59927202	-2.54023664
H	-1.62215723	4.29486549	-0.03352780
H	-3.13502765	4.78119069	1.86023622
H	-4.36956654	2.93412502	2.98961513
H	-4.07293211	0.58424074	2.20151332
H	-2.53997794	0.09043713	0.31994914
H	-2.80693730	3.62018186	-2.26380368
H	-2.67648946	5.43881552	-3.94637452
H	-0.51263409	5.95604806	-5.06657494
H	1.52280369	4.63149517	-4.50458428
H	1.38672237	2.79714034	-2.82228868
H	1.47447405	0.08990745	-3.16232946
H	3.66087593	-0.23834453	-1.59136235
H	2.86781787	-1.34136297	0.72445020
H	-1.16533412	-0.06777426	2.90241548
H	-0.26170726	0.80087441	5.04996675
H	1.82039040	-0.17762962	6.01120620
H	2.97551353	-2.03913258	4.82473590
H	2.06262737	-2.91467091	2.68736228
H	-0.76529589	-4.27614283	2.87186801
H	-0.29986560	-6.70592210	2.75174201
H	0.86890612	-7.66072524	0.76694754
H	1.57548758	-6.15807740	-1.09129830
H	1.13329758	-3.72356170	-0.96626331
H	2.23389797	0.53566774	2.38623073
H	0.07414566	2.10171759	1.96100220
H	0.64229350	3.72052072	-0.15886564
H	3.10323975	3.08266556	-1.05801936
H	4.08946238	1.14205170	0.53684632

Compound **48**

Energy: -126.24210087644042 eV

Atom	X (Å)	Y (Å)	Z (Å)
C	0.67368212	-2.38649734	-2.84861340
C	0.45010153	-1.04643233	-3.07908116
S	-0.22672476	-0.27423350	-1.71136707
C	-0.22472438	-1.73386440	-0.78686657
P	-0.72732236	-1.87984686	0.95103670
C	0.55217476	-0.94190812	1.82647882
C	1.33560877	-1.47043555	2.83978845
C	2.29378449	-0.55442366	3.34602624
C	2.23290248	0.66552086	2.70978210
S	1.01767350	0.69404964	1.50537860
C	-2.14134758	-0.74322848	1.04398142
C	-2.46195356	0.15342290	2.04861719
C	-3.75975135	0.71178171	1.90186837
C	-4.41954240	0.22641048	0.79510747
S	-3.46048893	-0.90627628	-0.05943478
C	0.28950602	-2.77674673	-1.53962917
H	1.09510045	-3.06121549	-3.59170199
H	0.63720867	-0.48040309	-3.98730662
H	1.21413556	-2.49228510	3.19831403
H	2.99860811	-0.78109980	4.14397907
H	2.83833287	1.54916535	2.89142737
H	-1.78041885	0.39781308	2.86147813
H	-4.18926641	1.44437055	2.58310170
H	-5.40985732	0.48429142	0.43141720
H	0.38558414	-3.78911818	-1.14812015

Compound **49**

Energy: -353.61102034291616 eV

Atom	X (Å)	Y (Å)	Z (Å)
C	-2.02822782	2.60154177	-4.79821714
C	-0.69349762	2.15970791	-4.18165956
C	-0.01374116	1.08808937	-5.04700319
C	-0.86228856	1.68872845	-2.74857625
C	-1.67563745	0.59423437	-2.43545141
C	-1.83137114	0.13529768	-1.12352038
C	-2.75388596	-1.04578409	-0.84777883
C	-4.22318975	-0.59204269	-0.84153264
C	-2.55038641	-2.20538541	-1.83047483
C	-1.14444943	0.80029535	-0.08206036
C	-1.32460409	0.36735984	1.34084218
C	-2.42293464	0.84369631	2.07282774
C	-2.61429991	0.48579240	3.40765139
C	-1.69590685	-0.35966348	4.03653591
C	-0.59677397	-0.83980036	3.32524317
C	-0.39918982	-0.49072887	1.97743521
P	1.04215827	-1.06810540	0.99245982
C	2.23417650	-1.51277031	2.31199967
C	2.73877293	-2.80721385	2.50450578

C	3.71639903	-3.04725876	3.47432552
C	4.20141297	-2.00244477	4.26312333
C	3.70912893	-0.70742272	4.07343153
C	2.74160986	-0.46298756	3.09987102
C	0.48084262	-2.70676637	0.39043442
C	1.11633126	-3.21261869	-0.75545187
C	0.75252958	-4.45290558	-1.28483281
C	-0.26230135	-5.19939711	-0.68143435
C	-0.90955318	-4.69812100	0.45144226
C	-0.54167111	-3.46232642	0.98632614
C	-0.31659971	1.90588034	-0.37308314
C	0.43360210	2.66142446	0.71486250
C	-0.13830487	4.07616800	0.89446837
C	1.94479427	2.70216814	0.44887139
C	-0.19348841	2.32730002	-1.70323191
H	-2.73137066	1.75773298	-4.86033543
H	-2.50241874	3.38844544	-4.19553253
H	-1.87927077	2.98821295	-5.81707420
H	-0.02902119	3.03950283	-4.15313582
H	0.95788022	0.79615934	-4.62498663
H	-0.63619490	0.18316356	-5.11040717
H	0.14936775	1.45638247	-6.07053928
H	-2.20283291	0.07702423	-3.23921176
H	-2.51781550	-1.42370651	0.15826844
H	-4.50681279	-0.18604462	-1.82421216
H	-4.39818140	0.19089015	-0.09137305
H	-4.89145652	-1.43679275	-0.61685609
H	-3.14744921	-3.07419606	-1.51788202
H	-1.49865449	-2.51583866	-1.87338111
H	-2.87088091	-1.93787474	-2.84828496
H	-3.12884254	1.51514945	1.58059847
H	-3.47509589	0.87083242	3.95623308
H	-1.83293305	-0.64257556	5.08121984
H	0.12651420	-1.48914302	3.82169856
H	2.36425299	-3.63292712	1.89806804
H	4.09909299	-4.05963432	3.61355993
H	4.96369699	-2.19370057	5.01936458
H	4.08674874	0.11635454	4.68131356
H	2.36921171	0.55275819	2.94885485
H	1.89761017	-2.62165044	-1.23828467
H	1.25673247	-4.83224684	-2.17493775
H	-0.55294126	-6.16546327	-1.09592350
H	-1.70928926	-5.27279995	0.92157781
H	-1.05990895	-3.07806750	1.86582348
H	0.28023869	2.12300461	1.66173589
H	0.38420937	4.60797766	1.70351499
H	-0.02426102	4.66676654	-0.02664526
H	-1.20884544	4.04219390	1.14036937
H	2.17664341	3.26244509	-0.46884006
H	2.46835106	3.19827520	1.27983224
H	2.34920694	1.68683662	0.33631157

H	0.44652576	3.18168343	-1.93630512

Compound **50**
Energy: -227.45732157212424 eV

Atom	X (Å)	Y (Å)	Z (Å)
C	-2.29376643	2.48633701	0.41790871
C	-1.33139411	1.42423965	0.97168893
C	-0.42049313	2.04130451	2.03715207
C	-2.15816196	0.29959300	1.62541127
P	-0.50088732	0.56618753	-0.50607747
C	0.76779958	-0.48529491	0.33411752
C	1.94218743	0.02673345	0.91894186
C	2.84288357	-0.78580196	1.60396071
C	2.57879856	-2.15117179	1.73777161
C	1.42379320	-2.68150449	1.16727163
C	0.51983120	-1.87821966	0.44943882
C	-0.66850728	-2.54819149	-0.14417203
C	-1.52746496	-3.30308078	0.66987806
C	-2.63300909	-3.96202394	0.12834208
C	-2.88980445	-3.88914630	-1.24259448
C	-2.03115943	-3.15596170	-2.06626847
C	-0.93241939	-2.49071146	-1.52258838
C	0.47027762	1.81449123	-1.56537087
C	-0.54930397	2.32565136	-2.60638082
C	1.12223677	3.02611571	-0.88876745
C	1.53743518	1.00135341	-2.31867171
H	-1.76194204	3.35784616	0.01324521
H	-2.93385262	2.07339839	-0.37527336
H	-2.94772885	2.84672943	1.22848137
H	0.21117914	2.84579276	1.64146129
H	-1.04309905	2.47321061	2.83889367
H	0.23178853	1.28554223	2.49265639
H	-1.50891720	-0.47396840	2.05771604
H	-2.82450497	-0.18660854	0.89915430
H	-2.77347838	0.72048120	2.43711913
H	2.14939164	1.09396098	0.85272927
H	3.74308281	-0.35134679	2.04164393
H	3.27036810	-2.80019252	2.27670875
H	1.21678589	-3.74987467	1.25085066
H	-1.33422200	-3.35341150	1.74290004
H	-3.29810066	-4.52982155	0.78080971
H	-3.75324928	-4.40220100	-1.66779419
H	-2.21841099	-3.09940445	-3.13953861
H	-0.26276229	-1.92540082	-2.17056008
H	-0.03408869	2.96235962	-3.34407291
H	-1.01900079	1.48826305	-3.14089767
H	-1.34817346	2.92274122	-2.14839056
H	1.88758429	2.74952788	-0.15330678
H	1.61867400	3.64071200	-1.65847058
H	0.38380821	3.66624580	-0.38873901

H	2.33490627	0.64760816	-1.65357950
H	1.99234401	1.63143214	-3.09965505
H	1.09525765	0.12309492	-2.81253307

Compound **51**
Energy: -130.23792999699006 eV

Atom	X (Å)	Y (Å)	Z (Å)
C	2.95558297	2.38260274	0.63869836
C	3.83676163	1.57103131	-0.02027369
O	3.16706910	0.70110459	-0.83322091
C	1.81559597	0.96174933	-0.68186529
P	0.69811831	-0.03452809	-1.70363947
C	-0.83413411	0.87373606	-1.36777171
C	-1.85250095	0.74737079	-0.45577741
C	-2.81067995	1.76249843	-0.76299457
C	-2.31952836	2.44292470	-1.84240038
O	-1.12001532	1.91759167	-2.23144189
C	0.44349581	-1.40423415	-0.54404896
C	0.80129104	-1.64689620	0.75899646
C	0.33645092	-2.96051272	1.07734962
C	-0.27345531	-3.43781477	-0.04939796
O	-0.21702869	-2.51058253	-1.05100026
C	1.64907521	1.99091468	0.21136867
H	3.20775453	3.16842720	1.34290865
H	4.91727644	1.48612387	-0.02857070
H	-1.90434721	0.00539163	0.33370229
H	-3.74577918	1.95960412	-0.24941564
H	-2.67933483	3.28001641	-2.42940827
H	1.34181823	-0.96184213	1.40324573
H	0.44424486	-3.48407535	2.02143519
H	-0.76973052	-4.36896042	-0.29733474
H	0.69838988	2.41659144	0.51386580

S9.4 Cartesian Coordinates of Optimized Radical Cation Structures

The Cartesian coordinates of the geometry-optimized radical cation structures for all 51 phosphines are provided below. Each structure was treated as a doublet radical cation (charge = +1, spin multiplicity = 2). The reported energies are the SCF electronic energies obtained from these DFT calculations and are given in electronvolts (eV). Note that these energies are not derived from PFP.

Compound **1** (radical cation)
Energy: -18960.648869805806 eV

Atom	X (Å)	Y (Å)	Z (Å)
C	-0.86413400	1.69829500	1.55473400
C	-0.33242100	1.67846300	0.10841900
C	-1.27250700	2.39740000	-0.87636600

P	0.04535400	-0.04858800	-0.44211600
C	1.82369500	-0.49082300	-0.22019900
C	2.16863600	-0.54068100	1.28412900
C	2.73784100	0.46230200	-1.00460100
C	-1.11330600	-1.33157900	0.21396300
C	-2.54805300	-1.07869100	-0.27946200
C	-0.62036200	-2.74516700	-0.12784200
H	-1.85049400	1.23440400	1.62959200
H	-0.19023400	1.20640600	2.26005700
H	-0.96702800	2.74261800	1.86457100
H	0.64683300	2.17017600	0.07771200
H	-0.86465800	2.42069700	-1.88958800
H	-2.25874300	1.92851400	-0.91119500
H	-1.40597600	3.42991400	-0.54246900
H	1.90849700	-1.49756400	-0.64360300
H	2.09844300	0.44893600	1.74325400
H	3.20380700	-0.88082700	1.38604400
H	1.53409100	-1.23485200	1.84094300
H	2.69417600	1.48300200	-0.61423600
H	2.49545200	0.48413900	-2.06987100
H	3.77101800	0.11968100	-0.90316300
H	-1.07709400	-1.18636900	1.30414200
H	-2.61723200	-1.17770800	-1.36605500
H	-3.20886000	-1.82586900	0.16766900
H	-2.92385300	-0.09374100	0.00486300
H	-1.32899600	-3.46925500	0.28291500
H	0.35912600	-2.96545600	0.30247300
H	-0.57491700	-2.90514800	-1.20896300

Compound **2** (radical cation)
Energy: -22170.70905132365 eV

Atom	X (Å)	Y (Å)	Z (Å)
C	-2.83454500	-3.01906800	0.87065900
C	-1.52041800	-2.31011500	0.54578400
C	-1.26639000	-2.19122600	-0.96058800
C	0.05381900	-1.48209800	-1.29697200
P	0.13469900	0.22336300	-0.63698200
C	-1.28775500	1.23983100	-1.17747600
C	-1.28791600	2.65142300	-0.56747800
C	-1.28067400	2.66716200	0.96754300
C	-2.48710900	1.97747100	1.60815700
C	1.73674600	1.03182600	-0.99744300
C	2.94585700	0.20451500	-0.53702800
C	2.94234500	-0.08585100	0.96750000
C	4.16667300	-0.88144300	1.41897700
H	-2.98877400	-3.08572100	1.95036000
H	-2.83876900	-4.03735900	0.46950100
H	-3.68932500	-2.48672000	0.44087800
H	-1.52872000	-1.30333100	0.99623900
H	-0.68019400	-2.83908900	1.01288200

H	-2.10280900	-1.67600400	-1.44708700
H	-1.22603400	-3.18975200	-1.40910900
H	0.21455000	-1.40246500	-2.38203500
H	0.91009800	-2.02314500	-0.88110000
H	-2.19159200	0.68582200	-0.90959500
H	-1.24183000	1.27318400	-2.27568600
H	-0.43418700	3.21778000	-0.95485100
H	-2.18462900	3.16066400	-0.93733100
H	-0.35130600	2.20248900	1.33531500
H	-1.23746500	3.71015700	1.29659100
H	-2.46125900	2.08247600	2.69543400
H	-3.42457300	2.41778000	1.25350100
H	-2.51959500	0.90402700	1.39003300
H	1.71450700	2.00881400	-0.50331800
H	1.75200800	1.21624300	-2.08165000
H	2.99629500	-0.73219800	-1.10428400
H	3.84744300	0.76691200	-0.80303000
H	2.88468000	0.86171800	1.51778500
H	2.02781000	-0.64330600	1.23099700
H	4.13328200	-1.07848200	2.49323800
H	5.08898800	-0.33084100	1.20919600
H	4.22712000	-1.84464300	0.90202700

Compound **3** (radical cation)
Energy: -15750.467718312724 eV

Atom	X (Å)	Y (Å)	Z (Å)
C	2.81667400	-0.47843800	-0.16683900
C	1.51745000	-1.00221100	0.45830200
P	0.00000200	-0.42025900	-0.38323900
C	-1.51744700	-1.00229100	0.45824600
C	-2.81666900	-0.47839900	-0.16679500
C	-0.00003700	1.38227000	-0.67388600
C	0.00002400	2.17762400	0.65017700
H	2.88419100	0.61084000	-0.11287700
H	3.66579900	-0.88941900	0.38342000
H	2.91587400	-0.78509400	-1.21087700
H	1.47843700	-2.09688000	0.45337900
H	1.41905000	-0.68766000	1.50767700
H	-1.47845100	-2.09696000	0.45315000
H	-1.41901600	-0.68790800	1.50766800
H	-2.88417800	0.61086900	-0.11263100
H	-3.66579400	-0.88947900	0.38339200
H	-2.91588100	-0.78486200	-1.21088900
H	0.88100500	1.61346200	-1.27959000
H	-0.88115100	1.61344100	-1.27949800
H	-0.00005300	3.24405700	0.40979700
H	-0.88851700	1.96903500	1.25069400
H	0.88868800	1.96912400	1.25054400

Compound **4** (radical cation)
Energy: -25281.681064197885 eV

Atom	X (Å)	Y (Å)	Z (Å)
C	3.86587500	-0.88946300	0.44698400
C	3.46931600	-2.25632300	-0.13681800
C	1.91642700	-2.30548800	-0.08097600
C	1.48063900	-0.84691200	0.27189900
P	-0.04476000	-0.33089100	-0.58780600
C	-0.49740000	1.42238000	-0.35358600
C	-0.29009000	1.95883600	1.11134100
C	0.36570800	3.35194600	0.93668600
C	0.12152000	3.73932200	-0.52862300
C	0.26824600	2.40819700	-1.27709500
C	-1.43252000	-1.46190900	-0.23954400
C	-2.64773400	-1.26015200	-1.19524700
C	-3.87574700	-1.38451700	-0.27829400
C	-3.41575800	-0.72479600	1.02639200
C	-1.98669100	-1.25440400	1.22513900
C	2.72174200	0.03681800	0.01106800
H	3.90626500	-0.93354200	1.54136800
H	4.84011100	-0.54271800	0.09542900
H	3.92127900	-3.09037600	0.40345800
H	3.80170500	-2.32559300	-1.17662500
H	1.54992400	-2.99822200	0.67978600
H	1.50698800	-2.63728400	-1.04017700
H	1.23244800	-0.79738200	1.34203800
H	-1.56173000	1.46706400	-0.60625100
H	-1.26507600	2.01820500	1.60126400
H	0.33376800	1.30551600	1.72538700
H	1.44191100	3.28031400	1.12495700
H	-0.03985700	4.07846300	1.64445900
H	0.81692700	4.49993500	-0.89052900
H	-0.89430900	4.12931100	-0.65965200
H	1.32659000	2.13712700	-1.34488800
H	-0.13991000	2.41758200	-2.29029000
H	-1.01920800	-2.46838000	-0.34607200
H	-2.63283200	-1.98224900	-2.01378400
H	-2.63336500	-0.26254500	-1.64937100
H	-4.76383800	-0.91790500	-0.71027200
H	-4.10838900	-2.44062700	-0.10282700
H	-3.40476200	0.36626300	0.91259100
H	-4.05593800	-0.95630200	1.88121700
H	-1.37503200	-0.59708800	1.84869300
H	-1.99571900	-2.23312100	1.71324700
H	2.81508400	0.26213000	-1.05745400
H	2.68715500	0.98359100	0.55543800

Compound **5** (radical cation)
Energy: -18960.718404909167 eV

Atom	X (Å)	Y (Å)	Z (Å)
C	-0.56566300	2.34093900	-0.19165100
P	-0.48993900	0.61249500	-0.75402100
C	2.05101500	-0.41594600	0.20632000
C	1.20952000	0.00603800	-1.03648600
C	3.39338400	-0.95316400	-0.31000800
C	2.25495600	0.72690800	1.20324400
C	-1.64938100	-0.52732200	0.15076900
C	-3.08865000	-0.06442200	-0.14869000
C	-1.35798800	-0.43107500	1.66486000
C	-1.41606500	-1.95551600	-0.37138700
H	0.07608600	2.95350400	-0.82911100
H	-1.59271300	2.70549800	-0.26076500
H	-0.22099500	2.42391900	0.84390000
H	1.52470900	-1.23728500	0.70636600
H	1.72234800	0.80606500	-1.58374700
H	1.11320200	-0.84164000	-1.72300600
H	3.96133500	-0.16697100	-0.81818500
H	3.25669500	-1.78374300	-1.00821000
H	3.99586400	-1.31313300	0.52845700
H	2.69193900	1.60472400	0.71511700
H	1.32445500	1.03077300	1.69201800
H	2.94140900	0.41545600	1.99440700
H	-3.77988200	-0.72877800	0.37832300
H	-3.32252600	-0.11582200	-1.21520400
H	-3.27997900	0.95189700	0.20656100
H	-1.52020300	0.57703000	2.05472200
H	-0.34633800	-0.75340700	1.91877100
H	-2.05822500	-1.09799800	2.17937900
H	-2.11903400	-2.62558200	0.13202700
H	-0.40720000	-2.31696500	-0.15649600
H	-1.59864800	-2.03361800	-1.44683700

Compound **6** (radical cation)
Energy: -12540.29887619395 eV

Atom	X (Å)	Y (Å)	Z (Å)
C	1.71475000	0.32028900	0.15368600
P	0.00001100	0.00000700	-0.34032400
C	-1.13476900	1.32484200	0.15368400
C	-0.57999500	-1.64513500	0.15368100
H	1.77756000	0.33184000	1.24976700
H	2.03840900	1.28877700	-0.23498800
H	2.36621900	-0.46590900	-0.23525500
H	-2.13535100	1.12078800	-0.23489000
H	-1.17606500	1.37348600	1.24976600
H	-0.77976100	2.28214400	-0.23534300
H	-1.58662300	-1.81619300	-0.23521100
H	0.09687000	-2.40968800	-0.23504700
H	-0.60133600	-1.70532100	1.24976000

Compound **7** (radical cation)
Energy: -28492.260445273972 eV

Atom	X (Å)	Y (Å)	Z (Å)
C	-3.76381500	-2.67510700	-0.37165700
C	-3.49121600	-1.24272400	-0.88445900
C	-2.81682000	-0.39469000	0.19708300
C	-1.41338200	-0.96565600	0.53983200
P	-0.09395100	-0.17191100	-0.46990000
C	1.50832300	-1.05826600	-0.33599500
C	2.06617300	-0.93062400	1.10822400
C	3.38138900	-1.71956300	1.22509000
C	4.40886900	-1.26342600	0.18428300
C	3.84617200	-1.36949300	-1.23750600
C	2.53363800	-0.58525400	-1.38658400
C	0.09464000	1.64120400	-0.19732500
C	-0.28410600	2.04927600	1.24787900
C	-0.01655800	3.54943100	1.44945400
C	-0.76192700	4.40027600	0.41569700
C	-0.40188200	3.98021100	-1.01381200
C	-0.66386400	2.48624400	-1.24854700
C	-1.35148600	-2.51052200	0.38972000
C	-2.72754700	-3.12083300	0.68226700
H	-3.75698100	-3.36935200	-1.21765800
H	-4.76552200	-2.72836400	0.06529200
H	-4.42071400	-0.76755600	-1.20862000
H	-2.83974200	-1.27160000	-1.76862800
H	-2.75891600	0.65522900	-0.10231900
H	-3.41886100	-0.41350100	1.11147600
H	-1.15723500	-0.68798000	1.57253600
H	1.26811400	-2.11139500	-0.52529400
H	1.33783000	-1.29631800	1.83930000
H	2.25489100	0.12632500	1.33228000
H	3.16932700	-2.78840300	1.09509500
H	3.77424000	-1.59588300	2.23954400
H	4.69505500	-0.22279800	0.38739400
H	5.32076600	-1.86111400	0.27341200
H	3.66334700	-2.42330500	-1.48435700
H	4.57107900	-0.99760600	-1.96767600
H	2.12800700	-0.70853600	-2.39563900
H	2.73732600	0.48417600	-1.24808900
H	1.16999300	1.81115800	-0.34069000
H	-1.34702100	1.84483100	1.41845400
H	0.28231500	1.46121500	1.97716900
H	-0.31370800	3.82577800	2.46625800
H	1.06262100	3.73577200	1.37462500
H	-1.84405300	4.29048400	0.56674800
H	-0.53104700	5.45890500	0.56614100
H	0.65862800	4.19213500	-1.20363500
H	-0.97309100	4.56306700	-1.74266800

H	-0.35607700	2.19724300	-2.25825900
H	-1.73954300	2.28723200	-1.17286200
H	-1.06110000	-2.78936800	-0.63099100
H	-0.58655500	-2.91616400	1.05792000
H	-2.64798900	-4.21098400	0.70398600
H	-3.04571300	-2.81755300	1.68639000

Compound **8** (radical cation)
Energy: -17758.764552750934 eV

Atom	X (Å)	Y (Å)	Z (Å)
C	-2.37100500	-1.49764100	0.28038600
P	-1.56727600	-0.00005300	-0.37957500
C	-2.37102200	1.49767300	0.28003500
C	0.18406400	-0.00000000	-0.14299000
C	0.89289000	-1.22615200	-0.09064200
C	2.26992800	-1.21736000	0.06437600
C	2.95989600	0.00002100	0.14196700
C	2.26988300	1.21738600	0.06458300
C	0.89284000	1.22616200	-0.09042500
H	-3.44237100	-1.43460300	0.07682200
H	-1.97717900	-2.39106200	-0.20725200
H	-2.20706700	-1.56529900	1.36094400
H	-2.20687800	1.56576200	1.36053300
H	-3.44242400	1.43446700	0.07671000
H	-1.97738600	2.39094100	-0.20804200
H	0.36727400	-2.17206500	-0.15654100
H	2.81221800	-2.15440800	0.12334700
H	4.03877300	0.00002500	0.25578100
H	2.81212900	2.15444900	0.12372300
H	0.36719600	2.17207200	-0.15613200

Compound **9** (radical cation)
Energy: -22170.534299031042 eV

Atom	X (Å)	Y (Å)	Z (Å)
C	-2.10091500	0.24459800	1.44568700
C	-1.85154100	-0.17250500	-0.02007000
C	-2.65038900	0.71967700	-0.99615000
C	-2.32738900	-1.62132000	-0.23107800
P	-0.00033800	0.00059800	-0.41700200
C	0.77659200	1.68927700	-0.01998800
C	-0.23883200	2.82693000	-0.23115800
C	1.94893600	1.93387000	-0.99591300
C	1.26122800	1.69540800	1.44610000
C	1.07466600	-1.51646100	-0.02007500
C	0.84024300	-1.93952500	1.44659200
C	0.70038300	-2.65493900	-0.99505700
C	2.56731500	-1.20560000	-0.23277700
H	-1.84475700	1.28635500	1.64038800

H	-3.17158600	0.12895400	1.64635000
H	-1.56173500	-0.38753300	2.15434700
H	-2.44992100	0.45846100	-2.03866000
H	-2.46982800	1.78286500	-0.85990400
H	-3.71430400	0.54224100	-0.81081500
H	-2.17992700	-1.95896000	-1.25886000
H	-3.40352300	-1.63934300	-0.03370500
H	-1.86410300	-2.33212000	0.45262200
H	-1.08673800	2.78105300	0.45161300
H	-0.60370200	2.86947200	-1.25940800
H	0.28424900	3.76726900	-0.03230900
H	2.32800200	2.94379600	-0.81116800
H	1.62241900	1.89031200	-2.03839700
H	2.77866800	1.24525100	-0.85918700
H	0.44288300	1.54508300	2.15342500
H	1.69739700	2.67978400	1.64778300
H	2.03403400	0.95147500	1.64133300
H	1.47575300	-2.80897900	1.64685500
H	-0.18956100	-2.23799200	1.64416600
H	1.12021700	-1.15589200	2.15369500
H	-0.30993500	-3.03077500	-0.85626600
H	0.82359200	-2.35082800	-2.03793800
H	1.38710700	-3.48703400	-0.81117000
H	3.12008500	-2.12904800	-0.03515100
H	2.95316000	-0.44886800	0.44984600
H	2.78533000	-0.91043300	-1.26113000

Compound **10** (radical cation)
Energy: -24178.884641386907 eV

Atom	X (Å)	Y (Å)	Z (Å)
C	-1.90731500	-1.66193700	1.32000100
C	-1.51145600	-1.53339300	-0.16442100
C	-0.61894400	-2.71039000	-0.60248700
C	-2.77171000	-1.49853600	-1.05599200
P	-0.55069700	0.04963200	-0.48062300
C	1.21839800	-0.08789800	-0.21167500
C	1.75088900	-0.80160000	0.88089200
C	3.12784700	-0.84540300	1.07067100
C	3.98287900	-0.20154300	0.17155500
C	3.46211500	0.49782400	-0.91874000
C	2.08535500	0.56027400	-1.11326900
C	-1.19209000	1.68801400	0.19289900
C	-0.81490800	1.75174200	1.68919900
C	-2.71118800	1.79755600	-0.00063000
C	-0.47581000	2.81159400	-0.57972300
H	-1.04360000	-1.67162800	1.98876400
H	-2.58701700	-0.86898800	1.63793800
H	-2.43236500	-2.61491600	1.44555900
H	0.26746000	-2.82558100	0.02168700
H	-1.21021500	-3.62748600	-0.52077200

H	-0.29746200	-2.61455700	-1.64323100
H	-3.45614300	-0.69267700	-0.79010900
H	-3.30637400	-2.44399100	-0.92236200
H	-2.51706000	-1.40812700	-2.11528100
H	1.09874800	-1.29692000	1.59027400
H	3.53485300	-1.38125800	1.92137600
H	5.05627400	-0.24724600	0.32159300
H	4.12696200	0.99051700	-1.61965800
H	1.68625900	1.09429500	-1.96907300
H	-1.30573900	0.97447200	2.27826100
H	-1.14946400	2.72156500	2.07391400
H	0.26444700	1.68459400	1.84119500
H	-3.00074800	1.71097300	-1.05089100
H	-3.02686900	2.78539100	0.34862100
H	-3.26087500	1.05619200	0.58256000
H	-0.82382000	3.77052800	-0.18348500
H	0.60777700	2.77511800	-0.44954900
H	-0.70894300	2.78743800	-1.64764800

Compound **11** (radical cation)
Energy: -35010.88685460648 eV

Atom	X (Å)	Y (Å)	Z (Å)
C	-2.08215900	1.11723800	-2.45333700
C	-0.80259600	1.72231000	-1.87343600
C	0.22708500	2.06522000	-2.95332000
C	1.49616600	2.75585800	-2.43564900
C	2.33742000	1.93568600	-1.44491600
C	2.70950100	0.54554800	-1.97361300
C	3.84377700	-0.14411700	-1.20636100
C	3.63226200	-0.24504800	0.30943700
P	2.11418800	-1.11188900	0.87429700
C	1.92403200	-2.79687700	0.16615300
C	1.40927500	-2.86875000	-1.28120500
C	-0.04572400	-2.41388900	-1.45806900
C	-1.07413800	-3.39098300	-0.87275000
C	-2.53191600	-2.99467700	-1.14737000
C	-3.02962500	-1.81765100	-0.30490400
C	-4.45667000	-1.38067600	-0.64521800
C	-4.96048000	-0.24473900	0.24707500
C	2.19205100	-1.22796800	2.70151500
C	0.85191400	-1.45415200	3.42605600
C	-0.09123800	-0.24660100	3.34601300
C	-0.72233900	-0.05259300	1.96928500
C	-1.49417100	1.25587000	1.77745600
C	-0.61206200	2.50666200	1.74716400
C	-1.39727600	3.79126200	1.46810600
C	-0.49389300	5.01618800	1.32032000
H	-2.83252400	0.93698800	-1.67926300
H	-2.52829100	1.78683900	-3.19548900
H	-1.87937500	0.16226300	-2.95111700

H	-1.04612900	2.63187900	-1.31043400
H	-0.36613000	1.02519300	-1.14418400
H	0.49256200	1.15343700	-3.50479300
H	-0.24631900	2.72574400	-3.68951400
H	1.22187600	3.70574600	-1.96026200
H	2.12938500	3.01473100	-3.29307600
H	3.25317800	2.49690100	-1.21716400
H	1.79586500	1.83794200	-0.49444900
H	1.81731200	-0.09423600	-1.97220600
H	3.01640400	0.62329700	-3.02285700
H	4.02825800	-1.14218200	-1.61743100
H	4.77265500	0.41958100	-1.35028900
H	3.58720500	0.74732800	0.76992000
H	4.46300200	-0.78234900	0.79023800
H	1.25662700	-3.33284800	0.84818600
H	2.91950300	-3.25491200	0.25679600
H	2.07039400	-2.29346300	-1.93405700
H	1.50343900	-3.91308100	-1.59988500
H	-0.23966700	-2.30473300	-2.53117200
H	-0.17821600	-1.41157000	-1.02790200
H	-0.93231400	-3.49469600	0.21287800
H	-0.88203100	-4.38274500	-1.30072100
H	-2.64457200	-2.75807700	-2.21396800
H	-3.17742500	-3.86056100	-0.95984500
H	-2.98510900	-2.09819500	0.75763700
H	-2.35776800	-0.95904000	-0.42658500
H	-5.12870300	-2.24345200	-0.55670300
H	-4.49572400	-1.07078400	-1.69735400
H	-4.95040800	-0.53772100	1.30287800
H	-4.33452900	0.64948600	0.14755300
H	-5.98456900	0.04057000	-0.00890700
H	2.66384100	-0.30461600	3.05485500
H	2.88934400	-2.05138000	2.91128300
H	0.35598900	-2.35159900	3.03567400
H	1.09298500	-1.66850900	4.47161400
H	-0.89463600	-0.38209500	4.07927900
H	0.45335100	0.65384400	3.65375600
H	0.07359500	-0.07461300	1.18506400
H	-1.36065000	-0.91210600	1.74227200
H	-2.24201600	1.34344600	2.57759500
H	-2.05703400	1.19464100	0.84027400
H	-0.06988000	2.61834500	2.69502500
H	0.15311500	2.38051500	0.96747000
H	-1.99065000	3.65805800	0.55414000
H	-2.12018800	3.95569700	2.27685900
H	0.20769500	4.88912200	0.48743900
H	0.09593600	5.18642100	2.22752300
H	-1.07665500	5.92116000	1.12837200

Compound **12** (radical cation)
Energy: -41135.790987448825 eV

Atom	X (Å)	Y (Å)	Z (Å)
C	2.95841300	-3.09516500	1.31888100
C	3.24786100	-2.69290300	-0.13642700
C	2.78746600	-3.80708900	-1.08802200
C	1.27502100	-4.01036200	-0.91853200
C	0.96110200	-4.41451000	0.53319400
C	1.44745300	-3.31443200	1.49259100
C	0.69773400	-1.99746100	1.19162300
C	0.97504500	-1.57538500	-0.28007300
P	-0.00016600	0.00001700	-0.68212300
C	-1.85218000	-0.05676300	-0.27998800
C	-2.07900900	0.39631000	1.19104300
C	-3.59433200	0.40563700	1.49195900
C	-4.30416000	1.37550600	0.53139700
C	-4.11104100	0.89989600	-0.91974600
C	-4.69097600	-0.51197100	-1.08741100
C	-3.95615100	-1.46640900	-0.13454300
C	-4.15977200	-1.01279600	1.32019700
C	-2.44799800	-1.47609500	-0.46678200
C	-2.60621900	0.88743700	-1.26086200
C	0.87679200	1.63203600	-0.27991000
C	-0.05398000	2.85786200	-0.46811300
C	0.70850500	4.15915000	-0.13591700
C	1.20206100	4.10921700	1.31924900
C	2.14743400	2.91024200	1.49235100
C	3.34299500	3.03913700	0.53262000
C	2.83553700	3.10924700	-0.91885500
C	1.90340100	4.31765300	-1.08777100
C	2.07217100	1.81241000	-1.26005100
C	1.38167400	1.60252200	1.19154300
C	2.50196700	-1.38201400	-0.46859200
C	0.53333000	-2.70099200	-1.25977300
H	3.31071300	-2.31760100	2.00779600
H	3.50160300	-4.01302200	1.56821400
H	4.31969500	-2.50865900	-0.26595100
H	3.32204800	-4.73677600	-0.86486100
H	3.02173200	-3.54288900	-2.12609500
H	0.92137400	-4.78718600	-1.60467100
H	1.45673900	-5.36152800	0.77201000
H	-0.11642300	-4.57810200	0.65871200
H	1.22516800	-3.59602800	2.52775800
H	1.03180800	-1.21367800	1.87845000
H	-0.37055900	-2.14565500	1.36203500
H	-1.56705400	-0.28391800	1.87874000
H	-1.67350400	1.39591700	1.35999400
H	-3.72712500	0.74029700	2.52668700
H	-5.37210700	1.41986300	0.77026900
H	-3.90728900	2.39070700	0.65566400
H	-4.60706400	1.59357600	-1.60682100
H	-4.57919900	-0.84830500	-2.12504200
H	-5.76341000	-0.50985500	-0.86433100

H	-4.33239200	-2.48697100	-0.26279600
H	-3.66249700	-1.70575000	2.01001900
H	-5.22626400	-1.02407300	1.56950500
H	-1.95336200	-2.19856300	0.18454800
H	-2.30874300	-1.80524600	-1.50120400
H	-2.45767500	0.53709000	-2.28849600
H	-2.22490500	1.90491400	-1.20648700
H	-0.40793000	2.90151000	-1.50281600
H	-0.92745300	2.79119300	0.18254400
H	0.01310400	4.99537800	-0.26507400
H	0.35276800	4.02537900	2.00845800
H	1.72544600	5.03849000	1.56865800
H	2.50281100	2.85842800	2.52739700
H	3.91555100	3.94173700	0.77145100
H	4.02333900	2.18763800	0.65778800
H	3.68483200	3.19143100	-1.60533400
H	2.44152900	5.24525100	-0.86444300
H	1.55724300	4.38874800	-2.12575100
H	1.69517400	1.85879500	-2.28793800
H	2.76243300	0.97338200	-1.20501100
H	2.04438200	0.75147300	1.36128700
H	0.53622500	1.49975100	1.87883100
H	2.71658300	-1.09753200	-1.50335700
H	2.88098200	-0.59213300	0.18201100
H	0.76166100	-2.39789700	-2.28779500
H	-0.53838000	-2.87933500	-1.20439300

Compound **13** (radical cation)
Energy: -37545.70831214864 eV

Atom	X (Å)	Y (Å)	Z (Å)
C	4.00692300	5.00985100	1.21403200
O	2.82811500	4.98567100	0.40247700
C	2.24979600	3.81024000	0.12159000
C	2.70646100	2.56261600	0.58578200
C	2.02677800	1.40577500	0.23264900
C	0.87668300	1.46490700	-0.57710600
P	-0.00036400	0.00012200	-1.08932500
C	-1.70743600	0.02702600	-0.57708800
C	-2.23138400	1.05203700	0.23330600
C	-3.57310700	1.06188600	0.58639700
C	-4.42511700	0.04276900	0.12156000
O	-5.73225700	-0.04442100	0.40220600
C	-6.34285900	0.96372600	1.21436600
C	-3.91109400	-0.98116900	-0.70249900
C	-2.57897400	-0.98120300	-1.05673200
C	0.82989900	-1.49186700	-0.57761800
C	0.20426400	-2.45827400	0.23268500
C	0.86683000	-3.62499000	0.58591200
C	2.17550600	-3.85303000	0.12121200
O	2.90490900	-4.94110400	0.40237600

C	2.33736800	-5.97368500	1.21506200
C	2.80502900	-2.89597200	-0.70308200
C	2.13878800	-1.74246100	-1.05741300
C	0.43929400	2.72399900	-1.05625700
C	1.10567400	3.87745200	-0.70196200
H	4.81728600	4.44402300	0.74330100
H	4.28809000	6.05876800	1.28972500
H	3.80248900	4.61045900	2.21278800
H	3.57646600	2.49342800	1.22573500
H	2.37814800	0.45043000	0.60521600
H	-1.57983000	1.83393200	0.60626900
H	-3.94832200	1.84955900	1.22672500
H	-5.89473600	0.98596100	2.21311800
H	-6.25835600	1.94871900	0.74416600
H	-7.39174200	0.68239800	1.28993800
H	-4.59084100	-1.74842300	-1.05461800
H	-2.19959700	-1.76327100	-1.70694600
H	-0.79870800	-2.28517000	0.60559600
H	0.37250800	-4.34380100	1.22638200
H	3.10557500	-6.74121900	1.29096900
H	1.44209600	-6.39337700	0.74518500
H	2.09408800	-5.59618200	2.21362300
H	3.80941600	-3.10086400	-1.05514600
H	2.62627600	-1.02288900	-1.70772300
H	-0.42791800	2.78679900	-1.70612200
H	0.78101900	4.84991800	-1.05357200

Compound **14** (radical cation)
Energy: -23240.848885927477 eV

Atom	X (Å)	Y (Å)	Z (Å)
C	2.64947200	1.29313200	-0.82308600
C	2.54813600	-0.00460700	-0.01070600
C	3.67541800	-0.04596700	1.03660400
C	2.67680700	-1.21008000	-0.95216900
C	1.20542100	-0.07083200	0.78372000
P	-0.36311400	-0.00115000	-0.17892100
C	-1.30963200	1.62139400	-0.00008700
C	-0.30922900	2.77896300	0.15556800
C	-2.14952000	1.84980900	-1.27277700
C	-2.20928200	1.54075300	1.25179800
C	-1.33109900	-1.60551400	-0.00530600
C	-2.65851500	-1.52349700	-0.77671500
C	-1.58881300	-1.88613500	1.49277000
C	-0.46097600	-2.73030000	-0.59987100
H	1.86705900	1.35139700	-1.58973800
H	3.61104000	1.33365700	-1.34139600
H	2.57926000	2.17799100	-0.18490900
H	3.63425500	-0.96249600	1.63355700
H	3.62086700	0.80913000	1.71765600
H	4.64890400	-0.01538100	0.53954500

H	2.64174900	-2.15686300	-0.40503600
H	3.63306900	-1.17040700	-1.48027900
H	1.88581500	-1.21919100	-1.71086000
H	1.16228200	0.75153500	1.50537500
H	1.17054700	-1.00659800	1.34989600
H	0.30504900	2.69244800	1.05446500
H	-0.88709200	3.70370600	0.24579000
H	0.34400500	2.88159000	-0.71162300
H	-2.90177800	1.07706300	-1.42976700
H	-1.52038300	1.91244900	-2.16454300
H	-2.67361900	2.80470200	-1.16781000
H	-1.63828600	1.33819600	2.16219700
H	-2.99905100	0.79484300	1.15347500
H	-2.69234200	2.51592500	1.37606100
H	-3.34888800	-0.79523100	-0.34807000
H	-3.14359500	-2.50260700	-0.72109800
H	-2.50707100	-1.29097000	-1.83386000
H	-0.66427400	-1.97807300	2.06754400
H	-2.22054000	-1.13141800	1.96177600
H	-2.11054900	-2.84679500	1.56143400
H	0.48728400	-2.84903600	-0.07287300
H	-0.25464500	-2.57436300	-1.66143300
H	-1.01148300	-3.67067300	-0.50012200

Compound **15** (radical cation)
Energy: -22977.03652318954 eV

Atom	X (Å)	Y (Å)	Z (Å)
C	-0.06909800	2.74434500	0.34538100
P	-0.02716000	1.12161500	-0.48984400
C	-1.49500800	0.17165300	-0.13567900
C	-1.43198800	-1.12864100	0.40902700
C	-2.60960100	-1.81883300	0.66787300
C	-3.84723200	-1.23671700	0.37490100
C	-3.91429500	0.04754800	-0.17671300
C	-2.74720100	0.75069400	-0.44096300
C	1.48981700	0.24422800	-0.20341000
C	2.53859700	0.83033500	0.53688500
C	3.72858400	0.13479300	0.70934800
C	3.89468900	-1.12911100	0.13270100
C	2.86868700	-1.70383100	-0.62551400
C	1.67355000	-1.02150000	-0.80701300
H	-1.01398900	3.23758600	0.10952500
H	0.75280100	3.36711600	-0.01231200
H	0.00076500	2.61087500	1.42934600
H	-0.47622800	-1.57395200	0.65695500
H	-2.56510500	-2.80895200	1.10828200
H	-4.76160700	-1.78437200	0.57701100
H	-4.87602600	0.49298400	-0.40576900
H	-2.80440500	1.73828900	-0.88810700
H	2.41907100	1.80768100	0.99030700

H	4.52840200	0.57500800	1.29459800
H	4.82968700	-1.66276600	0.26621800
H	3.00884400	-2.67689500	-1.08307200
H	0.88817300	-1.45661600	-1.41625800

Compound **16** (radical cation)
Energy: -34945.35216414957 eV

Atom	X (Å)	Y (Å)	Z (Å)
C	3.62648400	-2.20981600	1.47135600
C	2.24609200	-2.73956700	1.05325100
C	1.63903800	-3.52428300	2.23110200
C	2.40293900	-3.68805100	-0.14823400
C	1.24649800	-1.60713100	0.70561100
C	1.57418100	-0.64694700	-0.46033100
C	0.25373200	-0.21730900	-1.15551900
P	-1.05840100	0.45824200	-0.04777100
C	-1.46206200	2.23380600	-0.41980300
C	-0.28501000	3.17277400	-0.09539400
C	-0.04734100	3.28926500	1.41533400
C	-1.31938100	3.71278200	2.15964200
C	-2.49823700	2.78630800	1.83707300
C	-2.73266800	2.68501400	0.32449200
C	-2.59331200	-0.55979800	-0.09233000
C	-2.38785400	-2.02530800	0.33355900
C	-3.72625300	-2.77881900	0.30305000
C	-4.38664500	-2.69506600	-1.07723200
C	-4.56957200	-1.23941500	-1.51658300
C	-3.23973400	-0.46637300	-1.50231700
C	2.42041000	0.56507800	-0.01945900
C	3.39557600	1.19751700	-1.04936900
C	3.94568000	2.49190400	-0.42409500
C	4.56882500	0.23950200	-1.32224400
C	2.72371600	1.54071900	-2.39032100
H	4.26177600	-3.03910600	1.79725000
H	4.14089100	-1.71246300	0.64661500
H	3.55034500	-1.50564900	2.30694400
H	0.65833000	-3.93851000	1.97099500
H	2.28718100	-4.35850800	2.51487000
H	1.51435700	-2.88431900	3.11134400
H	2.87578600	-3.19588300	-1.00412100
H	1.43324100	-4.08136400	-0.47417400
H	3.03287500	-4.54117800	0.12010900
H	0.30042200	-2.11297400	0.48386100
H	1.06573300	-1.00226000	1.60762700
H	2.12211300	-1.20394500	-1.22739200
H	-0.20622000	-1.08355200	-1.64362000
H	0.43156600	0.54678300	-1.91574900
H	-1.63823700	2.22420300	-1.50656200
H	0.62340300	2.85768600	-0.61400500
H	-0.54524600	4.15650900	-0.50400700

H	0.29484100	2.31880800	1.80579600
H	0.76432400	3.99788700	1.60491500
H	-1.13711600	3.73036300	3.23827200
H	-1.58175400	4.73847600	1.86986900
H	-2.29625500	1.78345300	2.24623100
H	-3.41008000	3.13791000	2.32846900
H	-3.58431900	2.03833000	0.09567600
H	-2.99065300	3.67528600	-0.07013600
H	-3.26286200	-0.08011800	0.63166700
H	-1.94414100	-2.07438200	1.33224700
H	-1.68847300	-2.51373900	-0.35585200
H	-4.39646100	-2.35237600	1.06067700
H	-3.55605500	-3.82184200	0.58634400
H	-3.76335300	-3.22161400	-1.81223000
H	-5.35397000	-3.20591500	-1.06450600
H	-5.28050100	-0.73655600	-0.84849400
H	-4.99279900	-1.18743500	-2.52500700
H	-3.41018000	0.57609700	-1.78726300
H	-2.55588900	-0.89953300	-2.24257500
H	3.01815000	0.27336100	0.84717500
H	1.74386700	1.34349300	0.35493300
H	4.68850500	2.95707900	-1.07895700
H	4.42711800	2.29158500	0.53861400
H	3.14695600	3.22316700	-0.25409200
H	5.13414800	0.03759600	-0.40751800
H	4.23175600	-0.71869600	-1.73172500
H	5.25967500	0.67771900	-2.04884400
H	2.37547500	0.64655300	-2.91799900
H	1.87717300	2.22622200	-2.26623300
H	3.43996800	2.03901500	-3.05032200

Compound **17** (radical cation)
Energy: -31405.99510901984 eV

Atom	X (Å)	Y (Å)	Z (Å)
C	5.50802600	2.13107700	-0.67311100
C	4.14584700	1.62540400	-0.29005500
C	4.00631600	0.48302900	0.51764900
C	2.75435100	-0.00222000	0.86147900
C	1.59318300	0.64130500	0.38109700
P	0.00044000	0.00138100	0.86216400
C	-1.35022900	1.05922900	0.37830100
C	-1.37362200	2.38775900	0.85505100
C	-2.41632500	3.23057200	0.50318100
C	-3.47403200	2.77894000	-0.30543400
C	-4.61907200	3.68949600	-0.64873000
C	-3.44729000	1.45055700	-0.76093600
C	-2.40983900	0.59244100	-0.42416600
C	-0.24108000	-1.69816800	0.38174000
C	0.69823600	-2.38814500	-0.40964800
C	0.47663800	-3.71835700	-0.73735000

C	-0.66515100	-4.40146500	-0.28731900
C	-0.90789800	-5.83410600	-0.67016400
C	-1.58784400	-3.70706000	0.51458400
C	-1.38215200	-2.37985800	0.85739900
C	1.72051800	1.79604200	-0.41574100
C	2.98318100	2.26904700	-0.74452400
H	5.88058500	1.59078700	-1.55200800
H	6.23119300	1.97806900	0.13203600
H	5.48586500	3.19348600	-0.92566300
H	4.89419900	-0.01912700	0.88880300
H	2.67036000	-0.87465000	1.50192700
H	-0.57569600	2.75331500	1.49384300
H	-2.42187200	4.25360900	0.86595700
H	-4.28751600	4.72539300	-0.75602000
H	-5.37156200	3.66978800	0.14905800
H	-5.11293000	3.38280300	-1.57348100
H	-4.24947100	1.09058600	-1.39755400
H	-2.40380800	-0.42432500	-0.79927900
H	1.58087400	-1.87865700	-0.77830300
H	1.19713500	-4.23972500	-1.35980500
H	0.02623300	-6.35311200	-0.89638900
H	-1.42632700	-6.37720100	0.12399400
H	-1.53882800	-5.88621200	-1.56597200
H	-2.46916000	-4.22320500	0.88213900
H	-2.09830500	-1.86905100	1.49344700
H	0.83776900	2.30291700	-0.78773900
H	3.07408600	3.15067200	-1.37128800

Compound **18** (radical cation)
Energy: -28393.47959908911 eV

Atom	X (Å)	Y (Å)	Z (Å)
C	1.54597700	4.19040100	0.09683300
C	0.64519900	3.75618200	1.07470500
C	0.04513500	2.50774900	0.96744300
C	0.36578200	1.66516300	-0.11932800
P	-0.37214100	0.04975300	-0.29805200
C	-1.77683500	-0.19434100	0.88392200
C	-2.23606300	-1.66603500	0.89449600
C	-2.89669200	-2.07254500	-0.42856800
C	-4.05900300	-1.13750400	-0.78394100
C	-3.61781800	0.33086000	-0.80199200
C	-2.94850400	0.74340800	0.51509700
C	0.86527000	-1.31943200	-0.20144800
C	1.45284700	-1.37056500	1.23260800
C	2.40567000	-2.57259600	1.35549700
C	3.50859300	-2.53499100	0.29302400
C	2.91870900	-2.44979100	-1.11926100
C	1.97850500	-1.24265400	-1.26100100
C	1.26466500	2.11916200	-1.11095200
C	1.85146900	3.37288800	-0.99535200

H	2.00493500	5.16955800	0.18349600
H	0.41346600	4.39227200	1.92205800
H	-0.64561500	2.18184200	1.73573000
H	-1.37328300	0.07328000	1.87011600
H	-2.96590600	-1.75860000	1.70774700
H	-1.41139100	-2.33763000	1.15133800
H	-3.24122500	-3.10877700	-0.36323500
H	-2.15065200	-2.04907300	-1.23867600
H	-4.48448500	-1.41553100	-1.75272200
H	-4.85885300	-1.26391700	-0.04275000
H	-4.47147700	0.98640000	-0.99699000
H	-2.91331000	0.48933300	-1.63305500
H	-3.67447100	0.68056500	1.33461300
H	-2.61886800	1.78557300	0.47002400
H	0.27300000	-2.22502200	-0.38076200
H	0.65786400	-1.44499100	1.98126700
H	1.99776800	-0.43886700	1.42471600
H	1.82624300	-3.49947500	1.25649600
H	2.83567200	-2.57540400	2.36246700
H	4.15469900	-1.66509900	0.47044900
H	4.14422400	-3.42088100	0.38314300
H	3.71530000	-2.37336300	-1.86541300
H	2.36264400	-3.36886000	-1.34516700
H	1.54920900	-1.20823100	-2.26760500
H	2.55531600	-0.32273800	-1.11415300
H	1.48427700	1.50107000	-1.97397100
H	2.53959100	3.71812500	-1.75907200

Compound **19** (radical cation)
Energy: -56245.8348486424 eV

Atom	X (Å)	Y (Å)	Z (Å)
C	-6.75614000	0.53352000	-0.21769000
O	-5.66856700	1.30751000	-0.72029100
C	-4.41039500	0.97177600	-0.37124300
C	-3.40802800	1.79550800	-0.90344000
C	-2.07462900	1.53824400	-0.61061900
O	-1.05859700	2.27211100	-1.11119900
C	-1.35070500	3.40591400	-1.92149100
C	-1.71312500	0.44529000	0.22317200
P	-0.05174400	0.02066700	0.69901300
C	1.15514500	1.27714700	0.33596200
C	2.27847300	1.11261500	-0.50898100
O	2.40963400	-0.08981700	-1.10916100
C	3.51679500	-0.33236200	-1.96890100
C	3.17732200	2.16121800	-0.72471900
C	2.95873400	3.39032300	-0.09131600
O	3.76173300	4.46340400	-0.23770200
C	4.91311800	4.35800400	-1.07271500
C	1.85828700	3.58630200	0.75887200
C	0.98241200	2.53593000	0.97436300

O	-0.10014300	2.61213700	1.78188000
C	-0.41414900	3.85591100	2.40275200
C	0.44180800	-1.62067600	0.21625600
C	1.57890200	-2.16267200	0.86134600
O	2.16542700	-1.33815400	1.76059700
C	3.35090000	-1.75900400	2.42787100
C	2.05234500	-3.44101300	0.57346800
C	1.35998300	-4.21118900	-0.37111000
O	1.71763300	-5.46228400	-0.72606400
C	2.85397900	-6.05912200	-0.10458300
C	0.22415600	-3.71195700	-1.02471800
C	-0.23178800	-2.43158000	-0.73571000
O	-1.30014100	-1.87631800	-1.34704000
C	-2.02897200	-2.63917400	-2.30291200
C	-2.75518700	-0.35504000	0.75256700
O	-2.34155900	-1.36621000	1.55102900
C	-3.29751600	-2.28514500	2.07042100
C	-4.09472600	-0.11414300	0.45720300
H	-6.79306100	0.56691300	0.87667500
H	-7.65686800	0.98989800	-0.62593800
H	-6.68978300	-0.50737300	-0.55343500
H	-3.70972000	2.60979700	-1.54640200
H	-1.85682400	3.11322900	-2.84769100
H	-1.96857900	4.13048400	-1.38005100
H	-0.38415900	3.85124900	-2.15501600
H	4.46717100	-0.21580700	-1.43604600
H	3.49967200	0.33304100	-2.83903500
H	3.40687100	-1.36549800	-2.29707400
H	4.02059900	2.02610600	-1.38324700
H	5.60039400	3.58890400	-0.70341600
H	4.63219000	4.13579800	-2.10822900
H	5.39962300	5.33150700	-1.02927000
H	1.73872700	4.55137600	1.23000900
H	-1.32933100	3.68061600	2.96670400
H	0.38356800	4.16943900	3.08426100
H	-0.58511100	4.63543400	1.65254200
H	3.16653400	-2.64891000	3.03928100
H	3.63680800	-0.92821600	3.07164500
H	4.15430600	-1.96311100	1.71144900
H	2.92366200	-3.83041900	1.07594800
H	3.76554200	-5.48802200	-0.31349800
H	2.71309100	-6.14567600	0.97841300
H	2.94097600	-7.05349400	-0.54044600
H	-0.26035700	-4.34545300	-1.75374000
H	-2.84601400	-1.99474300	-2.62584400
H	-2.43488800	-3.55273600	-1.85469700
H	-1.40369300	-2.90112400	-3.16318900
H	-4.01240400	-1.78225700	2.73054000
H	-3.83443600	-2.79241000	1.26140700
H	-2.72435900	-3.01355300	2.64248300
H	-4.86934900	-0.74176200	0.86869800

Compound **20** (radical cation)
Energy: -34616.01865208008 eV

Atom	X (Å)	Y (Å)	Z (Å)
C	0.44483500	-6.14650100	1.71287400
C	1.18832600	-5.77827200	0.41488100
C	0.92380600	-4.35276800	0.00907800
C	-0.18203200	-4.02934800	-0.79572500
C	-0.45530500	-2.71513200	-1.14218300
C	0.37090700	-1.67512400	-0.66392800
P	-0.00014400	0.00022000	-1.14748000
C	1.26536500	1.15905900	-0.66401500
C	0.97738100	2.28513100	0.13273700
C	1.99351800	3.17027800	0.46354700
C	3.30820000	2.97640300	0.00891500
C	4.41072400	3.91786900	0.41483400
C	5.10059800	3.45797300	1.71318700
C	3.58100600	1.85637100	-0.79497900
C	2.57936900	0.96271100	-1.14136900
C	-1.63659500	0.51642600	-0.66405300
C	-2.12418600	1.75210100	-1.14197100
C	-3.39900700	2.17237000	-0.79543300
C	-4.23199000	1.37612700	0.00912400
C	-5.59856900	1.85990500	0.41549800
C	-5.54490700	2.68825600	1.71321600
C	-3.74190400	0.14108100	0.46427000
C	-2.46719000	-0.29598200	0.13333700
C	1.48940800	-1.98834800	0.13393500
C	1.74820700	-3.31088800	0.46465000
H	0.75967600	-5.50424900	2.54066100
H	0.65071700	-7.18405200	1.98779400
H	-0.63650900	-6.03354500	1.59082900
H	0.87448600	-6.45174200	-0.38936000
H	2.26405600	-5.92530200	0.55554600
H	-0.82314600	-4.82432800	-1.16393500
H	-1.30226400	-2.48915400	-1.78253500
H	-0.02810200	2.44967100	0.50220900
H	1.76980200	4.02799800	1.09060600
H	5.15124900	3.98213400	-0.38912700
H	4.00066900	4.92326700	0.55499200
H	5.54319500	2.46488000	1.59130600
H	4.38652000	3.40970500	2.54059300
H	5.89627600	4.15480400	1.98850900
H	4.59021500	1.69798600	-1.16240900
H	2.80726200	0.11573800	-1.78101200
H	-1.50496300	2.37286100	-1.78201700
H	-3.76690300	3.12527100	-1.16315700
H	-6.26370100	1.00185400	0.55675600
H	-6.02533600	2.46821300	-0.38867200
H	-4.90711000	3.56864800	1.59027400

H	-6.54631000	3.02814000	1.98916000
H	-5.14479600	2.09489200	2.54066100
H	-4.37238100	-0.48132600	1.09199300
H	-2.10624100	-1.24860300	0.50326000
H	2.13367500	-1.19940600	0.50420600
H	2.60233500	-3.54583800	1.09250600

Compound **21** (radical cation)
Energy: -28294.440210355886 eV

Atom	X (Å)	Y (Å)	Z (Å)
C	0.96722600	4.10943700	0.66213000
C	1.53038600	3.61027600	-0.51535100
C	1.14545800	2.36347600	-0.99657500
C	0.19075500	1.60527100	-0.29058700
P	-0.22692400	-0.02805800	-0.90039700
C	-1.85957400	-0.51873000	-0.40118000
C	-2.89840600	0.44231000	-0.38934800
C	-4.19143000	0.05906000	-0.06145300
C	-4.47209800	-1.27910900	0.23578600
C	-3.45598600	-2.24144000	0.19903900
C	-2.15866400	-1.87311300	-0.12848400
C	1.06479500	-1.27934100	-0.46995500
C	2.42191200	-0.93788200	-1.11560000
C	3.46718100	-1.99963700	-0.74084100
C	3.59511200	-2.15170200	0.77896800
C	2.24061800	-2.46906400	1.42057900
C	1.18320100	-1.40962600	1.06825600
C	-0.38183600	2.11639700	0.89214400
C	0.01619200	3.36300700	1.36335900
H	1.26887700	5.08306800	1.03363500
H	2.26252400	4.19570400	-1.06061100
H	1.57132700	1.98486300	-1.91987200
H	-2.68543100	1.47695900	-0.63439700
H	-4.98461800	0.79836000	-0.04464600
H	-5.48526300	-1.57503900	0.48643900
H	-3.68049600	-3.27786300	0.42586800
H	-1.38070100	-2.62717200	-0.15772900
H	0.70212500	-2.22300600	-0.89649800
H	2.75585900	0.04164100	-0.75321300
H	2.32119400	-0.86916600	-2.20355100
H	3.17893000	-2.96103400	-1.18551400
H	4.42964300	-1.72577800	-1.18342700
H	4.31701300	-2.93794200	1.01907800
H	3.99011000	-1.22058100	1.20639300
H	2.33040600	-2.52519900	2.51037100
H	1.89155500	-3.45309300	1.08147900
H	1.47938500	-0.43866900	1.48221000
H	0.21688700	-1.66930700	1.51061000
H	-1.11211200	1.53518900	1.44298200
H	-0.41241200	3.75140000	2.28102400

Compound **22** (radical cation)
Energy: -45415.422443056916 eV

Atom	X (Å)	Y (Å)	Z (Å)
C	-0.68868700	-2.77448200	2.51733200
C	-2.13909700	-2.65432500	2.02137200
C	-3.04238900	-2.31375600	3.22624500
C	-2.56322900	-4.01208200	1.41903800
C	-2.29375100	-1.56904200	0.94833300
C	-1.21663400	-0.81558200	0.49340000
C	-1.41012800	0.16468500	-0.50258200
P	-0.04646100	1.18172600	-1.07068200
C	1.50905000	0.36957400	-0.79206400
C	1.59270500	-1.02979100	-0.98666500
C	2.78583300	-1.70029300	-0.75426300
C	2.91680200	-3.22172000	-0.88816700
C	1.60130100	-3.87624700	-1.34026500
C	3.30925500	-3.80787700	0.48627800
C	4.01134700	-3.55036700	-1.92669600
C	3.90447200	-0.93409300	-0.36580200
C	3.87103200	0.45837900	-0.20940400
C	5.09695300	1.27976500	0.21205800
C	5.41400900	2.31266500	-0.89152100
C	4.77908300	2.01331700	1.53396200
C	6.33830100	0.39968700	0.42891800
C	2.65488100	1.10521500	-0.44112800
C	-0.10131400	2.86565400	-0.30191300
C	-1.37480000	3.63222100	-0.70755500
C	-1.38536400	5.02514900	-0.06043400
C	-1.25141100	4.93783300	1.46404600
C	0.00349600	4.15605800	1.86667200
C	0.02545400	2.75485300	1.23591400
C	-2.68248000	0.38666300	-1.04667800
C	-3.78649100	-0.34794500	-0.59671400
C	-5.17239400	-0.07420800	-1.19577100
C	-6.26132600	-0.95704200	-0.56573000
C	-5.13069100	-0.35419200	-2.71399200
C	-5.54032900	1.40601400	-0.95106100
C	-3.56051000	-1.31466000	0.38850300
H	-0.00169600	-3.04325600	1.70776800
H	-0.62557300	-3.56037800	3.27426200
H	-0.33578200	-1.84586500	2.97748400
H	-2.76216100	-1.35195700	3.66658700
H	-2.94429200	-3.08324000	3.99768500
H	-4.09729200	-2.26099600	2.94383500
H	-2.46282300	-4.80214000	2.16923000
H	-1.93520700	-4.27367900	0.56157000
H	-3.60380100	-4.00316700	1.08377100
H	-0.23108700	-0.96209400	0.91029800
H	0.71171400	-1.57331200	-1.29892000

H	0.79044400	-3.70244900	-0.62469500
H	1.28103800	-3.51349700	-2.32211100
H	1.74042900	-4.95733300	-1.41950300
H	4.26316100	-3.41005100	0.84351200
H	2.54750400	-3.58505000	1.24024800
H	3.40934700	-4.89484800	0.41429700
H	3.75690200	-3.13938100	-2.90817200
H	4.98659000	-3.15094400	-1.63522700
H	4.11584700	-4.63456100	-2.02775600
H	4.83724500	-1.45164800	-0.18678800
H	6.28591200	2.90851700	-0.60633600
H	5.63592500	1.81696600	-1.84114700
H	4.58203300	3.00346000	-1.05772000
H	3.93437700	2.70094300	1.42764900
H	5.64425100	2.60160600	1.85320200
H	4.53882500	1.30197400	2.32996300
H	6.62845300	-0.12861200	-0.48455200
H	6.18343900	-0.33685700	1.22358800
H	7.18181900	1.02838900	0.72511400
H	2.59288200	2.17989800	-0.32300500
H	0.76932700	3.39605400	-0.70791600
H	-1.43921900	3.71711800	-1.79731500
H	-2.25387800	3.07034700	-0.37022800
H	-2.30896500	5.54316400	-0.33672700
H	-0.55753000	5.61896600	-0.46970700
H	-1.22474300	5.94185500	1.89837000
H	-2.13741100	4.43872100	1.87864900
H	0.06467300	4.05511000	2.95535300
H	0.89904000	4.70737400	1.55059500
H	-0.81600700	2.16319200	1.61534300
H	0.94197600	2.22426600	1.50953400
H	-2.80917700	1.12505600	-1.83035400
H	-7.22797800	-0.72026300	-1.01761800
H	-6.07245100	-2.02154300	-0.73573600
H	-6.35162300	-0.78720600	0.51176600
H	-4.40471800	0.28240600	-3.22819200
H	-6.11203800	-0.16148900	-3.15744400
H	-4.86601800	-1.39698800	-2.91283000
H	-4.82819000	2.08849600	-1.42480000
H	-6.52871600	1.62059400	-1.36787300
H	-5.56632900	1.63172100	0.11944700
H	-4.39922900	-1.89944800	0.74176900

Compound **23** (radical cation)
Energy: -31405.8936170435 eV

Atom	X (Å)	Y (Å)	Z (Å)
C	-4.70028800	-0.18892800	1.47413600
C	-3.71796400	0.55329700	0.60586700
C	-4.01797200	1.84071000	0.12782300
C	-3.11893000	2.55165500	-0.66827400

C	-1.89509800	1.98974400	-1.01254200
C	-1.56963100	0.70632000	-0.52899600
P	-0.00034600	0.00018600	-1.00165300
C	1.39601500	1.00606500	-0.52939700
C	2.66994400	0.64594200	-1.01347400
C	3.76885900	1.42441800	-0.66939900
C	3.60330600	2.55826400	0.12711800
C	2.33865200	2.94242800	0.60571200
C	2.18784100	4.16394600	1.47454700
C	1.23670500	2.15936000	0.26416500
C	0.17304800	-1.71199400	-0.52928200
C	-0.77493300	-2.63582500	-1.01374800
C	-0.64950100	-3.97664800	-0.66947400
C	0.41521100	-4.39948000	0.12744100
C	1.37940900	-3.49566000	0.60641200
C	2.51303100	-3.97505200	1.47520900
C	1.25131500	-2.14988800	0.26492200
C	-2.48862300	-0.00891000	0.26418100
H	-4.33549800	-1.18532800	1.73231800
H	-5.66301400	-0.30041200	0.96520300
H	-4.88753000	0.35491100	2.40569200
H	-4.97369900	2.28895400	0.38313300
H	-3.37961200	3.54053200	-1.02968100
H	-1.20173800	2.53266600	-1.64644400
H	2.79300700	-0.22588400	-1.64761300
H	4.75537100	1.15553300	-1.03125400
H	4.46965000	3.16146100	0.38231800
H	2.57256800	5.05352500	0.96570600
H	1.14274800	4.34639500	1.73346800
H	2.75299600	4.05369600	2.40570900
H	0.25391400	2.43066500	0.63281800
H	-1.59136600	-2.30706300	-1.64834800
H	-1.37497900	-4.69705400	-1.03161000
H	0.50507000	-5.45130000	0.38262700
H	2.13518100	-4.41312100	2.40467200
H	3.19099700	-3.16000800	1.73714800
H	3.09387900	-4.74989900	0.96476800
H	1.97684400	-1.43384600	0.63407500
H	-2.23197900	-0.99578600	0.63241200

Compound **24** (radical cation)
Energy: -28195.3542803394 eV

Atom	X (Å)	Y (Å)	Z (Å)
C	4.29615800	-1.07812400	0.40723400
C	3.24696800	-1.87373700	0.87719900
C	1.93197100	-1.56486100	0.54830100
C	1.66495500	-0.43626900	-0.25235500
P	0.00000800	-0.00004500	-0.72217200
C	-0.45449900	1.66003700	-0.25239800
C	0.38976900	2.45565500	0.54773600

C	-0.00013700	3.74891000	0.87679300
C	-1.21410600	4.25956300	0.40756200
C	-2.04871100	3.47685700	-0.39703600
C	-1.67164500	2.18425000	-0.73859400
C	-1.21040300	-1.22371200	-0.25239700
C	-2.32254100	-0.89009000	0.54633300
C	-3.24753000	-1.87446300	0.87528800
C	-3.08194300	-3.18141500	0.40718500
C	-1.98609100	-3.51303900	-0.39635700
C	-1.05516700	-2.54016100	-0.73773500
C	2.72750900	0.35523400	-0.73937800
C	4.03552600	0.03550900	-0.39803300
H	5.31963500	-1.32799100	0.66609200
H	3.45515800	-2.73159700	1.50739200
H	1.11834800	-2.17434800	0.92346500
H	1.32457400	2.05572000	0.92243700
H	0.63902000	4.35822900	1.50657000
H	-1.50938800	5.27084400	0.66653400
H	-2.98577500	3.88008400	-0.76501100
H	-2.31073600	1.58237100	-1.37684300
H	-2.44475000	0.11977200	0.91981300
H	-4.09550200	-1.62537700	1.50402300
H	-3.81008100	-3.94281900	0.66608000
H	-1.86624700	-4.52634700	-0.76368500
H	-0.21395300	-2.79287300	-1.37533800
H	2.52583200	1.20914700	-1.37829100
H	4.85323400	0.64500900	-0.76674600

Compound **25** (radical cation)
Energy: -44345.136947972795 eV

Atom	X (Å)	Y (Å)	Z (Å)
C	-6.10954900	0.42487000	-0.10196500
C	-5.15643000	-0.04549600	-1.22296500
C	-5.21722200	0.97262200	-2.37267300
C	-5.62037500	-1.41811600	-1.75795600
C	-3.74209100	-0.18218300	-0.64661200
C	-2.65858500	0.54540100	-1.13264600
C	-1.38411400	0.38956100	-0.55428100
P	-0.02786000	1.42894300	-1.10622500
C	1.51870400	0.60333200	-0.81596500
C	1.60273500	-0.78447500	-1.08270700
C	2.78452300	-1.47347100	-0.84548800
C	2.92102300	-2.98384100	-1.06978600
C	1.62276600	-3.60600400	-1.60846200
C	3.26679800	-3.65790200	0.27653900
C	4.05179800	-3.24584800	-2.08846700
C	3.88858200	-0.73603100	-0.36947100
C	3.85202000	0.64427500	-0.12762500
C	5.06067500	1.42976800	0.39891200
C	5.41952100	2.54045000	-0.61242600

C	6.29353500	0.53354400	0.59760700
C	4.69138700	2.06425900	1.75812400
C	2.64858000	1.31082200	-0.37095900
C	-0.08839100	3.07405600	-0.27519200
C	-1.40641100	3.85082700	-0.58528800
C	-1.90987800	4.33417400	0.78780800
C	-1.46331200	3.22684300	1.74971900
C	-0.03727400	2.90817000	1.28408900
C	-1.18268600	-0.51843000	0.49591500
C	-2.25320700	-1.26455400	1.00110000
C	-2.00423700	-2.23437800	2.16447600
C	-1.52653600	-1.42756000	3.39185400
C	-3.27139900	-3.01313300	2.55093200
C	-0.91198700	-3.24684900	1.75426500
C	-3.50899100	-1.07555200	0.41701000
H	-7.12533500	0.52901400	-0.49447500
H	-5.79728400	1.39523400	0.29646200
H	-6.14670900	-0.28503600	0.72883300
H	-4.58077800	0.67854900	-3.21342600
H	-4.92270400	1.97542200	-2.04566500
H	-6.24185900	1.03975500	-2.74692300
H	-5.64375500	-2.17711800	-0.97121200
H	-4.95682800	-1.77407500	-2.55181500
H	-6.63069900	-1.33641000	-2.16944600
H	-2.78585200	1.22115000	-1.96828300
H	0.72992200	-1.30471500	-1.45356400
H	0.78803400	-3.47634200	-0.91223100
H	1.33870400	-3.18158900	-2.57668400
H	1.76572000	-4.68010300	-1.75115200
H	3.37627900	-4.73728500	0.13646400
H	2.47611100	-3.48988000	1.01465900
H	4.20453700	-3.28112600	0.69441900
H	5.01636300	-2.86896900	-1.73743600
H	4.15942300	-4.32134800	-2.25676900
H	3.83205800	-2.77080600	-3.04919700
H	4.81285800	-1.26741900	-0.18697400
H	5.67707200	2.11575700	-1.58712900
H	4.59511200	3.24472100	-0.75820200
H	6.28026300	3.11064600	-0.25112600
H	6.11041700	-0.25704100	1.33204700
H	6.61675900	0.07056300	-0.33990300
H	7.12550800	1.13773700	0.96811700
H	4.42666900	1.29602700	2.49099400
H	5.54121300	2.63031000	2.15038600
H	3.84654100	2.75426900	1.67118900
H	2.58143800	2.37532500	-0.18403900
H	0.78057600	3.62363100	-0.64571500
H	-1.22987400	4.66102000	-1.29528600
H	-2.15808500	3.19221200	-1.02814900
H	-1.42569000	5.27947900	1.05696700
H	-2.98917500	4.50407700	0.79291000

H	-1.49641400	3.52505800	2.80073400
H	-2.10117700	2.34298500	1.63305800
H	0.31615200	1.92763700	1.61116900
H	0.66910000	3.64936300	1.67341800
H	-0.19644000	-0.62795000	0.92766600
H	-2.28140100	-0.69669100	3.69719300
H	-1.34174000	-2.09897400	4.23568600
H	-0.59682000	-0.88792000	3.18692400
H	-3.65116700	-3.61181600	1.71719700
H	-3.04229100	-3.69837800	3.37120100
H	-4.07006800	-2.34802500	2.89336400
H	0.03536200	-2.75282300	1.51788700
H	-1.22183100	-3.82389800	0.87750600
H	-0.72280200	-3.94783600	2.57258100
H	-4.34734300	-1.64543000	0.79464300

Compound **26** (radical cation)
Energy: -30566.40958272608 eV

Atom	X (Å)	Y (Å)	Z (Å)
C	-2.37692100	-2.36189800	1.54353300
C	-1.68440500	-0.99519800	1.64817600
C	-2.62860100	0.06191900	2.26085900
C	-0.40684400	-1.10655000	2.50170000
P	-1.14629900	-0.39595700	-0.04913000
C	-0.32473300	1.20326400	0.05951800
C	-1.09481900	2.38278700	0.09669600
C	-0.46483300	3.61589800	0.20559500
C	0.93059900	3.67423400	0.26341900
C	1.68988600	2.50863200	0.21457000
C	1.08468600	1.24983900	0.10883600
C	1.91797300	-0.00599200	-0.01765800
C	2.99071100	-0.15958400	1.07384600
C	3.74926800	-1.48368000	0.91761200
C	4.36888300	-1.61245600	-0.47928500
C	3.31131700	-1.44787400	-1.57770700
C	2.54139500	-0.13097300	-1.42471300
C	-2.32576000	-0.53901300	-1.50515900
C	-3.74331500	-0.06534800	-1.13060600
C	-1.74614200	0.32579700	-2.63948100
C	-2.34835900	-2.01809600	-1.94436300
H	-1.73573600	-3.11244300	1.07390100
H	-2.60583500	-2.70877000	2.55584000
H	-3.32042600	-2.31024300	0.99621700
H	-2.14091300	1.03397100	2.35948500
H	-3.55071300	0.18214700	1.68924200
H	-2.90211200	-0.27879200	3.26547100
H	-0.69703900	-1.39893300	3.51547500
H	0.27806700	-1.86625000	2.11663900
H	0.12350800	-0.15384000	2.56862200
H	-2.17758300	2.33642000	0.05896300

H	-1.05599700	4.52413200	0.24776100
H	1.42763200	4.63551700	0.34171000
H	2.77164900	2.57557700	0.24856500
H	1.22825500	-0.86733400	0.08647200
H	3.70262000	0.67201600	0.99850600
H	2.53135800	-0.08756700	2.06546400
H	3.05594000	-2.31973100	1.08805100
H	4.52223500	-1.55919100	1.68871600
H	4.87249700	-2.57871700	-0.58233500
H	5.14125500	-0.84177100	-0.60325100
H	3.77599300	-1.49096300	-2.56789700
H	2.60204300	-2.28631800	-1.52844000
H	3.21802900	0.71881100	-1.58247300
H	1.75651000	-0.04751700	-2.18614400
H	-3.76335300	0.97786500	-0.80682800
H	-4.19782500	-0.68471100	-0.35498400
H	-4.37144500	-0.14418200	-2.02447200
H	-1.74162500	1.38851300	-2.39265200
H	-2.37107200	0.18423200	-3.52656800
H	-0.72641500	0.02839800	-2.90007700
H	-1.35221200	-2.37209700	-2.22319800
H	-2.75104900	-2.67899500	-1.17555700
H	-2.99167100	-2.10412700	-2.82559300

Compound **27** (radical cation)
Energy: -34780.66506868477 eV

Atom	X (Å)	Y (Å)	Z (Å)
C	1.36698600	3.17623500	2.49453500
C	0.08407900	2.85008000	2.94708100
C	-0.64908800	1.87707700	2.28309200
C	-0.08663300	1.20869000	1.17537500
P	-1.00094600	-0.09123400	0.35103300
C	-0.42452900	-1.80623500	0.77661300
C	0.66959100	-1.79407200	1.86170900
C	1.06232200	-3.23298300	2.22877100
C	1.50181200	-4.03163800	0.99680600
C	0.40962400	-4.03482200	-0.07837300
C	0.00485700	-2.60771700	-0.47126600
C	-2.82980800	0.09382900	0.56266200
C	-3.29184000	1.48900600	0.09298100
C	-3.15125600	1.65599500	-1.42532400
C	-3.88163900	0.54518100	-2.18906300
C	-3.42626800	-0.84617500	-1.73352000
C	-3.56537900	-1.01832500	-0.21519100
C	1.20797700	1.53673700	0.70446300
C	1.76046900	0.90976000	-0.55531900
C	3.18905800	0.32817300	-0.41237000
C	3.92248900	0.24441300	-1.76713700
C	2.93706100	-0.02808900	-2.90772200
C	1.95116800	1.15017500	-3.07550700

C	1.68974400	1.88967100	-1.74654600
C	1.91641300	2.52813600	1.39042100
H	1.93764700	3.94696500	3.00212100
H	-0.33682100	3.35016600	3.81235500
H	-1.63929100	1.61630500	2.63967800
H	-1.32911300	-2.27415500	1.19057100
H	0.32408200	-1.25087100	2.74647300
H	1.54848400	-1.25931400	1.48751300
H	1.86170600	-3.20242700	2.97601600
H	0.20735000	-3.73137600	2.70423100
H	2.41797200	-3.58843400	0.58326400
H	1.75206300	-5.05753300	1.28289200
H	0.74598000	-4.57163800	-0.97076900
H	-0.47300200	-4.56990800	0.29626000
H	-0.80400200	-2.62795500	-1.20861000
H	0.85724000	-2.10483500	-0.94443800
H	-3.01261700	-0.02782100	1.64071600
H	-2.75203200	2.28008800	0.61987600
H	-4.34655200	1.58703500	0.37729000
H	-3.52989400	2.63893300	-1.72044000
H	-2.08377700	1.64479000	-1.69592800
H	-4.96166100	0.64254900	-2.01817800
H	-3.72480600	0.65955600	-3.26591000
H	-3.99892300	-1.62605600	-2.24415900
H	-2.37506900	-0.99611200	-2.02501200
H	-3.22993800	-2.01223600	0.09820000
H	-4.62322000	-0.95288400	0.06540000
H	1.08852500	0.07677100	-0.82555000
H	3.77358100	0.93149000	0.28890200
H	3.11825100	-0.66970300	0.03573500
H	4.69281500	-0.53069700	-1.72011400
H	4.44674800	1.18668600	-1.96523500
H	3.46960700	-0.20972300	-3.84528000
H	2.38582700	-0.95294600	-2.68888900
H	1.00314600	0.78230100	-3.48407900
H	2.34353800	1.86570500	-3.80483800
H	2.43568500	2.67683100	-1.58634100
H	0.71618300	2.39030100	-1.76776000
H	2.90521400	2.81007400	1.04551900

Compound **28** (radical cation)
Energy: -36295.90380462519 eV

Atom	X (Å)	Y (Å)	Z (Å)
F	-3.37354200	4.57912900	-0.63017200
C	-2.60317900	3.54594800	-0.30210900
C	-3.13233800	2.54655800	0.51723300
C	-2.32472100	1.47425100	0.86117000
C	-1.00272700	1.39110300	0.36780900
P	-0.00002700	0.00001500	0.85681300
C	-0.70327400	-1.56389900	0.36763200

C	-0.11344300	-2.75031000	0.86012000
C	-0.63832800	-3.98594500	0.51650400
C	-1.76924300	-4.02749800	-0.30167000
F	-2.27885500	-5.21130500	-0.62945600
C	-2.38481500	-2.87278300	-0.78728400
C	-1.85344900	-1.63795000	-0.44627800
C	1.70597500	0.17292500	0.36765200
C	2.43930900	1.27545700	0.86224600
C	3.77182700	1.43869000	0.51854200
C	4.37244300	0.48147100	-0.30188400
F	5.65245400	0.63195000	-0.62972100
C	3.67939700	-0.62750200	-0.78970000
C	2.34432400	-0.78468000	-0.44861800
C	-0.49204500	2.42345400	-0.44721900
C	-1.29572000	3.50097100	-0.78856300
H	-4.15082400	2.63271600	0.87693800
H	-2.71560700	0.69917800	1.51255800
H	0.75390400	-2.70121700	1.51061300
H	-0.20312200	-4.91101700	0.87562100
H	-3.25588100	-2.96051000	-1.42598900
H	-2.31297300	-0.73354800	-0.82698200
H	1.96377600	2.00087200	1.51453600
H	4.35597700	2.27699000	0.87931800
H	4.19026700	-1.33688900	-1.43015200
H	1.79014500	-1.63352700	-0.83122200
H	0.52065700	2.36861900	-0.82862900
H	-0.93638600	4.29858400	-1.42816700

Compound **29** (radical cation)
Energy: -53876.827280268735 eV

Atom	X (Å)	Y (Å)	Z (Å)
C	-5.19740200	0.82949800	-1.96401000
C	-4.90087100	2.04352700	-1.06891100
C	-5.74392800	1.91957000	0.21965600
C	-5.30269500	3.32410000	-1.83247200
C	-3.41966000	2.12661200	-0.68071000
C	-2.48749500	1.17338600	-1.07538200
C	-1.14650400	1.27458900	-0.64696700
P	0.00006100	0.00030900	-1.14777600
C	1.67702900	0.35580200	-0.64708200
C	2.40184800	-0.54929800	0.13910600
C	3.71215600	-0.25262900	0.52940100
C	4.47677000	-1.25489900	1.40477000
C	4.50269800	-2.62904200	0.69928400
C	5.92557400	-0.81197900	1.66283000
C	3.75029300	-1.38680000	2.76144300
C	4.25415400	0.96776500	0.11309300
C	3.55232800	1.89745800	-0.68112900
C	4.22171300	3.22127200	-1.06971600
C	3.31912800	4.08529600	-1.96513100

C	5.53154700	2.92818300	-1.83307300
C	4.53615300	4.01360000	0.21865100
C	2.26060700	1.56724500	-1.07602300
C	-0.53028300	-1.62975200	-0.64697400
C	-1.67684400	-1.80537600	0.13873000
C	-2.07491400	-3.08867400	0.52851500
C	-3.32550900	-3.25034300	1.40332300
C	-3.66614800	-4.72664300	1.66060700
C	-3.07720000	-2.55568600	2.76034800
C	-4.52836100	-2.58559500	0.69767800
C	-1.28849600	-4.16795300	0.11232200
C	-0.13199800	-4.02450600	-0.68121600
C	0.68028000	-5.26583400	-1.06962000
C	1.88113800	-4.91547400	-1.96302200
C	-0.22756500	-6.25286300	-1.83506400
C	1.20738300	-5.93549000	0.21888000
C	0.22768400	-2.74057400	-1.07569900
C	-0.72531400	2.35546100	0.13858900
C	-1.63775000	3.34174800	0.52857300
C	-1.15245600	4.50590800	1.40305100
C	-2.26053900	5.53947700	1.65932700
C	0.02510100	5.21467400	0.69761200
C	-0.67581100	3.94399200	2.76053600
C	-2.96574800	3.19994900	0.11278400
H	-6.26100100	0.81303600	-2.21528400
H	-4.63860900	0.87015900	-2.90442100
H	-4.96612600	-0.11586300	-1.46232000
H	-5.47804100	1.01527300	0.77654100
H	-5.60375400	2.77726100	0.88335300
H	-6.80719900	1.86347800	-0.03192600
H	-4.71212500	3.43653200	-2.74661700
H	-5.16322400	4.22386800	-1.22722300
H	-6.35898100	3.27541200	-2.11301300
H	-2.78351800	0.34291200	-1.70361600
H	1.93380000	-1.46910900	0.46414900
H	5.05538900	-3.35164900	1.30687100
H	3.49566100	-3.02941100	0.54776800
H	4.99143800	-2.56096600	-0.27739900
H	6.43429900	-1.56487200	2.27049000
H	5.97157000	0.13465600	2.20998500
H	6.48961800	-0.70276600	0.73125400
H	4.27603700	-2.10151700	3.40177400
H	2.72267700	-1.74192100	2.63840800
H	3.71509900	-0.42499400	3.28187800
H	5.26372600	1.21557400	0.41245700
H	3.83713300	5.01434800	-2.21651100
H	2.38481700	4.35823100	-1.46367600
H	3.07489700	3.58093300	-2.90547000
H	6.01804400	3.86701300	-2.11366400
H	6.24065700	2.35722800	-1.22763600
H	5.33344300	2.36050400	-2.74716900

H	5.20868600	3.46329600	0.88250300
H	3.62015200	4.23591400	0.77551500
H	5.01949600	4.96224500	-0.03318600
H	1.68997000	2.23881800	-1.70483700
H	-2.23963700	-0.94028300	0.46387800
H	-2.86958300	-5.23983100	2.20807000
H	-3.85285100	-5.26939800	0.72868700
H	-4.57292100	-4.79122900	2.26764200
H	-2.87120300	-1.48807100	2.63772000
H	-2.22673500	-3.00605800	3.28095400
H	-3.95925500	-2.65407800	3.40031300
H	-4.71327800	-3.04236800	-0.27934800
H	-4.37165100	-1.51317500	0.54690600
H	-5.43074700	-2.70341900	1.30481000
H	-1.57847700	-5.16639500	0.41132500
H	1.56784600	-4.45131800	-2.90358800
H	2.42728100	-5.82836100	-2.21404800
H	2.58371400	-4.24290300	-1.46010300
H	-0.61887900	-5.79662900	-2.74928500
H	0.34250400	-7.14343500	-2.11564500
H	-1.07743600	-6.58184500	-1.23103000
H	1.78760800	-6.82817500	-0.03295800
H	1.85712700	-5.25388200	0.77727500
H	0.39364900	-6.24342400	0.88129300
H	1.09495400	-2.58169200	-1.70388900
H	0.30530800	2.41059900	0.46347200
H	-3.10356500	5.10678000	2.20675400
H	-2.63681900	5.97198700	0.72698200
H	-1.86313700	6.35736700	2.26598600
H	0.87547400	4.54260700	0.54739000
H	0.37414600	6.05526900	1.30454300
H	-0.27755600	5.60287600	-0.27969100
H	-0.31984200	4.75721300	3.40023800
H	-1.49151100	3.43329400	3.28109900
H	0.14544900	3.23131200	2.63855300
H	-3.68545700	3.95018500	0.41190400

Compound **30** (radical cation)
Energy: -45196.05144918603 eV

Atom	X (Å)	Y (Å)	Z (Å)
C	2.77336100	-3.01476000	1.17164800
C	1.75032500	-3.26266100	0.07799800
C	2.18083800	-4.31630300	-0.93075400
O	1.54425200	-1.98180000	-0.60788100
C	0.29360600	-1.81330100	-1.14780500
C	0.08099800	-2.07016600	-2.50116400
C	-1.20958500	-1.96822400	-3.01273300
C	-2.29215900	-1.63124300	-2.19692000
C	-2.08109100	-1.35872600	-0.84220900
O	-3.04702700	-1.01320500	0.04080600

C	-4.44916200	-1.05209500	-0.33570500
C	-5.20948000	-1.26901200	0.96270100
C	-4.83999300	0.24062500	-1.04153400
C	-0.76838900	-1.40000300	-0.30706900
C	-0.50853600	-1.08061300	1.11708300
C	-1.10082400	-1.81433100	2.15160600
C	-0.75350200	-1.57758500	3.47876700
C	0.19028800	-0.59706400	3.80106600
C	0.77370800	0.16176600	2.79260500
C	0.40587000	-0.05485400	1.45343300
P	1.04689300	0.96896900	0.11632000
C	-0.12389400	2.34513800	-0.29442900
C	-0.31729400	3.25331700	0.94529200
C	-1.21614400	4.44877700	0.58747100
C	-2.55593700	3.99590700	-0.00052400
C	-2.34400600	3.08460300	-1.21398400
C	-1.47152600	1.87255700	-0.85733000
C	2.67816100	1.70347800	0.61612300
C	3.12870900	2.79609800	-0.37145500
C	3.51453100	2.20944700	-1.73429000
C	4.60539100	1.14220300	-1.58354500
C	4.18525300	0.04160200	-0.60172400
C	3.75895500	0.61328300	0.75698300
H	3.71312700	-2.65526700	0.74296200
H	2.40332600	-2.27812200	1.88864400
H	2.97862600	-3.94487300	1.70846900
H	0.79289700	-3.54653100	0.53104300
H	1.42174200	-4.45499700	-1.70464700
H	3.11975700	-4.02271600	-1.40930000
H	2.33272400	-5.27737700	-0.43070300
H	0.91952400	-2.36317700	-3.12068000
H	-1.38709200	-2.16969200	-4.06405400
H	-3.28321800	-1.57466500	-2.62557600
H	-4.60073700	-1.91348500	-0.99629600
H	-5.01630700	-0.44668600	1.65766200
H	-4.90701300	-2.20540100	1.43771800
H	-6.28404800	-1.31123900	0.76667900
H	-4.71306500	1.09000600	-0.36406600
H	-4.23481800	0.41643300	-1.93403700
H	-5.88974600	0.19916000	-1.34517300
H	-1.81710100	-2.58825300	1.90267000
H	-1.20892300	-2.16808300	4.26685200
H	0.45994700	-0.41737700	4.83621500
H	1.48992300	0.93284600	3.05157100
H	0.39965300	2.90999300	-1.07544200
H	-0.78171600	2.66387700	1.74447500
H	0.64498000	3.61307600	1.32289200
H	-1.37057000	5.05533100	1.48615200
H	-0.69307800	5.08600200	-0.13735600
H	-3.15703000	4.86715200	-0.27875200
H	-3.12372800	3.45230900	0.76692400

H	-3.30440100	2.73783700	-1.60631400
H	-1.86318600	3.65290100	-2.02122500
H	-1.31458600	1.24115800	-1.73591900
H	-1.98588200	1.26141600	-0.10889000
H	2.48663100	2.15995100	1.59699900
H	4.00679400	3.28036500	0.07347900
H	2.37210200	3.57960400	-0.47315200
H	3.84748800	3.01112000	-2.40054700
H	2.62738400	1.76109400	-2.20833100
H	4.84363500	0.70528400	-2.55838400
H	5.52423000	1.62363100	-1.22334700
H	3.35468200	-0.53767900	-1.02275500
H	5.00830500	-0.66502900	-0.45160100
H	4.62007200	1.08321600	1.24832800
H	3.41666600	-0.18477900	1.42100100

Compound **31** (radical cation)
Energy: -93591.08847416141 eV

Atom	X (Å)	Y (Å)	Z (Å)
C	0.97954800	3.41359200	-1.24554200
C	0.10890900	2.37753100	-0.53053500
N	0.67679500	1.78862300	0.68224300
C	0.73567800	2.63919300	1.86535400
P	1.52705500	0.31481100	0.52401200
C	0.94291400	-0.63728300	2.05159000
C	0.59179700	-2.10898600	1.78679800
C	-0.16533400	-2.71464300	2.97552000
C	0.63015600	-2.58195400	4.27687400
C	1.01441000	-1.12167500	4.53046100
C	1.78147600	-0.52066400	3.34152700
C	3.29091600	0.81167500	0.98302300
C	3.84007100	2.04740900	0.25095000
C	4.59028700	1.72691500	-1.04026300
C	5.75147200	0.76546300	-0.75587200
C	5.28442100	-0.52418000	-0.06609900
C	4.38869900	-0.26568800	1.15575000
C	-0.29024700	1.23393200	-1.41147200
C	0.07010300	1.03287500	-2.76747900
C	-0.63626700	-0.10470700	-3.24296200
C	-1.35475600	-0.65520500	-2.16865100
C	-1.17167200	0.15498300	-1.01039700
P	-2.10830300	0.02280900	0.57339500
C	-2.99211600	1.63415900	0.52055400
C	-3.20826600	2.28604900	1.74274300
C	-3.83338100	3.53344900	1.78139300
C	-4.24073600	4.14549400	0.59618000
C	-4.02640800	3.50731400	-0.62821400
C	-3.40628300	2.25994300	-0.66687400
C	-3.36773500	-1.22224900	0.06007200
C	-4.59970900	-0.90691400	-0.52774500

C	-5.47932700	-1.92114800	-0.90635600
C	-5.13721100	-3.26000800	-0.71166300
C	-3.91636600	-3.58471600	-0.11857500
C	-3.04349800	-2.57151100	0.27629000
Fe	0.79210900	-0.56858900	-1.50509700
C	2.70793400	-1.22638100	-2.20891700
C	1.86993700	-1.64891200	-3.27962800
C	0.95132500	-2.58736900	-2.79058000
C	1.17880400	-2.74497600	-1.39376300
C	2.32300100	-1.97619200	-1.05362000
H	1.87260000	2.96309500	-1.68620100
H	0.40683400	3.89654800	-2.04259500
H	1.29537600	4.19383700	-0.54901900
H	-0.81710100	2.88061600	-0.22425100
H	-0.19460000	3.21185900	1.92991800
H	0.80345100	2.03234200	2.76881900
H	1.57801800	3.34345700	1.85673100
H	-0.01391700	-0.13198500	2.22236700
H	1.50359200	-2.69192800	1.60344300
H	-0.02338900	-2.17673800	0.88899200
H	-0.39343900	-3.76638100	2.76935200
H	-1.12742500	-2.19553000	3.07789100
H	0.05184000	-2.97457300	5.11919200
H	1.54220900	-3.19059200	4.21028300
H	0.10595100	-0.52999200	4.70589300
H	1.62562200	-1.03586900	5.43492700
H	2.02997800	0.51890500	3.57260800
H	2.73220800	-1.05196500	3.22716500
H	3.05914300	1.15703200	1.99577300
H	3.04599900	2.77247600	0.08002800
H	4.54566300	2.52875000	0.94151700
H	3.89659100	1.29987900	-1.77455600
H	4.97130800	2.65353800	-1.48205100
H	6.47221600	1.28108700	-0.10801700
H	6.28710800	0.51923500	-1.67864700
H	4.77244500	-1.16135900	-0.78848400
H	6.15451900	-1.10071900	0.26485300
H	3.96953800	-1.20972000	1.51847200
H	5.02993700	0.09518900	1.96983800
H	0.72283900	1.66778300	-3.34795900
H	-0.56200200	-0.52442700	-4.23516200
H	-1.95457600	-1.55312600	-2.19489400
H	-2.88155300	1.81434700	2.66511400
H	-3.99849100	4.02666200	2.73393500
H	-4.72235800	5.11754500	0.62411900
H	-4.34369300	3.98210000	-1.55136600
H	-3.23623600	1.77312000	-1.62221200
H	-4.87886200	0.12902100	-0.68184300
H	-6.43403700	-1.66436600	-1.35448700
H	-5.82376900	-4.04600600	-1.00911800
H	-3.65228800	-4.62366800	0.05241100

H	-2.10936300	-2.82965000	0.76575100
H	3.56132700	-0.57922600	-2.30597100
H	1.91278000	-1.26585500	-4.28963200
H	0.14500500	-3.04074500	-3.34949300
H	0.64990400	-3.42119900	-0.74110300
H	2.83413800	-2.00185400	-0.10587100

Compound **32** (radical cation)
Energy: -65713.9400928534 eV

Atom	X (Å)	Y (Å)	Z (Å)
Cl	-5.91250000	-1.46315900	-0.62874600
C	-4.28248700	-1.06923600	-0.19825300
C	-3.23343000	-1.86686900	-0.67217500
C	-1.92659100	-1.55317000	-0.32837700
C	-1.65973000	-0.43111200	0.48198200
P	-0.00010000	-0.00014100	0.97513400
C	0.45641400	1.65274400	0.48227800
C	1.66872300	2.18929500	0.96868400
C	2.05290100	3.47495600	0.62112600
C	1.21514200	4.24310000	-0.19822900
Cl	1.68906500	5.85161100	-0.62897400
C	0.00015700	3.73313200	-0.67264300
C	-0.38159400	2.44456600	-0.32870400
C	1.20307000	-1.22183400	0.48205900
C	2.30814200	-0.89168700	-0.32831600
C	3.23342200	-1.86639600	-0.67215200
C	3.06743100	-3.17376900	-0.19827300
Cl	4.22373300	-4.38823800	-0.62891900
C	1.98294600	-3.51555200	0.62046200
C	1.06141600	-2.54018200	0.96793900
C	-2.73046700	0.35094500	0.96787300
C	-4.03599800	0.04081800	0.62043900
H	-3.44736600	-2.71566200	-1.31086200
H	-1.11398500	-2.16209700	-0.70670300
H	2.30803000	1.60006500	1.61842100
H	2.98407900	3.89247900	0.98504100
H	-0.62770200	4.34256700	-1.31180200
H	-1.31500600	2.04508200	-0.70736800
H	2.42896400	0.11654800	-0.70659500
H	4.07541600	-1.62708100	-1.31083600
H	1.87883500	-4.53085600	0.98402200
H	0.23119500	-2.79948600	1.61721500
H	-2.53973700	1.19961500	1.61706100
H	-4.86309000	0.63883500	0.98395900

Compound **33** (radical cation)
Energy: -34483.872133634315 eV

Atom	X (Å)	Y (Å)	Z (Å)

C	-3.79837700	-0.72720300	-2.08364400
C	-2.65878800	-0.18728300	-2.67502200
C	-1.84911600	0.69193400	-1.95312800
C	-2.17158100	1.02987600	-0.62334400
C	-1.26035400	1.88143600	0.17248900
C	-1.71630000	3.03102000	0.82643500
C	-0.83480300	3.83745500	1.54438100
C	0.52103100	3.50518700	1.63519000
C	0.99662600	2.35327800	1.02002700
C	0.10756500	1.52876200	0.30753700
P	0.62732400	-0.04586000	-0.37886700
C	-0.10855600	-1.42886700	0.49149400
C	0.00731200	-2.71499800	-0.06806300
C	-0.57003600	-3.80259400	0.57912100
C	-1.27806900	-3.61223700	1.76860400
C	-1.40857300	-2.33304900	2.31563100
C	-0.83292200	-1.23741400	1.68060500
C	2.40961100	-0.16555100	-0.45654200
C	3.10365000	-1.25714600	0.10117500
C	4.48847900	-1.31847900	-0.00980000
C	5.18694400	-0.31065300	-0.68080100
C	4.50060900	0.76856500	-1.24740300
C	3.11692200	0.84058000	-1.14909900
C	-3.33708500	0.50357400	-0.04992100
C	-4.14070200	-0.37309000	-0.77381800
H	-4.42978300	-1.40974800	-2.64251100
H	-2.40778900	-0.43033900	-3.70202000
H	-1.00548800	1.17149400	-2.43877200
H	-2.76078800	3.30880200	0.73417100
H	-1.20318800	4.73702100	2.02629100
H	1.20052500	4.13513200	2.19883500
H	2.03963200	2.07484000	1.11517800
H	0.54213800	-2.85986000	-1.00155700
H	-0.47715800	-4.79529900	0.15220900
H	-1.73167900	-4.46170600	2.26829400
H	-1.95529000	-2.19073500	3.24172100
H	-0.92878500	-0.24559500	2.10684800
H	2.56449500	-2.03231700	0.63278200
H	5.02602700	-2.14951200	0.43402700
H	6.26708000	-0.36688900	-0.76514900
H	5.04536200	1.54611400	-1.77175800
H	2.58359600	1.67250100	-1.59839900
H	-3.58877100	0.75570000	0.97509900
H	-5.03091100	-0.79031600	-0.31489700

Compound **34** (radical cation)
Energy: -29800.588753335804 eV

Atom	X (Å)	Y (Å)	Z (Å)
C	-4.26499900	-0.72234600	-0.39529400
C	-3.57740900	0.07852200	0.73139700

C	-2.48085500	-0.74904400	1.41203300
C	-1.39473100	-1.23735300	0.41290100
P	0.15764100	-0.17421700	0.46783400
C	1.34748200	-1.07080000	-0.81230000
C	1.42531700	-2.57461400	-0.49992800
C	2.21831000	-2.83969400	0.78419900
C	3.59492600	-2.16843700	0.71145500
C	3.45212500	-0.68508900	0.36592700
N	2.66013400	-0.51863500	-0.84919700
C	-0.50822000	1.48137600	-0.39206900
C	-0.87723900	2.51865200	0.66902700
C	0.35424100	3.26622200	1.18651000
C	1.13330200	3.87903900	0.01894300
C	1.52606500	2.79218300	-0.98373800
N	0.37908100	1.98158400	-1.35825700
N	-1.92464400	-1.26692200	-0.95671700
C	-3.31433000	-1.76806900	-0.99927900
H	-4.60764100	-0.03444200	-1.17330300
H	-5.15226600	-1.23565100	-0.01240600
H	-3.15449600	0.99962100	0.32239700
H	-4.31025600	0.39079800	1.48048500
H	-2.93876100	-1.63348000	1.86717500
H	-2.02216300	-0.19613500	2.23863600
H	-1.05457200	-2.22674000	0.75759800
H	0.85677000	-0.91053100	-1.77979300
H	0.43027800	-3.02332700	-0.45190100
H	1.93925600	-3.03546000	-1.35092300
H	1.66400000	-2.44810900	1.64927500
H	2.32130500	-3.91674000	0.94363800
H	4.12626200	-2.27095300	1.66198400
H	4.20275100	-2.65380100	-0.05985100
H	2.96121000	-0.16693700	1.21428900
H	4.42693100	-0.21704900	0.21013600
H	2.72854000	0.40147300	-1.26053300
H	-1.37427300	1.03599300	-0.89852100
H	-1.57102500	3.22797200	0.20118000
H	-1.41481000	2.03504700	1.48802600
H	0.04981700	4.04219300	1.89372500
H	0.99925000	2.56926700	1.73880800
H	2.03903300	4.38151400	0.36951600
H	0.51817700	4.62863300	-0.49047400
H	2.29510300	2.14894100	-0.51365500
H	1.96065800	3.21323500	-1.89348800
H	0.42449800	1.49045100	-2.24096600
H	-1.34098000	-1.86933800	-1.53093700
H	-3.40695600	-2.72560800	-0.46255700
H	-3.56616000	-1.96307700	-2.04457900

Compound **35** (radical cation)
Energy: -40915.42644918239 eV

Atom	X (Å)	Y (Å)	Z (Å)
C	-2.45557500	-3.49063800	0.17350000
O	-1.39029500	-2.56587500	0.41229100
C	-0.37947000	-2.50322700	-0.48811000
C	-0.42957700	-3.08430400	-1.75841500
C	0.69601800	-3.01772500	-2.57476700
C	1.88122100	-2.41021800	-2.15530800
C	1.93631600	-1.84162100	-0.88196700
O	3.04093900	-1.27114800	-0.34572000
C	4.23187600	-1.21931100	-1.13188800
C	0.78834000	-1.81989200	-0.05032500
C	0.84084300	-1.22842400	1.31163600
C	1.57783400	-1.81241100	2.34584000
C	1.52669900	-1.27598600	3.63045500
C	0.74225200	-0.15024900	3.90053600
C	0.01723700	0.45575000	2.87906900
C	0.08818100	-0.06846000	1.57916500
P	-0.69162000	0.70709400	0.14602400
C	-2.32578300	1.41554400	0.67439700
C	-3.33905900	0.26945300	0.87560800
C	-3.78089200	-0.33543800	-0.46321200
C	-4.31703500	0.72739700	-1.42861900
C	-3.29312900	1.84763000	-1.64538100
C	-2.86443800	2.46816400	-0.31047400
C	0.38599900	2.05417100	-0.52872000
C	0.65422500	3.12230900	0.55842600
C	1.50174300	4.26480200	-0.02580400
C	2.81077900	3.74332900	-0.62679400
C	2.54200900	2.66960400	-1.68712300
C	1.69758000	1.51882500	-1.12197800
H	-2.06346000	-4.50267800	0.03475800
H	-3.05125000	-3.20092000	-0.69688200
H	-3.07887400	-3.45485200	1.06604900
H	-1.31848400	-3.59512500	-2.10246600
H	0.65728100	-3.46535200	-3.56246500
H	2.74257200	-2.40190200	-2.80906400
H	4.57286600	-2.22485400	-1.39824000
H	4.07600400	-0.62932500	-2.04179400
H	4.97794600	-0.73457100	-0.50377300
H	2.16975300	-2.69764000	2.14261200
H	2.09110400	-1.74355100	4.43045900
H	0.70612300	0.26024600	4.90399000
H	-0.57173600	1.34129700	3.09045000
H	-2.11958100	1.89189600	1.64248100
H	-2.91783700	-0.50187000	1.52784300
H	-4.20860500	0.68874800	1.39599400
H	-2.92086400	-0.83829800	-0.92667200
H	-4.53719900	-1.10617300	-0.28400500
H	-5.23976200	1.15870700	-1.01894000
H	-4.58452100	0.26828100	-2.38557900
H	-2.41268500	1.43925700	-2.16483000

H	-3.70252100	2.62412300	-2.29860600
H	-2.14212100	3.27540200	-0.46328500
H	-3.73817000	2.93012100	0.16617700
H	-0.21075400	2.50157000	-1.33342200
H	-0.28439400	3.51767700	0.95992800
H	1.19253100	2.65299600	1.38975100
H	1.70214500	4.99633200	0.76413400
H	0.92144500	4.78544900	-0.79867300
H	3.38088200	4.57062000	-1.06075300
H	3.43133700	3.31726600	0.17292100
H	2.01740200	3.11992600	-2.54023000
H	3.48498200	2.27075300	-2.07599900
H	2.25993000	0.99883300	-0.34035200
H	1.48252000	0.78294600	-1.90292900

Compound **36** (radical cation)
Energy: -34747.23893814424 eV

Atom	X (Å)	Y (Å)	Z (Å)
C	3.34872400	1.84234300	0.79863200
C	2.57499500	1.19290700	1.96006300
C	3.41942400	1.30358200	3.24183400
C	1.25809200	1.96249000	2.16637900
C	2.34369500	-0.31725800	1.68634800
C	1.43002500	-0.67832400	0.49104900
C	-0.02796600	-0.78613800	0.99505700
P	-1.27984000	-0.51175200	-0.32229000
C	-1.18052200	1.16697900	-0.95101600
C	-2.02782800	2.17395000	-0.44501500
C	-1.85464700	3.48870600	-0.86485300
C	-0.85910100	3.80577400	-1.79299200
C	-0.03096200	2.80642400	-2.31011400
C	-0.18946100	1.48745700	-1.89732900
C	-2.93179200	-0.90282700	0.19694600
C	-3.17089800	-1.72268100	1.32271000
C	-4.47348100	-2.05018900	1.67269900
C	-5.54615500	-1.58973100	0.89994100
C	-5.31744100	-0.79749500	-0.23102500
C	-4.02090200	-0.46074200	-0.59229000
C	1.79618800	-2.00769000	-0.20623100
C	2.91009600	-2.02513200	-1.28300700
C	2.97475500	-3.46241800	-1.83370300
C	2.56145000	-1.06867100	-2.43625700
C	4.29233700	-1.65343200	-0.72091200
H	3.53322500	2.90027100	1.00953500
H	2.79702200	1.79348500	-0.14659600
H	4.32029200	1.36148900	0.65046200
H	4.36893100	0.76890000	3.13586000
H	2.89002500	0.88125600	4.10259400
H	3.64820500	2.34934000	3.46909300
H	0.64472300	1.98456000	1.25903400

H	0.66474700	1.53575500	2.98392400
H	1.46860100	3.00299700	2.43228100
H	3.32130400	-0.77899100	1.53476700
H	1.93733200	-0.78446100	2.59406500
H	1.48703400	0.13557900	-0.23662600
H	-0.19993000	-1.77738200	1.42559400
H	-0.25942400	-0.03773100	1.75904000
H	-2.79359600	1.93285900	0.28322500
H	-2.49431700	4.26778700	-0.46422600
H	-0.73178200	4.83341400	-2.11659400
H	0.73266100	3.05340900	-3.03954000
H	0.44364600	0.71171400	-2.31107100
H	-2.34761100	-2.08384700	1.92793100
H	-4.65796900	-2.66379500	2.54760800
H	-6.56121000	-1.85564200	1.17549500
H	-6.15180900	-0.45164000	-0.83118400
H	-3.84246300	0.14445400	-1.47450400
H	0.89052900	-2.39042900	-0.70232300
H	2.03914800	-2.74517900	0.57065000
H	2.01805300	-3.76513900	-2.27341400
H	3.74086500	-3.54488700	-2.61038100
H	3.22322300	-4.17715200	-1.04232200
H	1.56804200	-1.28875100	-2.84730100
H	3.28213900	-1.17249200	-3.25268300
H	2.58790600	-0.02116100	-2.11815100
H	4.34026700	-0.60857000	-0.40682100
H	4.55879500	-2.28375100	0.13378400
H	5.05925900	-1.79730100	-1.48834100

Compound **37** (radical cation)
Energy: -44312.58992838365 eV

Atom	X (Å)	Y (Å)	Z (Å)
C	4.52879800	2.40463700	-2.37496000
C	3.56072300	2.55160800	-1.18851100
C	2.52616300	3.65979500	-1.44835200
C	2.88128100	1.23517800	-0.86175100
C	2.03844500	0.61277900	-1.78315100
C	1.38070100	-0.58764000	-1.48933400
C	0.72186600	-1.38663000	-2.61053800
C	0.04383100	-0.52305000	-3.67977300
C	1.78467000	-2.30868300	-3.24235300
C	1.54406800	-1.15412100	-0.18590200
C	0.60468200	-2.22556400	0.23928800
C	0.99116500	-3.53077600	0.55159700
C	0.04144000	-4.48201500	0.92332000
C	-1.31299700	-4.14294300	0.98973400
C	-1.72314700	-2.85075900	0.67924500
C	-0.77017600	-1.88024900	0.32411000
P	-1.21834800	-0.20514900	-0.17427400
C	-0.62428500	1.11018100	0.98478100

C	-1.09979500	2.50203800	0.52411000
C	-0.54013900	3.58882500	1.45226500
C	-0.92654500	3.32379100	2.91105100
C	-0.43232800	1.94563600	3.36174600
C	-0.96942900	0.82007000	2.46217200
C	-3.03095900	-0.16286100	-0.55130300
C	-3.37621900	0.83653300	-1.67846300
C	-4.85941600	0.71989000	-2.05739700
C	-5.77269000	0.91014800	-0.84113200
C	-5.41469500	-0.07109700	0.28016800
C	-3.93700900	0.05109700	0.68341200
C	2.46382000	-0.59405200	0.72495500
C	2.78242400	-1.25033200	2.06214600
C	4.09622000	-2.04671200	1.94148300
C	2.85722200	-0.26520400	3.23652500
C	3.11040900	0.59363800	0.36206200
H	5.27499500	1.62843800	-2.18399700
H	3.99314200	2.13917400	-3.29225000
H	5.05341400	3.34689200	-2.55705400
H	4.14932700	2.84079500	-0.30948300
H	1.85478400	3.78624300	-0.59343300
H	1.91450900	3.43162500	-2.32760200
H	3.02640300	4.61499100	-1.63135600
H	1.91201300	1.05406200	-2.76468900
H	-0.03966100	-2.03760100	-2.17256100
H	-0.64641500	0.20031900	-3.23394800
H	0.76998600	0.03361600	-4.27950700
H	-0.52062300	-1.15713200	-4.36868000
H	1.33746400	-2.92002600	-4.03170500
H	2.21994700	-2.97883100	-2.49576400
H	2.59558000	-1.72049500	-3.68284400
H	2.03733300	-3.80569600	0.47903100
H	0.35641100	-5.49532800	1.14929700
H	-2.04702000	-4.88550200	1.28320500
H	-2.77570900	-2.59935800	0.73344500
H	0.46488600	1.07641000	0.87548500
H	-0.78547900	2.68303600	-0.50912600
H	-2.19393600	2.55234900	0.54554200
H	-0.90340300	4.56790600	1.12467600
H	0.55415400	3.61057100	1.36341500
H	-0.51243800	4.10150600	3.56003000
H	-2.01881000	3.37639800	3.01481800
H	-0.72554600	1.74485900	4.39735200
H	0.66419000	1.93763400	3.33673100
H	-0.54683700	-0.14511300	2.75891800
H	-2.05412600	0.74790800	2.58765800
H	-3.21149000	-1.17157800	-0.94653500
H	-2.74803300	0.64828700	-2.55426300
H	-3.16359600	1.85798400	-1.34547900
H	-5.03918600	-0.26944100	-2.49890900
H	-5.09341900	1.45586600	-2.83296300

H	-6.81995600	0.78354300	-1.13189700
H	-5.67255000	1.93820900	-0.46779200
H	-5.61440500	-1.09885300	-0.05120600
H	-6.04126500	0.10243600	1.16117200
H	-3.75847400	1.05071800	1.09398200
H	-3.69571100	-0.66482200	1.47527600
H	1.98235500	-1.95993500	2.28973200
H	4.30988400	-2.57796800	2.87361000
H	4.93640300	-1.37643800	1.73332800
H	4.05270300	-2.77937400	1.13030600
H	1.93212500	0.30632800	3.33898100
H	3.01904900	-0.81251000	4.16933300
H	3.68445200	0.44284300	3.13099400
H	3.79921000	1.05336200	1.06335400

Compound **38** (radical cation)
Energy: -29503.96426879961 eV

Atom	X (Å)	Y (Å)	Z (Å)
C	1.01227700	4.31513600	0.30542500
C	2.03112500	3.38668700	0.49888900
C	1.78226000	2.03822900	0.24550100
C	0.49184900	1.68797600	-0.17918800
P	-0.00009000	-0.00006600	-0.60697500
C	-1.70794800	-0.41804500	-0.17932200
C	-2.65667500	0.52442800	0.24476900
C	-3.94887800	0.06568800	0.49823500
C	-4.24327900	-1.28103600	0.30543900
C	-3.22558600	-2.13600700	-0.13922600
N	-1.98541300	-1.71886400	-0.38682900
C	1.21585400	-1.27006900	-0.17930900
C	0.87429300	-2.56289300	0.24515500
C	1.91789700	-3.45237500	0.49855200
C	3.23129400	-3.03387000	0.30522400
C	3.46257300	-1.72515600	-0.13992500
N	2.48101600	-0.85991300	-0.38742300
N	-0.49571300	2.57880300	-0.38736200
C	-0.23683700	3.86139700	-0.13982400
H	1.17320000	5.37251500	0.48310900
H	3.01196400	3.70262100	0.83766800
H	2.54675100	1.28207300	0.35599900
H	-2.38417900	1.56464300	0.35480200
H	-4.71304500	0.75719900	0.83659800
H	-5.23942300	-1.67042300	0.48318500
H	-3.42078100	-3.19082800	-0.31043100
H	-0.16273900	-2.84718000	0.35553400
H	1.70135700	-4.45985300	0.83726700
H	4.06671400	-3.70171500	0.48291700
H	4.47356100	-1.36669900	-0.31158500
H	-1.05256900	4.55793400	-0.31150400

Compound **39** (radical cation)
Energy: -28211.229514346014 eV

Atom	X (Å)	Y (Å)	Z (Å)
C	-4.14661800	-0.93157700	-0.17139400
C	-3.75858800	0.41608200	0.42508400
O	-2.39573100	0.23779200	0.88376400
C	-1.78830900	-0.80914300	0.15945700
P	-0.20695400	-0.14806700	-0.60811000
C	0.40051500	1.21667800	0.58168100
C	-0.22597600	2.57663800	0.26471400
C	0.79368200	3.16286600	-0.72079200
C	2.11667400	2.62217100	-0.17767900
O	1.78867300	1.35671100	0.46900700
C	1.00300900	-1.58890600	-0.54293400
C	2.39943400	-1.35334100	-1.14979300
C	3.31813200	-1.94087800	-0.06136700
C	2.55002400	-1.63343700	1.21813500
O	1.15106700	-1.80439900	0.84891700
C	-2.82888100	-1.37415200	-0.83042600
H	-4.96867800	-0.85900000	-0.88584300
H	-4.43470600	-1.62822800	0.62094500
H	-3.78555400	1.21777500	-0.32519700
H	-4.34932000	0.71936000	1.29069200
H	-1.42954100	-1.57421300	0.86595200
H	0.13738100	0.82468400	1.57053200
H	-1.24175600	2.50922500	-0.12714800
H	-0.26145700	3.16173900	1.18886600
H	0.77335700	4.25340500	-0.75521300
H	0.60865700	2.78995000	-1.73393000
H	2.55331400	3.27588400	0.58352000
H	2.86658200	2.42124000	-0.94600800
H	0.47900400	-2.44360200	-0.98585000
H	2.60003800	-0.28879300	-1.27648400
H	2.50284600	-1.84563600	-2.11783800
H	4.31189800	-1.48955600	-0.07011100
H	3.42774100	-3.02132000	-0.18967500
H	2.70133900	-0.60430500	1.55561000
H	2.72947100	-2.32603900	2.04226900
H	-2.72793400	-0.91478800	-1.81848200
H	-2.73903100	-2.45561800	-0.94658500

Compound **40** (radical cation)
Energy: -40097.915933331395 eV

Atom	X (Å)	Y (Å)	Z (Å)
C	3.58352400	-3.03368300	-1.27450900
C	4.13565500	-1.79422000	-0.54874000
C	5.01768400	-2.19448000	0.64608300
C	3.00822200	-0.87088300	-0.13040100

C	2.05901900	-1.26868700	0.80890800
C	1.00649900	-0.42971200	1.19102000
C	0.25303600	-0.70796300	2.48563000
C	0.94215300	0.07910300	3.62163300
C	0.15573300	-2.19217800	2.85274400
C	0.84724900	0.83065600	0.52404400
C	-0.41024100	1.60136300	0.74109100
C	-0.42047000	2.86709300	1.34168200
C	-1.61199100	3.56057300	1.53769900
C	-2.82592900	3.00555700	1.12821600
C	-2.84447000	1.74601200	0.53756300
C	-1.64710700	1.04026300	0.33501400
P	-1.62021700	-0.61437800	-0.42031500
C	-3.05062000	-1.69482200	0.21341200
C	-4.36425100	-1.46490300	-0.55944000
C	-3.28003900	-1.40415500	1.70766200
C	-2.61349600	-3.16698400	0.05507900
C	-1.55647500	-0.43477200	-2.30562600
C	-1.64150600	-1.81635300	-2.97194900
C	-0.20602300	0.21060300	-2.64421000
C	-2.68678800	0.49351300	-2.79115900
C	1.85933100	1.29294200	-0.34924500
C	1.86082700	2.69673400	-0.94423000
C	2.31650200	2.75979100	-2.41179200
C	2.76141700	3.61469600	-0.09201900
C	2.91174000	0.42570400	-0.65473400
H	2.95539600	-3.63285100	-0.60682500
H	4.40291600	-3.66949200	-1.62166200
H	2.98047700	-2.75047200	-2.14277500
H	4.76307900	-1.24098300	-1.25782100
H	5.85962000	-2.80486200	0.30753800
H	4.45436000	-2.78360700	1.37702300
H	5.41700600	-1.31424500	1.15752300
H	2.16871100	-2.23004000	1.29535800
H	-0.75739300	-0.30358500	2.39802600
H	0.94241700	1.15311500	3.41887200
H	0.41931700	-0.08873400	4.56787900
H	1.98017500	-0.24566400	3.74077000
H	-0.21641700	-2.79750700	2.02145800
H	1.12595900	-2.59826700	3.15271700
H	-0.51963000	-2.32427800	3.70258000
H	0.51444200	3.30007700	1.67523600
H	-1.59403600	4.53447900	2.01538400
H	-3.75299700	3.55151700	1.26569900
H	-3.78934000	1.32519400	0.21582500
H	-5.13004700	-2.10988300	-0.11497800
H	-4.72397200	-0.43640200	-0.48925500
H	-4.28434200	-1.73514400	-1.61268800
H	-4.08730100	-2.05558400	2.05715900
H	-3.57850700	-0.37240900	1.89747000
H	-2.39857700	-1.62695500	2.30829200

H	-1.69085100	-3.37733900	0.60011000
H	-3.39863800	-3.80747800	0.46928300
H	-2.46972300	-3.45404700	-0.98695500
H	-0.86475200	-2.49361600	-2.60624200
H	-2.61435900	-2.29167900	-2.83824400
H	-1.48838500	-1.68760200	-4.04813500
H	-0.12654600	1.20755500	-2.21146700
H	0.63900200	-0.38794800	-2.29916800
H	-0.13534500	0.31201500	-3.73214400
H	-3.68123500	0.08401900	-2.61626500
H	-2.62519400	1.47945900	-2.32512700
H	-2.56972000	0.62788300	-3.87238700
H	0.83958600	3.08722900	-0.89775100
H	2.16881000	3.77231800	-2.79738200
H	1.76071400	2.07068400	-3.05116900
H	3.38110700	2.53133600	-2.51616400
H	3.80257100	3.28107900	-0.14612700
H	2.47547900	3.61428200	0.96296200
H	2.71658700	4.64397900	-0.45971100
H	3.67772500	0.75527300	-1.34855100

Compound **41** (radical cation)
Energy: -28327.513588685513 eV

Atom	X (Å)	Y (Å)	Z (Å)
C	2.69708800	-0.45303900	-2.24440400
C	2.70016100	-0.86807500	-0.76520500
C	3.20127600	-2.30529100	-0.56508600
P	1.00116500	-0.66334700	-0.05449400
C	0.51926000	1.07404600	-0.13831500
C	1.46864400	2.10754000	-0.06082100
C	1.04921300	3.43265000	-0.01578800
C	-0.31584300	3.73588400	-0.05781900
C	-1.26180200	2.72018100	-0.15828300
C	-0.86255400	1.37857000	-0.22466600
C	-1.84527800	0.29027200	-0.38767900
C	-2.99830800	0.23215500	0.40880800
C	-3.85988300	-0.85637300	0.31815200
C	-3.59207600	-1.90425300	-0.57565200
C	-2.47609000	-1.84334800	-1.40278000
C	-1.61181900	-0.74480600	-1.32593700
C	0.95377200	-1.27662900	1.70177000
C	1.99690800	-0.51772100	2.54151000
C	-0.45296800	-1.18834700	2.30012600
H	3.71596500	-0.50814800	-2.63714600
H	2.33589900	0.56804700	-2.38793600
H	2.07450500	-1.12783000	-2.83969900
H	3.34722100	-0.18360800	-0.19932700
H	2.54518000	-3.02925000	-1.05786900
H	4.19472800	-2.40261600	-1.01113600
H	3.28688600	-2.57604900	0.49002000

H	2.52733700	1.88107100	-0.00267200
H	1.78243300	4.22778800	0.06444000
H	-0.64004300	4.77069700	-0.02811300
H	-2.31661200	2.96239400	-0.23049000
H	-3.18828400	1.01835000	1.13190900
H	-4.73682500	-0.90182700	0.95530300
H	-4.27081500	-2.74807100	-0.63792700
H	-2.29120700	-2.62342100	-2.13330700
H	-0.83470300	-0.62296200	-2.07304800
H	1.24283000	-2.33032800	1.61852500
H	3.01376900	-0.63796800	2.15977900
H	1.97750100	-0.91242200	3.56224700
H	1.76304500	0.54935600	2.58829500
H	-0.43597300	-1.61800900	3.30564900
H	-0.78126800	-0.14964800	2.38595800
H	-1.18723800	-1.73582100	1.70636200

Compound **42** (radical cation)
Energy: -40739.99210293336 eV

Atom	X (Å)	Y (Å)	Z (Å)
C	2.74064900	-4.60934900	-1.14150200
C	2.79078000	-3.28590400	-1.63314200
C	1.89649200	-2.33507700	-1.18962800
C	0.91037800	-2.65810100	-0.22580700
C	-0.04015600	-1.71110000	0.29577500
P	-0.00014500	0.00003300	-0.23336600
C	1.50172800	0.82077700	0.29602200
C	2.34815600	0.16959600	1.19253800
C	3.52486100	0.78793900	1.64395300
C	3.86123200	2.04771000	1.19013200
C	3.04625300	2.73754900	0.25829100
C	3.40506500	4.02579000	-0.21733700
C	2.62275500	4.67730100	-1.14231800
C	1.45203900	4.05870500	-1.63479800
C	1.07525600	2.80907800	-1.19095100
C	1.84710200	2.11714100	-0.22599000
C	-1.46196800	0.89029000	0.29595700
C	-1.32127100	1.94935400	1.19193400
C	-2.44519600	2.65917100	1.64333500
C	-3.70438900	2.32023600	1.18982500
C	-3.89426100	1.26925500	0.25827200
C	-5.18930400	0.93554500	-0.21717000
C	-5.36227200	-0.06792800	-1.14194000
C	-4.24110700	-0.77231300	-1.63448300
C	-2.97053100	-0.47351600	-1.19076800
C	-2.75732300	0.54102100	-0.22590800
C	-1.02792500	-2.11893600	1.19143100
C	-1.08104000	-3.44735600	1.64223100
C	-0.15791100	-4.36835100	1.18862400
C	0.84771600	-4.00704500	0.25772000

C	1.78438400	-4.96163700	-0.21763400
H	3.45193900	-5.34497200	-1.50085500
H	3.53769500	-3.01646100	-2.37242000
H	1.94754800	-1.32997600	-1.59495600
H	2.08691100	-0.81340700	1.56594000
H	4.15720200	0.27480300	2.35989600
H	4.76923700	2.52887300	1.54091100
H	4.31270600	4.48524500	0.16138400
H	2.90455600	5.66089500	-1.50195900
H	0.84600300	4.57040400	-2.37500000
H	0.17975800	2.35044900	-1.59702400
H	-0.33931600	2.21497800	1.56496400
H	-2.31698600	3.46364000	2.35896900
H	-4.57513000	2.86597800	1.54057000
H	-6.04109300	1.49178500	0.16150500
H	-6.35498100	-0.31593000	-1.50143600
H	-4.38116600	-1.55313000	-2.37456600
H	-2.12552100	-1.01960000	-1.59684600
H	-1.74889600	-1.40134300	1.56453400
H	-1.84214000	-3.73874600	2.35746300
H	-0.19544800	-5.39543500	1.53895100
H	1.72815500	-5.97761600	0.16047400

Compound **43** (radical cation)
Energy: -36887.68161760058 eV

Atom	X (Å)	Y (Å)	Z (Å)
C	3.63305800	-2.52840000	-0.63219800
C	3.25333100	-1.27969500	0.18153100
C	4.53088300	-0.66500600	0.79047100
C	2.50860100	-0.21581800	-0.61755300
C	2.92000300	0.06359800	-1.92457600
C	2.30750300	1.05766900	-2.68995800
C	1.32166400	1.85596500	-2.12869600
C	0.89901800	1.64750100	-0.80734200
C	0.13024200	2.74333100	-0.08012000
C	1.14762600	3.63795000	0.66099200
C	-0.76217900	3.59672700	-0.98763100
C	1.43480600	0.53621100	-0.08116700
C	0.81712700	0.17712000	1.22999200
C	1.51961200	0.27476900	2.43767400
C	0.91925200	-0.05744500	3.64972000
C	-0.40305700	-0.50382600	3.68230400
C	-1.12775600	-0.59881900	2.49859100
C	-0.52802600	-0.26713100	1.27203600
P	-1.45207800	-0.36344600	-0.28953900
C	-1.34096100	-2.12173300	-0.98090200
C	-1.78861200	-3.13561700	0.09117300
C	-2.18588600	-2.24689500	-2.25759700
C	0.13504000	-2.37490600	-1.31588900
C	-3.21924200	0.31004300	-0.11347500

C	-4.21094700	-0.75327800	0.39916100
C	-3.21210000	1.50925100	0.85129800
C	-3.66115400	0.79516600	-1.51096400
H	4.04710900	-3.28837900	0.03612400
H	4.40210000	-2.30737600	-1.37797900
H	2.77783100	-2.96546400	-1.15167000
H	2.60836300	-1.60262600	1.00469500
H	4.32063600	0.22538400	1.38821700
H	5.22358100	-0.36691800	-0.00286300
H	5.04000300	-1.39398600	1.42763600
H	3.72307300	-0.51760400	-2.36273400
H	2.63537500	1.23310100	-3.70945100
H	0.91221700	2.68059700	-2.69897000
H	-0.49397500	2.28317200	0.68843400
H	0.62637900	4.43085500	1.20554000
H	1.83814600	4.10527500	-0.04744200
H	1.73586400	3.06161600	1.37958100
H	-1.38976300	4.25646400	-0.38203100
H	-0.17131000	4.23938900	-1.64635400
H	-1.41501200	2.98541500	-1.61711600
H	2.54150300	0.63284700	2.42481200
H	1.48328800	0.03682100	4.57164500
H	-0.86731400	-0.77752300	4.62353300
H	-2.15173200	-0.95115100	2.53021500
H	-1.63718500	-4.14108600	-0.31722400
H	-1.18833100	-3.05248300	0.99991100
H	-2.84173800	-3.04270200	0.35482300
H	-1.89764100	-1.50956200	-3.01172700
H	-3.25628100	-2.15624000	-2.06709200
H	-2.01502900	-3.24025300	-2.68430000
H	0.22547500	-3.38523400	-1.72784400
H	0.52226000	-1.66814100	-2.05181200
H	0.75599400	-2.32380900	-0.42177800
H	-5.19230600	-0.27525900	0.49014200
H	-3.94521000	-1.14146600	1.38453200
H	-4.32165100	-1.59179500	-0.28897300
H	-2.89402800	1.24014800	1.85929600
H	-4.23464200	1.89451700	0.91485200
H	-2.57998200	2.32053000	0.49183400
H	-2.99036500	1.56120200	-1.90558700
H	-4.65819900	1.23819600	-1.42307700
H	-3.72339600	-0.01515500	-2.23795400

Compound **44** (radical cation)
Energy: -34682.073149589654 eV

Atom	X (Å)	Y (Å)	Z (Å)
C	2.43491200	-1.59043400	-3.16277700
C	3.40825700	-1.21882000	-2.22518000
C	3.10365300	-1.18730500	-0.86823600
C	1.81490400	-1.51872000	-0.42286300

C	1.45082400	-1.39046000	1.00242400
C	2.28057000	-1.90562900	2.00735400
C	1.90988800	-1.82662600	3.34635000
C	0.70088300	-1.22628600	3.71171800
C	-0.13136000	-0.69215800	2.73470100
C	0.24615300	-0.74440700	1.38083700
P	-0.74246900	0.06071200	0.10315900
C	-2.50869600	0.15694200	0.66526600
C	-3.35528400	0.98986300	-0.31654600
C	-3.53663300	0.27958000	-1.66355700
C	-4.13710400	-1.11934000	-1.47681700
C	-3.30375600	-1.96706900	-0.50776500
C	-3.09489000	-1.25982600	0.83778500
C	-0.10446000	1.75671800	-0.27923200
C	1.33972900	1.75510600	-0.80411900
C	1.79137900	3.18652800	-1.12714500
C	1.64766500	4.10763900	0.08921500
C	0.21266400	4.09556400	0.62453700
C	-0.25842700	2.67038300	0.96054900
C	0.85639200	-1.94284900	-1.37009200
C	1.16434100	-1.96223700	-2.73387200
H	2.68101000	-1.61113200	-4.21906300
H	4.40210900	-0.94038500	-2.55961500
H	3.84859900	-0.85968300	-0.15057700
H	3.19995400	-2.40742300	1.72551100
H	2.55629700	-2.24839000	4.10870700
H	0.41453100	-1.16561000	4.75605000
H	-1.05480500	-0.20790000	3.03037700
H	-2.47665100	0.66553200	1.63818700
H	-4.33656400	1.13226500	0.15234400
H	-2.93561400	1.99172800	-0.44989800
H	-4.17083800	0.88613800	-2.31688700
H	-2.56301600	0.19514800	-2.17083200
H	-5.15693800	-1.02043800	-1.08285900
H	-4.22389300	-1.62648900	-2.44263900
H	-3.77915900	-2.93789700	-0.33929500
H	-2.32683700	-2.17898400	-0.96604600
H	-4.06132500	-1.14703800	1.34381000
H	-2.46594400	-1.86231600	1.50061700
H	-0.76729100	2.11680300	-1.07632300
H	1.42279000	1.11615600	-1.68714800
H	1.99861900	1.33286800	-0.03755300
H	1.18932300	3.57677000	-1.95829000
H	2.82905100	3.16608100	-1.47478300
H	2.33319600	3.77531400	0.88024300
H	1.94152700	5.12884900	-0.17198000
H	0.12776300	4.71563800	1.52305000
H	-0.46410600	4.52900000	-0.12317600
H	0.34684900	2.26213500	1.77884800
H	-1.29763700	2.69372600	1.30314800
H	-0.08474100	-2.35873900	-1.02800400

H	0.42115600	-2.29934300	-3.44852900

Compound **45** (radical cation)
Energy: -54578.46933095534 eV

Atom	X (Å)	Y (Å)	Z (Å)
C	-3.99745700	-1.06937300	0.69325100
C	-3.75914400	-0.10824500	-0.47119300
S	-2.22826700	-0.72649400	-1.28196600
C	-1.56872200	-1.45147200	0.27248600
P	0.06669800	-0.60928000	0.68345400
C	-0.29647900	1.22297000	0.57847400
C	0.52659300	2.09345500	1.55204400
C	0.47055500	3.52226900	1.00402400
C	0.80312000	3.43148400	-0.48688700
S	-0.04175500	1.90751700	-1.09667100
C	1.22748400	-1.09058000	-0.68227900
C	1.70162700	-2.54499500	-0.50484500
C	2.75709400	-2.54413800	0.60549100
C	3.73439600	-1.40977800	0.29160300
S	2.73969100	-0.04075200	-0.46744600
C	-2.64031500	-1.27393200	1.37752700
H	-4.37899200	-2.02074400	0.31132500
H	-4.73888700	-0.66467100	1.38811700
H	-3.60355400	0.92352900	-0.14118400
H	-4.55136100	-0.11558300	-1.22278100
H	-1.33671300	-2.50203700	0.09044300
H	-1.35995200	1.29875400	0.82405100
H	0.12030900	2.01419400	2.56388800
H	1.56066500	1.74071900	1.57100700
H	-0.53117200	3.94371800	1.13718400
H	1.17949200	4.17637200	1.51889100
H	0.43713200	4.27776800	-1.07183400
H	1.87419800	3.31167300	-0.66849900
H	0.80104000	-0.90992500	-1.66980500
H	0.87285600	-3.22673800	-0.28534100
H	2.14490100	-2.87199600	-1.45074200
H	2.28064000	-2.36633600	1.57653300
H	3.27968700	-3.50260200	0.66735600
H	4.24399600	-1.02807100	1.17705600
H	4.48251400	-1.71271600	-0.44422600
H	-2.40845100	-0.39716200	1.99211200
H	-2.63603500	-2.13927900	2.04379800

Compound **46** (radical cation)
Energy: -93393.63818742383 eV

Atom	X (Å)	Y (Å)	Z (Å)
C	1.23537900	-3.58876600	-0.72732600
C	0.37743400	-2.33178400	-0.90491900

N	1.08521300	-1.20739200	-1.53339600
C	1.66776000	-1.45725600	-2.85043100
P	1.81109900	-0.16538200	-0.41409900
C	1.98723300	1.36282500	-1.38010600
C	3.07684500	2.22999400	-1.22339000
C	3.10542200	3.44656400	-1.90540600
C	2.05068200	3.80323200	-2.74589600
C	0.96501700	2.93888500	-2.90901100
C	0.92648000	1.72515800	-2.22675400
C	3.51007400	-0.75637500	-0.08702400
C	3.81429300	-1.37836400	1.13241000
C	5.06963200	-1.94563000	1.34213700
C	6.03316800	-1.89933300	0.33339800
C	5.73969000	-1.28336800	-0.88435700
C	4.48513300	-0.71486700	-1.09756900
C	-0.19010600	-1.82508900	0.39603600
C	0.22748100	-2.18692300	1.70657000
C	-0.69499100	-1.60850600	2.62827600
C	-1.59838500	-0.82225700	1.90853700
C	-1.26533000	-0.87929400	0.51290400
P	-2.16563300	-0.13885600	-0.90895600
C	-3.65503700	-1.22243400	-0.95298300
C	-3.71927900	-2.46679300	-0.31296300
C	-4.85065400	-3.27345000	-0.44861500
C	-5.93012500	-2.84505000	-1.21925000
C	-5.87346500	-1.60673100	-1.86308900
C	-4.74177000	-0.80538400	-1.73896800
C	-2.82149200	1.38271200	-0.09379700
C	-2.19304500	2.60132300	-0.38689700
C	-2.58898700	3.77734900	0.25171300
C	-3.62726300	3.74886800	1.18395500
C	-4.27878500	2.54434000	1.46221000
C	-3.88336200	1.36928900	0.82291800
Fe	0.48830100	-0.03077000	1.41690900
C	-0.26735000	1.84782800	2.35235900
C	0.75521200	2.07224800	1.39824100
C	1.94357900	1.43226400	1.88964600
C	1.61822900	0.77438500	3.10374400
C	0.26769800	1.08206100	3.41158500
H	2.12462000	-3.38352500	-0.12529200
H	0.65592000	-4.37893800	-0.24286900
H	1.56626900	-3.96725000	-1.69647900
H	-0.46972600	-2.57804000	-1.55951100
H	0.90723000	-1.92908300	-3.47926700
H	1.94821900	-0.51136100	-3.31484100
H	2.55039600	-2.10732600	-2.81421500
H	3.91136600	1.95414100	-0.58791200
H	3.95424200	4.11115000	-1.78339400
H	2.07659500	4.74897500	-3.27709600
H	0.14770600	3.20968200	-3.56974900
H	0.08205300	1.05294700	-2.34103500

H	3.06447300	-1.41705500	1.91455200
H	5.29660900	-2.42088100	2.29090600
H	7.01117200	-2.33993700	0.49636100
H	6.48852900	-1.24332600	-1.66856400
H	4.27087000	-0.22828100	-2.04264100
H	1.01729200	-2.88177000	1.94914400
H	-0.65164400	-1.71098500	3.70336500
H	-2.37831000	-0.20428600	2.32649800
H	-2.89220600	-2.81044900	0.29937300
H	-4.88861700	-4.23451200	0.05477200
H	-6.81050000	-3.47131500	-1.32010200
H	-6.71020700	-1.26727400	-2.46529300
H	-4.70636000	0.15499700	-2.24606700
H	-1.38793100	2.62942800	-1.11374000
H	-2.09403800	4.71434600	0.01608500
H	-3.93925300	4.66249800	1.67958100
H	-5.10054900	2.52275300	2.17120900
H	-4.40677900	0.44009800	1.02445200
H	-1.28929700	2.18401000	2.25673900
H	0.67103400	2.67033200	0.50602100
H	2.92862400	1.48069000	1.45306100
H	2.29848000	0.19504800	3.71140000
H	-0.28267600	0.71979700	4.26717100

Compound **47** (radical cation)
Energy: -92323.65161279013 eV

Atom	X (Å)	Y (Å)	Z (Å)
C	0.08266000	-3.54546300	-1.86029700
C	0.23594900	-2.07353800	-1.47326200
N	1.63889500	-1.66099100	-1.50148200
P	2.07573400	-0.37233400	-0.50049200
C	2.07755400	1.15427800	-1.49696400
C	3.01991600	2.17917900	-1.32373500
C	2.89315700	3.37522800	-2.02959600
C	1.82513400	3.56142700	-2.90815900
C	0.88371000	2.54523000	-3.08531000
C	1.00567500	1.34823400	-2.38293000
C	3.81273200	-0.76097500	-0.12618400
C	4.82604200	-0.50819600	-1.06308400
C	6.13409400	-0.91473500	-0.80229100
C	6.43875300	-1.57487900	0.38853400
C	5.43232700	-1.83681400	1.32012100
C	4.12411600	-1.43393500	1.06363900
C	-0.23266100	-1.81080200	-0.06988900
C	0.33845700	-2.38729200	1.11192500
C	-0.47789300	-1.98319800	2.20755600
C	-1.47366500	-1.12015900	1.73037200
C	-1.29569800	-0.94581900	0.31635200
P	-2.30697300	-0.01302300	-0.90532100
C	-2.75098300	1.48493600	0.06760300

C	-2.09694100	2.68084000	-0.26097700
C	-2.33045600	3.84194800	0.47736500
C	-3.23042600	3.82050100	1.54379600
C	-3.90696100	2.63935100	1.86179200
C	-3.67297100	1.47822800	1.12527200
C	-3.86072400	-0.99967800	-0.84836000
C	-5.03974200	-0.41128200	-1.33356600
C	-6.22336500	-1.14311800	-1.38841100
C	-6.24428000	-2.47662100	-0.97439300
C	-5.07519000	-3.07245200	-0.50362500
C	-3.88916400	-2.33962000	-0.43948800
Fe	0.59306700	-0.27096100	1.19961800
C	-0.17921900	1.53762000	2.31031300
C	0.82041200	1.84954000	1.35026600
C	2.02276200	1.20310500	1.77367200
C	1.72983300	0.42700500	2.92869300
C	0.38343500	0.69618200	3.29053200
H	0.43451300	-3.70931300	-2.88315100
H	-0.97038900	-3.83604400	-1.81791700
H	0.65772400	-4.18730200	-1.18853400
H	-0.37940200	-1.46936400	-2.15240600
H	2.14440300	-1.79333400	-2.37105100
H	3.86175600	2.04414900	-0.65360300
H	3.63090700	4.15922700	-1.89429000
H	1.72887500	4.49313500	-3.45575400
H	0.05370500	2.68282600	-3.77073600
H	0.25233100	0.57986000	-2.51103600
H	4.59894400	0.00812000	-1.98976900
H	6.91423900	-0.71436700	-1.52922900
H	7.45833900	-1.88639300	0.58998000
H	5.66715700	-2.35437400	2.24441500
H	3.34018900	-1.64321600	1.78325500
H	1.14800800	-3.10183900	1.13892400
H	-0.31515400	-2.25484300	3.24137700
H	-2.20500100	-0.59790100	2.32812600
H	-1.39916700	2.69797100	-1.09323600
H	-1.81816400	4.76255200	0.21615200
H	-3.41600000	4.72311700	2.11690400
H	-4.62117500	2.62605800	2.67914500
H	-4.21706500	0.56870800	1.35871500
H	-5.03517900	0.62558700	-1.65734300
H	-7.12962800	-0.67283300	-1.75651800
H	-7.16642700	-3.04662200	-1.02010200
H	-5.08454500	-4.10832300	-0.17920000
H	-2.99071400	-2.81609000	-0.06116900
H	-1.20668500	1.86466200	2.26714400
H	0.71133400	2.51257100	0.50749100
H	3.00167300	1.31245200	1.33490700
H	2.43539700	-0.17695200	3.48019100
H	-0.14649900	0.25484700	4.12212200

Compound **48** (radical cation)
Energy: -54379.41860351298 eV

Atom	X (Å)	Y (Å)	Z (Å)
C	-0.50390100	3.83785900	0.32548600
C	0.56216200	3.79906100	-0.54712500
S	1.20909100	2.21491800	-0.74299400
C	0.02083400	1.56436500	0.36680200
P	-0.04454800	-0.13685300	0.83630700
C	-1.57938900	-0.85861300	0.34274000
C	-2.03848600	-2.12312700	0.69831600
C	-3.32290800	-2.40017500	0.18175700
C	-3.84127900	-1.34311700	-0.53486100
S	-2.77193900	0.00560700	-0.60531800
C	1.30730400	-1.05265400	0.16045700
C	1.23707300	-2.19957100	-0.62450300
C	2.50641300	-2.75650000	-0.88758900
C	3.52874500	-2.05348600	-0.28647500
S	2.96558400	-0.70105400	0.61692200
C	-0.80744100	2.56841100	0.86182600
H	-1.03978400	4.74579100	0.57124300
H	0.99823900	4.62360500	-1.09546300
H	-1.46695600	-2.81065300	1.31080000
H	-3.85105600	-3.33306300	0.33304200
H	-4.80009300	-1.28964200	-1.03336500
H	0.30335400	-2.59365700	-1.00507400
H	2.67003700	-3.62954600	-1.50667500
H	4.59095200	-2.25164600	-0.34116500
H	-1.59501700	2.38536100	1.58330800

Compound **49** (radical cation)
Energy: -44114.51193188426 eV

Atom	X (Å)	Y (Å)	Z (Å)
C	5.19802200	0.43944700	-2.21680600
C	3.74309600	0.90413100	-2.39405100
C	3.60287500	2.41158500	-2.11456700
C	2.78751000	0.10453800	-1.52950800
C	2.95456700	0.05395800	-0.13569700
C	2.07806000	-0.63355100	0.70292800
C	2.31220300	-0.63495100	2.20687700
C	3.30919100	-1.74628500	2.58702600
C	2.79546200	0.71934900	2.74622100
C	0.98205700	-1.30668100	0.11777000
C	-0.10796900	-1.87115200	0.96345200
C	0.00668800	-3.01326100	1.75733100
C	-1.08714100	-3.47018400	2.49510000
C	-2.30967900	-2.79334200	2.45578800
C	-2.44959100	-1.65137900	1.67138400
C	-1.34735300	-1.18446900	0.94180100

P	-1.35703000	0.27993100	-0.10523400
C	-3.03559300	0.72278400	-0.53934400
C	-3.46925200	2.06181700	-0.48804800
C	-4.76213300	2.38246400	-0.88736200
C	-5.62217000	1.38432300	-1.35438300
C	-5.18922700	0.05597800	-1.42749900
C	-3.89913000	-0.27742100	-1.03494900
C	-0.53352500	1.66433900	0.70653400
C	0.30156400	2.49250100	-0.05655000
C	0.90875000	3.59595300	0.53972600
C	0.68146900	3.87218600	1.88910100
C	-0.14039900	3.03824700	2.65279300
C	-0.74361000	1.92661000	2.06956600
C	0.81815300	-1.32126200	-1.30331500
C	-0.17430800	-2.26241000	-1.97420200
C	0.54785500	-3.58266000	-2.31279300
C	-0.85567900	-1.67113300	-3.21470600
C	1.72536200	-0.59611100	-2.09067300
H	5.55663800	0.62591900	-1.19938400
H	5.29977900	-0.63025000	-2.41939300
H	5.85495600	0.98179800	-2.90270000
H	3.45968400	0.73008200	-3.43901100
H	2.58412100	2.75881500	-2.31385800
H	3.84279200	2.64367600	-1.07162600
H	4.28450300	2.98320100	-2.75093300
H	3.78191300	0.59492800	0.30859100
H	1.35645000	-0.85814100	2.69137000
H	4.28473700	-1.55823000	2.12705000
H	2.97297000	-2.73126500	2.25246300
H	3.44765300	-1.78274900	3.67163700
H	2.79024600	0.70468000	3.83986300
H	2.15581400	1.53736900	2.41154600
H	3.82181900	0.93765100	2.43522800
H	0.94339300	-3.55793700	1.77867500
H	-0.98807000	-4.36736400	3.09720700
H	-3.15039700	-3.15479500	3.03810100
H	-3.39530100	-1.12232900	1.63366600
H	-2.80251600	2.83432400	-0.12176200
H	-5.10249800	3.41094200	-0.83199200
H	-6.62858300	1.64165400	-1.66736800
H	-5.85724600	-0.71467700	-1.79686300
H	-3.55931700	-1.30583600	-1.10291300
H	0.48281200	2.26427300	-1.10118100
H	1.55945200	4.23503300	-0.04739300
H	1.15180100	4.73402000	2.35089700
H	-0.30718400	3.25199900	3.70324500
H	-1.37484200	1.27197800	2.66138800
H	-0.95621000	-2.50862000	-1.25042900
H	-0.15434600	-4.29802000	-2.75090600
H	1.35466800	-3.41157600	-3.03187700
H	0.98376000	-4.03476600	-1.41737900

H	-0.14817500	-1.49965000	-4.03094100
H	-1.61282900	-2.36605600	-3.58861100
H	-1.34703500	-0.71926800	-2.99009400
H	1.61328500	-0.60352900	-3.16946900

Compound **50** (radical cation)
Energy: -30467.513322670453 eV

Atom	X (Å)	Y (Å)	Z (Å)
C	-1.70417700	-2.30804200	1.78645100
C	-1.03101200	-0.92761300	1.74691200
C	-1.84634700	0.11344800	2.53821300
C	0.39241200	-1.02371000	2.32653400
P	-0.84426700	-0.36113600	-0.04076100
C	-0.10241400	1.29023400	-0.07762700
C	-0.86448000	2.45885800	0.08246700
C	-0.23383900	3.69805900	0.14182100
C	1.15806500	3.77807000	0.04223500
C	1.92008600	2.62520200	-0.12469800
C	1.30475700	1.36893700	-0.20437300
C	2.09111900	0.13257600	-0.39901600
C	3.20661500	-0.14750200	0.40087000
C	3.85996100	-1.37273500	0.29225100
C	3.41427900	-2.33842600	-0.62073300
C	2.33196900	-2.06201200	-1.44954500
C	1.67791600	-0.82808700	-1.35168800
C	-2.33643500	-0.50126100	-1.18170600
C	-2.43428700	-1.97611200	-1.62530400
C	-3.64357000	-0.06355800	-0.49443200
C	-2.06130500	0.38087500	-2.41350300
H	-2.73895400	-2.27883300	1.43933300
H	-1.15453700	-3.04773200	1.19808700
H	-1.71846900	-2.65435400	2.82453300
H	-2.87040400	0.21026100	2.17400200
H	-1.89494000	-0.21794700	3.58140200
H	-1.37101700	1.09664100	2.52320100
H	0.89983200	-0.05684700	2.32062300
H	1.00829800	-1.74400800	1.78341700
H	0.31616100	-1.35446100	3.36712600
H	-1.94184700	2.40253500	0.18855500
H	-0.82448000	4.59762900	0.27703500
H	1.64837800	4.74477700	0.08603300
H	2.99784600	2.69319400	-0.22784000
H	3.53091800	0.57912400	1.13855500
H	4.71143700	-1.58543700	0.93013200
H	3.92956300	-3.28992000	-0.69657100
H	2.01346200	-2.78306300	-2.19479100
H	0.93584800	-0.55637000	-2.09515600
H	-3.26144700	-2.07144800	-2.33571400
H	-1.52151700	-2.30693300	-2.12831400
H	-2.63383000	-2.65212000	-0.79256500

H	-3.61254200	0.97522600	-0.15781900
H	-4.45625100	-0.14846900	-1.22391600
H	-3.89908100	-0.69912200	0.35521800
H	-2.00935600	1.44205000	-2.16636300
H	-2.88052900	0.23793500	-3.12494400
H	-1.13442400	0.10152500	-2.92260800

Compound **51** (radical cation)
Energy: -28012.487387263984 eV

Atom	X (Å)	Y (Å)	Z (Å)
C	1.48697800	3.49138700	0.51653400
C	2.55424600	2.78593800	0.02429000
O	2.16805400	1.54781900	-0.37983100
C	0.82348400	1.44855800	-0.11362100
P	-0.00001800	0.00001500	-0.70722700
C	-1.66632300	-0.01116300	-0.11379700
C	-2.46318900	-1.00724600	0.41589100
C	-3.76707000	-0.45796500	0.51680400
C	-3.68990400	0.81897400	0.02436200
O	-2.42465800	1.10356900	-0.38005100
C	0.84272800	-1.43744400	-0.11368200
C	2.10376400	-1.62946000	0.41609300
C	2.28020600	-3.03329400	0.51665400
C	1.13575600	-3.60496600	0.02426500
O	0.25659700	-2.65156300	-0.38007000
C	0.35933900	2.63680100	0.41590900
H	1.51043700	4.49704900	0.90878600
H	3.60334100	3.01120000	-0.09083500
H	-2.13871900	-1.99725300	0.69829000
H	-4.64961000	-0.94042300	0.90939200
H	-4.40956500	1.61487400	-0.09065100
H	2.79880700	-0.85339900	0.69854600
H	3.13939800	-3.55635100	0.90904100
H	0.80642200	-4.62617500	-0.09091800
H	-0.66028600	2.85093800	0.69814100

References

1. Matlantis (https://matlantis.com/), software as a service style material discovery tool.
2. S. Takamoto, D. Okanohara, Q.-J. Li, and J. Li, "Towards universal neural network interatomic potential", *J. Mater.*, **9** (3), 447–454 (2023). DOI: 10.1016/j.jmat.2022.12.007
3. L. Martínez, R. Andrade, E. G. Birgin, J. M. Martínez, "PACKMOL: A package for building initial configurations for molecular dynam-ics simulations", *J. Comput. Chem.*, **30** (13), 2157–2164 (2009). DOI: 10.1002/jcc.21224
4. T. Gensch, G. Dos Passos Gomes, P. Friederich, E. Peters, T. Gaudin, R. Pollice, K. Jorner, A. Nigam, M. Lindner-D'Addario, M. S. Sigman and A. Aspuru-Guzik, "A Comprehensive

Discovery Platform for Organophosphorus Ligands for Catalysis", *J. Am. Chem. Soc.*, **144** (3), 1205–1217 (2022). DOI: 10.1021/jacs.1c09718

5. H. Clavier and S. P. Nolan, "Percent buried volume for phosphine and N-heterocyclic carbeneligands: steric properties in organo-metallic chemistry", *Chem. Commun*., **46**, 841–861 (2010). DOI: 10.1039/B922984A
6. (49) S. H. Newman-Stonebraker, S. R. Smith, J. E. Borowski, E. Peters, T. Gensch, H. C. Johnson, M. S. Sigman, and A. G. Doyle, "Univariate classification of phosphine ligation state and reactivi-ty in cross-coupling catalysis", *Science*, **374** (6565), 301–308 (2021). DOI: 10.1126/science.abj4213
7. M. J. Frisch, G. W. Trucks, H. B. Schlegel et al., Gaussian 16 Revi-sion C.01; Gaussian, Inc., Wallingford CT, 2016. https://gaussian.com/
8. Landrum, G. RDKit: Open-source cheminformatics software, 2016. https://github.com/rdkit/rdkit/releases/tag/Release_2016_09_4
